# Application of 2D EXSY and qNMR Spectroscopy for Diastereomeric Excess Determination Following Chiral Resolution of β‐Lactams

**DOI:** 10.1002/open.202200119

**Published:** 2022-07-25

**Authors:** Eavan C. McLoughlin, John E. O'Brien, Cristina Trujillo, Mary J. Meegan, Niamh M. O'Boyle

**Affiliations:** ^1^ School of Pharmacy and Pharmaceutical Sciences Panoz Institute and Trinity Biomedical Sciences Institute 152-160 Pearse Street Trinity College Dublin Dublin 2 Ireland; ^2^ School of Chemistry Trinity College Dublin Dublin 2 Ireland; ^3^ Trinity Biomedical Sciences Institute School of Chemistry Trinity College Dublin Dublin 2 Ireland

**Keywords:** chiral resolution, chirality, NMR spectroscopy, liquid chromatography, structure elucidation

## Abstract

*Trans‐*β‐lactam isomers have garnered much attention as anti‐cancer microtubule targeting agents. Currently available synthetic methods are available for the preparation of enantiopure β‐lactams and favour isomeric *cis/trans* β‐lactam mixtures. Indirect chiral resolution offers the opportunity for isolation of exclusively enantiopure *trans‐*β‐lactams. In this study, liquid chromatography chiral resolution of β‐lactams derivatized as diastereomer mixtures with a panel of *N*‐protected amino acids is explored, where *N‐(*Boc)‐L‐proline served as the optimal chiral derivatising reagent. High‐performance liquid chromatography failed to adequately determine diastereomeric excess (*de*) of resolved diastereomers. Variable temperature, ^1^H NMR and 2D EXSY spectroscopic analyses of proline‐derivatised diastereomers were successfully employed to characterise equilibrating rotamers of resolved diastereomers and determine their *de*. Integration of resolved resonances corresponding to H_3_ and H_4_ of the β‐lactam ring served as a quantitative qNMR tool for the calculation of *de* following resolution.

## Graphical abstract

**Equilibrating rotamers** for resolved L‐proline‐derivatized β‐lactam diastereomers **3DS1** and **3DS2** characterised through qNMR methods following resolution, thereby allowing determination of *de*.

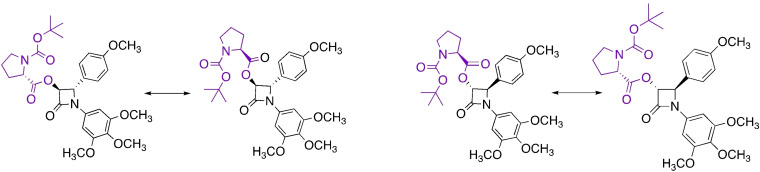

## Introduction

**Scheme 1:**
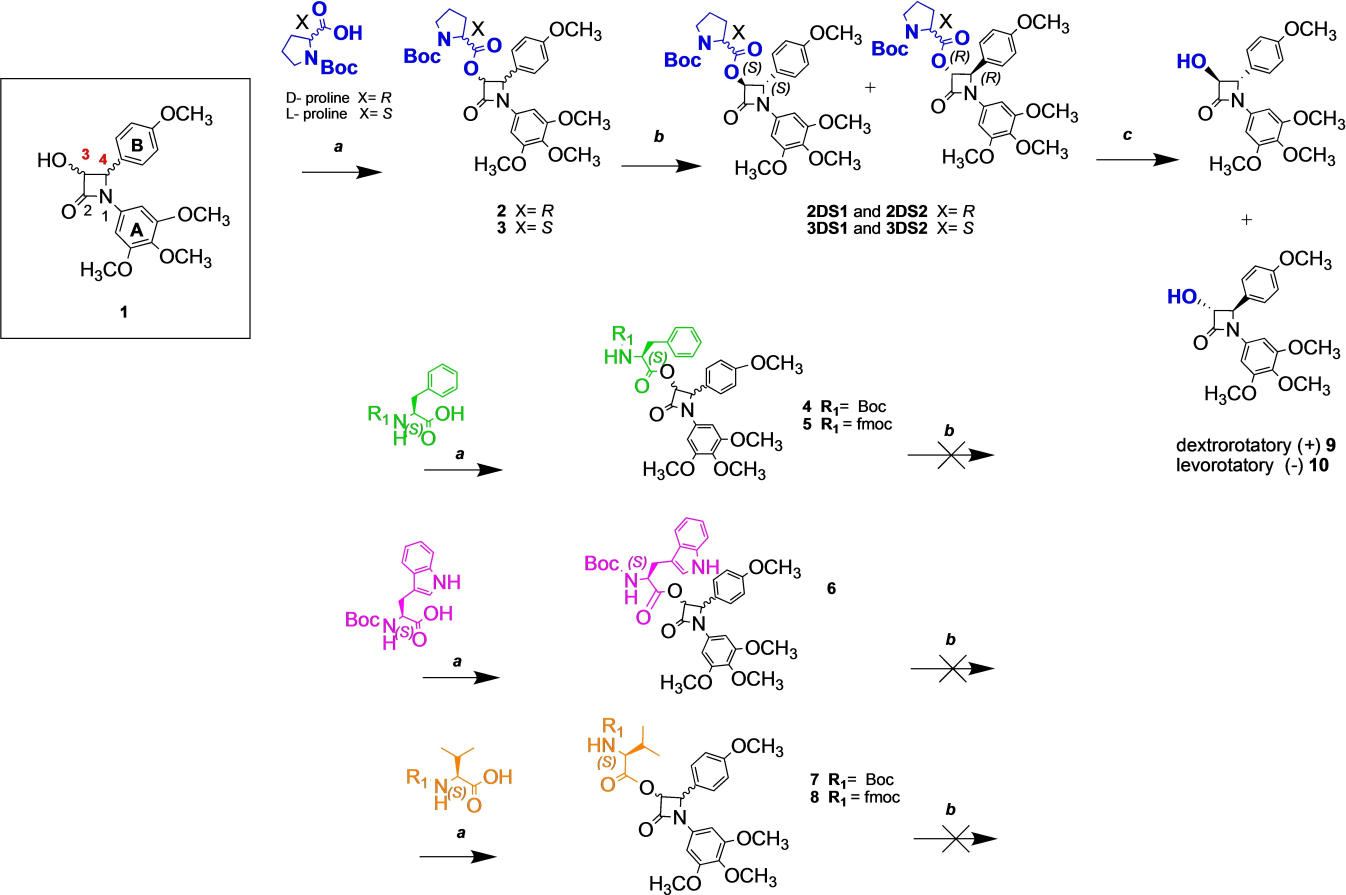
Synthesis and resolution of diastereomers of β‐lactam 1 by coupling with amino acid CDRs. Reagents and conditions: **a**: β‐lactam **1** (1 eq), amino acid CDR (2.2 eq), HBTU (2 eq), DIPEA (30 eq), anhydrous MeCN (30 mL), N_2_, rt, 24 hr. Yield=31–77 %. **b**: resolution of diastereomers using gravity and gradient elution of MTBE: *n‐*hexane (2 : 8–2 : 1) (relative stereochemistry shown). **c**: Hydrazine dihydrochloride (5 eq), triethylamine (TEA) (9 eq), methanol (MeOH) (30 mL) at 0 °C, then reflux for 6 h.

Microtubule‐targeting β‐lactams are of interest for development as anti‐cancer agents for targeting the colchicine binding site of tubulin.^[1]^ 3,4‐*Trans‐substituted‐*β‐lactam isomers have garnered much attention as the more optimal configuration for colchicine binding site activity.^[2]^ Initially reported by ourselves and others as racemates,^[3]^ resolution and biological evaluation of *trans*‐3‐hydroxyl substituted β‐lactams by Tripodi has demonstrated that the (+) enantiomer of *trans*‐3‐hydroxyl β‐lactams demonstrates superior antiproliferative potency.^[4]^ In the context of future drug development opportunities for 3‐hydroxyl β‐lactams, it is essential to prepare specifically the *trans‐*enantiopure analogues for comparative biological evaluation. Direct or indirect techniques are options for resolution of enantiomeric mixtures with direct methods employing chiral chromatographic separations, while indirect methods separate enantiomers using optically active chiral derivatising reagents (CDRs).^[5]^ While direct chiral chromatographic separations using preparative high performance liquid chromatography (HPLC) are proven as one of the best approaches for enantiomer separations, this strategy is often limited, by high costs of instrumentation and sample loading limitations.^[6]^ In contrast, indirect techniques remain an economical method for small scale preparative isolation of enantiopure products for preliminary in vitro evaluation.^[7]^ Asymmetric catalysis yields optically pure *cis*/*trans* β‐lactam isomeric mixtures, where the *cis* isomer is a less potent anti‐proliferative agent than the desirable enantiopure *trans* isomer.[^1^, ^8^] In this study we have pursued an alternative method for isolation of the enantiopure *trans* isomers of β‐lactam **1** (Scheme 1), employing liquid chromatography (LC) chiral resolution of *trans* β‐lactams racemates as the corresponding diastereomer derivatives using a series of amino acids as CDRs.^[4]^ Optimal selection of the CDR determines the efficacy of chiral resolution and there is a plethora of data on the use of CDRs for indirect separation of enantiomers using HPLC.^[9]^ Indirect chiral resolution using CDRs is extensively reported for the enantiopure isolation of L‐amino acids[^10^, ^11^] and indeed amino acids, peptide and peptidomimetic have been employed as chiral selectors for enantioresolution as chiral mobile phase additives for TLC and HPLC.^[12]^ However, the use and selection of amino acids as CDRs themselves for chiral resolution is not as widely reported.^[4]^ Considering our previous work demonstrating augmented solubility of β‐lactam racemates via amino acid prodrug formation,^[13]^ a series of *N‐*protected amino acids including *N‐*Boc*‐* l *‐*proline was chosen as CDRs for derivatization of β‐lactam **1**. Dual aims of trialling a panel of amino acid analogues included in situ LC chiral resolution of enantiopure β‐lactams of **1** as derivatised amino acid prodrugs.^[13]^ Resolving powers amongst the panel of *N*‐protected amino acid CDRs chosen differed significantly. Diastereomer mixtures of **1** (**2**–**8**, Scheme 1) were not readily resolved on achiral reverse phase HPLC and therefore HPLC could not readily be employed as a method to determine diastereomer purity or excess (*de)* ^[14]^ following LC chiral resolution. Resolution was not achieved for the majority of diastereomer mixtures with the exception of d‐ and l‐proline‐derivatized **1**. ^1^H NMR spectroscopy typically serves as a robust and reliable primary analytical tool for acquisition of purity, orthogonal to HPLC,^[15]^ particularly as HPLC is associated with drawbacks such as inability to detect inorganic impurities, water content, solvent residues and co‐eluting compounds with similar retention times to analyte in question.^[16]^ NMR using chiral solvating reagents (CSAs), for example, chiral lanthanide shift reagents, is widely reported in particular by Wenzel et al., for enantiodifferentiation, *ee* calculations and absolute configuration determination^[17]^ and involves non‐covalent association of analyte enantiomers with the CSA.^[18]^ Additionally, quantitative NMR (qNMR) is a well‐established method for purity determination to provide a method for calculation of *de* following LC chiral resolution.^[19]^

Equilibrating species such as conformational rotamers formed due to addition of protecting groups are known to create complex ^1^H NMR presentations. Rotamers are often distinguishable as independent molecules due to their high kinetic barrier to rotation.[^4^, ^20^] Chemical exchange experiments can distinguish between equilibrating conformers.[^20b^, ^21^] Indeed Hu et al. have demonstrated the use of 1D NOESY for distinguishing between rapidly equilibrating rotamers in the presence of non‐equilibrating diastereomers of Boc*‐*protected valine depsipeptides, due to their resonances being well resolved from one another.^[20b]^ Resonances under significant exchange demonstrate same phase negative peaks on the 1D NOE difference spectrum due to inversion transfer.[^20b^, ^22^] Additionally 2D gradient NOESY spectra may detect chemical exchange in the format of the 2D EXSY(exchange spectroscopy) spectrum which has been applied most recently to determine equilibrating *E* and *Z* isomers of conjugated enamino carbonyl systems. The presence of EXSY cross peaks indicative of equilibrating isomers with protons undergoing chemical exchange are reported as same phase signals with the diagonal of the EXSY spectrum.^[21]^

The use of NMR spectroscopy is emerging as a popular technique for diastereomer ratio determination. The plausibility of calculating diastereomeric ratios using the EXSY technique is suggested by Hussaini et al. and most recently an approach for in situ monitoring of the diastereoselectivity of peptide coupling has been reported using ^31^P or ^1^H NMR integration, accurately determining diastereomer ratios in crude reaction mixtures.^[23]^ The possibility of utilising NMR for the calculation of *de* following LC chiral resolution for complex β‐lactam mixtures is therefore plausible.[^21^, ^24^] EXSY studies are highly advantageous for precious materials as a non‐destructive technique which can be carried out with standard NMR concentration samples (10–50 mM) without loss of material.[^20b^, ^25^] Preinstalled and common pulse sequences on NMR instruments ensure that the NOESY/EXSY method combined with regular ^1^H NMR are accessible for application to confirm diastereomer separation and importantly calculate diastereomeric ratios and *de* values respectively.[^20b^, ^25^]

To our knowledge, this is the first time a panel of amino acids is employed and investigated as CDRs for chiral resolution of β‐lactam analogues. We report a convenient and accessible technique using LC chiral resolution in tandem with NMR spectroscopy for qualitative visual confirmation using 2D EXSY spectra of diastereomeric separation. Identification of diastereomeric signals using 2D EXSY further allows accurate and precise *de* calculation using the ^1^H NMR axis as a qNMR strategy to confirm the degree of success of the chiral resolution.

## Results and Discussion

### Coupling of β‐lactam 1 to amino acid CDRs

**Figure 1:**
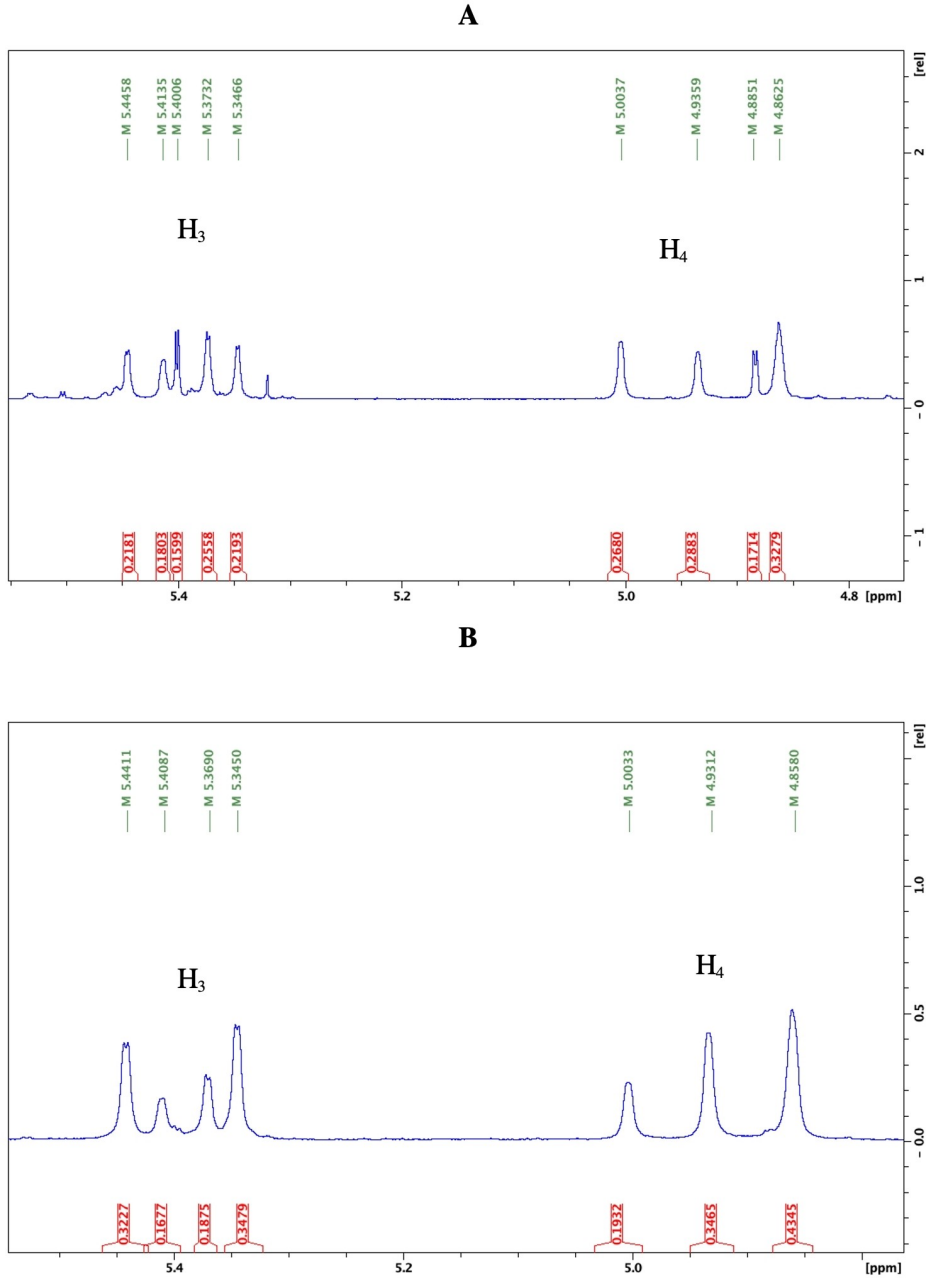
400 MHz ^1^H NMR spectra at 25 °C of the H_3_ and H_4_ region (δ 4.4‐5.5 ppm) for diastereomeric mixtures **A**: **2** and **B**: **3** in CDCl_3_. A degree of overlap is noted for H_4_ of **3** with only three of four resonances observed.

Esterification at the 3‐hydroxyl position of the β‐lactam ring of compound **1** using selected *N*‐protected amino acids was achieved using (2‐(1*H*‐benzotriazol‐1‐yl)‐1,1,3,3‐tetramethyluronium hexafluorophosphate (HBTU), N,N‐diisopropylethylamine (DIPEA) and acetonitrile (MeCN) at room temperature under inert conditions for 24 h (Scheme 1). Diastereomeric mixtures **2**–**8** (Scheme 1) were successfully synthesised in moderate to good yields (31–77 %). Coupling of *N‐tert*‐butoxycarbonyl‐l‐histidine and **1** was unsuccessful. A preliminary LC purification to isolate the diastereomer mixtures was first required. The subsequent resolution of purified diastereomer mixtures employed gravity LC over laboratory grade silica gel to obtain maximum resolution and minimal diastereomer co‐elution. Sufficient resolution of diastereomers was achieved using a slow and gradual gradient of 4 : 1 *n‐*hexane: MTBE (methyl *tert*‐butyl ether) to 2 : 1 MTBE: *n‐*hexane. Esterification and LC chiral resolution was first trialled with *N‐*Boc‐d‐proline as the CDR with the diastereomer mixture **2**, isolated in 52 % yield. Success of d *‐*proline diastereomer LC resolution was limited due to significant diastereomer co‐elution, prohibiting progression to the corresponding enantiomers of **1**. Only 15 % of **2DS1** and 5 % of **2DS2** from their original diastereomer mixture^[26]^ were isolated as partially resolved diastereomers. Using the corresponding l‐proline CDR, a much higher isolated yield of 77 % was achieved for diastereomer mixture **3**. LC chiral resolution of **3** resulted in successful isolation of purified diastereomers **3DS1** and **3DS2** in good yields of 32 % and 37 % of the original diastereomer mixture for progression towards their s3‐hydroxyl enantiomers **9** and **10** (Scheme 1) using hydrazine dihydrochloride, triethylamine and methanol for removal of the CDR. For all other amino acid CDRs trialled (**4**–**8**, Scheme 1) separation of diastereomers was not achieved on TLC or LC using identical conditions of MTBE:*n‐*hexane. Both diastereomeric mixtures **2** and **3** and their respective isolated diastereomers (**2DS1**, **2DS2** and **3DS1**, **3DS2**) demonstrated complex ^1^H NMR spectra preventing purity or diastereomeric excess (*de)* calculations from preliminary ^1^H NMR analysis (Figures 1 and 6). Adjunctive NMR experiments, including 2D NOESY/EXSY studies and variable temperature (VT) experiments were required for both resolved diastereomers, their parent diastereomer mixtures and other unresolved diastereomer mixtures **2**–**8**. Such experiments sought to understand the spectroscopic behaviour of proline diastereomers to distinguish between individual diastereomer resonances within the diastereomer mixtures of **2** and **3**. This was required in order to establish a reliable method for determining if LC resolution had been achieved when analysing ^1^H NMR spectra of resolved **2DS1**, **2DS2**, **3DS1** and **3DS2** (i. e., determine the diastereomeric purity or *de* values using ^1^H NMR).

### ^1^H NMR spectra for diastereomer derivatives 2–8

The ^1^H NMR spectra of the proline derivatized diastereomer mixtures (**2** and **3**) were analysed in CDCl_3_ (Figure 1 and Figure S1.5 in Supporting Information) and DMSO‐*d_6_* (Figures S1.14 and S1.41, S1.102). In each solvent and for both mixtures **2** and **3**, additional resonances in the H_3_ and H_4_ β‐lactam region (δ 4.5–5.5 ppm) were observed, raising the question of both diastereomer and rotamer presence in proline diastereomer mixtures which behave spectroscopically in NMR solvents as if they are chemically exchanging. Additional resonances are not observed for the H_3_ and H_4_ region for phenylalanine‐, tryptophan‐ and valine‐derivatized diastereomer mixtures (**4**–**8**, Scheme 1) in either CDCl_3_ (Figures S1.48, S1.58, S1.66, S1.81, S1.93) or DMSO‐*d_6_* (Figures S1.51, S1.60, S1.73, S1.84, S1.95). Only four anticipated diastereomeric H_3_ and H_4_ resonances are observed for **4**–**8**. Since the ^1^H NMR spectra for diastereomer mixtures **4**–**8** appear less complex the presence of rotamers is not indicated. Comparative chemical exchange experiments, including 2D EXSY and VT studies were carried out to investigate possibility of exchange for proline diastereomer mixtures **2** and **3** (Scheme 1) versus presence of diastereomeric resonances only for all other diastereomeric mixtures (**4**–**8**) and further understand the complex ^1^H NMR presentation for proline diastereomer mixtures.

### 2D EXSY spectra for 2 and 3 in CDCl_3_ confirm presence of equilibrating rotamers

The EXSY spectroscopy experiment is useful for detecting coherence transfer through chemical or confirmational exchange as a homonuclear shift correlated spectrum. EXSY spectra demonstrate off diagonal responses for spins in chemical exchange when the rate of exchange is greater or equal to the T_1_ relaxation time. Quantitative 2D EXSY can be used to deduce kinetics or rates of exchange by recording a series of EXSY spectra with different mixing times in combination with VT studies,[^27^, ^28^] reviewed in detail by Perrin and Dwyer.^[29]^ Relative intensities of cross peaks on an EXSY spectrum gives an indication of relative rate, with slow exchange generating intense cross peaks for independent conformers or rotamers.^[30]^

**Figure 2:**
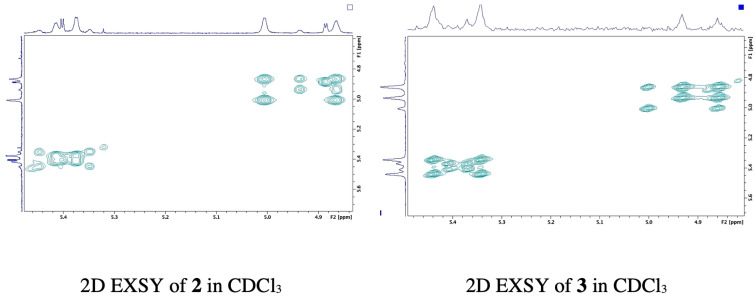
400 MHz 2D EXSY spectra for the β‐lactam H_3_ and H_4_ region (δ 4.5–5 ppm) in CDCl_3_ at 25 °C demonstrating independent sets of cross peaks for each proline‐derivatized diastereomer and their equilibrating rotamers within mixtures **2** and **3**.

2D EXSY spectra were obtained for **2** and **3** in CDCl_3_ (Figure 2) and DMSO‐*d_6_* (Figures S1.16 and S1.42 in Supporting Information) to further investigate multiplication of H_3_ and H_4_ resonances in the ^1^H NMR spectra (Figure 1, Figures S1.6, S1.14 and S1.41). While EXSY spectroscopy has potential for exchange rate calculation, this study employs the EXSY experiment for confirming presence of exchange only, accompanied by assignment of diastereomeric versus rotamer resonances on the ^1^H NMR axis. EXSY spectra were collected using 2 scans per increment, 2048×256 time domains, a mixing time of D8/500 m/s and the NOESY gpphp pulse programme (phase sensitive NOESY) from the Bruker pulse library. Equilibrating rotamers are identified and confirmed by observation of cross peaks off the diagonal of the 2D EXSY spectrum for both **2** and **3** in both solvents; CDCl_3_ (Figure 2) and DMSO‐*d_6_* (Figures S1.16 and S1.42). The complex presentation in the expanded H_3_ and H_4_ region on ^1^H NMR in CDCl_3_ (Figure 1) is accounted for by both diastereomeric and exchanging rotamer signals, detectable on ^1^H NMR at 25 °C. Furthermore both **2** and **3** in CDCl_3_ demonstrate independent equilibrating exchange signals with each of the H_3_ and H_4_ diastereomer resonances having resolved exchanging rotamer cross peaks, observed on the 2D EXSY spectra as two independent sets, one for each diastereomer (Figure 2). This serendipitous outcome suggested that CDCl_3_ would serve as an optimal solvent for qualitative confirmation of proline diastereomer purity on foot of LC chiral resolution using their 2D EXSY spectra. Successful resolution should demonstrate one set of cross peaks only per resolved diastereomer on the 2D EXSY spectrum in contrast to two sets for the parent diastereomer mixture. Rotamers are unequivocally confirmed for both **2** and **3** using a similar method to Hussaini et al. via manual phasing of the 2D EXSY cross peaks which resulted in same phase signals for cross peaks as the diagonal (Figures S1.8, S1.43 and S1.44)^[21]^ Standard zero order and first order phase correction was employed to get pure absorption signals.

### VT experiments for proline diastereomers mixtures 2 and 3 in DMSO‐d_6_

**Figure 3:**
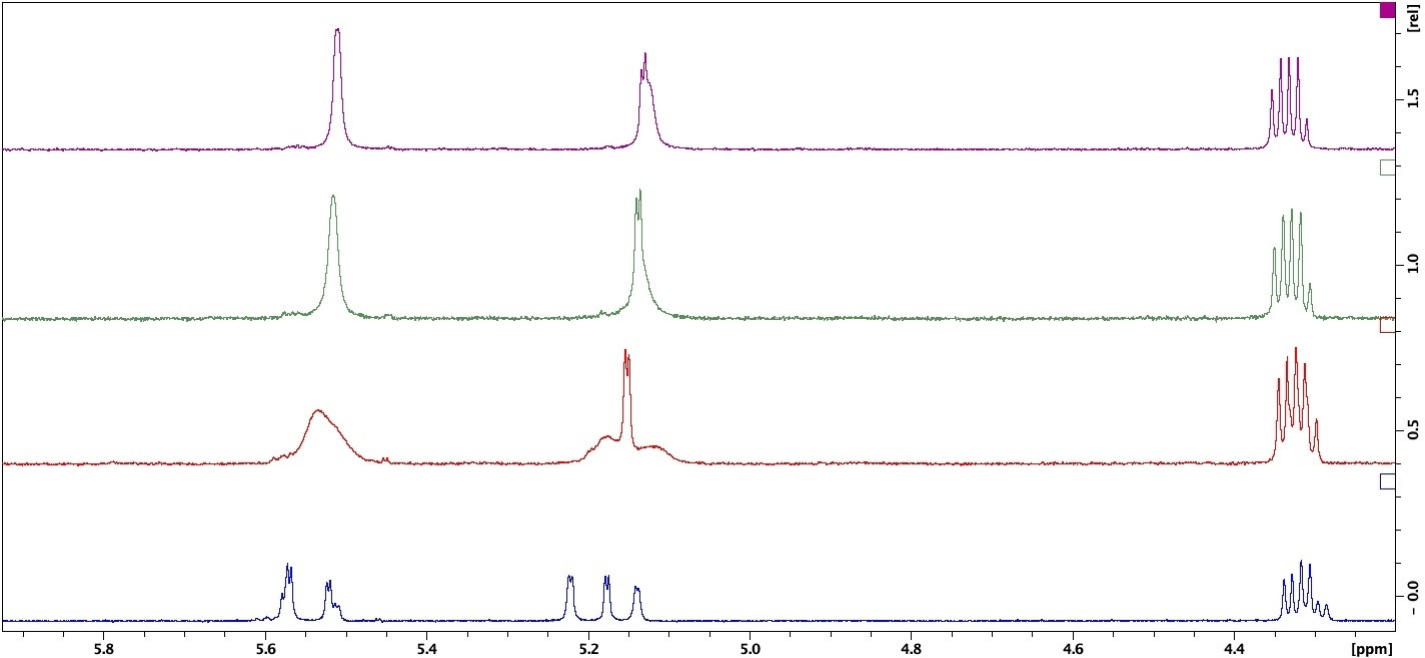
400 MHz ^1^H NMR spectra in DMSO‐*d_6_* for H_3_ and H_4_ (4.5–5.5 ppm) region of l *‐*proline CDR derivatized diastereomer mixture **3** as a function of temperature. Blue: 25 °C, red: 60 °C, green: 80 °C, purple: 90 °C.

**Figure 4:**
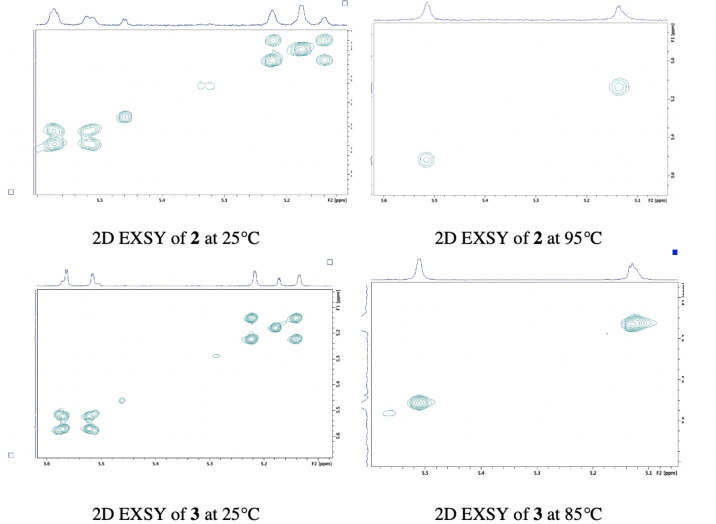
400 MHz 2D EXSY spectra at 25 °C and elevated temperatures in DMSO‐*d_6_* for expanded H_3_ and H_4_ region of **2** and **3** demonstrating one equivalent independent set of cross peaks for diastereomers in the mixture at 25 °C and a single diastereomeric signal for H_3_ and H_4_ at elevated temperatures of 95 °C and 85 °C for **2** and **3** respectively.

VT experiments were carried out for proline diastereomer mixtures **2** (Figures S1.26 and S1.29 in Supporting Information) and **3** (Figure 3 and Figures S1.44–S1.46) in DMSO‐*d_6_*. At lower temperatures of 25 °C equilibrating rotamers are detectable on ^1^H NMR, observed as cross peaks off the diagonal on 2D EXSY spectra for both **2** and **3** (Figure 4). At elevated temperatures ^1^H NMR no longer distinguishes between rotamers due to fast exchange resulting in coalescence of equilibrating resonances. With each progressive increase in temperature, notable changes occur in the H_3_ and H_4_ region for both **2** (Figures S1.26 and S1.29) and **3** (Figure 3 and Figures S1.44–S1.46). For diastereomer mixture **3**, coalescence at 60 °C to broad singlets is observed for both H_3_ and H_4_ at δ 5.2 and δ 5.5 ppm respectively with exchange broadening of 25–30 Hz observed. At higher temperatures of 80 and 90 °C in the H_3_ and H_4_ region, exchange broadening is no longer observed (green and purple, Figure 3). The H_4_ doublet is observed with a ^3^ *J* value of 1.8 Hz while exchange broadening for H_3_ is reduced to 3.6 Hz at 80 and 90 °C (green and purple, Figure 3). This progressive coalescence corroborates evidence indicative of equilibrating rotamers from 2D EXSY studies (Figure 2). Visualisation of both diastereomers within the mixture in absence of exchange signals was anticipated at elevated temperatures in the ^1^H NMR spectrum of **2** and **3** at elevated temperatures (80 and 90 °C, green and purple, Figure 3). However, resolution of diastereomer resonances was not observed. DMSO‐*d*_6_ does not distinguish between individual diastereomers within mixture **3** at either 80 and 90 °C with only one set of H_3_ and H_4_ resonances present (green and purple, Figure 3).

Additionally, only equilibrating rotamers are distinguished in DMSO‐*d*_6_ with one set of cross peaks on 2D EXSY spectra for diastereomer mixtures **2** and **3** (Figure 4). In contrast to CDCl_3_, which detects both rotamers and diastereomer pairs for **2** and **3** (Figure 1 and 2) on ^1^H NMR, DMSO‐*d_6_* cannot be used as a solvent for calculation of diastereomeric ratios since diastereomers are equivalent on both ^1^H NMR and 2D EXSY spectra. Coalescence of cross peaks on the 2D EXSY spectra at 85 °C and 95 °C for **2** and **3** towards single H_3_ and H_4_ resonances (Figure 4) further verifies that DMSO‐*d_6_* is an unsuitable solvent for calculation of *de* values due to inability to distinguish between independent diastereomer resonances. CDCl_3_ was therefore determined as the solvent choice for qualitative analysis of resolved diastereomers using 2D EXSY spectra and for *de* calculation using the ^1^H NMR axis as a qNMR technique.

### VT experiments and 2D NOESY spectra for non‐proline diastereomer mixtures 4–8

VT experiments confirm absence of rotamers for diastereomer mixtures **4**–**6**. Evidence of equilibrating rotamers on 2D NOESY spectra for CDR conjugates **4**–**6** was not observed (Figure S1.101 in Supporting Information) due to absence of H_3_ and H_4_ resonance coalescence at elevated temperatures. (Figures S1.54, S1.63, S1.79‐80, S1.88). This contrasts observations for proline diastereomer mixtures **2** and **3** with evident and progressive coalescence in the H_3_ and H_4_ region. (Figure 3 and Figures S1.26, S1.33, S1.44–S1.46) Minor evidence of coalescence is observed for valine diastereomer mixtures **7** and **8** in the H_4_ and B ring regions (δ 5.11 and 7.37–7.42 ppm respectively) (Figures S1.91–92 and S1.98–99). Coalescence for valine diastereomers was observed in VT experiments while cross peaks indicative of equilibrating rotamers were not on 2D NOESY studies. This indicates that VT experiments are more sensitive for rotamer detection than 2D chemical exchange studies. Nevertheless, exchange for valine diastereomers is faster and therefore more subtle than successfully resolved proline diastereomers (i. e., the *N‐*Boc*‐* d *‐* and l *‐* proline diastereomer mixtures **2** and **3**) which hold the unique spectroscopic property of slow conformational exchange with equilibrating rotamers which are readily observed on both ^1^H NMR (Figure 1) and 2D EXSY spectra in CDCl_3_ and DMSO‐*d_6_*. (Figures 2 and 4). Taken together, results from ^1^H NMR, 2D NOESY and VT experiments confirm that equilibrating rotamers are absent in diastereomeric mixtures **4**–**6** prepared using *N‐*Boc*‐* l‐phenylalanine, *N‐*Boc*‐* l‐tryptophan and fmoc‐phenylalanine. It is possible that the unique property of slow exchange for proline diastereomers **2** and **3** may influence separation of proline diastereomers during LC chiral resolution compared to other non‐proline diastereomer mixtures which do not form rotamers in CDCl_3_ or DMSO‐*d_6_*.

### Computational data for diastereomers of β‐lactam 1

**Figure 5:**
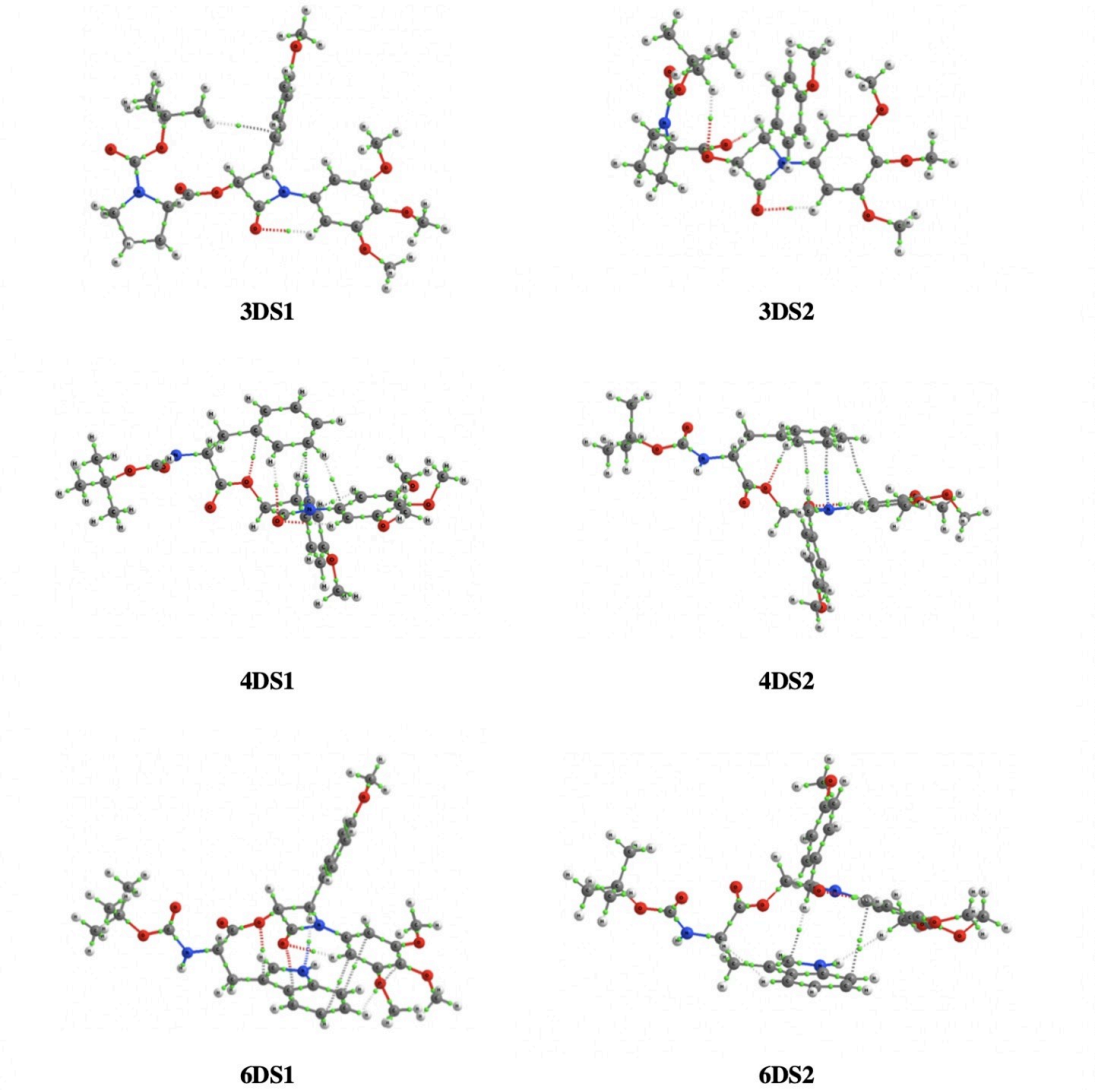
Computational and theoretical model of proline, phenylalanine and tryptophan diastereomers of β‐lactam **1**. Non‐covalent intra‐molecular π‐π interactions indicated by grey dotted lines are present for aromatic moieties of phenylalanine and tryptophan diastereomers only. Hydrogen bonds illustrated as red dotted lines. Representative examples of major rotamers only are illustrated. Atom colours: red=oxygen, grey=carbon, blue=nitrogen.

A computational study was carried out for selected *N‐*Boc protected amino acid diastereomers (**3**, **4**, and **6**, Figure 5) to examine the process of rotamer exchange that is unique in the case of the proline diastereomers and is not observed for other amino acid diastereomers studied. Intramolecular non‐covalent interactions (NCIs) are not observed between the l *‐*proline CDR and β‐lactam scaffold of **1**, which potentially explains the rotamer presence. The absence of non‐covalent intramolecular π‐π stacking interactions between CDR and β‐lactam scaffold of **1**, due to proline's non‐aromatic structure, may enable free rotation of the proline ring for **2** and **3** and therefore enables exchange and rotamer formation. In contrast to proline, the aromatic amino moieties of phenylalanine (**4**) and tryptophan (**6**) may form intramolecular non‐covalent π‐π interactions with the β‐lactam scaffold of **1** (Figure 5). We propose that these intramolecular π‐π interactions between the CDR and β‐lactam scaffold of **1** for diastereomers of **4** and **6** stabilise phenylalanine (**4**) and indole (**6**) aromatic rings, limiting their free rotational capacity and preventing exchange. Steric effects are traditionally considered as a major factor for consideration in computational models since electronic energies of non‐bonded atoms increases exponentially with decreasing distance. However, in recent years NCIs are more widely regarded as factors influencing selectivity of organocatalytic reactions.^[31]^ NCIs have previously been reported as major determinants of the stereoselectivity for histamine complexes.^[32]^

Major conformers for these diastereomers (**4DS1**, **4DS2**, **6DS1** and **6DS2**) arise on account of non‐covalent π‐π interactions, stabilising one predominant conformation (Figure 5). Notwithstanding the complexities associated with accurate computational predictions of structures where NCIs in addition to steric effects are possible, our β‐lactam diastereomer models (Figure 5) which illustrate these NCI π‐π interactions gives rise to a plausible explanation for major conformational exchange observed in the H_3_ and H_4_ region only for proline diastereomers (Figure 1 and 2) and absence of exchange for non‐proline CDRs on both ^1^H NMR (Figure S1.100 in Supporting Information) and 2D NOESY studies (Figure S1.101). Due to the non‐aromatic structure of valine, π‐π interactions were not possible between the amino acid CDR and the β‐lactam ring in diastereomeric mixtures **7** and **8**, explaining why valine diastereomers were the only non‐proline diastereomer mixtures with minor evidence of exchange on 2D EXSY and VT experiments (Figures S1.90–92, S1.98–99).

### ^1^H NMR and 2D EXSY spectra for isolated N‐Boc‐l‐ and d‐ proline diastereomers

**Figure 6:**
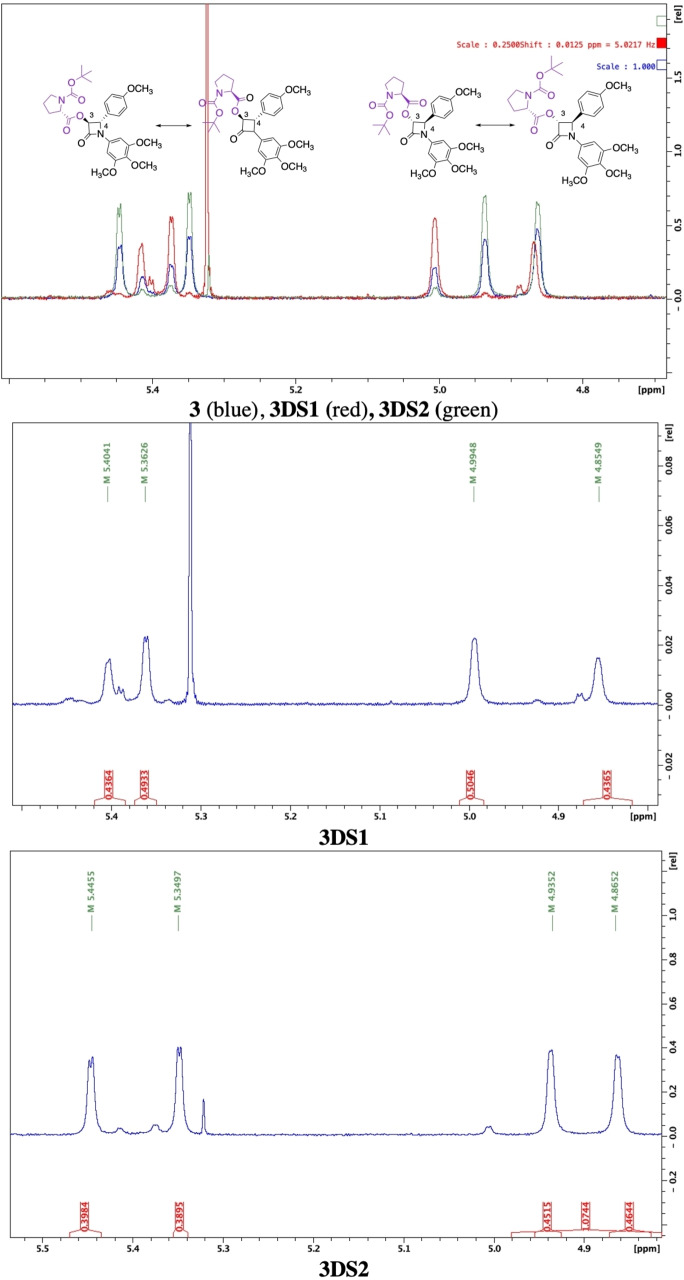
Overlay of 400 MHz ^1^H NMR spectra (Top) of resolved **3DS1** (middle) and **3DS2** (bottom) at 25 °C. Expanded H_3_ and H_4_ region (δ 4.8–5.5 ppm) in CDCl_3_ shown.

Both **2** and **3** were successfully resolved using LC with superior resolution and minimal diastereomer co‐elution of **3DS1** and **3DS2** using *N‐*Boc*‐* l *‐*proline compared to the corresponding d *‐*proline CDR for resolution of **2DS1** and **2DS2**. Significant co‐elution of **2DS1** and **2DS1** reduced isolated yields to 25 % for **2DS1** and 5 % for **2DS2**. On the other hand, 32 % and 37 % of the original diastereomer mixture were resolved for **3DS1** and **3DS2** respectively. *N‐*Boc*‐* l *‐*proline was therefore determined as the optimal CDR in this study for resolution of 3‐hydroxyl β‐lactam **1**. Direct comparison of the ^1^H NMR axis of **3DS1** and **3DS2** by overlaying the ^1^H NMR spectra for isolated diastereomer mixtures **3DS1** (red, Figure 6) and **3DS2** (green, Figure 6) with parent diastereomer mixture **3** (blue, Figure 6) demonstrates unique resonances for H_3_ and H_4_ for **3DS1** and **3DS2** and infers successful resolution. Two major resonances are observed for both H_3_ and H_4_, representing exchanging rotamer pairs for each diastereomer. Additionally, the ratio of exchanging rotamers was determined for **3DS1** as 1 : 1.14 while rotamers for **3DS2** were determined in equal ratios of 1 : 1. Identification and quantification of the H_3_ and H_4_ rotameric and diastereomeric resonances allowed further application of the qNMR technique for *de* calculations (Table 1).

**Table 1:** Integration of H_3_ and H_4_ resonances on ^1^H NMR axis for qNMR determination of d.r^1^ and *de* values for resolved proline diastereomers **2DS1**, **2DS2**, **3DS1** and **3DS2**.

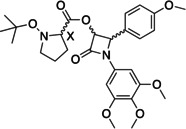							
	X	d.r (H_3_)^[a]^ (D1:D2)	de % (H_3_)^[b]^	d.r (H_4_)^[a]^ (D1:D2)	de % (H_4_)^[b]^	de % (average of H_3_ and H_4_ de)	ee %^[c]^
**2DS1**	*R*	83 : 17	66	80 : 20	60	63	Nd
**2DS2**	*R*	73 : 27	46	72 : 28	44	45	Nd
**3DS1**	*S*	94 : 6	88	96 : 4	92	90	94
**3DS2**	*S*	86 : 14	72	88 : 12	76	74	71

**[a]** d.r (diastereomeric ratio) is the ratio of the percentage of one diastereomer in a mixture (D1) to that of the other (D2). D1 and D2 are calculated as the integrals of rotamers for the major and minor diastereomers, respectively, normalised to 100 %.^[14]^; **[b]** *de=*D1–D2; **[c]** *ee* (enantiomer excess) calculated using chiral HPLC analysis of enantiomers **9** and **10**. Nd=not determined.

**Figure 7:**
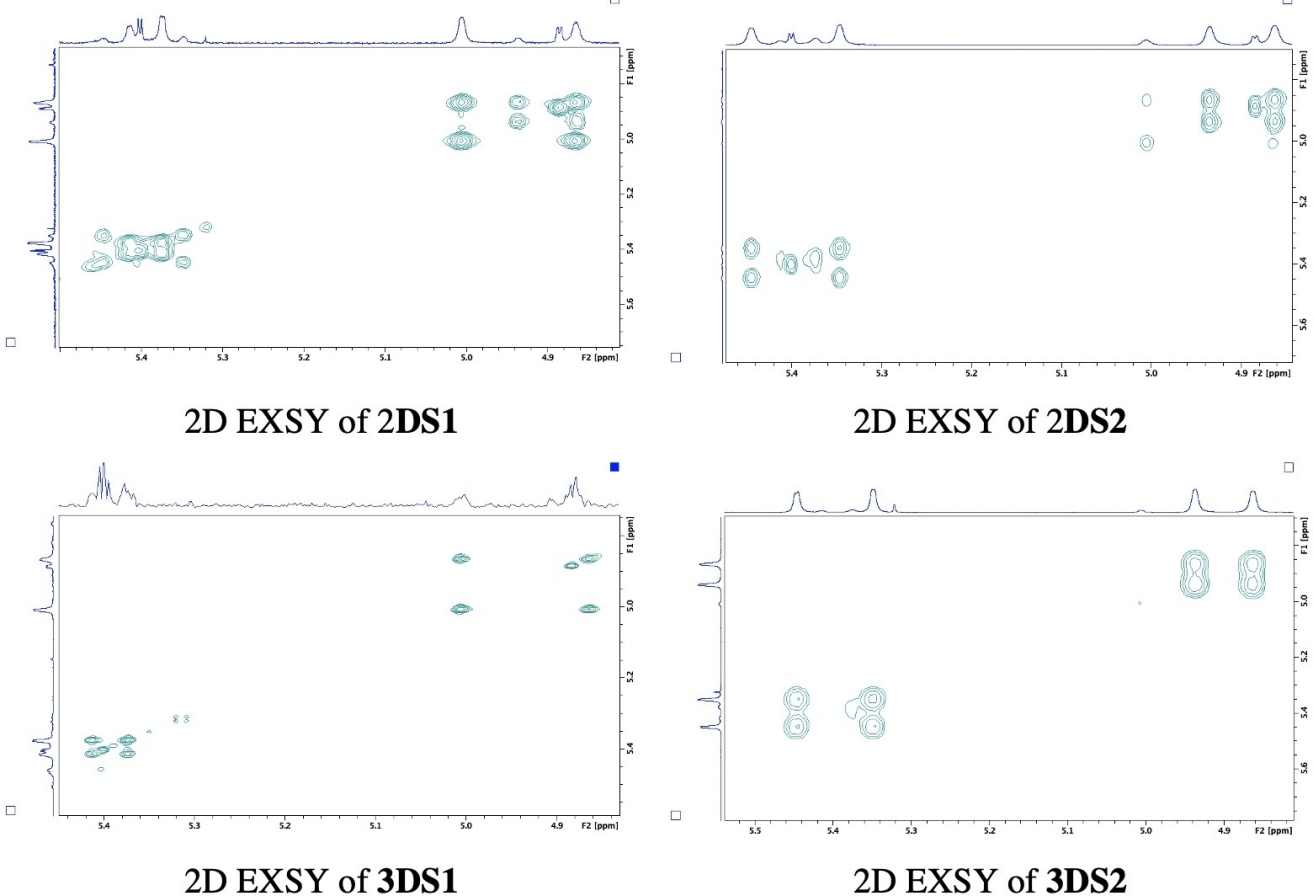
400 MHz 2D EXSY spectra at 25 °C for H_3_ and H_4_ region of resolved diastereomers **2DS1**, **2DS2**, **3DS1** and **3DS2** in CDCl_3_.

Equilibrating rotamers for each diastereomer within the mixtures enabled use of 2D EXSY spectra as a qualitative tool to determine diastereomeric purity. Chemical exchange can be readily detected as cross peaks off the diagonal, following LC chiral resolution. Figure 7 compares the 2D EXSY spectra for isolated **2DS1** and **2DS2** versus **3DS1** and **3DS2** in CDCl_3_. Co‐elution during LC chiral resolution and poor *de* as a qualitative determination is evident on the 2D EXSY spectra for **2DS1** and **2DS2**. One major set of cross peaks is accompanied by minor presence of a second set, indicative of the presence of a second diastereomer and its exchanging rotamers and therefore incomplete resolution. In contrast, only one set of cross peaks are present on 2D EXSY spectra for resolved l *‐*proline diastereomers **3DS1** and **3DS2** (Figure 7). This contrasts the 2D EXSY spectrum obtained for parent diastereomer mixture **3** (Figure 2) with two independent sets of cross peaks representative of each diastereomer and their own exchanging rotamers. Comparing 2D EXSY for isolated diastereomers therefore serves as qualitative and visual tool for confirmation of diastereomer purity following LC chiral resolution of β‐lactam proline diastereomers of **1**. 2D EXSY spectra also support the use of *N‐*Boc‐l‐proline as the superior amino acid CDR for β‐lactam **1** which results in the optimal diastereomer resolution.

### qNMR for calculation of diastereomeric excess for β‐lactam proline diastereomers

By virtue of seeking a robust method for structural characterisation of the H_3_ and H_4_ β‐lactam resonances of resolved diastereomer mixtures, in order to confirm successful diastereomer resolution, we now report a method for *de* quantitative calculation using qNMR of the H_3_ and H_4_ region. This qNMR strategy accurately and precisely reports diastereomeric purity in the format of a *de* value in line with IUPAC guidelines for *de* calculation.^[14]^ Quantitative (q) ^1^H experiments utilised a 90° excitation pulse and a 60 second recycle delay between scans to allow the spins to return to equilibrium ensuring 5 times the T_1_ (spin lattice relaxation) was exceeded. Distinguishing between diastereomeric and rotameric resonances for H_3_ and H_4_ protons of the β‐lactam ring has enabled integration of H_3_ and H_4_ diastereomeric resonances and calculation of *de* values (Table 1).

*De* values were obtained independently for H_3_ and H_4_ of resolved diastereomers **2DS1**, **2DS2**, **3DS1** and **3DS2** and reported as the average of both H_3_ and H_4_ integrations listed in Table 1 (illustrated in Supporting Information, Figure S1.103). The *de* value of 90 % for l *‐*proline **3DS1** is superior to the corresponding d *‐*proline conjugate **2DS1** (63 %). Similarly **3DS2** was isolated in larger *de* of 74 % when compared to 45 % *de* for **2DS2** further validating that *N‐*Boc*‐* l *‐*proline is the more optimal CDR evaluated in this study for the chiral resolution of β‐lactam **1**. ^1^H NMR spectroscopy as a qNMR tool for determination of *de* values of resolved diastereomers **3DS1** and **3DS2** was validated by comparison to *ee* values of 94 % and 71 % obtained for 3‐hydroxyl enantiomers **9** and **10** respectively using chiral HPLC (Scheme 1). The standard error for *de* and *ee* values of **3DS1** and **3DS2** (4 % and 3 % respectively) underpins the utility of this qNMR method for quantitative determination of the degree of diastereomer resolution using LC.

## Conclusions and Outlook

In summary we prepared a series of diastereomer mixtures of β‐lactam **1** with aim of selecting the optimal CDR for the resolution of **1** in the form of amino acid derivatized diastereomer mixtures. d‐ and l *‐*proline derivatised mixtures **2** and **3** were the only diastereomers that were both successfully resolved and were observed with strong evidence of rotamer exchange in all NMR experimental conditions using both CDCl_3_ and DMSO‐*d_6_. N‐*Boc‐l‐proline was determined as the optimal CDR in this study. Theoretical computational models of relevant amino acid diastereomers demonstrated that intramolecular π‐interactions between the aromatic CDRs (phenylalanine and tryptophan) and β‐lactam scaffold of **1** may prevent the formation of rotamers. 2D EXSY spectra served as a visual and qualitative tool to determine diastereomeric purity in resolved diastereomers. Successful characterisation of H_3_ and H_4_ rotamer versus diastereomer resonances in CDCl_3_ has enabled use of quantitative integration as a robust qNMR method for determination of *de* values. Calculated *de* values are in close agreement with chiral HPLC *ee* values determined for the final 3‐hydroxyl enantiomeric products. Future work will involve application of the optimised LC chiral resolution in tandem with qualitative 2D EXSY and qNMR spectroscopy for *de* calculations to a panel of 3‐hydroxyl β‐lactam analogues of biological interest. In principle, this method of LC chiral resolution in conjunction with qNMR is widely applicable and of synthetic relevance for racemates bearing an hydroxyl moiety for resolution and confirmation of diastereomeric purity.

## Experimental Section

All reagents were commercially available and were used without any further purification unless otherwise indicated. Infrared (IR) spectra were recorded on a Perkin Elmer FTIR Paragon 1000 spectrometer.

Nuclear magnetic resonance (NMR) spectra were recorded at 25 °C on a Bruker Avance III 400 or Avance II 600 (400.13 MHz/ 600.13 MHz, ^1^H; 100.61 MHz, ^13^C/150.61 MHz, ^13^C)) in either CDCl_3_ or DMSO‐*d*_6_. For CDCl_3_, the internal standard is tetramethylsilane (TMS) with ^1^H NMR spectra assigned relative to this TMS peak at δ 0.00 ppm. ^13^C NMR spectra were assigned relative to the middle peak of CDCl_3_ triplet at δ 77.00 ppm. For ^1^H NMR assignments, chemical shifts are reported: shift value (multiplicity, integral value, coupling constant(s)). High resolution mass spectrometry (HRMS) was performed by Dr. Gary Hessman in the School of Chemistry. ESI mass spectra were acquired using a Bruker micrOTOF‐Q III spectrometer interfaced to a Dionex UltiMate 3000 LC in positive and negative modes as required. Masses were recorded over the range 100–1400 *m/z*. APCI experiments were carried out on a Bruker microTOF‐Q III spectrometer interfaced to a Dionex UltiMate 3000 LC or direct insertion probe. The instrument was operated in positive or negative mode as required. Masses were recorded over a range of 100–1600 *m/z*. Thin Layer Chromatography (TLC) was carried out on silica gel on Sigma Aldrich/Merck's silica gel on TLC aluminium foils with fluorescent indicator F‐254 nm. Retention factor (R_f_) values are quoted for each compound. Flash column chromatography was carried out on silica gel technical grade, pore size 60 Å, 40–63 μM particle size, 230–400 mesh. HPLC instrumentation consisted of a Waters 1525 Binary HPLC Pump in‐line vacuum degasser, a 717plus auto sampler and a Waters 2487 dual wavelength detector. Achiral HPLC was carried out using a using a reverse phase C18 Agilent Eclipse Plus column. 4.6×100 mm. The mobile phase was 70 % HPLC grade Acetonitrile and 30 % Water at an injection volume of 10 μL and flow rate of 1 mL/min. Chiral HPLC was carried using the chiral stationary phase column, Chrompak‐IH‐3 (150×4.6 mm) supplied by Chiral Technologies Europe with a Chiral‐ IH‐3 guard column. The following conditions were used: An injection volume 5 μL, flow rate of 1 mg/mL and run time of 10 minutes, using the mobile phase of HPLC grade *n*‐hexane:propan‐2‐ol, 1 : 1. Enantiomeric excess (%) was calculated using the following formula: [(% Major Peak ‐% Minor Peak)×100].

β‐Lactam **1** [3‐hydroxy‐4‐(4‐methoxyphenyl)‐1‐(3,4,5‐trimethoxyphenyl)azetidin‐2‐one]^[33]^ was obtained using general method previously described by our group,^[3b]^ and used as starting material for all diastereomer derivatizations. ^**1**^ **H NMR** (**400 MHz**, **CDCl_3_**): δ 7.26 Hz (d, 2H, *J=*8.4 Hz), 6.90 (d, 2H, *J=*8.4 Hz), 6.5 (s, 2H), 5.29 (s,1H, OH), 4.79 (d, 1H, *J=*1.9 Hz), 4.74 (d, 1H, *J=*1.89 Hz), 3.80 (s, 3H), 3.75 (s, 3H), 3.68 (s, 6H); ^**13**^ **C NMR** (**100 MHz**, **CDCl_3_**): δ 167.9, 160.0, 153.3, 135.2, 134.2, 127.8, 127.5, 114.5, 95.2, 83.6, 66.0, 60.9, 55.9, 55.3.

### General Method I: Diastereomer derivatisation of β‐lactam 1 using Fmoc and *N‐(tert‐*butoxycarbonyl) protected amino acids amino acids as CDRs

The relevant racemate (1 eq, 1 mmol) was added to anhydrous MeCN (20 mL), under inert conditions at room temperature. HBTU (2.2 eq, 2.2 mmol, 0.83 g) and the respective *N*‐Boc or Fmoc amino acid (2 eq, 2 mmol) were added to the reaction vessel dissolved in 5–10 mL of ACN. DIPEA (61 eq, 61 mmol, 10.76 mL) was added dropwise and the mixture was stirred for 24 h under inert conditions. The solvent was removed *in vacuo* to afford a crude yellow oil. The residue was diluted with deionised water (20 mL) and extracted with dichloromethane (3×50 mL). The organic layer was washed with KHSO_4_ (30 mL), saturated NaHCO_3_ solution (30 mL) and brine (30 mL). The organic layer was retained and dried with anhydrous Na_2_SO_4_ and solvent subsequently removed *in vacuo*. Crude samples were dissolved in minimal quantity of DCM and purified using flash column chromatography over silica (set at *n*‐hexane/ethyl acetate 7 : 3, eluent: *n*‐hexane/ethyl acetate 3 : 2). The purified diastereomers were analysed using TLC under various ratios of TBME/*n‐*hexane. In most cases separation of diastereomers was not observed and thus no further purification was carried out. Diastereomer derivatives are therefore characterised and reported as the diastereomer mixture. In the case of *N‐*(*tert*‐butoxycarbonyl)‐l and d‐proline derivatives, purification of individual diastereomers was achieved by gravity LC with silica gel using gradient elution (*n‐*hexane*/*MTBE 1 : 1 to TMBE/*n‐*hexane 4 : 1).

**1‐**(***tert*** **‐Butyl**) **2‐**(**2‐**(**4‐methoxyphenyl**)**‐4‐oxo‐1‐**(**3**,**4**,**5‐trimethoxyphenyl**)**azetidin‐3‐yl**) (***S***)**‐pyrrolidine‐1**,**2‐dicarboxylate** (**3**) A mixture of diastereomers **3** was synthesised using General Method 1 from **1** and *N*‐(*tert*‐butoxycarbonyl)‐l‐proline **Yield**: 1.08 g (1.7 mmol) 70–77 % . **Appearance**: white powder **m.p**.: 65–69 °C ^**1**^ **H NMR** (**400 MHz**, **CDCl_3_** )**: δ** 7.32 (doublet, 2H, *J*=8.9 Hz), 6.95 (d, 1.2H, *J*=8.9 Hz), 6.91 (d, 0.8H, *J*=8.9 Hz), 6.57 (s, 0.7H), 6.56 (s, 1.3H), 5.45 (d, 0.3H, *J*=1.9 Hz), 5.41 (apparent singlet, 0.16H), 5.38 (d, 0.3H, *J*=1.9 Hz), 5.34 (d, 0.2H, *J*=1.9 Hz), 5.00 (apparent singlet, 0.25H), 4.94 (apparent singlet, 0.35H), 4.86 (apparent singlet, 0.5H), 4.40 (m, 1H), 3.87 (s, 1.4H), 3.83 (s, 0.1H), 3.82 (s, 0.6H), 3.79 (s, 1.2H), 3.79 (s, 1.7H), 3.72 (s, 6H, OCH_3_), 3.6–3.4 (m, 2H), 2.36–1.9 (m, 4H), 1.53 (s, 3H), 1.47(s, 2.5H), 1.45 (s, 4.6H) ^**13**^ **C NMR** (**100 MHz**, **CDCl_3_**): 172.4, 172.2, 172.0, 171.9, 161.5, 161.2, 160.3, 160.0, 154.5, 153.7, 153.6, 153.5, 135.0, 135.0, 134.9, 133.1, 133.0, 127.8, 127.0, 126.8, 116.2, 114.8, 114.6, 114.4, 112.0, 95.5, 95.3, 82.9, 82.8, 80.4, 80.3, 80.2, 63.7, 63.5, 63.4, 61.0, 59.1, 58.8, 58.6, 56.1, 55.4, 46.7, 46.4, 31.1, 30.1, 29.8, 24.6, 24.5, 23.7^**1**^ **H NMR** (**400 MHz**, **DMSO‐*d***_**6**_ ): δ 7.45 (d, 1.2H, *J*=7.8 Hz), 7.40 (d, 0.8H, *J*=7.8 Hz), 6.97 (d, 2H, *J*=7.8 Hz), 6.54 (s, 0.1H), 6.53 (s, 1H), 5.58 (d, 0.1H, *J=*1.7 Hz), 5.56 (d, 0.4H, *J*=1.7 Hz), 5.52 (d, 0.3 H, *J*=1.7 Hz), 5.50 (d, 0.1H, *J=*1.7 Hz), 5.21 (d, 0.3H, *J=*1.7 Hz),5.17 (d, 0.2H, *J=*1.7 Hz), 5.13 (d, 0.4H, *J=*1.7 Hz), 4.31 (m, 1H), 3.75 (s, 3H), 3.63 (s, 6H), 3.58 (s, 3H), 3.45 (m, 2H), 2.28 (m, 1H), 2.00 (m, 1H), 1.87 (m, 2H), 1.42 (s, 1H), 1.38 (s, 3H), 1.35 (s, 2H), 1.34 (s, 4H); ^**13**^ **C NMR** (**100 MHz**, **DMSO‐*d***_**6**_ ): δ 172.4, 172.3, 172.1, 172.0, 161.7, 161.5, 160.1, 154.1, 153.6, 153.2, 134.8, 132.8, 132.7, 129.1, 129.0, 128.9, 128.9, 127.7, 127.6, 127.5, 114.8, 114.7, 96.1, 96.0, 82.3, 82.1, 79.7, 79.6, 62.7, 62.6, 62.5, 60.6, 58.9, 58.7, 56.3, 55.6, 46.9, 46.7, 30.9, 30.8, 29.9, 29.8, 28.5, 28.3, 24.5, 23.7; **IR** (**ATR**): $\tilde{v}$ 1756 cm^−1^ (β‐lactam C=O), 1694 cm^−1^ (*N*‐Boc‐ l‐proline C=O); **HRMS (APCI)**: *m/z* calcd for C_29_H_36_N_2_O_9_+H^+^: 557.2482 **[*M*+H^+^]**; found 557.2493; HRMS (**ESI)** *m/z* calcd for C_29_H_36_N_2_O_9_+Na^+^: 579.2323 **[*M*+Na^+^]**; found 579.2313 **Purity RP‐HPLC**: 98 %

**1‐**(***tert*** **‐Butyl**) **2‐**((**2*S***,**3*S***)**‐2‐**(**4‐methoxyphenyl**)**‐4‐oxo‐1‐**(**3**,**4**,**5‐trimethoxyphenyl**)**azetidin‐3‐yl**) (***S***)**‐pyrrolidine‐1**,**2‐dicarboxylate** (**3DS1**) **3DS1** was purified from **3** using the column chromatography conditions described in General Method 1. **Yield**: 300 mg (0.54mmols); 30 %; **R_f_**: 0.38 (*n‐*hexane/TBME 1 : 2) *(Plate developed twice)*; **m.p**.: 66–72 °C; white amorphous fluffy powder; ^**1**^ **H NMR** (**400 MHz**, **CDCl_3_**): rotamers present: δ 7.23 (d, 2H, *J=*8 Hz), 6.94 (d, 1H, *J=*8.3 Hz), 6.90 (d, 1H, *J*=8.3 Hz), 6.55 (s, 1H), 6.54 (s, 1H), 5.4 (apparent s, 0.4H), 5.36 (d, 0.5H, *J=*1.3 Hz), 5.0 (apparent s, 0.5), 4.85 (apparent s, 0.5H), 4.38 (m, 1H), 3.83 (s, 1.2H), 3.81 (s, 1.7H), 3.76 (s, 3H), 3.71 (s, 6H), 3.6–3.38 (m, 2H), 2.3–1.9 (m, 4H), 1.51 (s, 6H), 1.46 (s, 3H); ^**13**^ **C NMR** (**100 MHz**, **CDCl_3_**): δ 172.4, 172.2, 161.4, 161.2, 160.3, 159.9, 154.5, 153. 5, 153.5, 153.4, 134.9, 134.8, 132.9, 130.9, 128.8, 127.8, 127.7, 127.6, 127.0, 127.0, 114.6, 114.4, 95.4, 95.1, 82.8, 82.7, 80.2, 80.2, 63.6, 62.0, 58.6, 58.5, 56.0, 55.4, 55.3, 46,7, 46.4, 31.0, 29.9, 28.4, 28,4, 24.5, 23.7, 23.6; **IR** (**ATR**): $\tilde{v}$ 1756 cm^−1^ (β‐lactam C=O), 1697 cm^−1^ (*N‐*Boc‐l‐proline C=O); **HRMS (APCI)**: *m/z* calcd for C_29_H_36_N_2_O_9_+H^+^: 557.2482 **[*M*+H^+^]**; found 557.2493; HRMS (**ESI)**: *m/z* calcd for C_29_H_36_N_2_O_9_+Na^+^: 579.2323 **[*M*+Na^+^]**, ; found 579.2313 **Purity RP‐HPLC**: 99 %

**1‐**(***tert*** **‐Butyl**) **2‐**((**2*R***,**3*R***)**‐2‐**(**4‐methoxyphenyl**)**‐4‐oxo‐1‐**(**3**,**4**,**5‐trimethoxyphenyl**)**azetidin‐3‐yl**) (***S***)**‐pyrrolidine‐1**,**2‐dicarboxylate** (**3DS2**) **3DS2** was purified from the above mixture of **2** using the column chromatography conditions described in General Method I **Yield**: 350 mg (0.63mmols); 37 %; white powder; **R_f_**: 0.33 (*n‐*hexane/TBME 1 : 2) *(Plate developed twice)*; **m.p**.: 62–65 °C; ^**1**^ **H NMR** (**400 MHz**, **CDCl_3_**), **rotamers present**: δ 7.31 (apparent t, 2H, *J*=8 Hz), 6.94 (apparent t, 2H, *J*=8.5 Hz), 6.5 (s, 2H), 5.45 (d, 0.5H, *J*=1.5 Hz), 5.34 (d, 0.4 H, *J*=1.3 Hz), 4.93 (apparent singlet, 0.5H), 4.86 (apparent singlet, 0.5H), 4.39 (m, 1H), 3.83 (s, 1.5H), 3.82 (s, 1.5H), 3.79 (s,3H), 3.72 (s, 3H), 3.71 (s, 3H), 3.61–3.42 (m, 2H), 2.4–2.2 (m, 2H), 2.1–1.9 (m, 2H), 1.44 (s, 9H)^**13**^ **C NMR** (**100 MHz; CDCl_3_**): δ 172.1, 161.5, 161.2, 160.2, 160.1, 154.5, 154. 5, 153.6, 153.5, 153.4, 135.0, δ 134.9, 133.0, 132.9, 128.8, 127.7, 127.0, 126.8, 126.5, 114.7, 114.5, 95.3, 95.2, 82.8, 82.6, 80.5, 80.1, 63.4, 63.3, 63.9, 59.0, 58.8, 56.0, 56.0, 55.3, 55.3, 49.4, 46.6, 46.3, 31.6, 31.0, 29.8, 28.2, 28.4, 28.3, 26.9, 24.7, 23.7. **IR** (**ATR**): $\tilde{v}$ 1756 cm^−1^ (β‐lactam C=O), 1694 cm^−1^ (*N‐*Boc‐l‐proline C=O);**HRMS (APCI)**: *m/z* calcd for C_29_H_36_N_2_O_9_+H^+^: 557.2488 **[*M*+H^+^]**; found 557.2493; HRMS (**ESI)**: *m/z* calcd for C_29_H_36_N_2_O_9_+Na^+^: 579.2324 **[*M*+Na^+^]**; found 579.2313 **Purity RP‐HPLC**: 95 %

### General Method II: Hydrolysis of *N*‐(*tert*‐butoxycarbonyl)‐l‐Proline from β ‐ lactam diastereomers affording optically pure enantiomers

Hydrazine dihydrochloride (5 eq) was added to a stirring solution of respective diastereomer in anhydrous methanol (30 mL) at 0 °C (on ice) under a nitrogenous atmosphere. Anhydrous TEA (9 eq) was added dropwise to the vessel. The solution was allowed reach room temperature before heating to reflux for 6 h. The temperature was maintained at 80 °C. The solvent was removed under reduced pressure and residue treated with a saturated solution of potassium hydrogen sulfate (KHSO_4_). Extraction was performed using ethyl acetate (2×30 mL). The organic layers were washed with sodium bicarbonate (3×20 mL) to ensure removal of free proline. The organic phase was dried with anhydrous Na_2_SO_4,_ filtered and solvent removed under reduced pressure. The crude was then purified using flash chromatography over silica gel (eluent: *n*‐hexane/ethyl acetate 1 : 2) to afford the desired product.

### *dextrorotatory ‐* 3‐hydroxy‐4‐(4‐methoxyphenyl)‐1‐(3,4,5‐trimethoxyphenyl)azetidin‐2‐one (9)

**9** was synthesized from **3DS1** using General Method II**; Yield**: 108 mg (0.3 mmol), 57 %, **R_f_**: 0.36 (1 : 1 ; n‐hexane:ethyl acetate); **m.p**.: 120–122 °C, **[α]^20^**_**D**_ : +25.14, ^**1**^ **H NMR (400 MHz, CDCl_3_)**: δ 7.26 Hz (d, 2H, *J=*8.4 Hz, H_2’’&6’’_), 6.90 (d, 2H, *J=*8.4 Hz, H_3’’&5’’_), 6.5 (s, 2H, H_1’&3’_), 5.29 (s,1H, OH), 4.79 (d, 1H, *J=*1.9 Hz, H_4_), 4.74 (d, 1H, *J=*1.89 Hz, H_3_), 3.80 (s, 3H, H_10’_), 3.75 (s, 3H, H_8’_), 3.68 (s, 6H, H_7’&9’_) ^**13**^ **C NMR (100 MHz, CDCl_3_)**: δ167.9 (C_2_), 160.0 (C_4’’_), 153.3 (C_4’&6’_), 135.2 (C_5’_), 134.2 (C_2’_), 127.8 (C_1’’_), 127.5 (C_2’’&6’’_), 114.5 (C_3’’&5’’_), 95.2 (C_1’&3’_), 83.6 (C_3_), 66.0 (C_4_), 60.93 (C_8’_), 55.93 (C_7’&9’_), 55.32 (C_10’_) **IR (ATR)**: $\tilde{v}$ 3284 cm^−1^ (OH), 1726 cm^−1^ (β‐lactam C=O); HRMS (**APCI)** *m/z* calcd for **[M+H^+^]**, 360.1445; found 360.1441**[M+ Na^+^]**, 382.1266; found 382.1261; **Purity (RP‐HPLC)**: 97 %; ***ee (*** **Chiral HPLC)**: 94 %.

### *levorotatory‐* 3‐hydroxy‐4‐(4‐methoxyphenyl)‐1‐(3,4,5‐trimethoxyphenyl)azetidin‐2‐one (10)

**10** was synthesized from **3DS2** using General Method II**; Yield**: 150 mg (0.42 mmol) 66 %, **R_f_**: 0.36 (1 : 1 ; n‐hexane:ethyl acetate); **m.p**.: 120–125 °C, **[α]^20^**_**D**_ **: −2**5.38, ^**1**^ **H NMR (400 MHz, CDCl_3_)**: δ 7.26 Hz (d, 2H, *J=*8.4 Hz, H_2’’&6’’_), 6.90 (d, 2H, *J=*8.4 Hz, H_3’’&5’’_), 6.5 (s, 2H, H_1’&3’_), 5.29 (s,1H, OH), 4.79 (d, 1H, *J=*1.9 Hz, H_4_), 4.74 (d, 1H, *J=*1.89 Hz, H_3_), 3.80 (s, 3H, H_10’_), 3.75 (s, 3H, H_8’_), 3.68 (s, 6H, H_7’&9’_) ^**13**^ **C NMR (100 MHz, CDCl_3_)**: δ167.9 (C_2_), 160.0 (C_4’’_), 153.3 (C_4’&6’_), 135.2 (C_5’_), 134.2 (C_2’_), 127.8 (C_1’’_), 127.5 (C_2’’&6’’_), 114.5 (C_3’’&5’’_), 95.2 (C_1’&3’_), 83.6 (C_3_), 66.0 (C_4_), 60.93 (C_8’_), 55.93 (C_7’&9’_), 55.32 (C_10’_); **IR (ATR)**: $\tilde{v}$ 3295 cm^−1^ (OH), 1723 cm^−1^ (β‐lactam C=O); **HRMS (APCI)** *m/z* calcd for C_19_H_20_NO_6_ **+H^+^** 360.144698; found 360.144164, and **[M+ Na^+^]**, 382.126695; found 382.126108; **Purity (RP‐HPLC)**: 91 %; ***ee (*** **Chiral HPLC)**: 71 %

Additional information is available as Supporting Information, detailing full NMR experiments for **2**–**8** inclusive of expansions of 2D NOESY, EXSY and VT experiments to support experiments discussed. Characterisation for diastereomers **2** and **4**–**8** are available in **S4** of the Supporting Information. Computational experimental procedures are reported in addition to chiral HPLC data for enantiomers **9** and **10**.

## Conflict of interest

The authors declare no conflict of interest.

1

## Supporting information

As a service to our authors and readers, this journal provides supporting information supplied by the authors. Such materials are peer reviewed and may be re‐organized for online delivery, but are not copy‐edited or typeset. Technical support issues arising from supporting information (other than missing files) should be addressed to the authors.

Supporting Information

## Data Availability

The data that support the findings of this study are available in the supplementary material of this article.
