# Supplementary material for: Application of 2D EXSY and qNMR Spectroscopy for Diastereomeric Excess Determination Following Chiral Resolution of β‐Lactams
Source: ChemistryOpen. 2022 Jul 25;12(6):e202200119. doi: 10.1002/open.202200119 (PMC10233219; doi:10.1002/open.202200119)
Supplement: Supplementary file 1 — Supporting Information [file OPEN-12-e202200119-s001.pdf]

ChemistryOpen

Supporting Information

Application of 2D EXSY and qNMR Spectroscopy for Diastereomeric Excess Determination Following Chiral Resolution of β-Lactams

Eavan C. McLoughlin,* John E. O'Brien, Cristina Trujillo, Mary J. Meegan, and Niamh M. O'Boyle*

Supplementary Index

Appendix S1: ^{1}H, ^{13}C NMR, 2D EXSY/NOESY and VT data
Appendix S2: Chiral HPLC data for enantiomers 9 and 10
Appendix S3. Computational data
Appendix S4: Characterisation for non proline diastereomers

Appendix S1: ^{1}H & ^{13}C NMR data

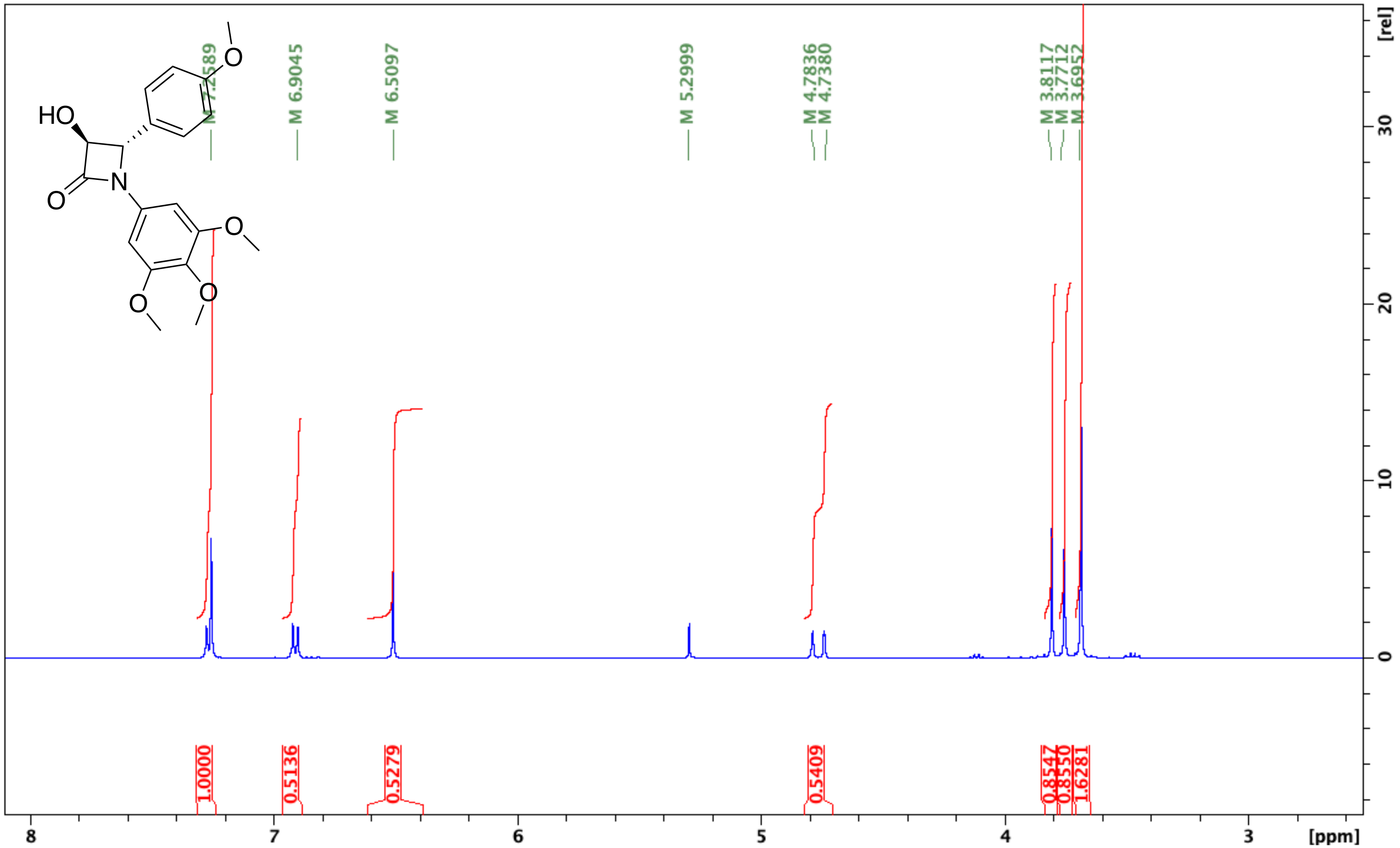

S1.1 ^{1}H NMR of **1**, $CDCl_3$, 400 MHz. RP HPLC purity > 95%

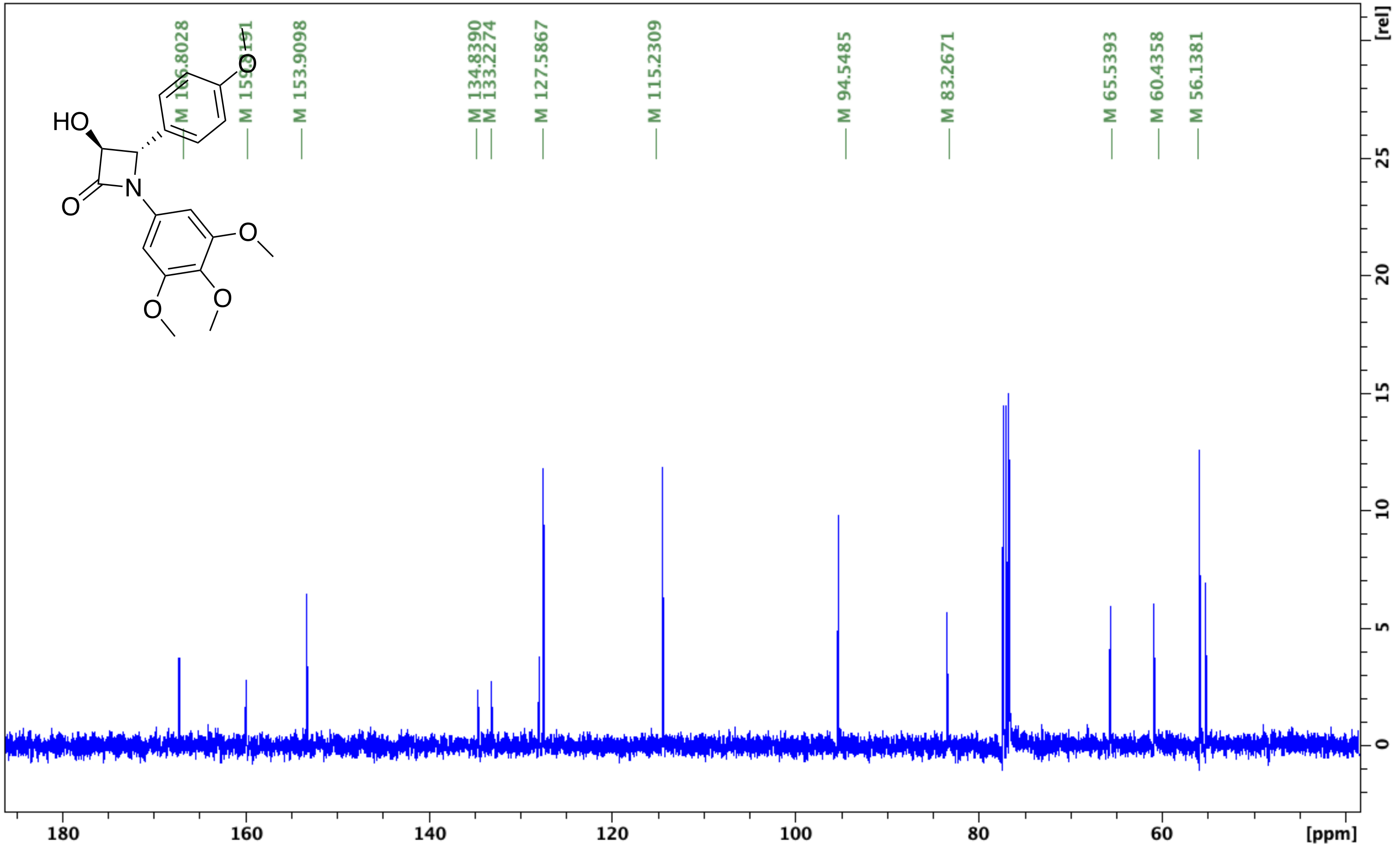

S1.2 ^{13}C NMR of **1,** $CDCl_3$, 100 MHz. RP HPLC purity > 95%

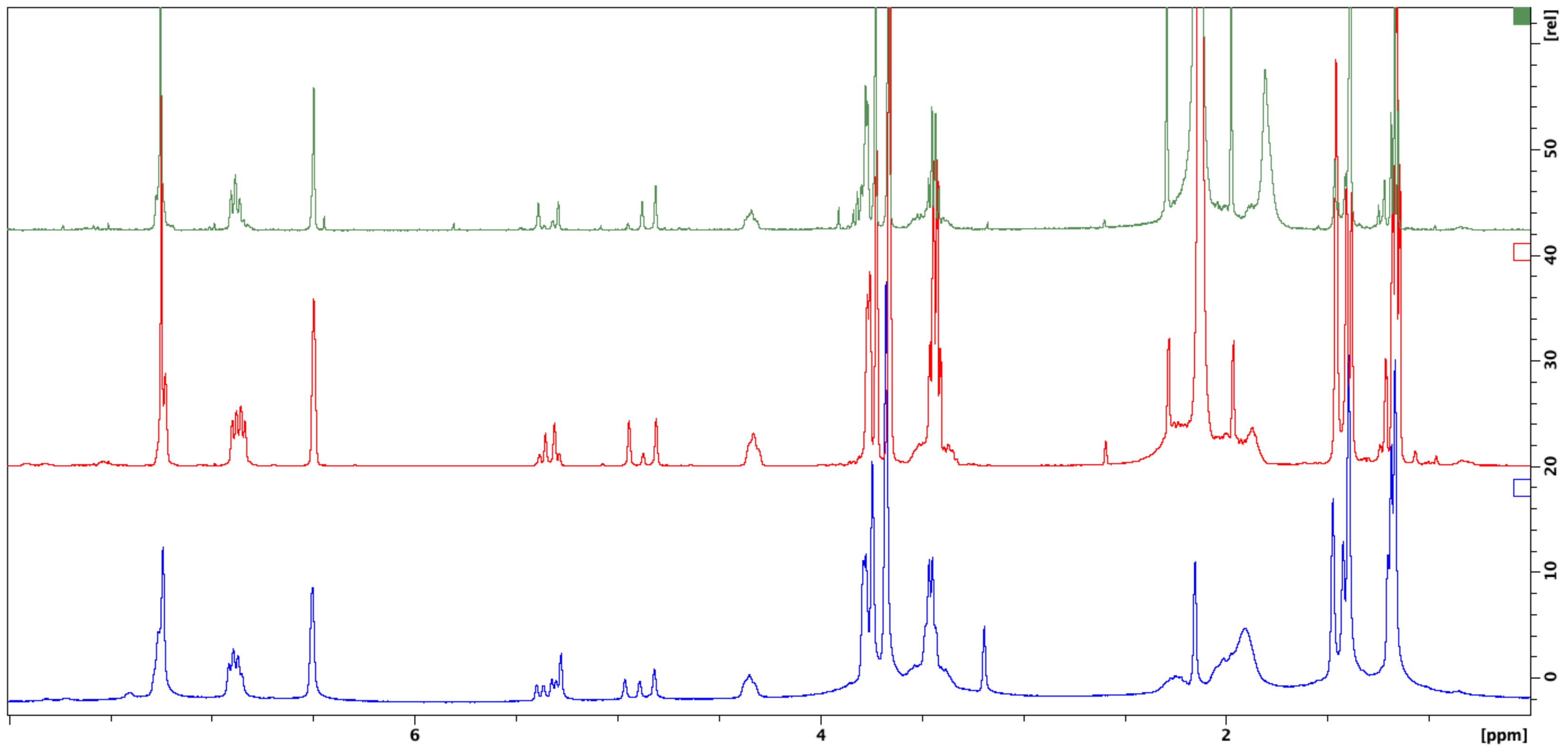

S1.3 [1] H NMR for diastereomers of **2** at 400 MHz, CDCl$_3$. **Blue:** Diastereomer mixture **2, red: 2DS1, green: 2DS2**

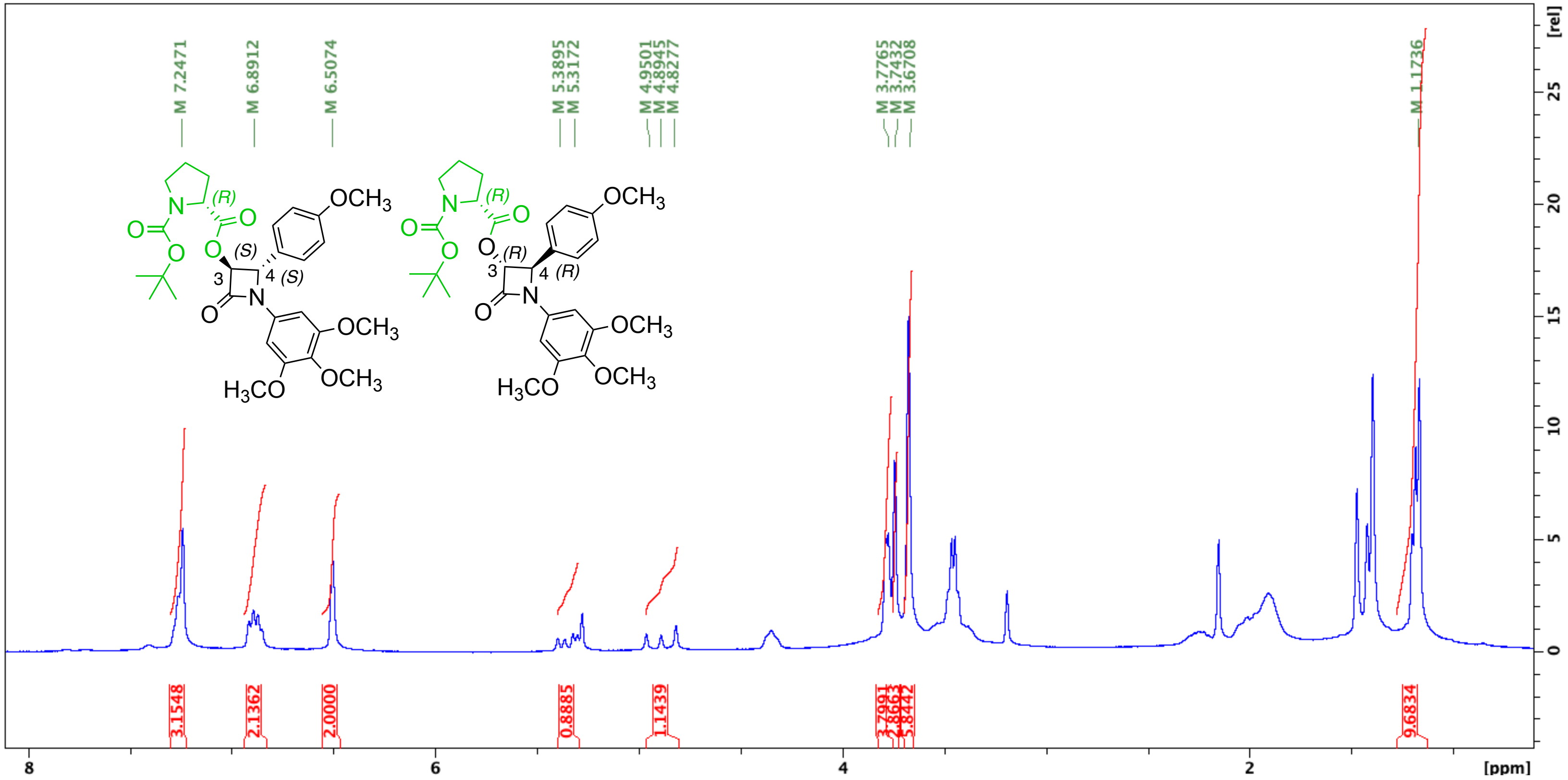

S1.4 ^{1}H NMR for diastereomer of **2** at 400 MHz, $CDCl_3$. Rotamer and diastereomer resonances observed in H_3 and H_4 region from 4.9 -5.4 ppm. RP-HPLC purity 85%

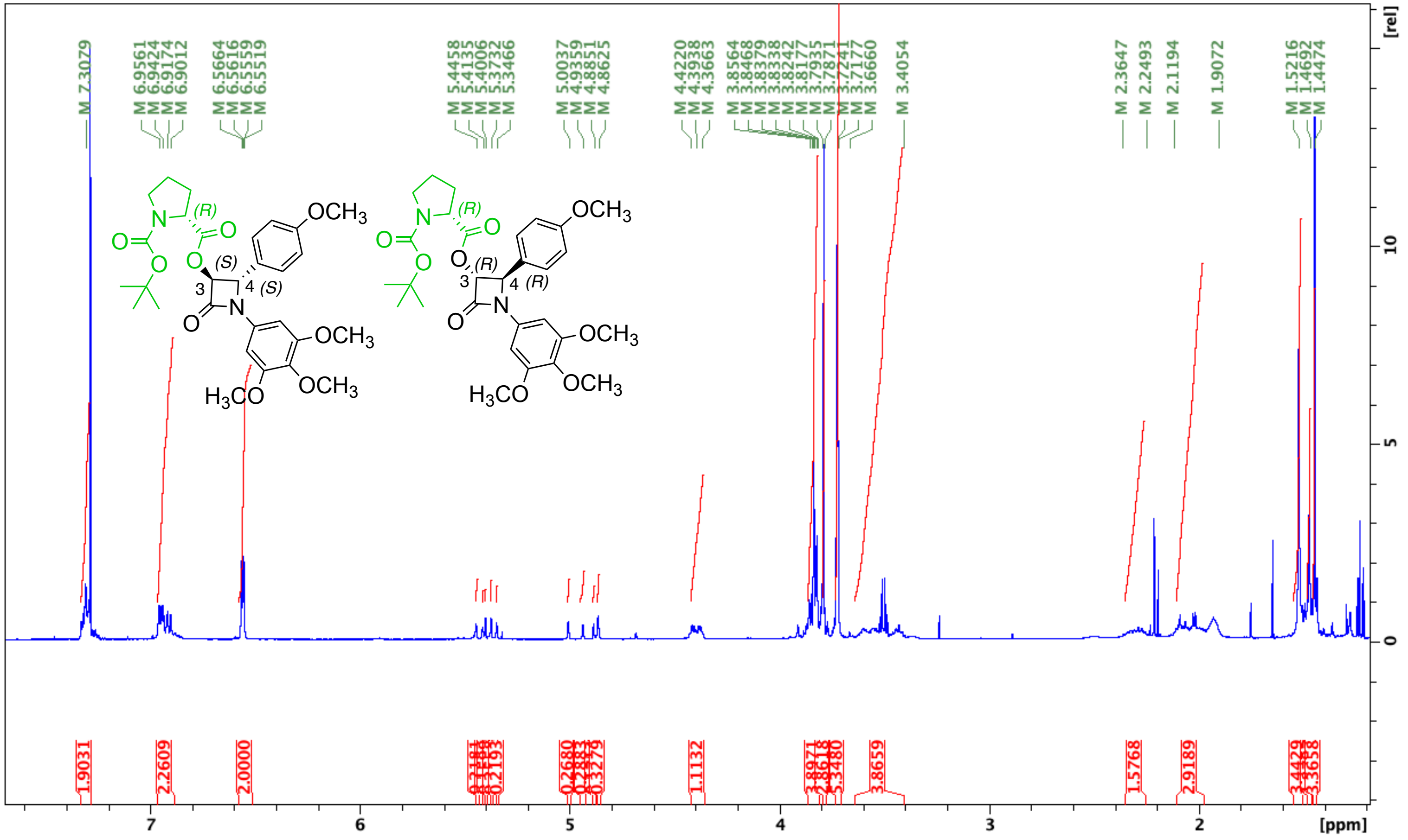

S1.5 [1] H NMR for diastereomers **2** at 600 MHz, $CDCl_3$. Impurities present from 0-1 ppm. RP-HPLC purity: 85-88%

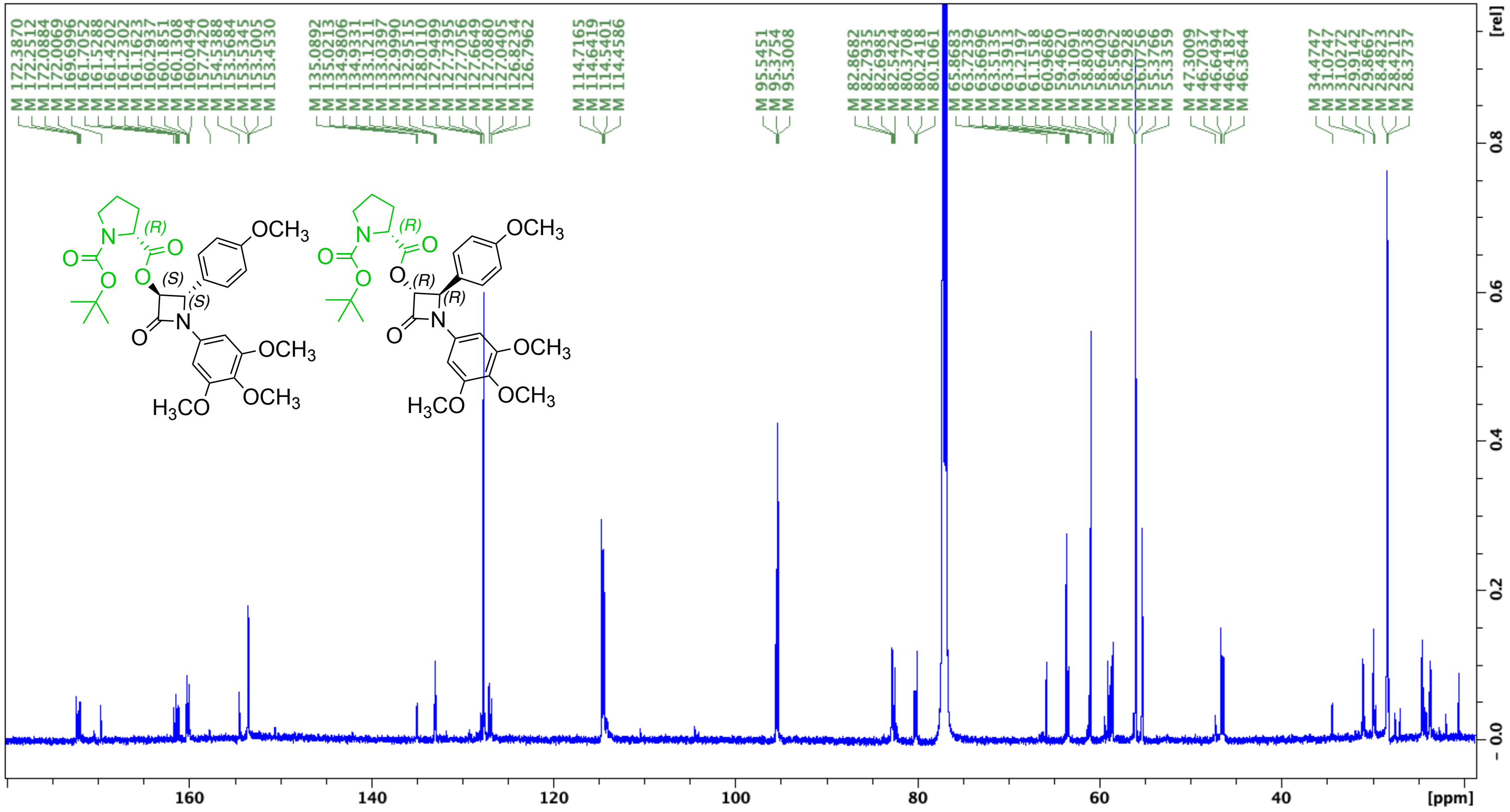

S1.6 ^{13}C NMR for diastereomers **2** at 100 MHz, $CDCl_3$. Impurities present from 0-15 ppm. RP-HPLC purity: 85-88%

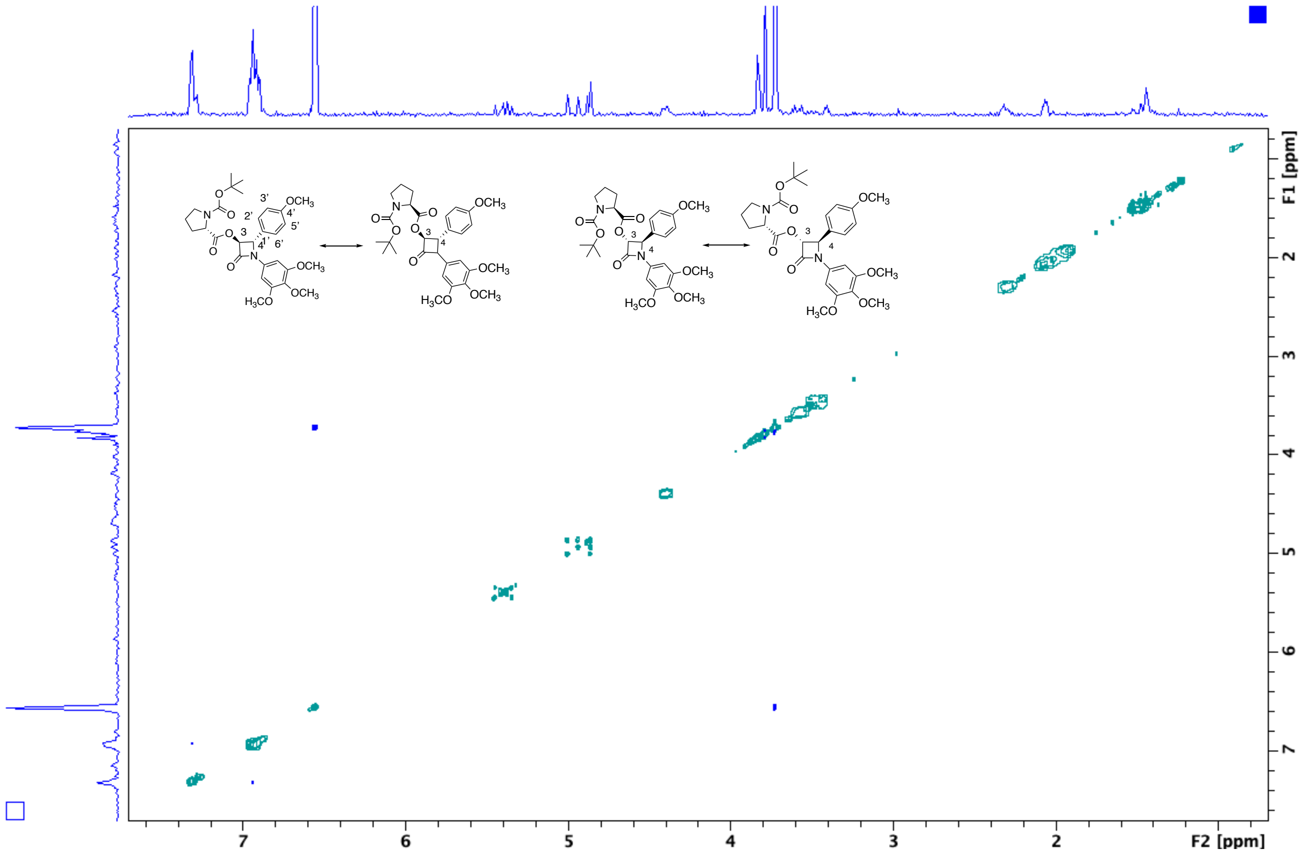

S1.7 2D EXSY spectrum for **2,** $CDCl_3$, 400 MHz. Slowest exchange observed for H_3 and H_4 as cross peaks from 4.5-4.5 ppm. Faster exchange also observed with minor evidence of cross peaks in B ring region for $H_{3''}$ and $H_{5''}$ at 6.90 -7 ppm.

A B

a b c d

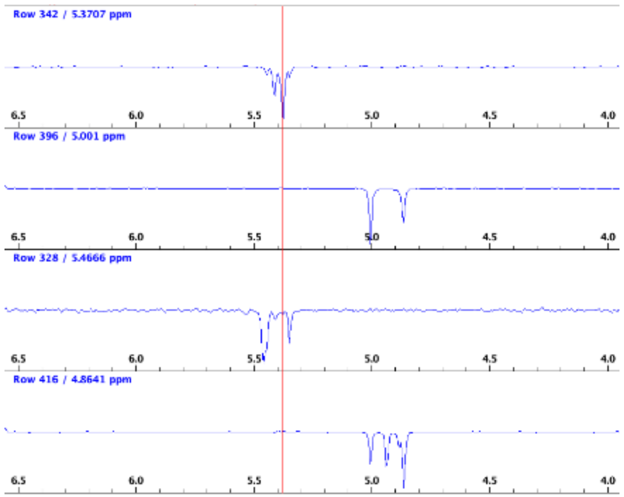

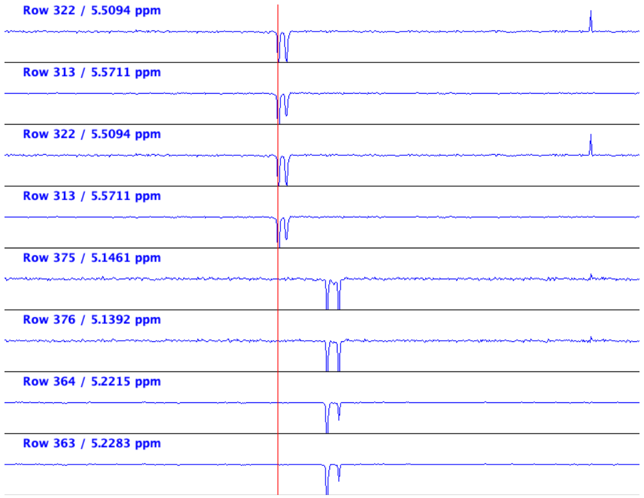

S1.8: Manual phasing of 2D EXSY spectra for **2** **A**: $CDCl_3$ at 400 MHz **a: 2DS1** (H_3) **b**:**2DS1** (H_4), **c: 2DS2** (H_3) and **d: 2DS2** (H_4). **B:** DMSO-d_6 at 400 MHz demonstrating identical anti-phase signals for **2DS1** and **2DS2**

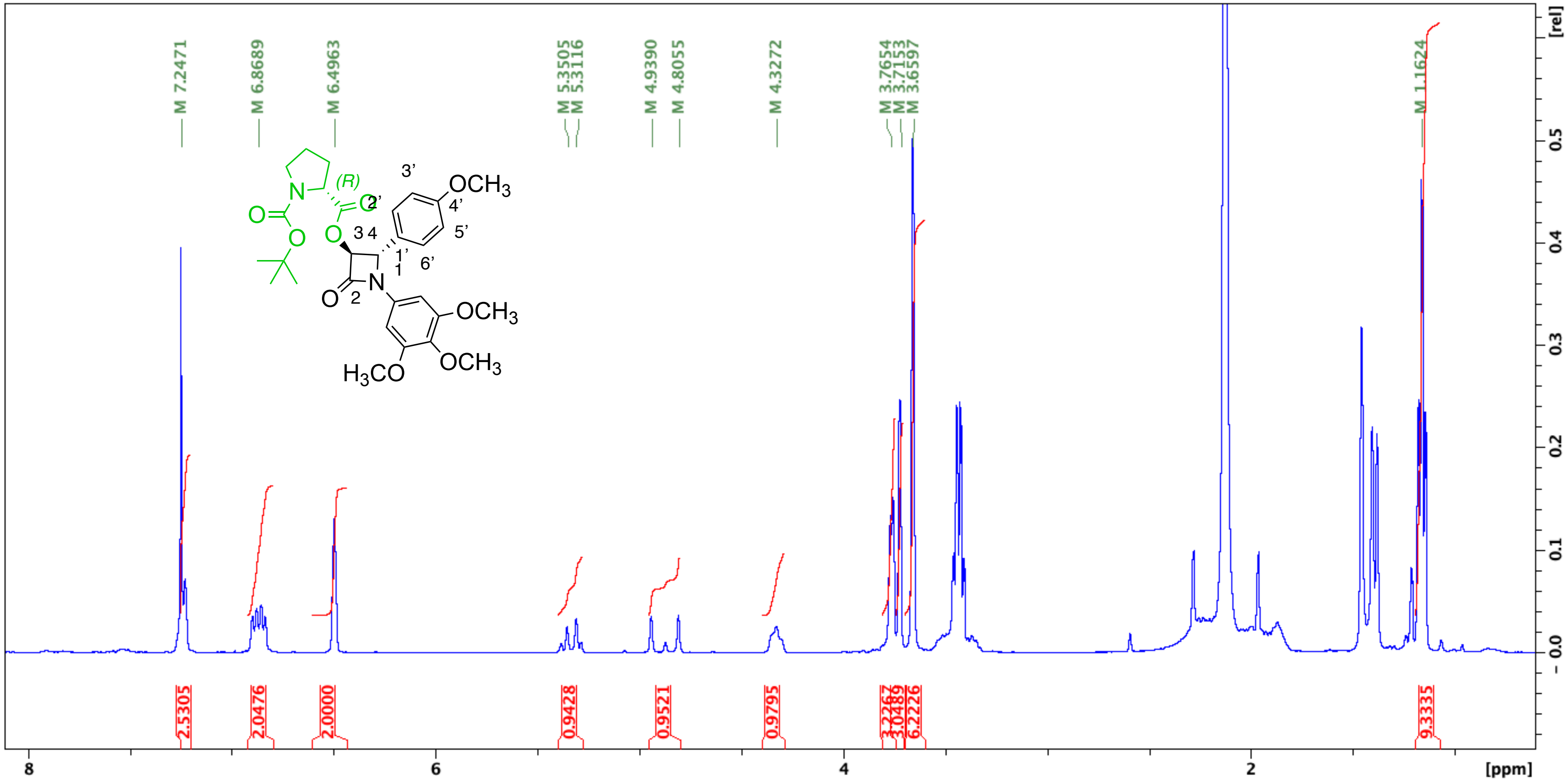

S1.9 [1] H NMR for diastereomer **2DS1** at 400 MHz, $CDCl_3$. RP-HPLC purity: 85%. Rotameric resonances observed in H_3 and H_4 region in addition to B ring region for $H_{3'\&5'}$ at 6.87 ppm.

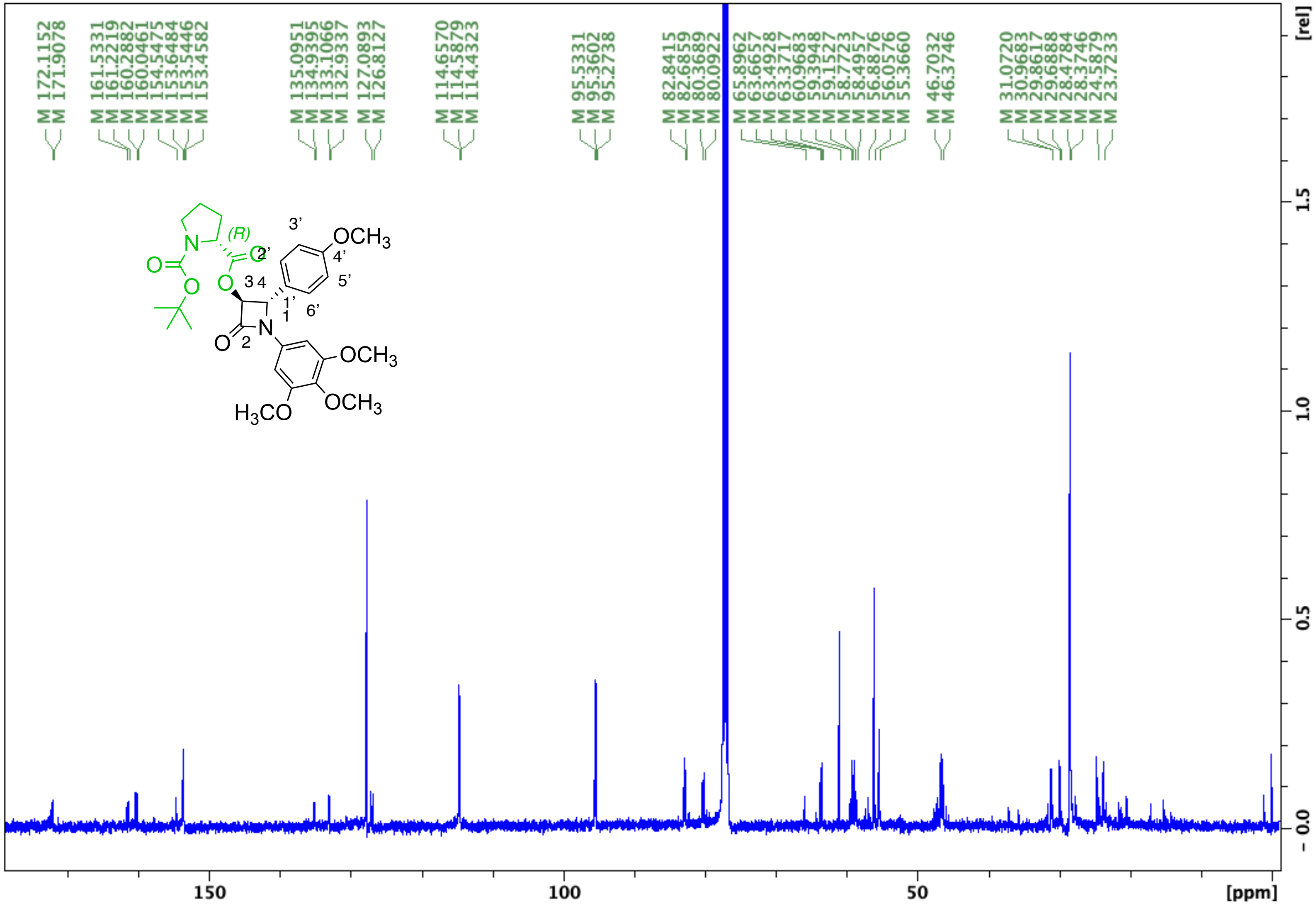

S1.10 ^{13}C NMR for diastereomer **2DS1** at 100 MHz, $CDCl_3$. RP-HPLC purity: 85%

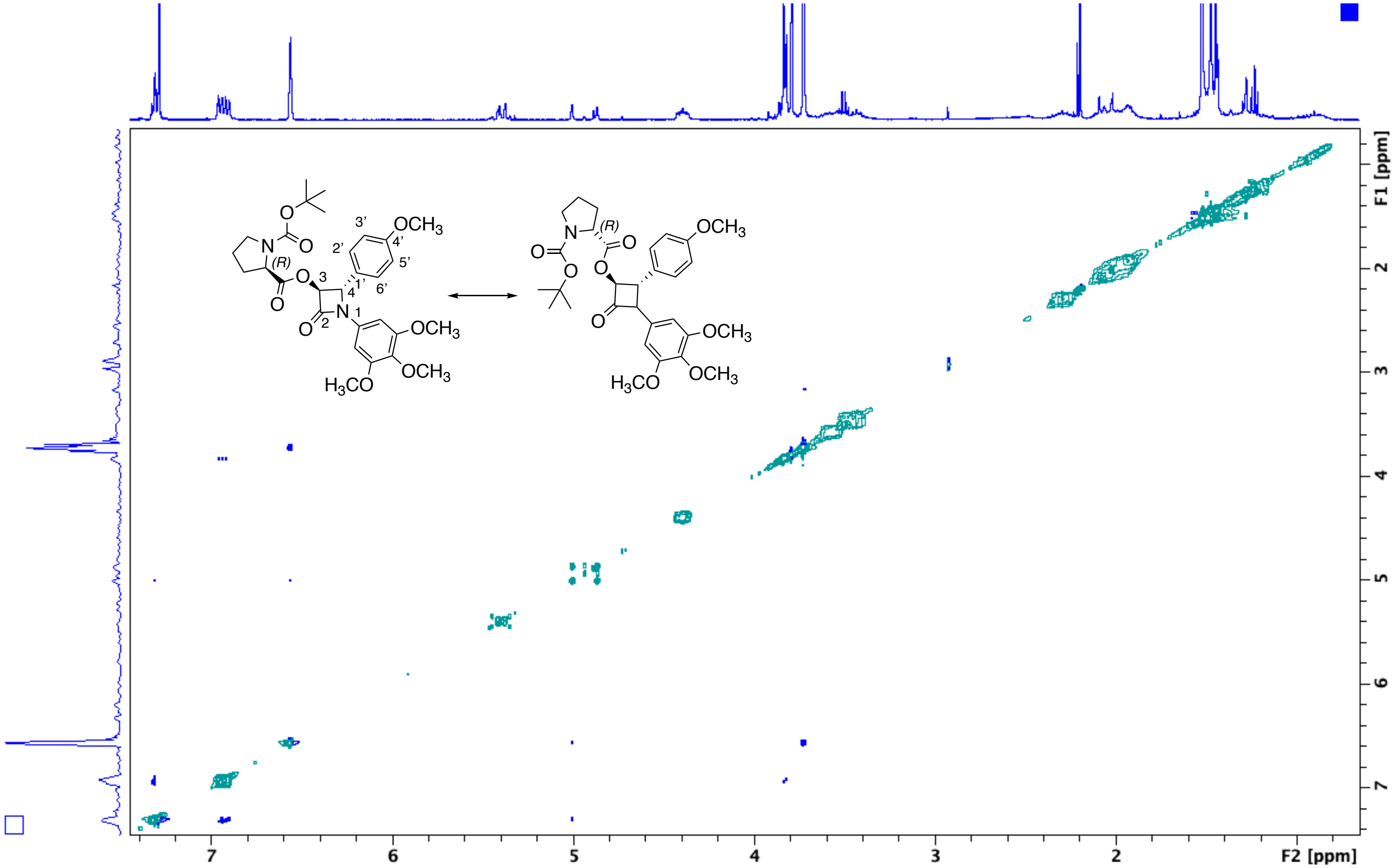

S1.11 2D EXSY spectrum for **2DS1** at 400 MHz, $CDCl_3$. Rotamers observed for H_3 and H_4 (4.5-5.5 ppm) and $H_{2'}$ and $H_{6'}$ (6.9-7.1 ppm). RP-HPLC purity 95%/

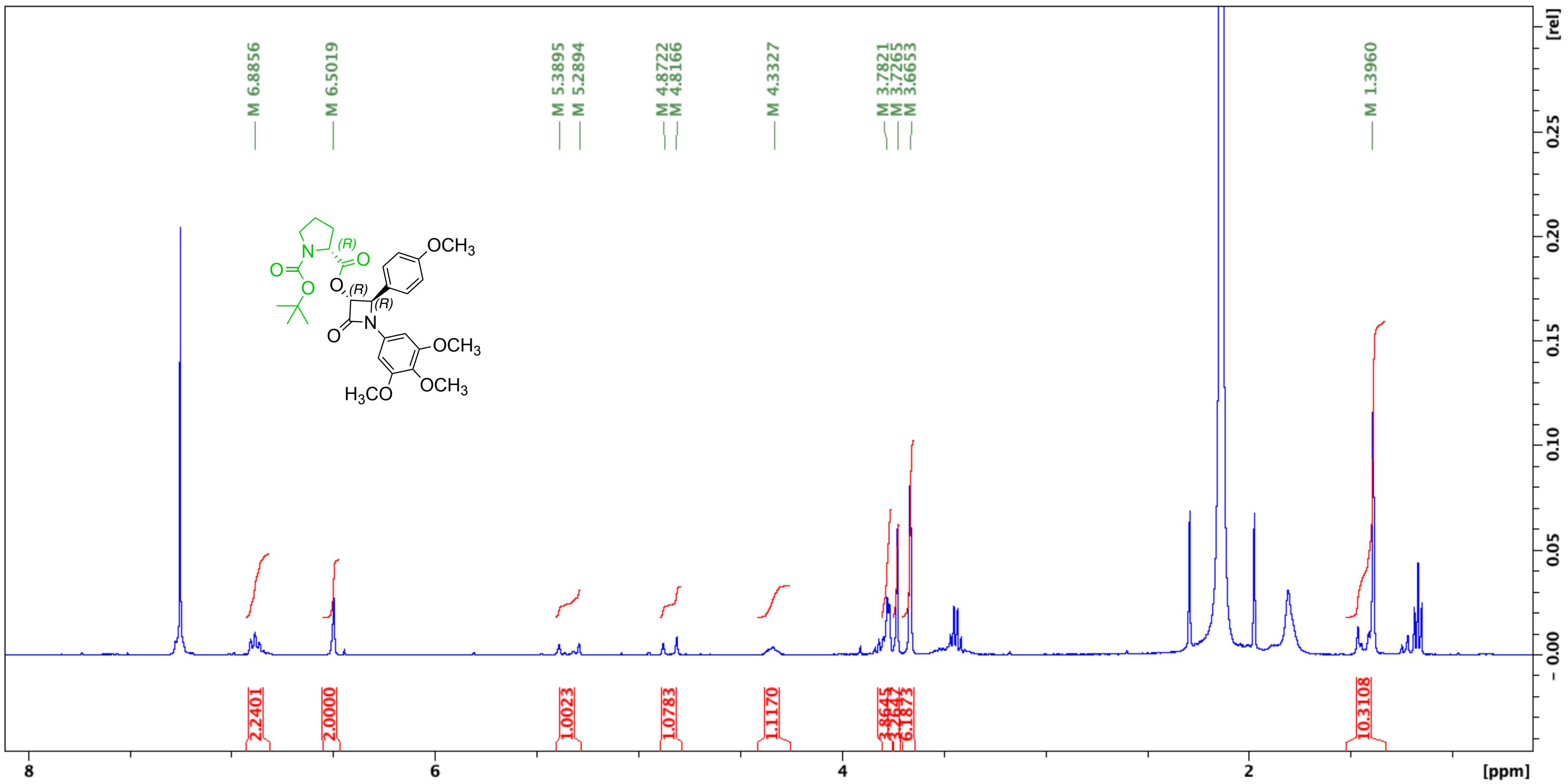

S1.12 [1] H NMR for diastereomer **2DS2** at 400 MHz, $CDCl_3$. RP-HPLC purity: 95 % (residual ethyl acetate in spectrum)

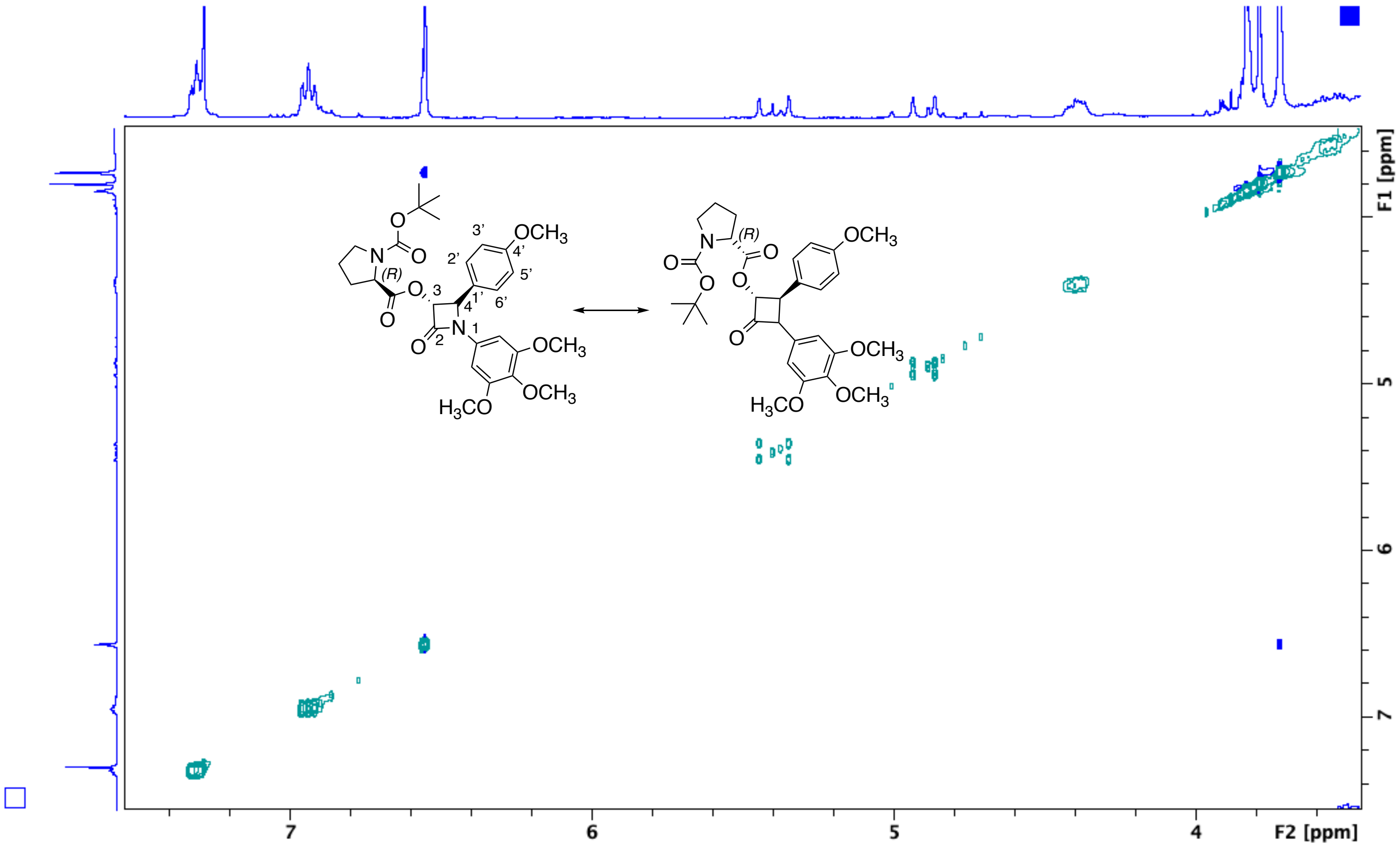

S1.13 . 2D EXSY spectrum for **2DS2** at 400 MHz, $CDCl_3$. Rotamers observed for H_3 and H_4 (4.5-5.5 ppm) and $H_{2'}$ and $H_{6'}$ (6.9-7.1 ppm). Structures of **2DS2** illustrated for reference. (The same trend applies to all spectra for proline diastereomers)

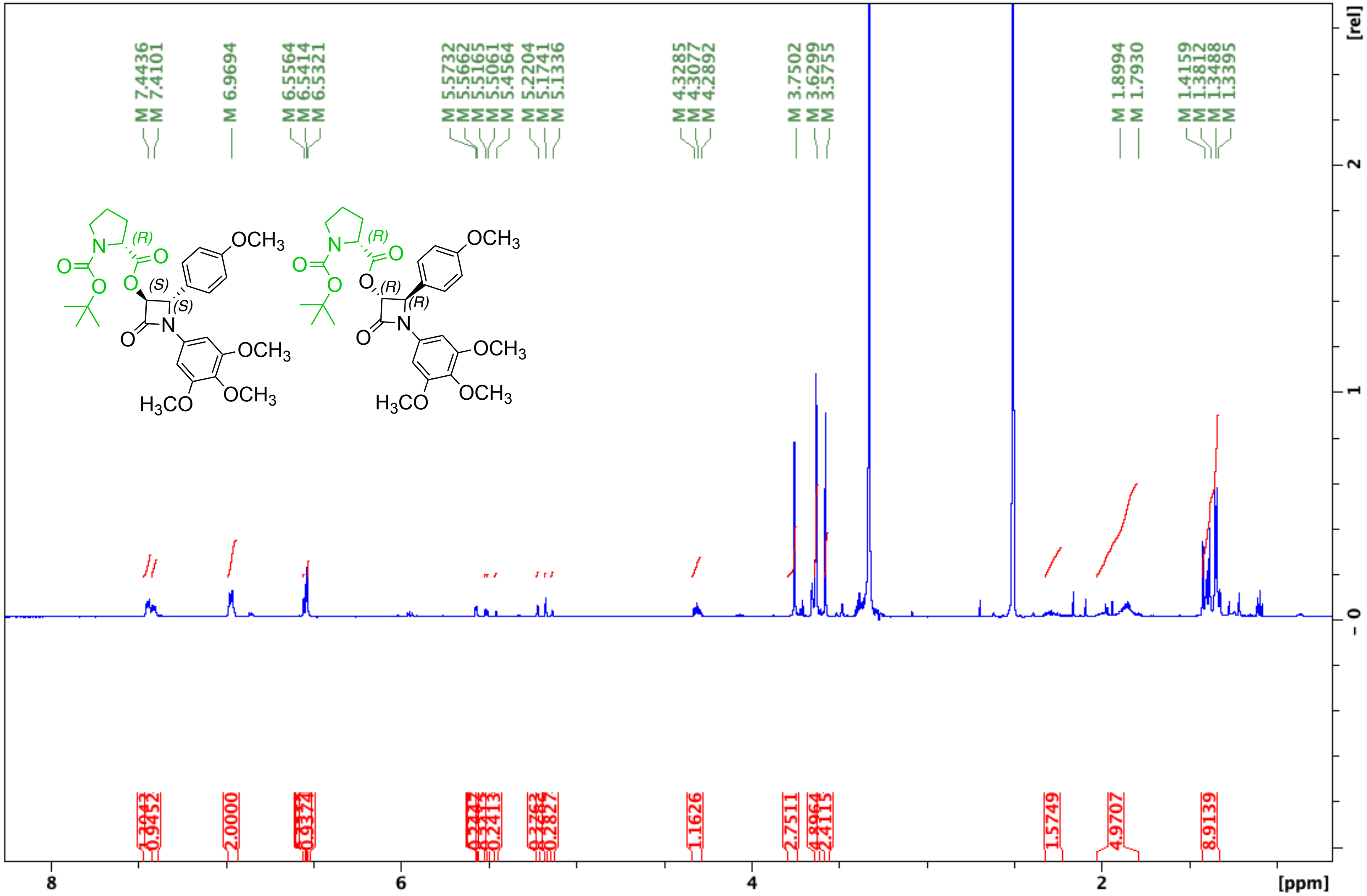

S1.14 ^{1}H NMR for diastereomer mixture **2** DMSO-d_6, 600 MHz. RP-HPLC purity: 85%

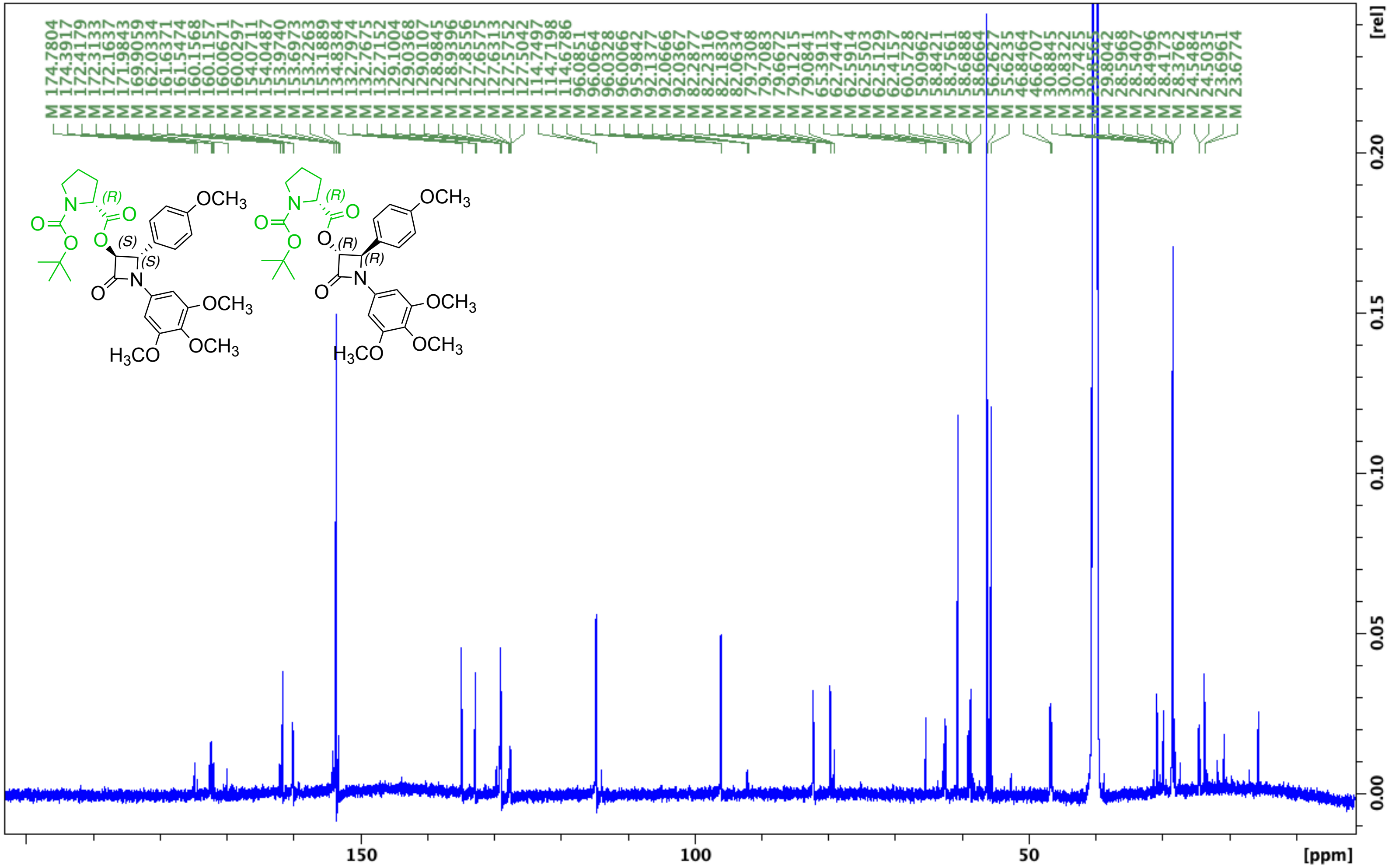

S1.15 ^{13}C NMR for diastereomer of **2** DMSO-d_6, 100 MHz. RP-HPLC purity 85%

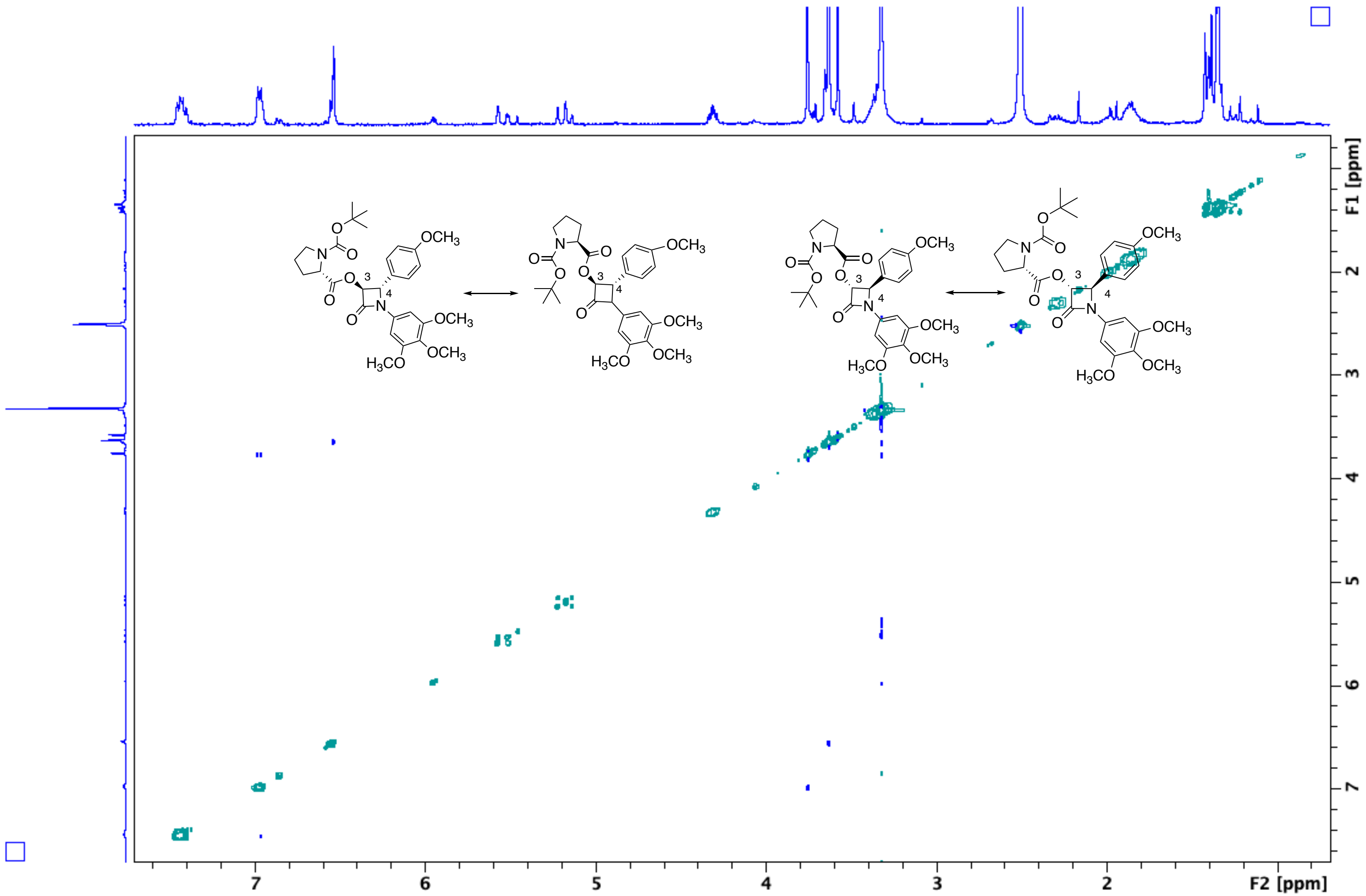

S1.16 2D EXSY spectrum for **2** DMSO-d_6, 400 MHz. Rotamers only observed for H_3 and H_4 from 5-6 ppm.

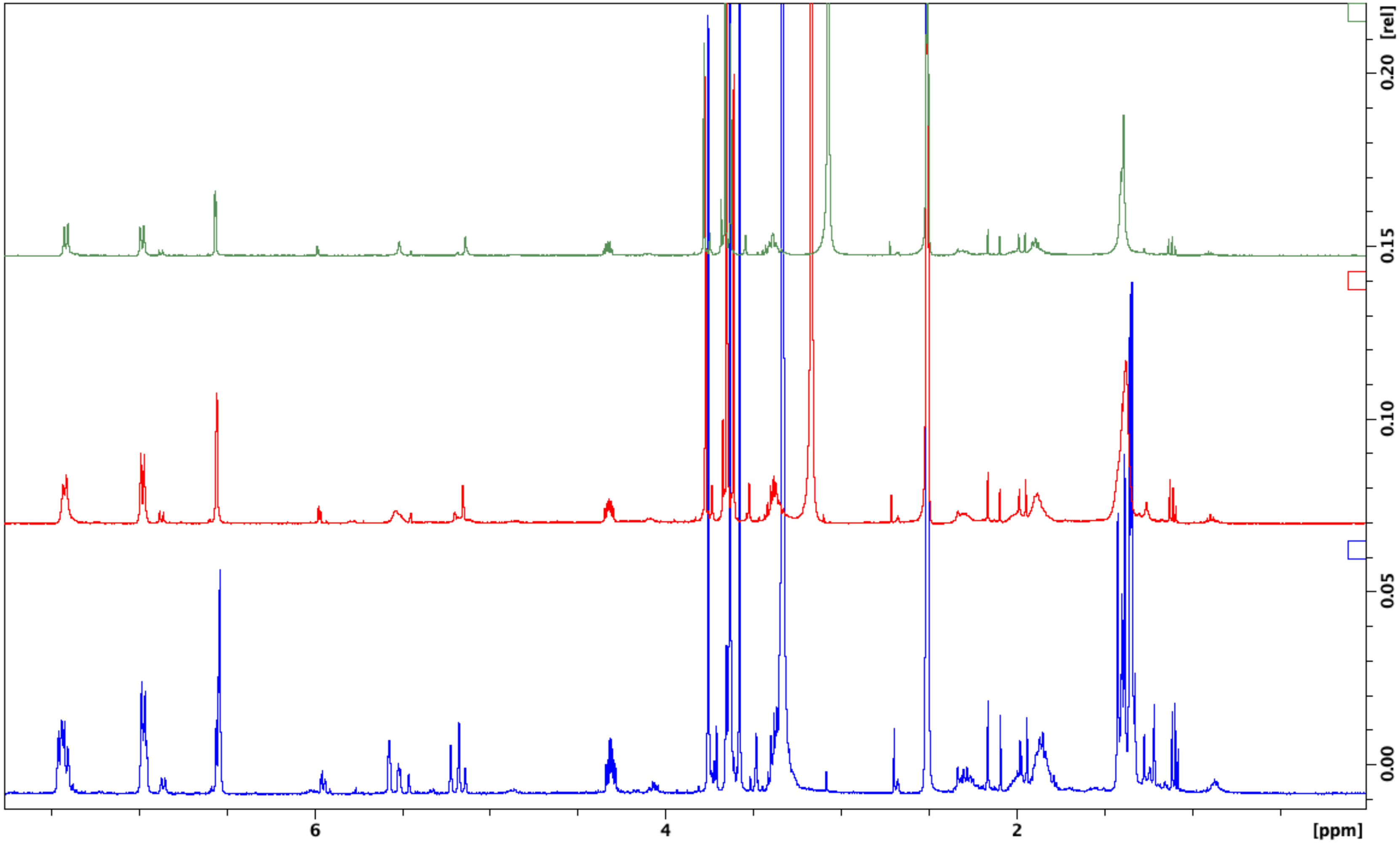

S1.17 VT Experiment for **2** DMSO-d_6, 400 MHz. Blue: 25 °C, Red: 60 °C, Green: 80 °C.

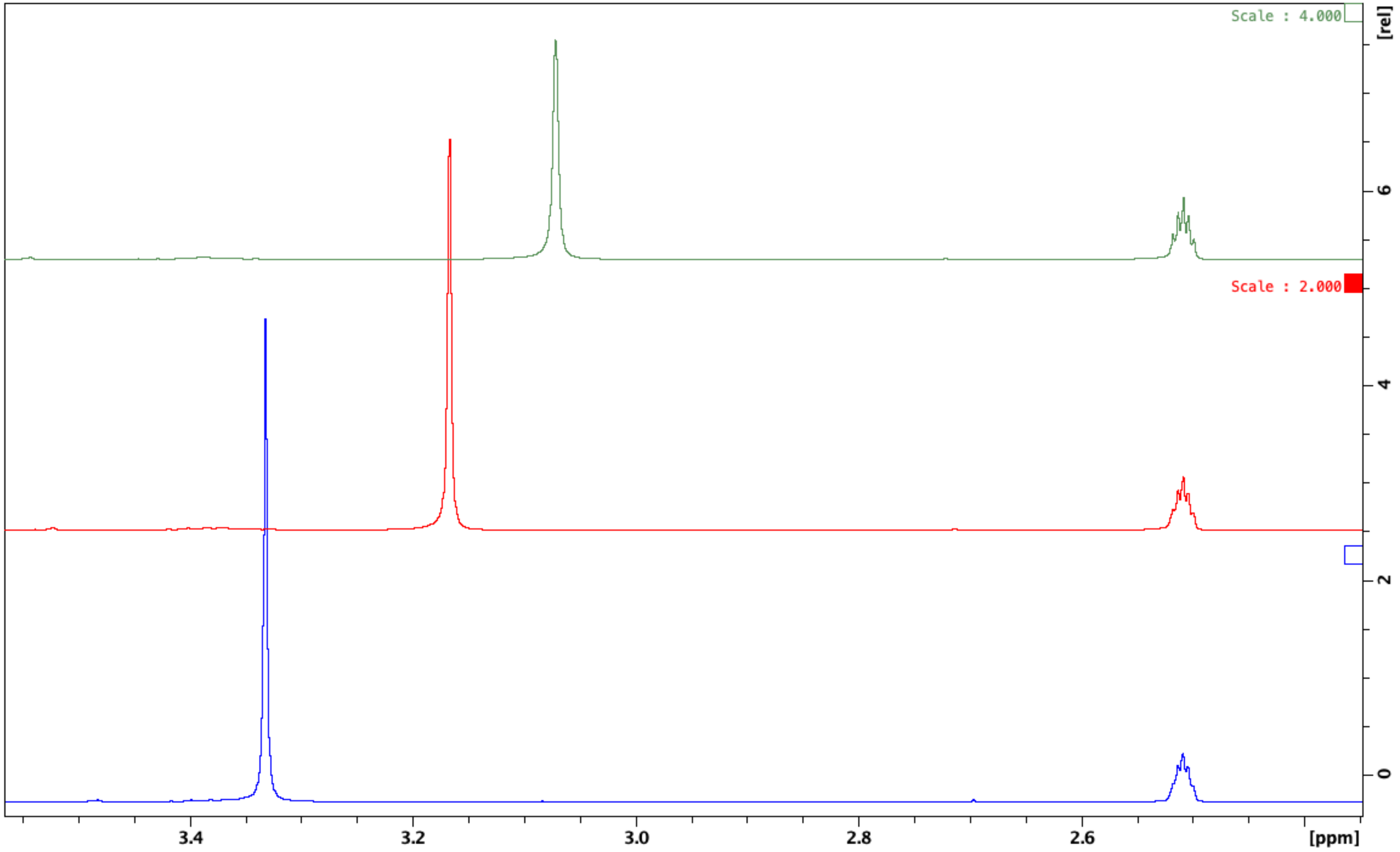

S1.18 VT experiment for diastereomer mixture of **2** illustrating H_2O signal at δ 3.3 ppm in DMSO-d_6 and shifting upfield while DMSO signal δ 2.5 ppm remains stationary. Blue: 25 °C, Red: 60 °C, Blue: 80 °C. Water at 25 °C resonates at δ 3.3 ppm in DMSO-d_6 and shifts upfield with increasing temperature while the DMSO signal at δ 2.5 ppm remains stationary. This confirms the progressive increase in temperature required for VT studies and ensures the validity of of results.

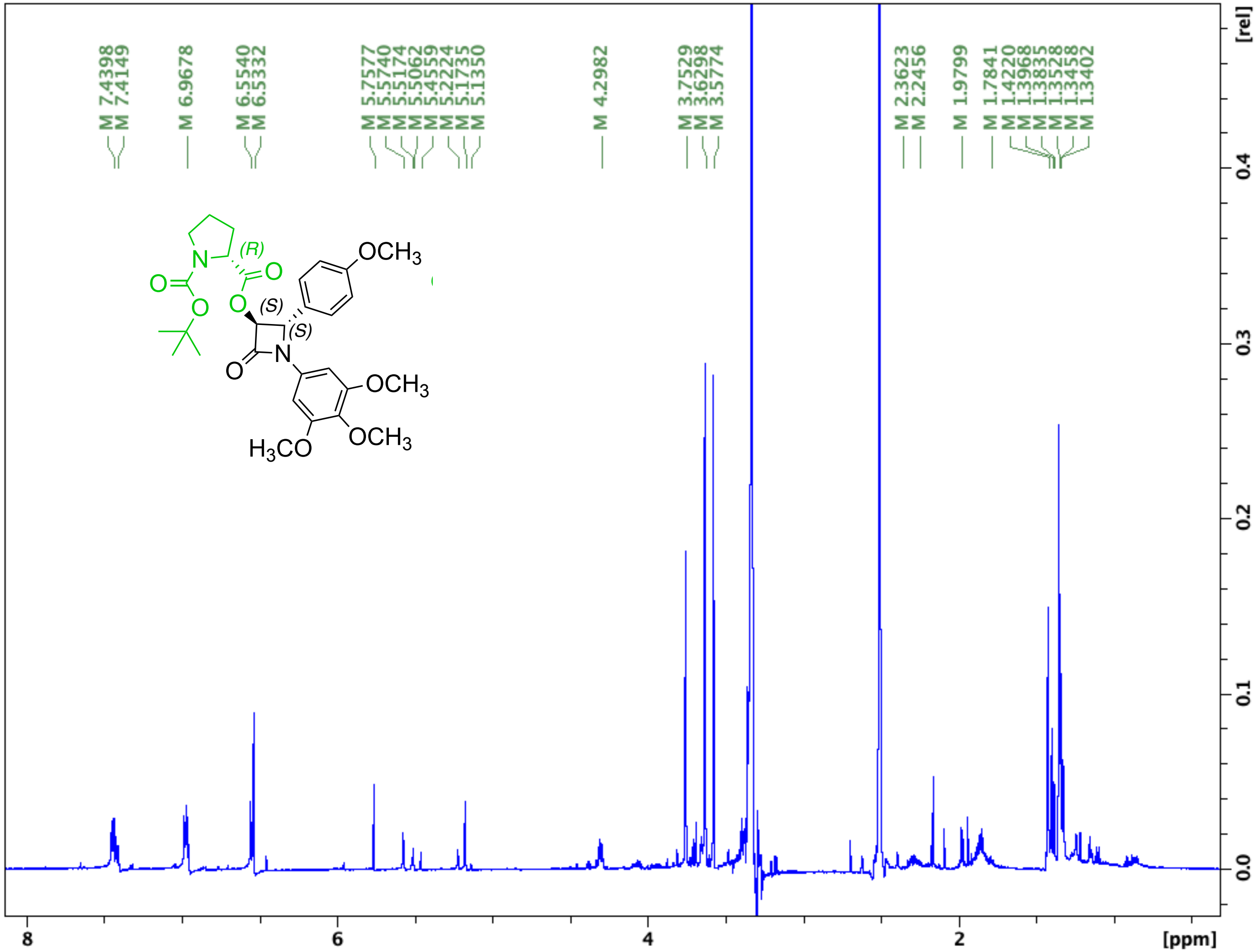

S1.19 [1] H NMR for diastereomer **2DS1,** DMSO-d_6, 600 MHz. RP-HPLC purity: 85%

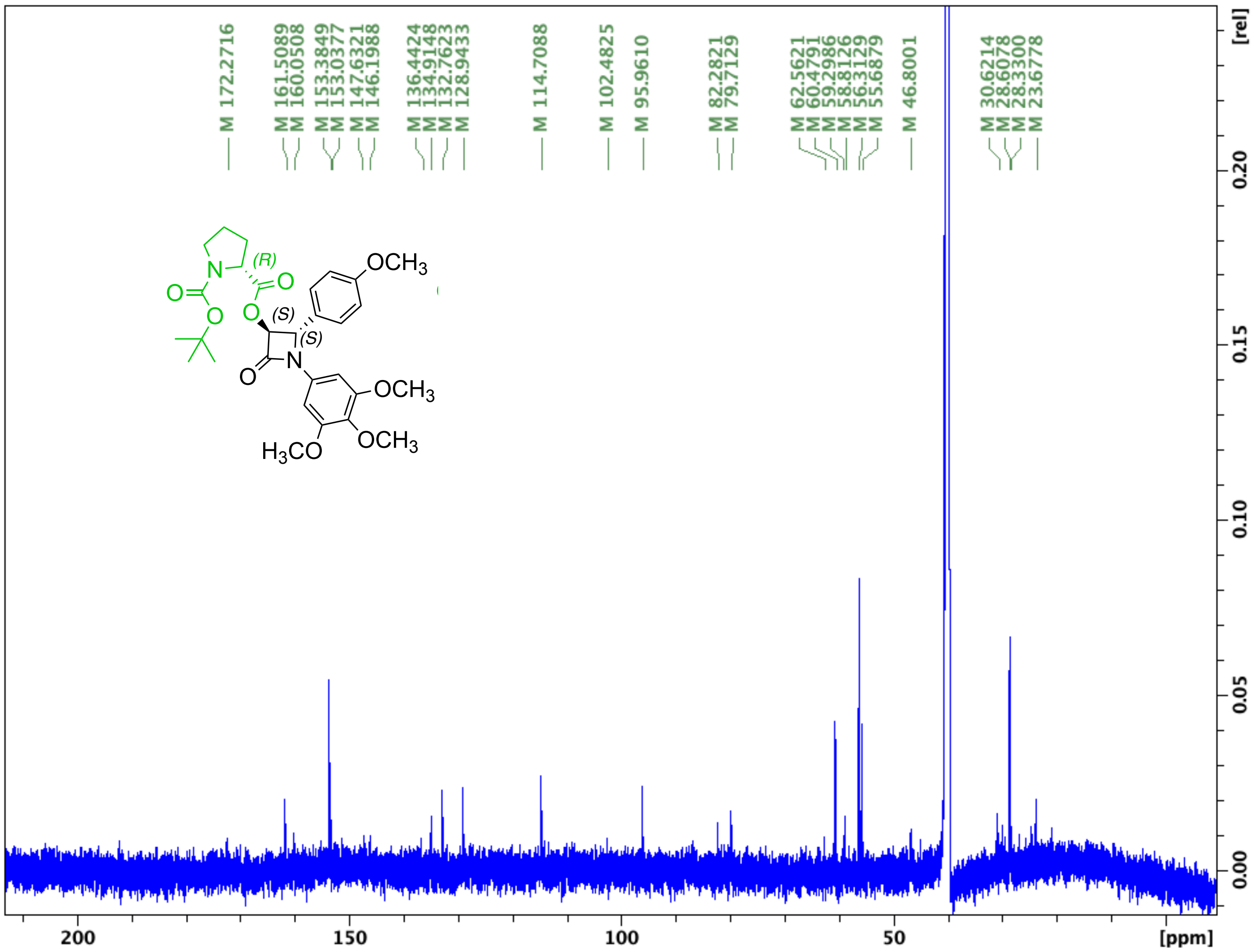

S1.20 [13] C NMR for diastereomer **2DS1**, DMSO-d_6, 100 MHz. RP-HPLC purity: 85%

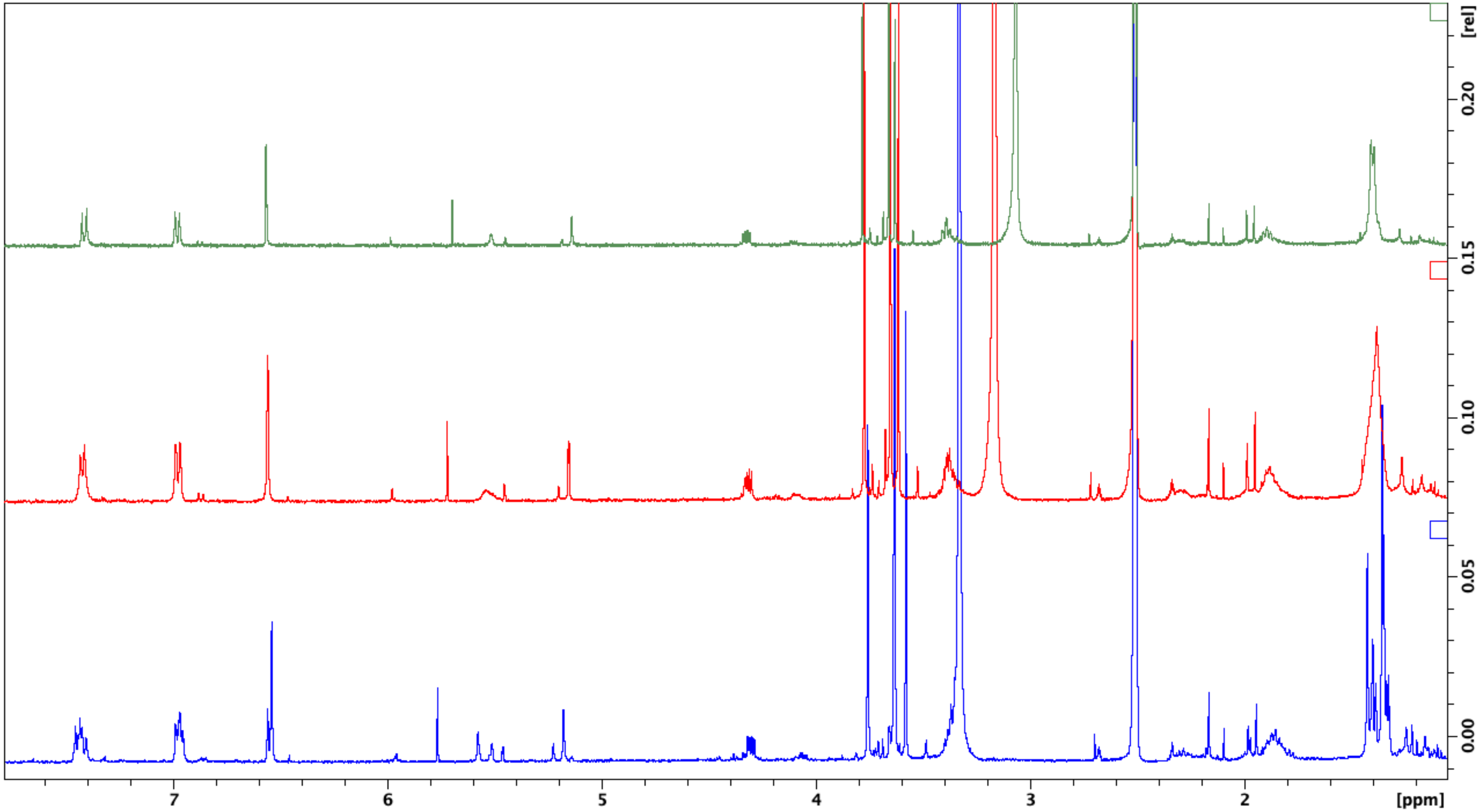

S1.21 VT experiment for diastereomer **2DS1,** DMSO-d_6, 400 MHz. Blue: 25 °C Red: 60 °C, Green: 80 °C

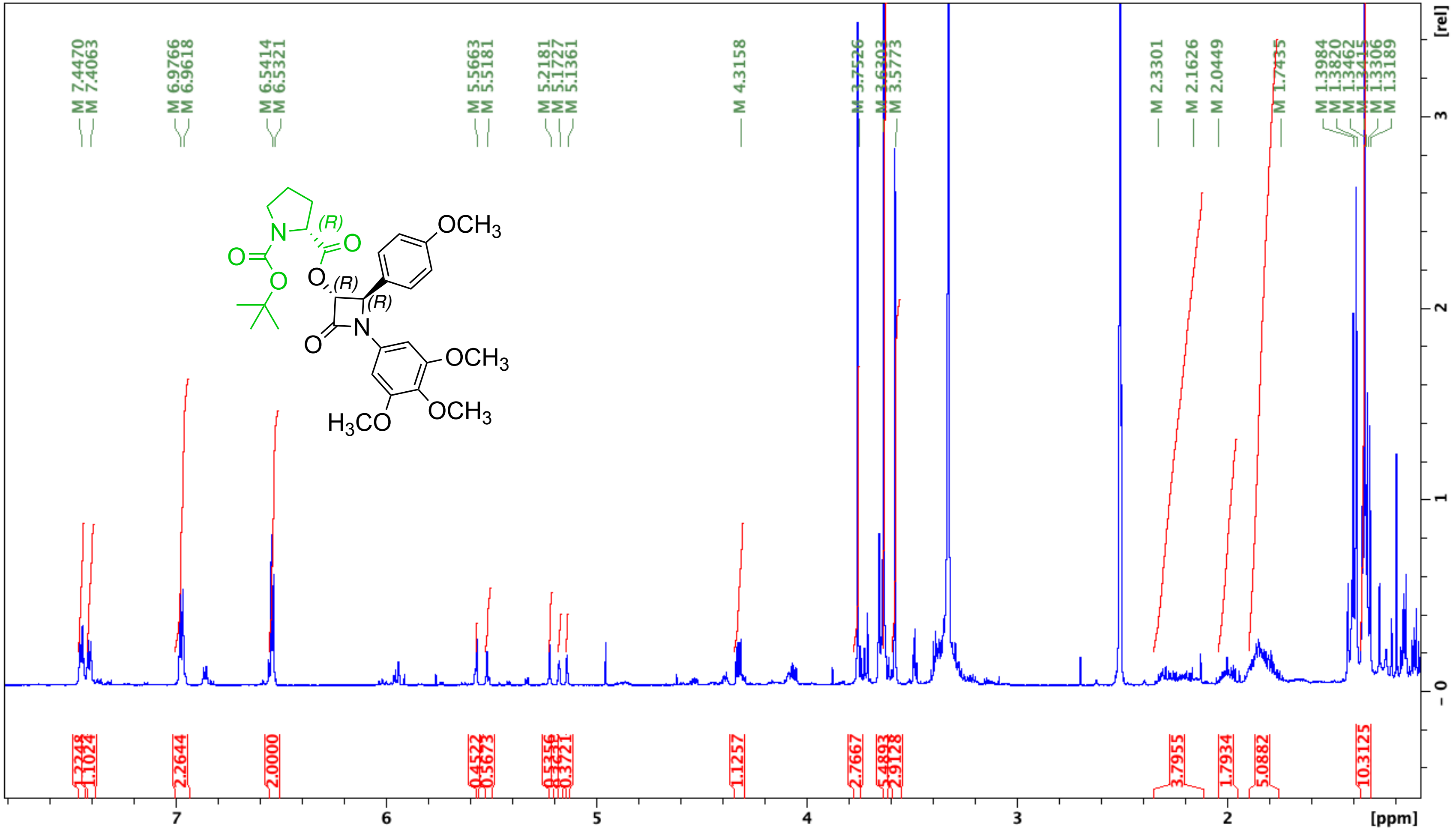

S1.22 S1. ^{1}H NMR for diastereomer **2DS2,** DMSO-d_6, 600 MHz. RP-HPLC purity: 95%. Impurities observed at 0-1.3 ppm on spectra above.

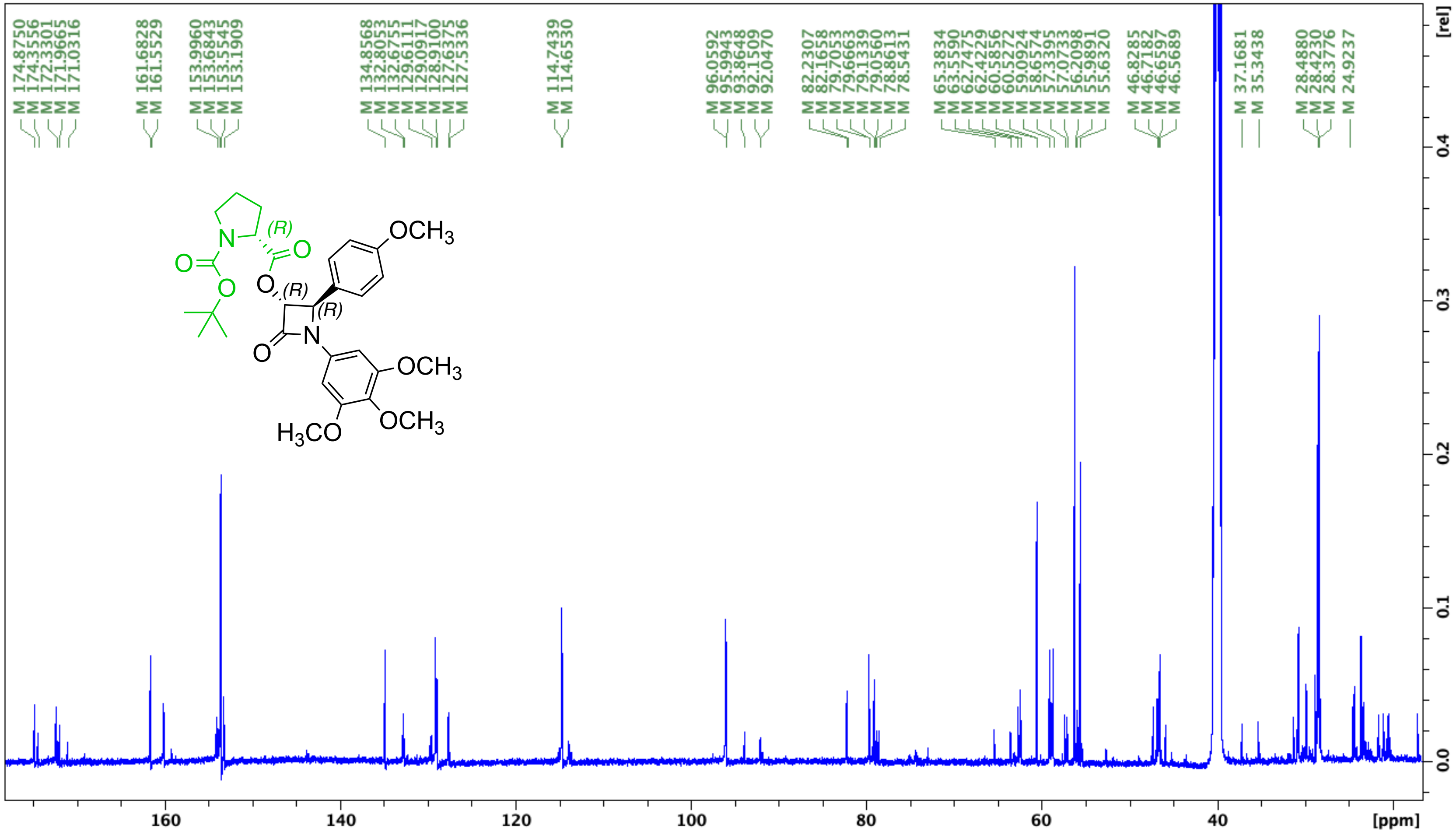

S1. 23 ^{13}C NMR for diastereomer **2DS2,** DMSO-d_6, 100 MHz. RP-HPLC purity 95%

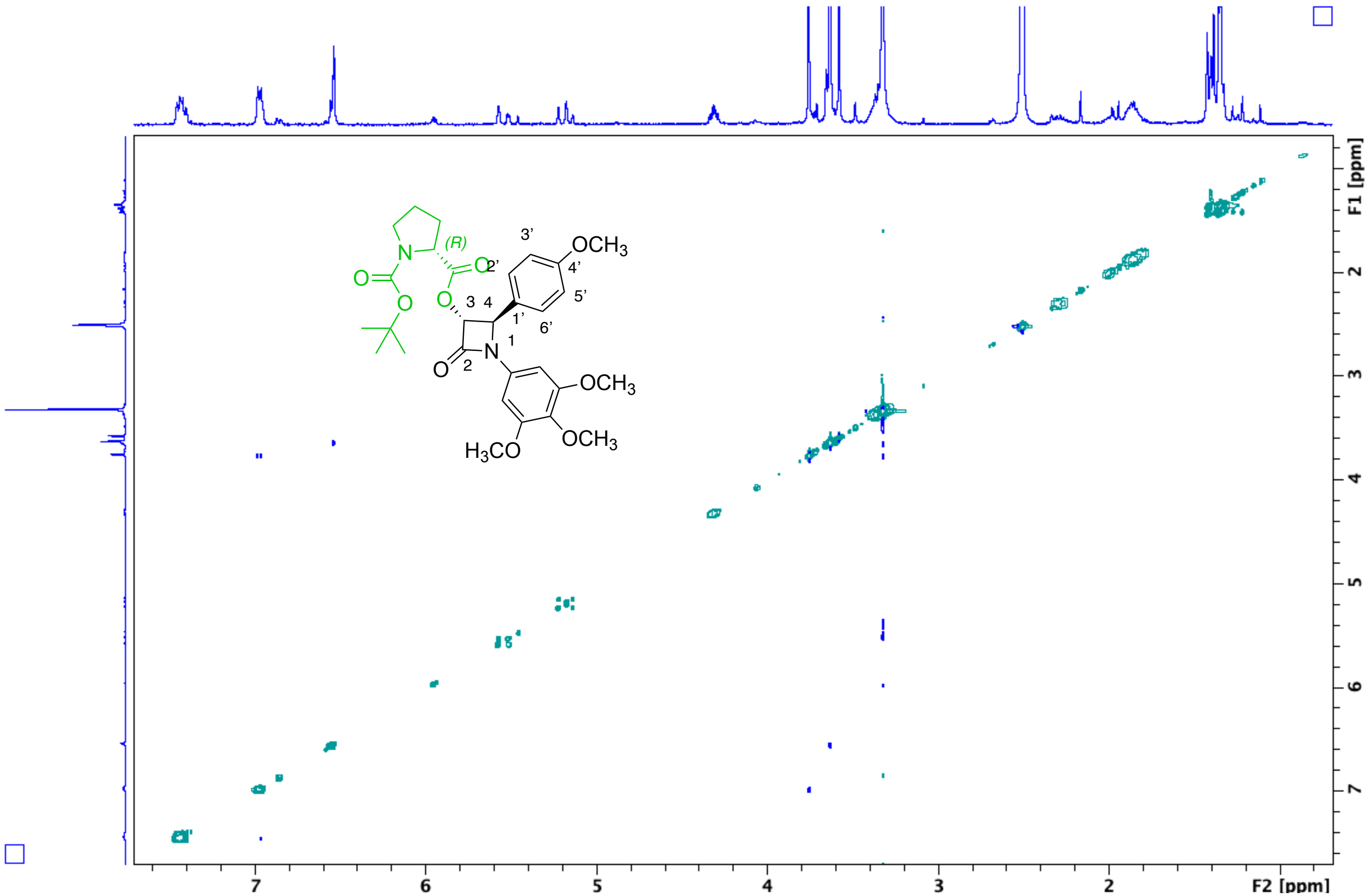

S1.24 2D EXSY at 25°C for **2DS2,** DMSO-d_6, 400 MHz. RP-HPLC purity 95%

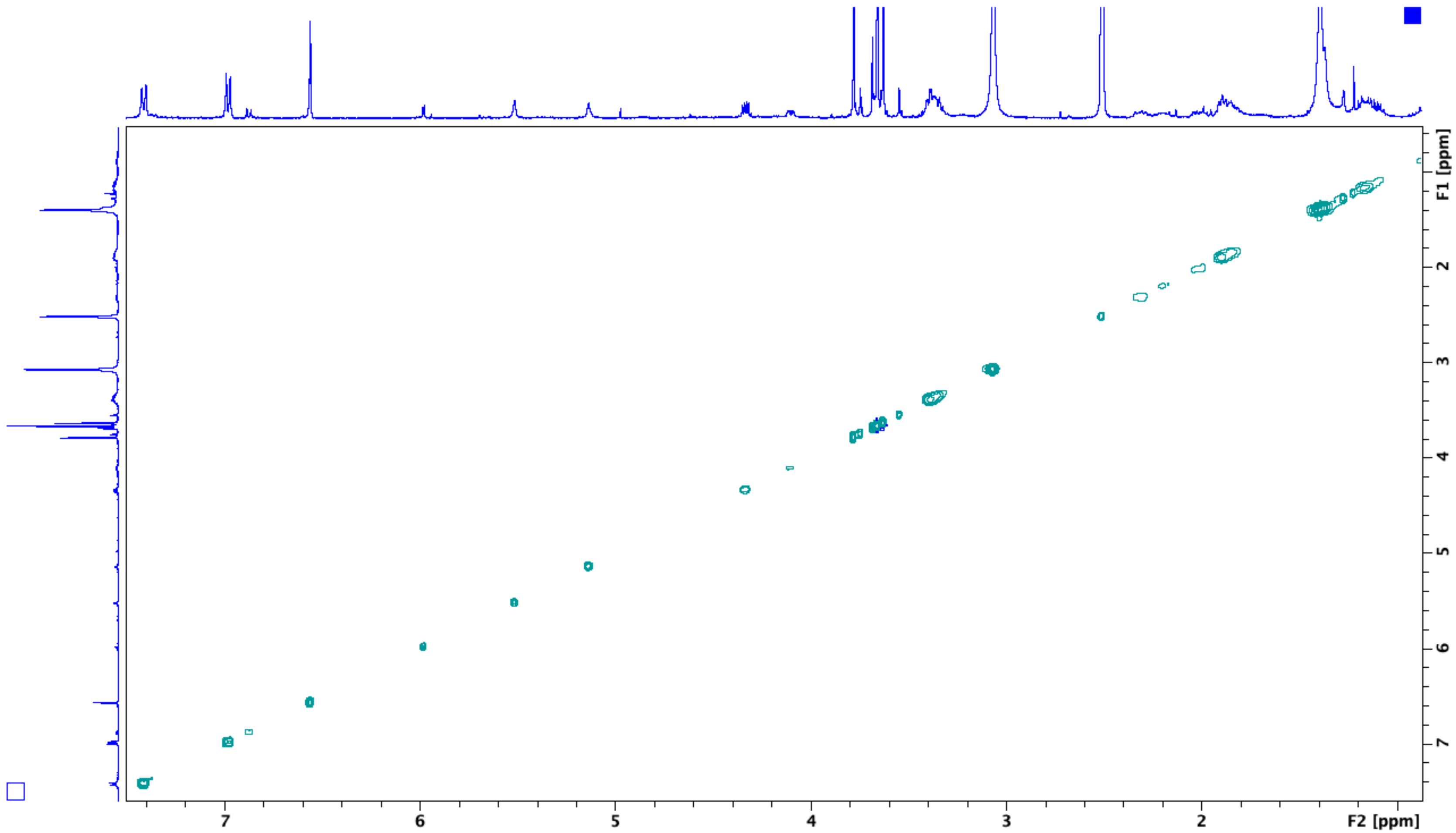

S1.25 2D EXSY at 85°C for **2DS2,** DMSO-d_6, 400 MHz. RP-HPLC purity 95%

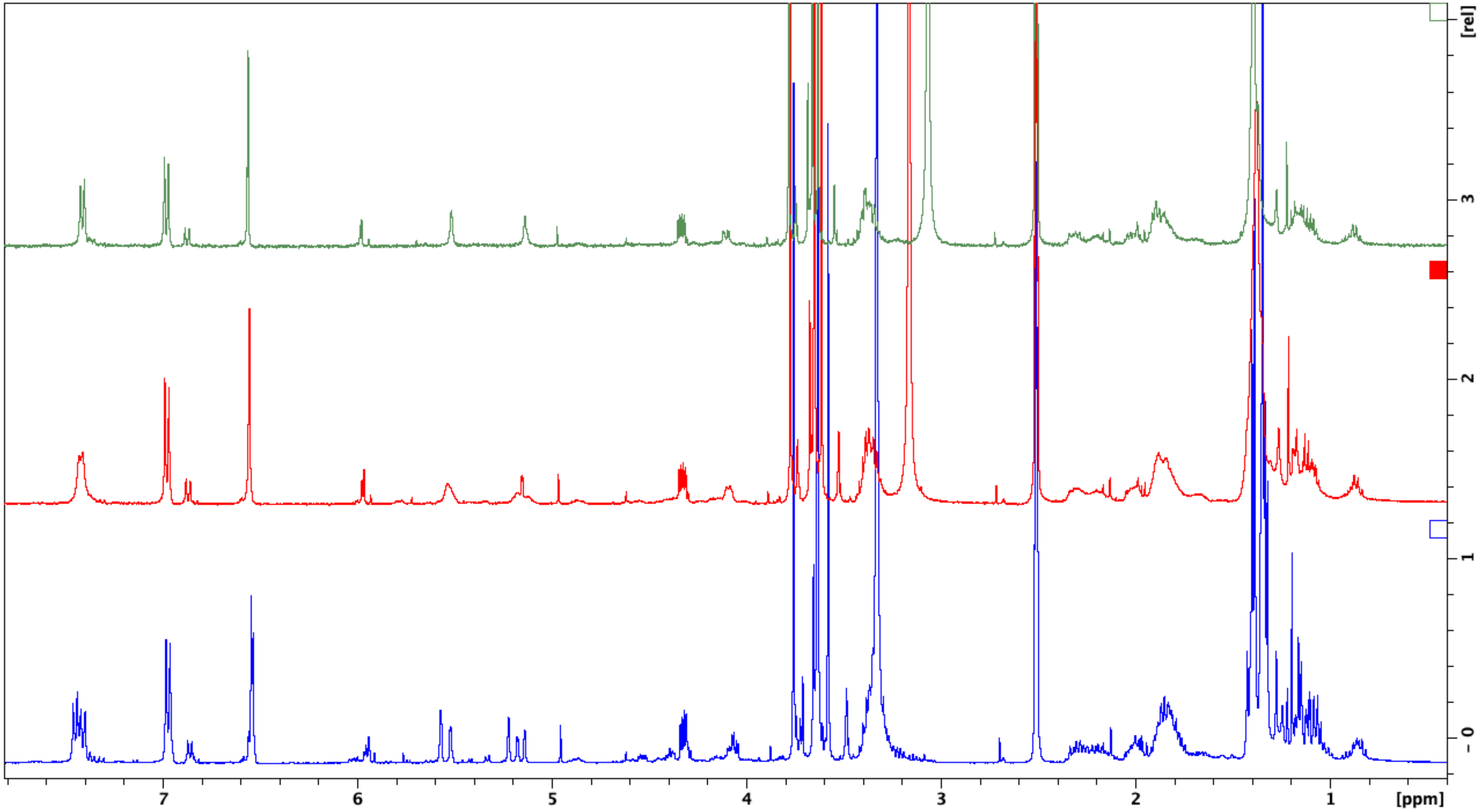

S1.26 VT experiment for diastereomer **2DS2**, DMSO-d_6, 400 MHz. Blue: 25 °C, Red: 60 °C, Green: 80 °C

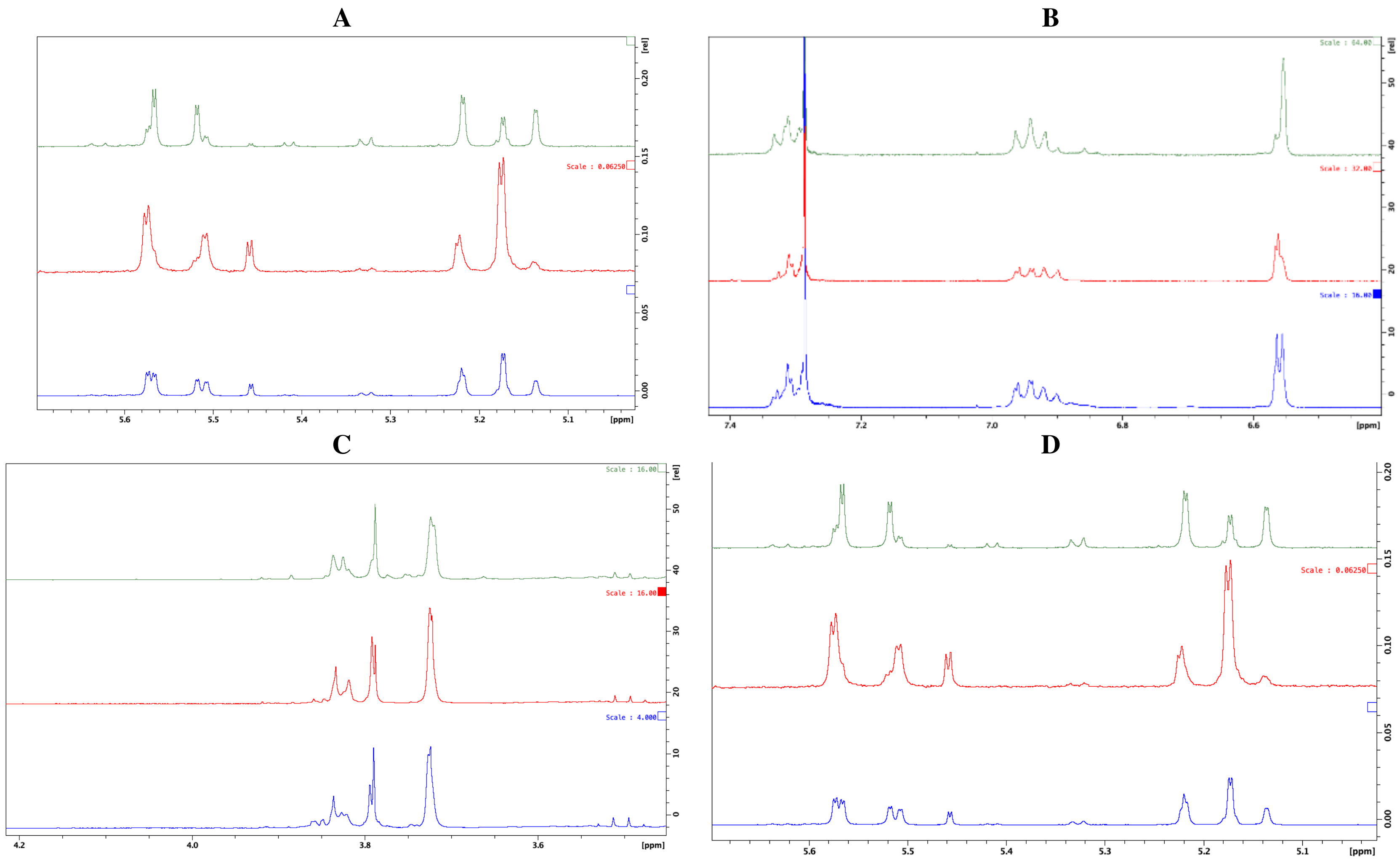

S1.27: [1] H NMR spectra of **2** in $CDCl_3$ at 400 MHz (**2**= blue, **2DS1** = red, **2DS2** = green). **A:** H_3 and H_4 region. **B:** A ring singlet at ~δ 6.5 ppm and B ring doublets at ~ 6.8 – 7.3 ppm **C**: Methoxy singlet region at ~ δ 3.7- 3.9 ppm **D:** [1] H NMR spectra of H_3&H_4 region for *N*-(*BOC*)-D-Proline Diastereomers of **1** in DMSO-d_6 (**2** = blue, **2DS1** = red, **2DS2** = green) at 600 MHz

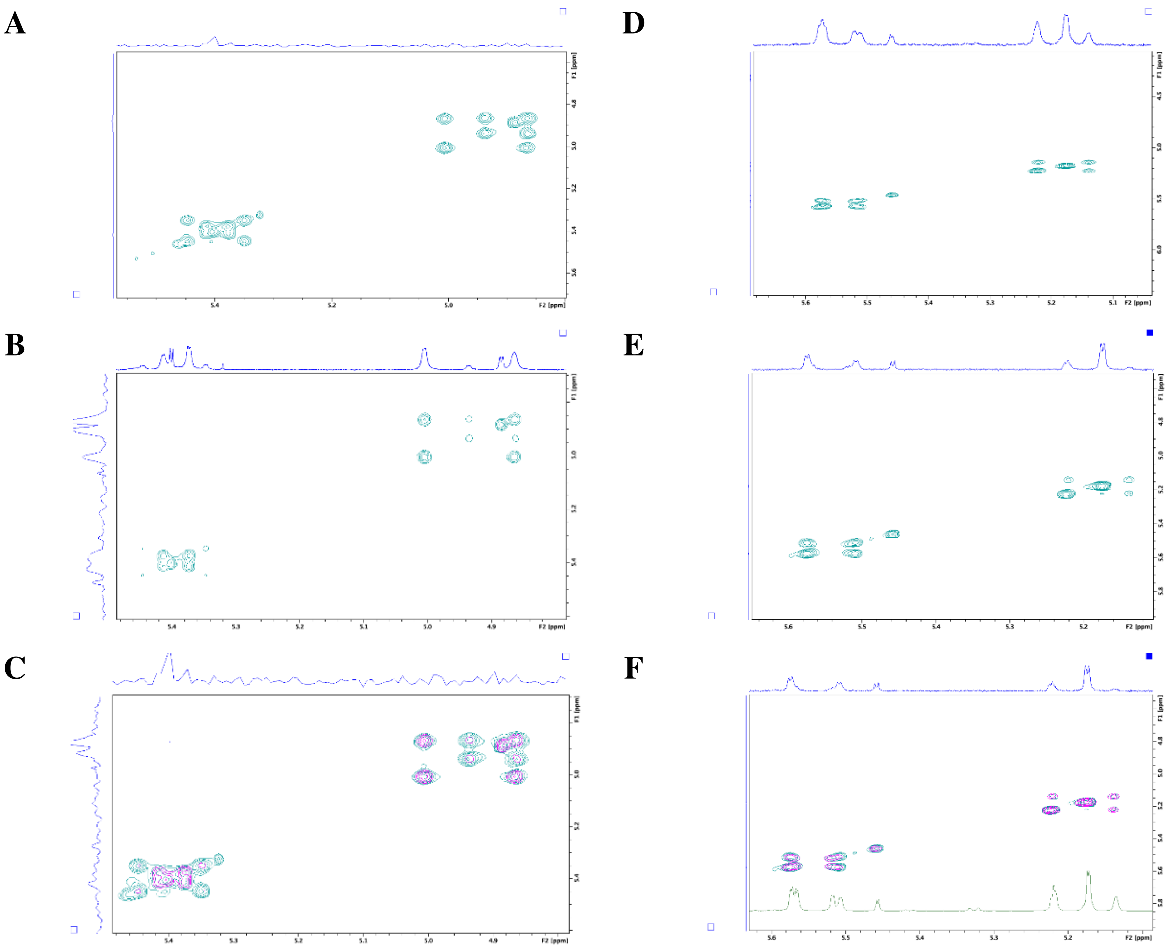

S1.28: 2D EXSY for H_3 and H_4 of **2** at 400 MHz (4.8 – 5.5 ppm).. **A**. Diastereomer mixture of **2** in $CDCl_3$, **B. 2DS1** in $CDCl_3$, **C**: Superimposition of A (green) and B (pink) in $CDCl_3$. **D:** Diastereomer mixture of **2** in DMSO-d_6**. E: 2DS1** in DMSO-d_6 **F:** Superimposition of C (green) and D (pink) in DMSO-d_6

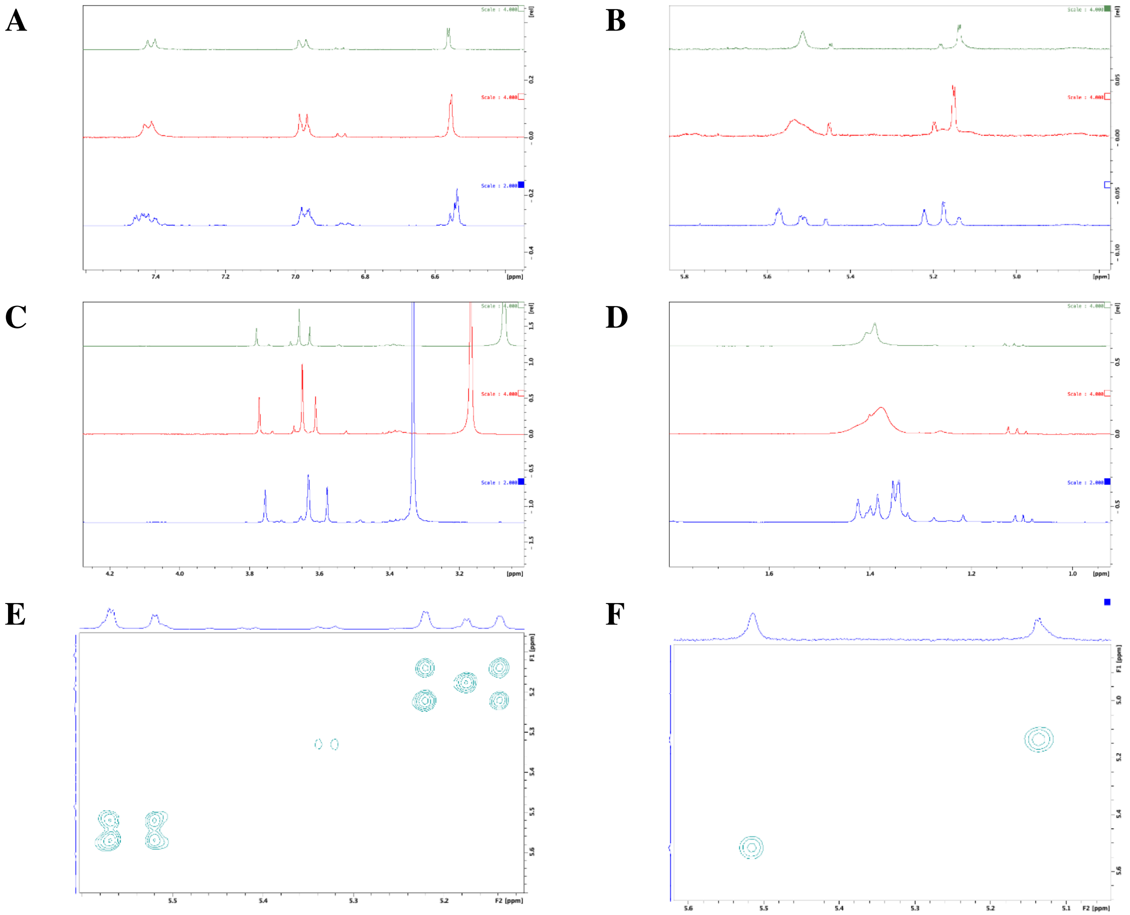

S1.29: VT experiment for **2** in DMSO-d_6 at 400 MHz **A:** B ring region with A ring singlet at δ 6.68 ppm. **B**: H_3 and H_4 region. **C**: A and B ring methoxy singlet region. **D:** *t*-butoxy region. Blue: 25 °C Red: 60 °C Green: 80 °C **E:** 2D NOESY for **2DS2** at 25 °C F: 2D NOESY for **2DS2** at 85 °C

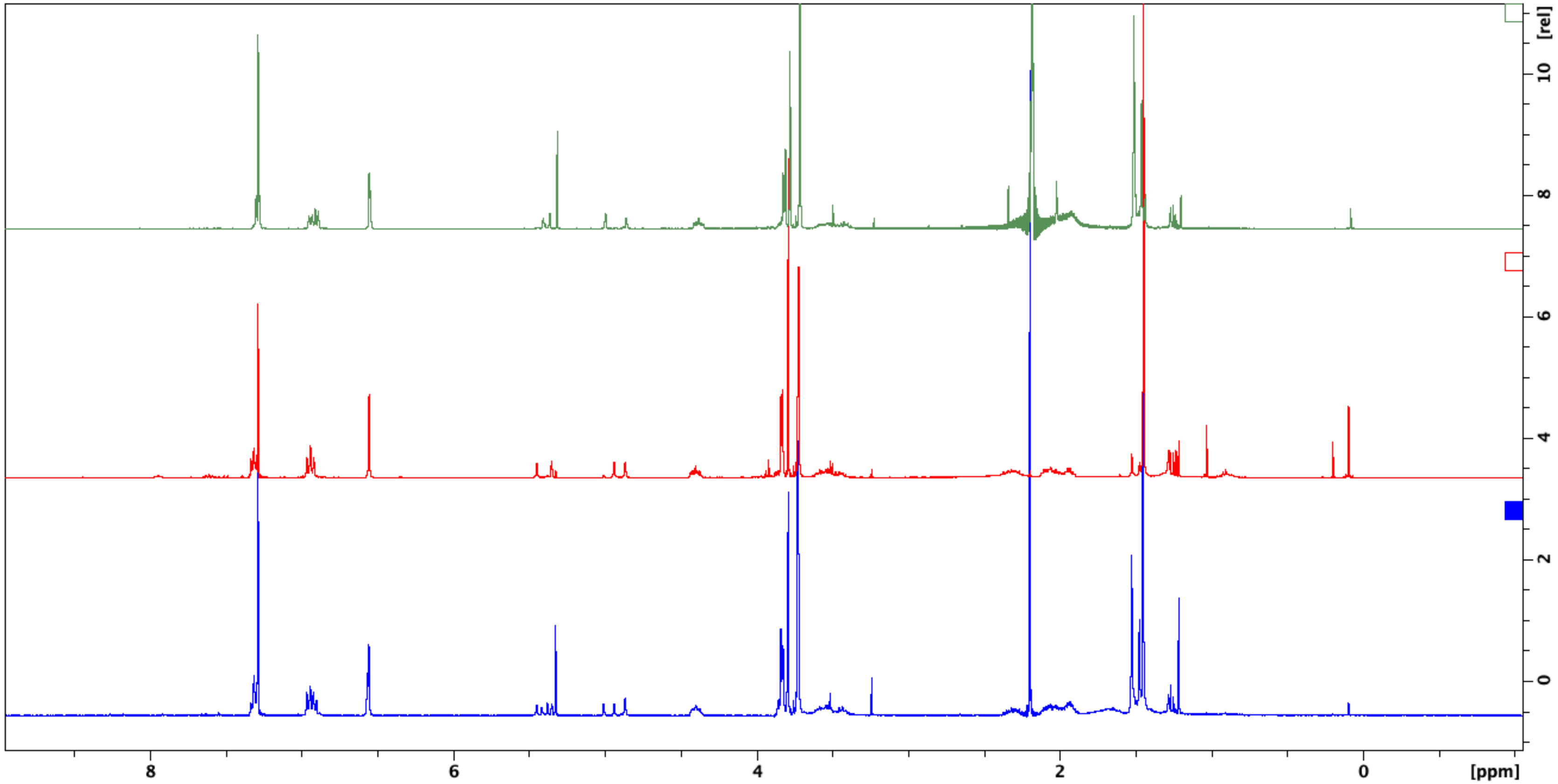

S1.30 ^{1}H NMR of **3** (blue), **3DS2** (middle, red), **3DS1** (top, green) at 400 MHz, $CDCl_3$

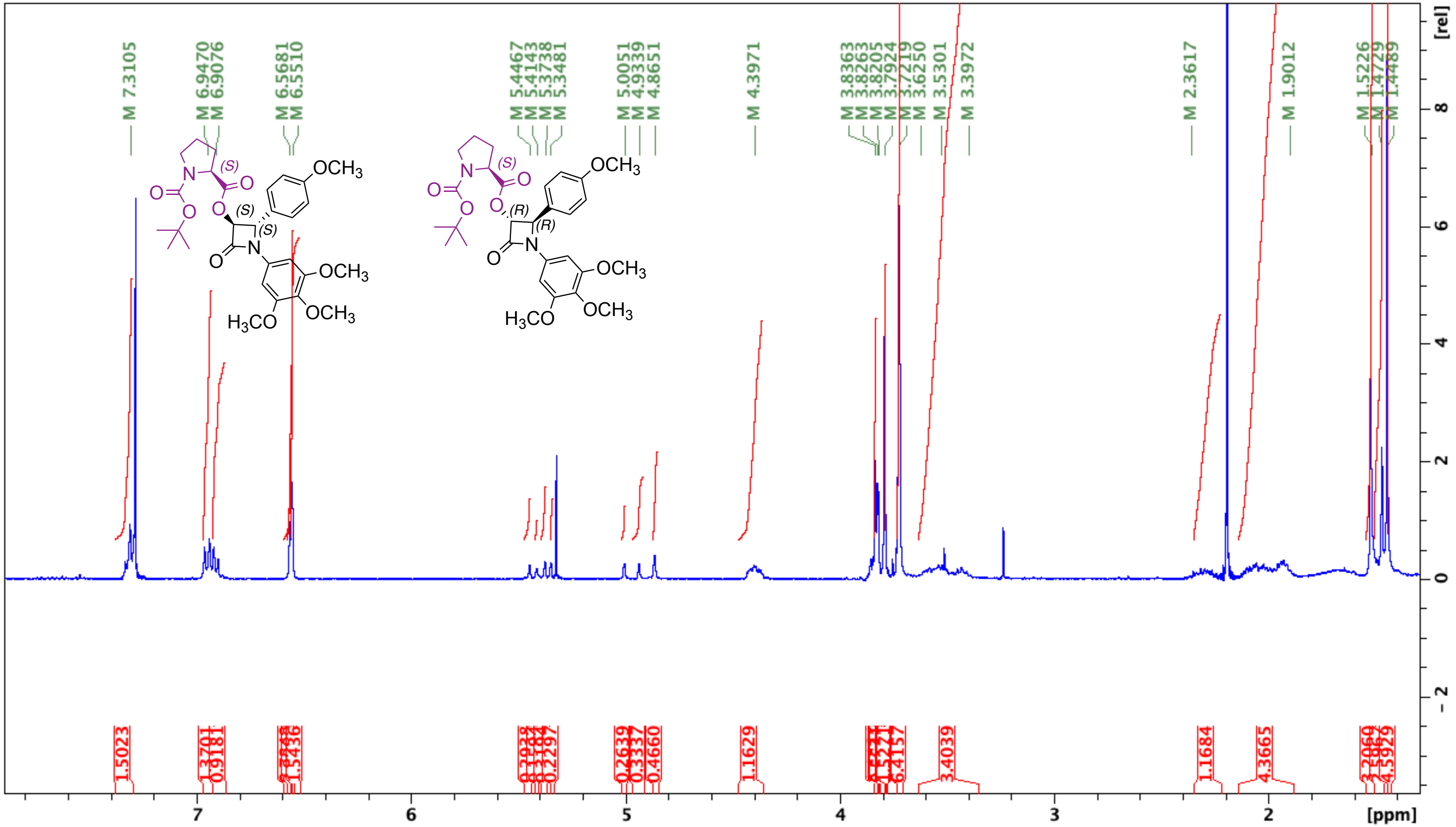

S1.31 ^{1}H NMR for **3** at 400 MHz, $CDCl_3$

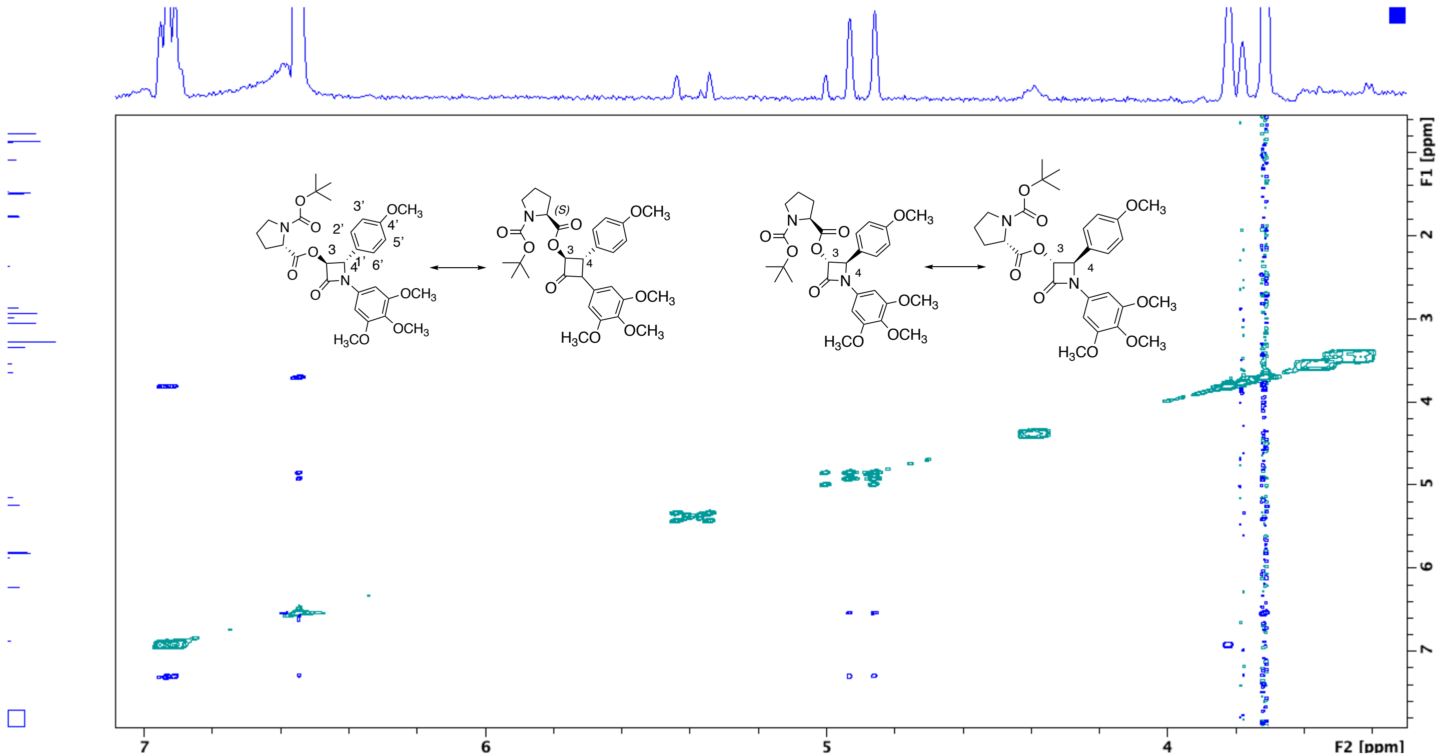

S1.32 2D EXSY spectrum for **3** at 400 MHz, $CDCl_3$. Rotamers only observed in H_3 and H_4 region from 4.9-5.5 ppm.

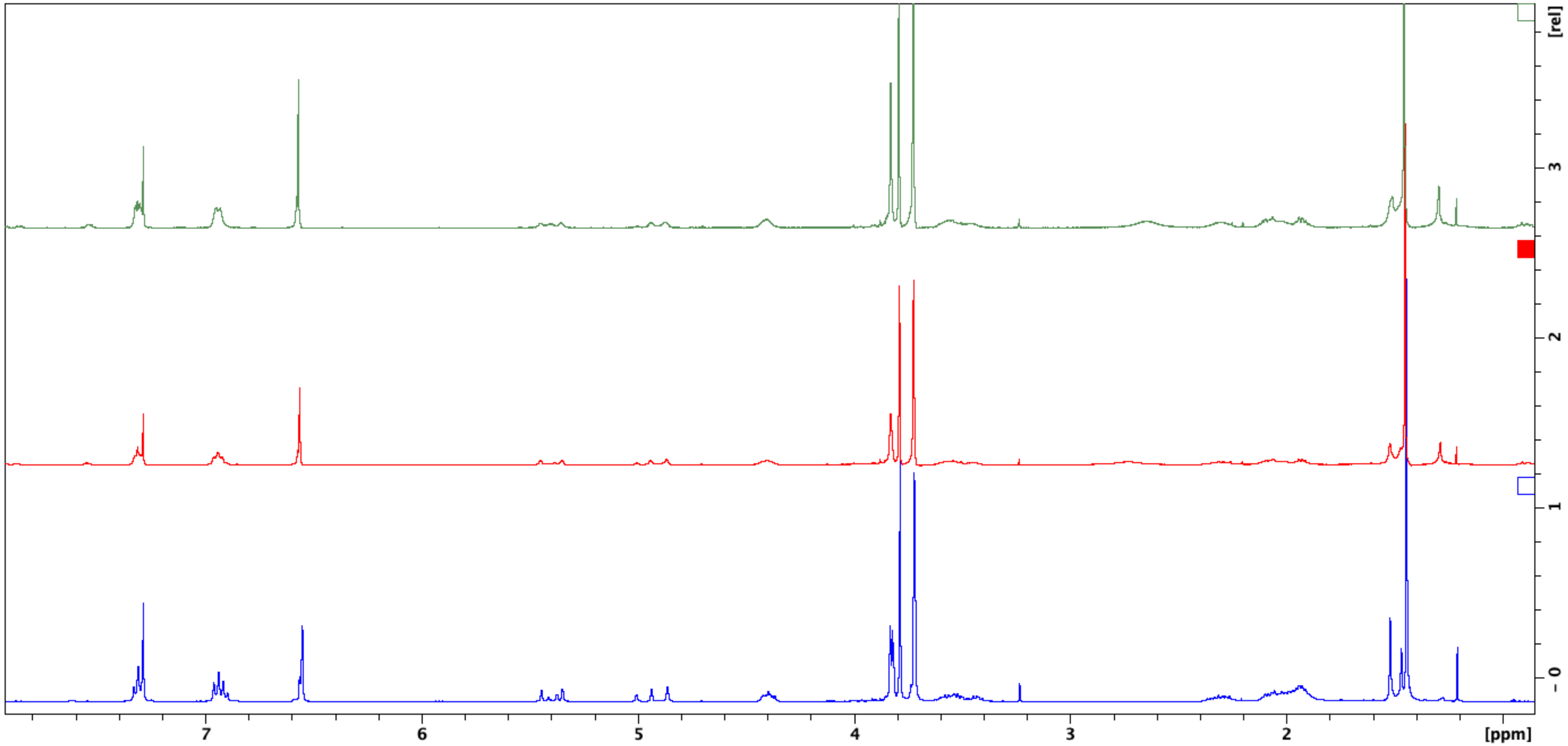

S1.33 VT Experiment for **3** in $CDCl_3$ at 400 MHz. Blue: 25 °C, red: 40°C, green: 50 °C.

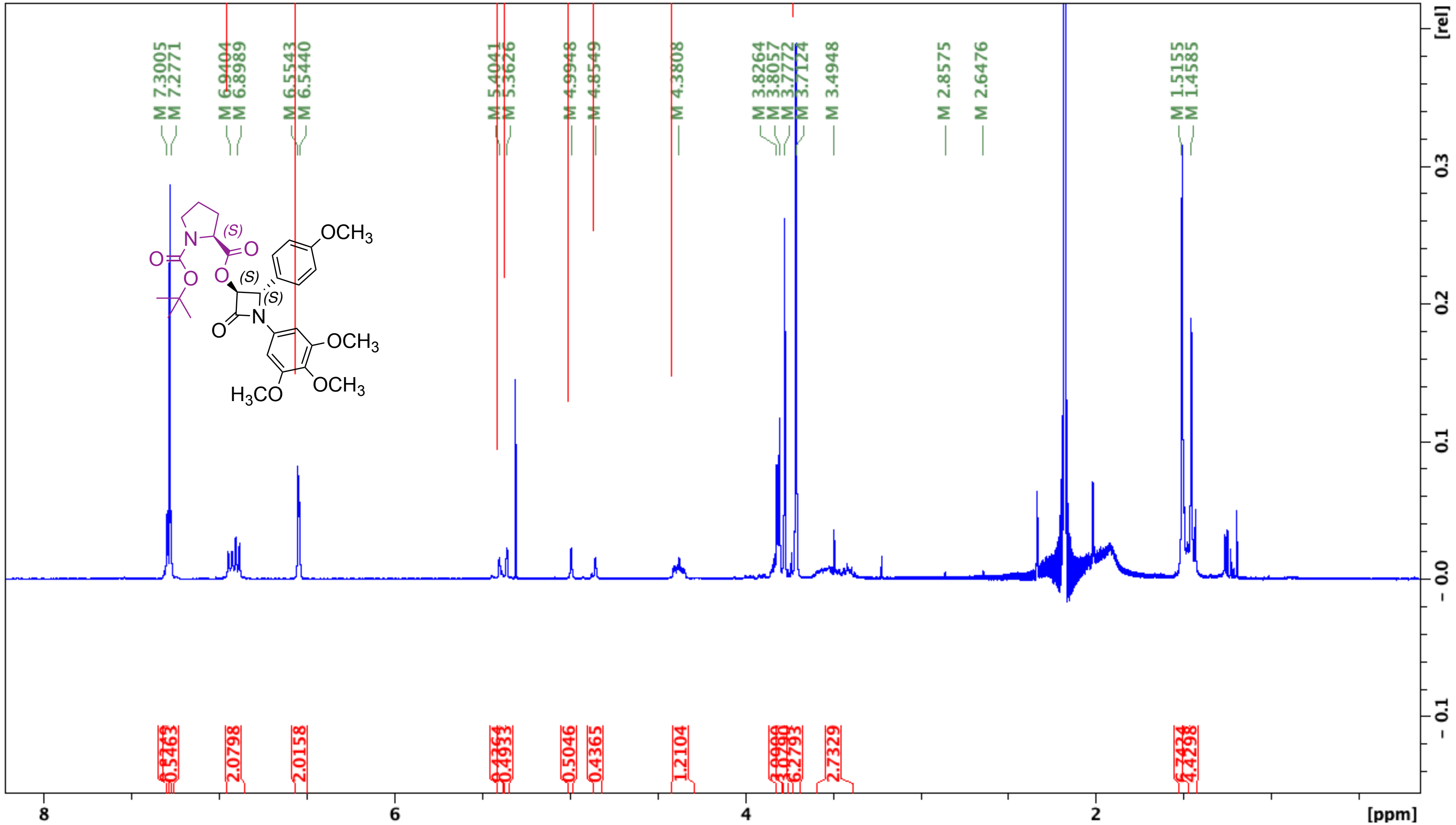

S1.34 ¹H NMR for diastereomer **3DS1** at 400 MHz, $CDCl_3$

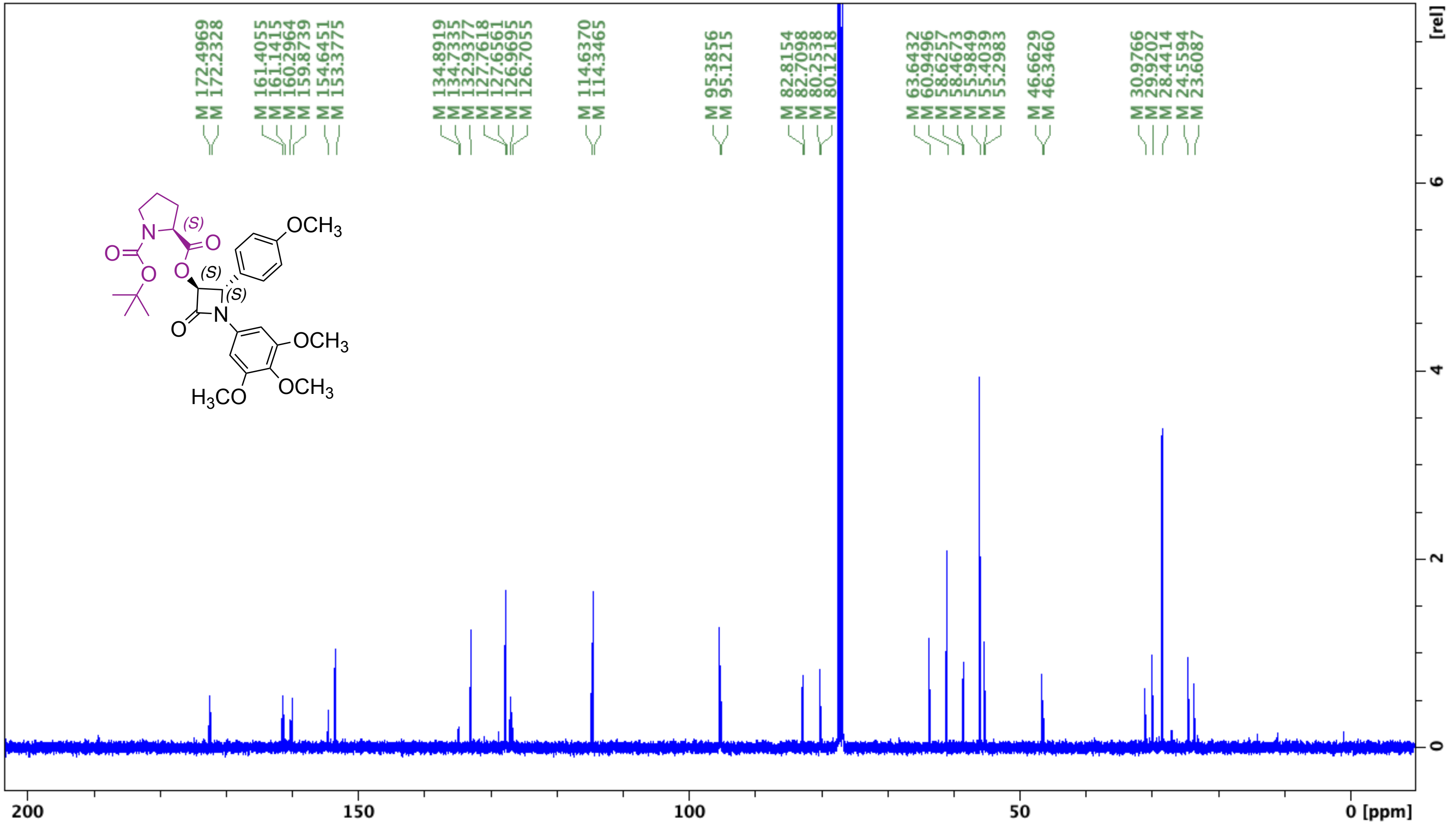

S1.35 ^{13}C NMR for diastereomer **3DS1** at 100 MHz, $CDCl_3$

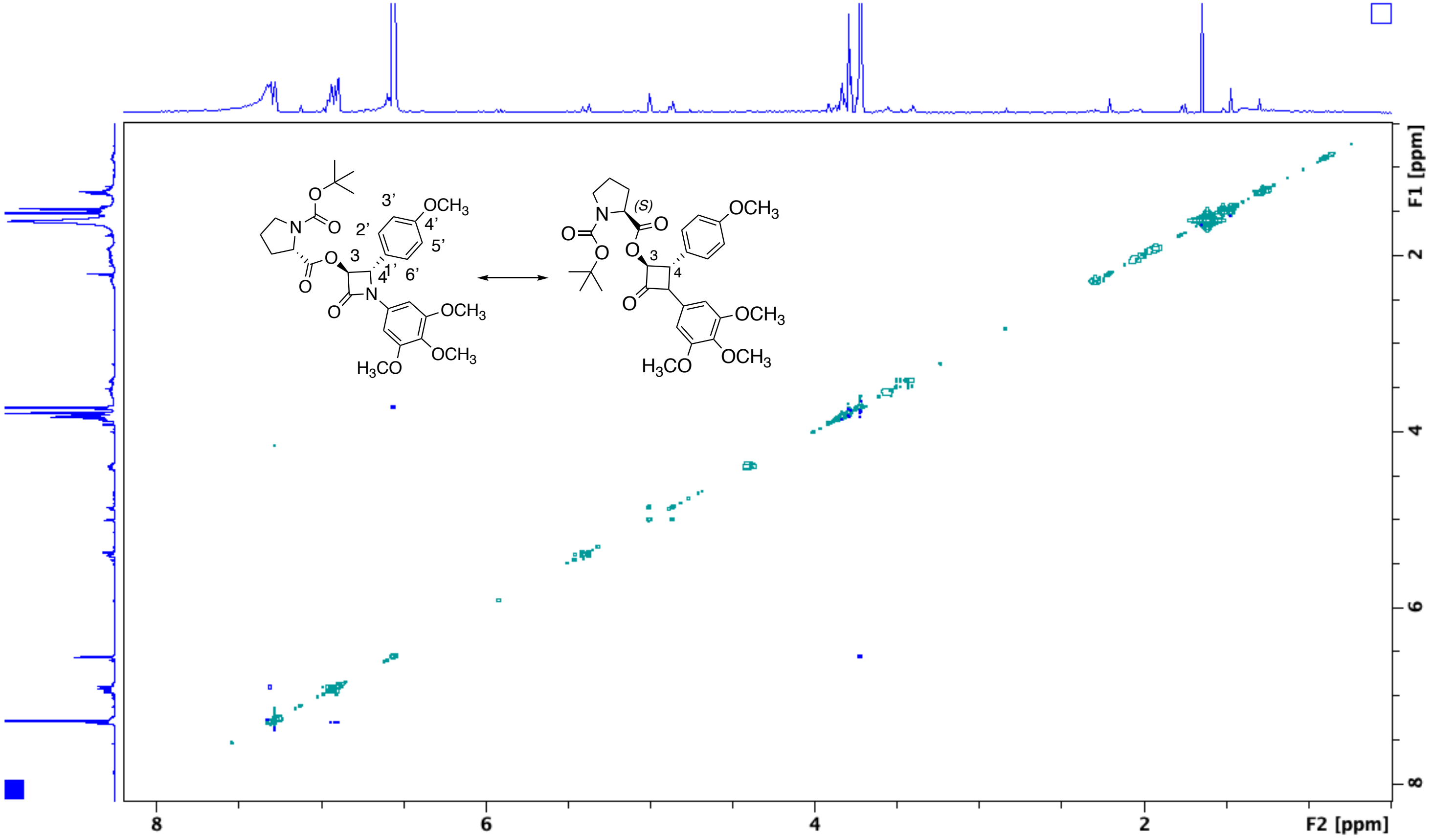

S1.36 2D EXSY spectrum for **3DS1** in $CDCl_3$, 400 MHz. Rotamers observed in H_3 and H_4 region from 4.9-5.5 ppm. Rotamers also observed in $H_{3'\&5'}$ region at 6.8-7 ppm.

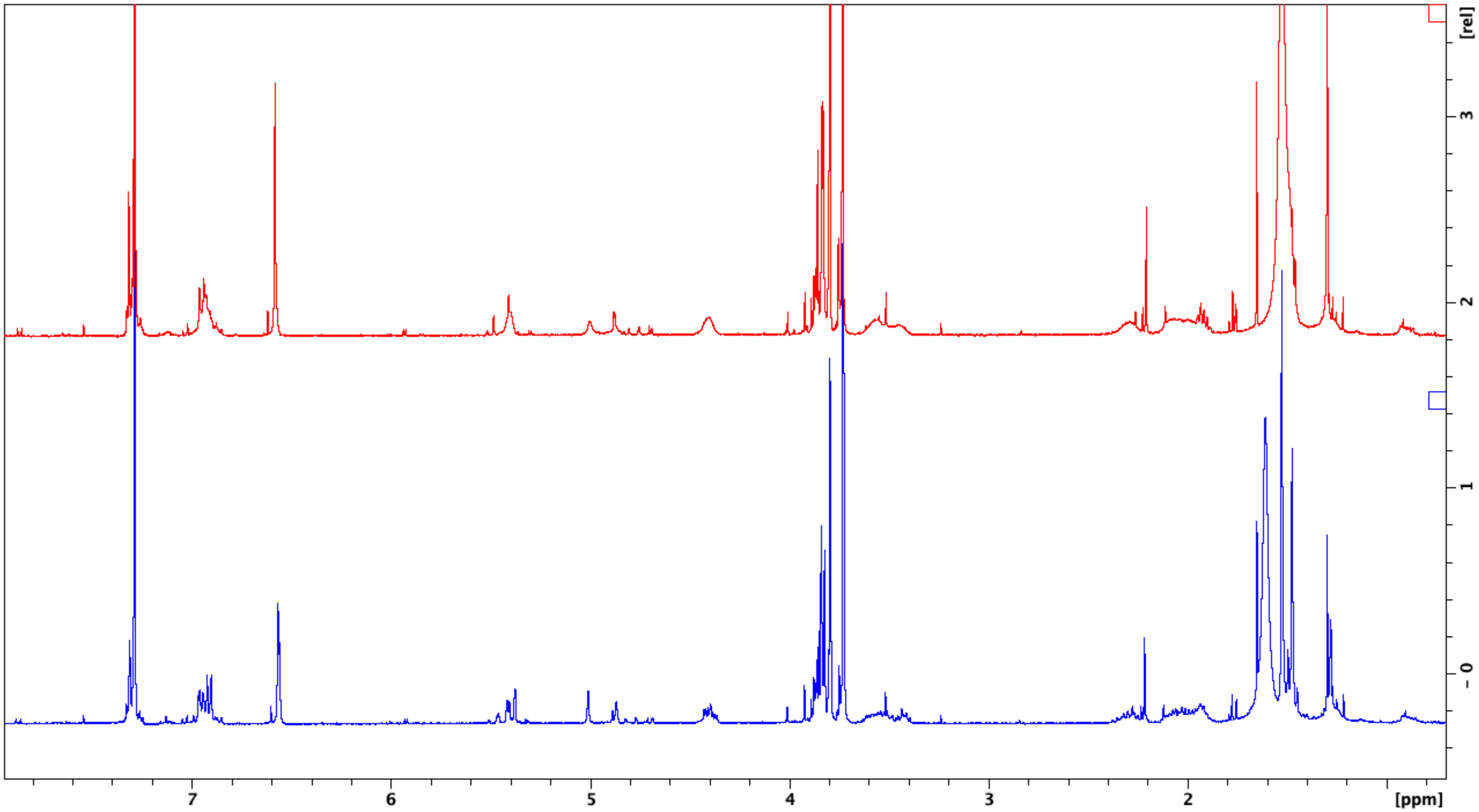

S1.37 VT Experiment for **3DS1** in $CDCl_3$ at 400 MHz. Blue: 25 °C, red: 40°C.

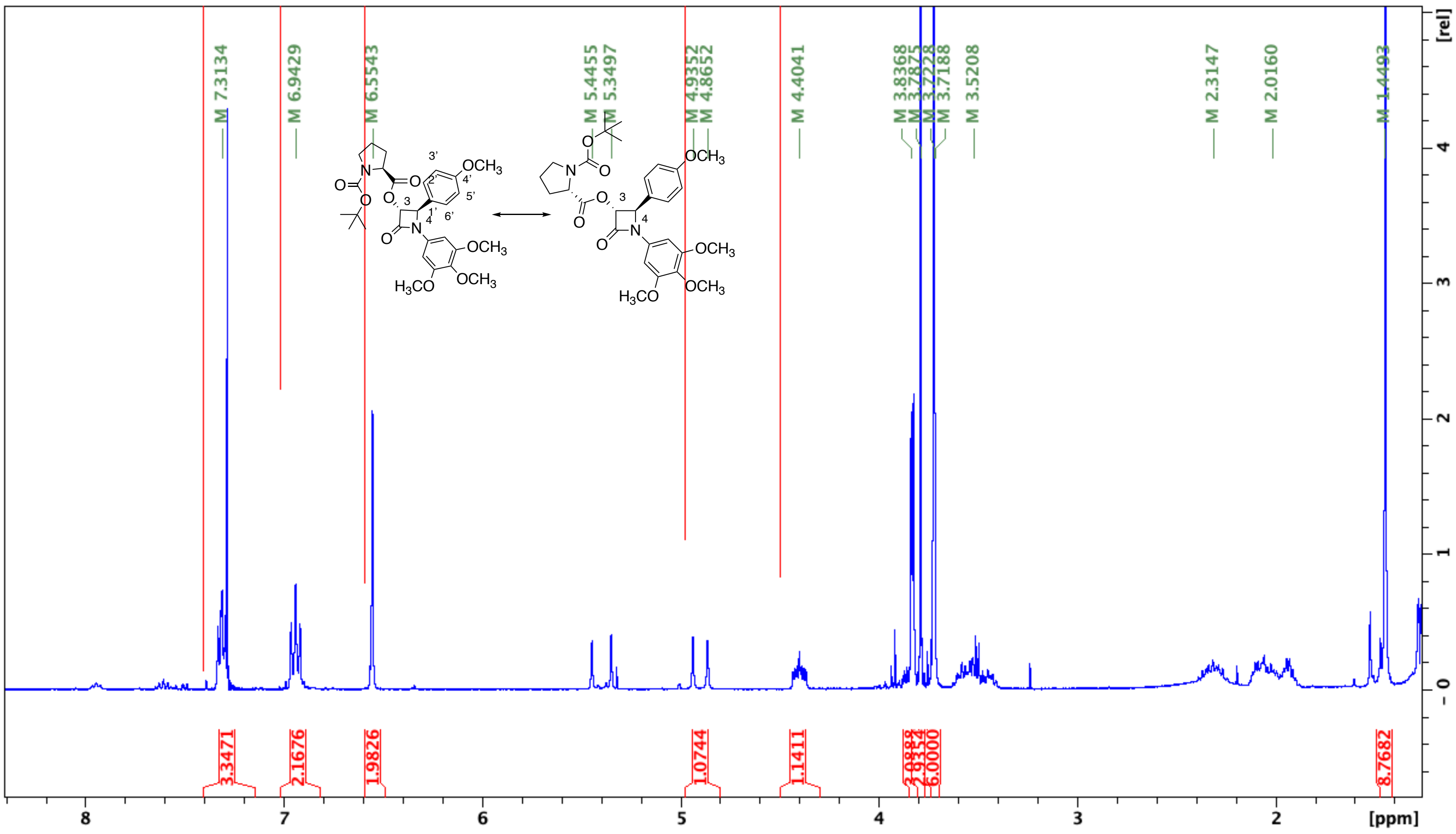

S1.37 ^{1}H NMR for diastereomer **3DS2** at 400 MHz, $CDCl_3$. Rotamers observed for H_3 and H_4 at 4.9-5.5 ppm. Rotamers also observed for the $H_{2'}$ and $H_{6'}$ doublet at 7.31 ppm and $H_{3'}$ and $H_{5'}$ doublet at 6.94 ppm.

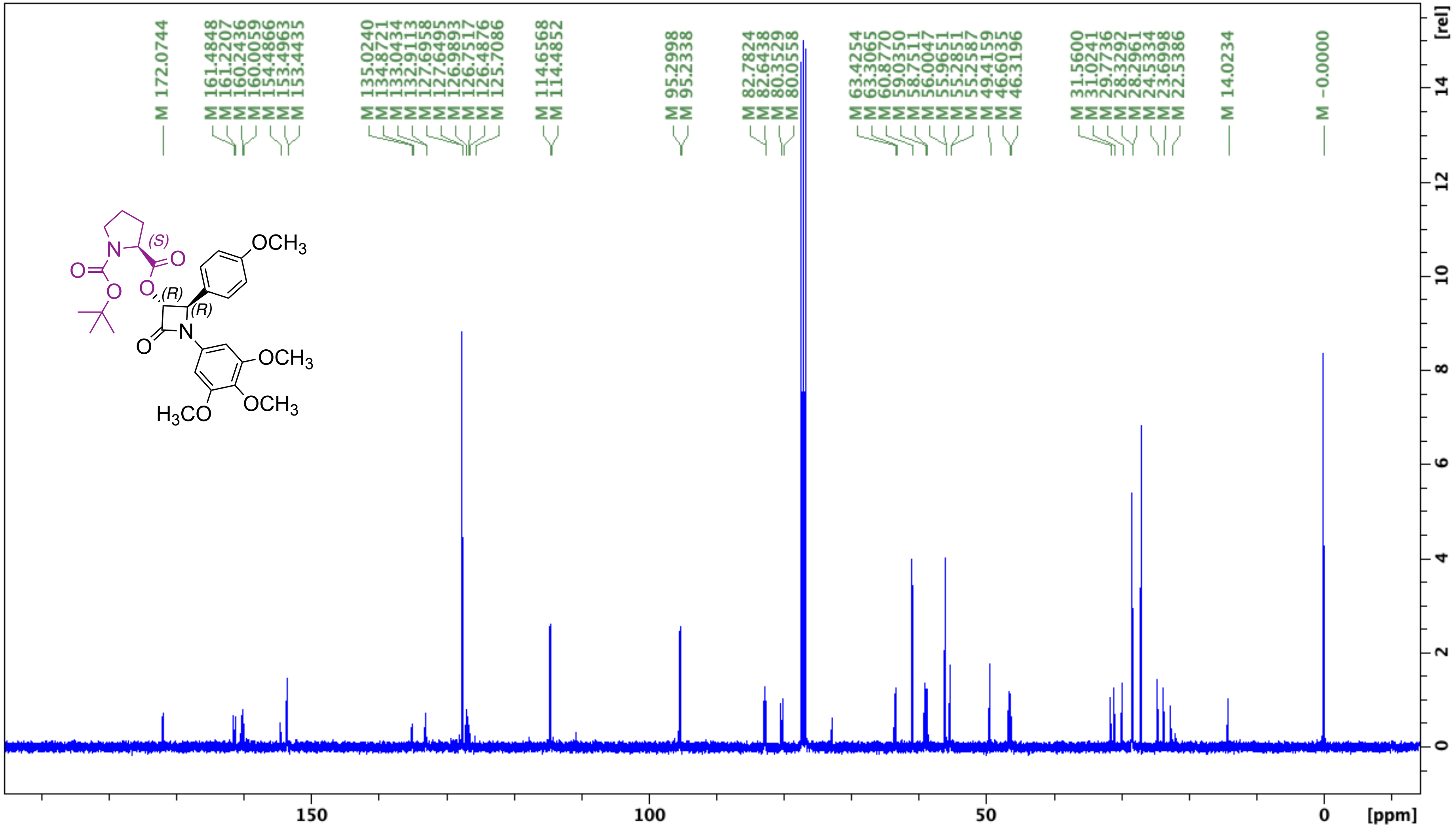

S1.38 ^{13}C NMR for diastereomer **3DS2,** 100 MHz, $CDCl_3$

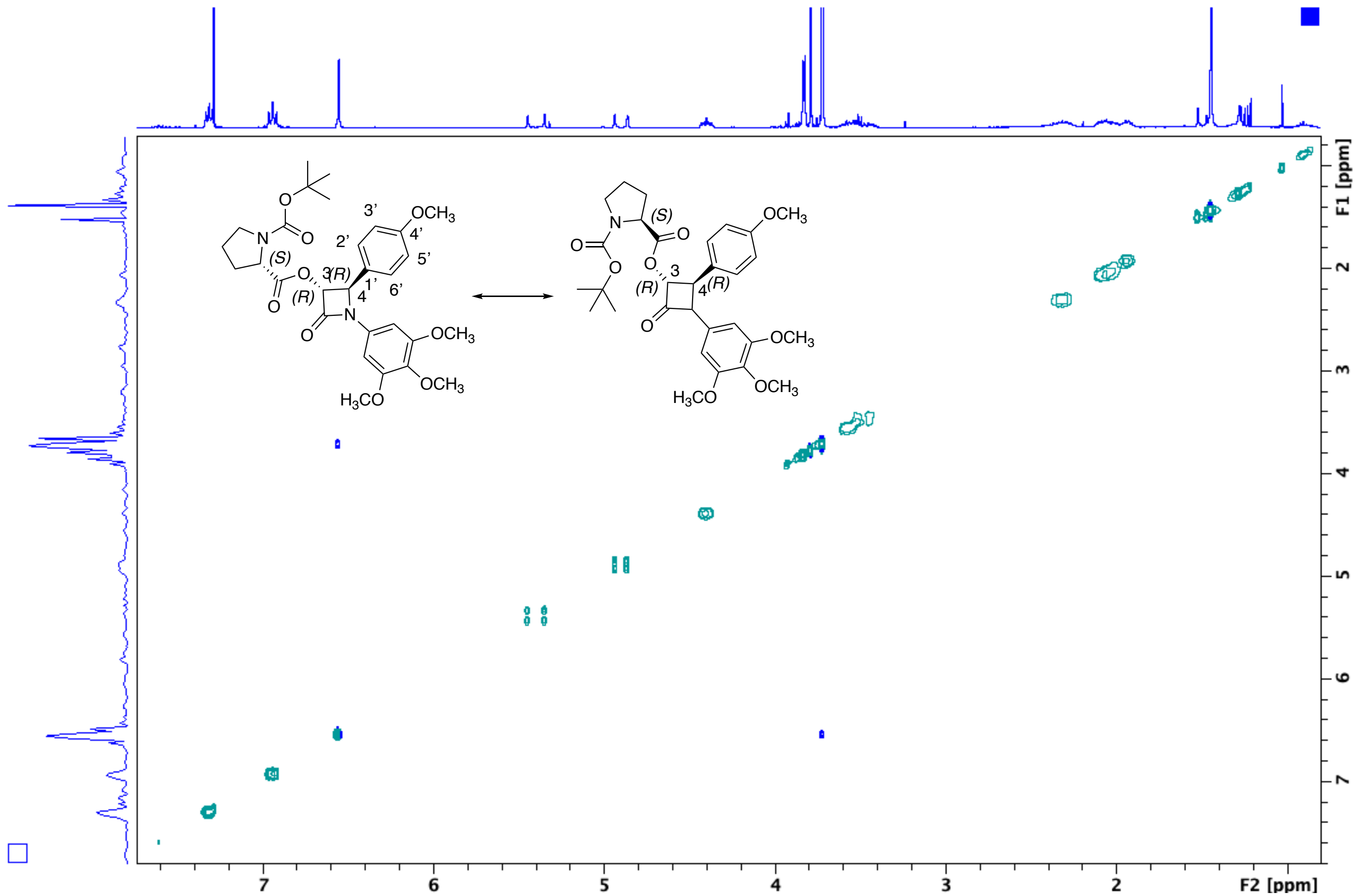

S1.39 2D EXSY spectrum for **3DS2** in $CDCl_3$,400 MHz. Rotamers for **3DS2** illustrated. Rotamers observed for H_3 and H_4 as cross peaks at 4.5-5.5 ppm.

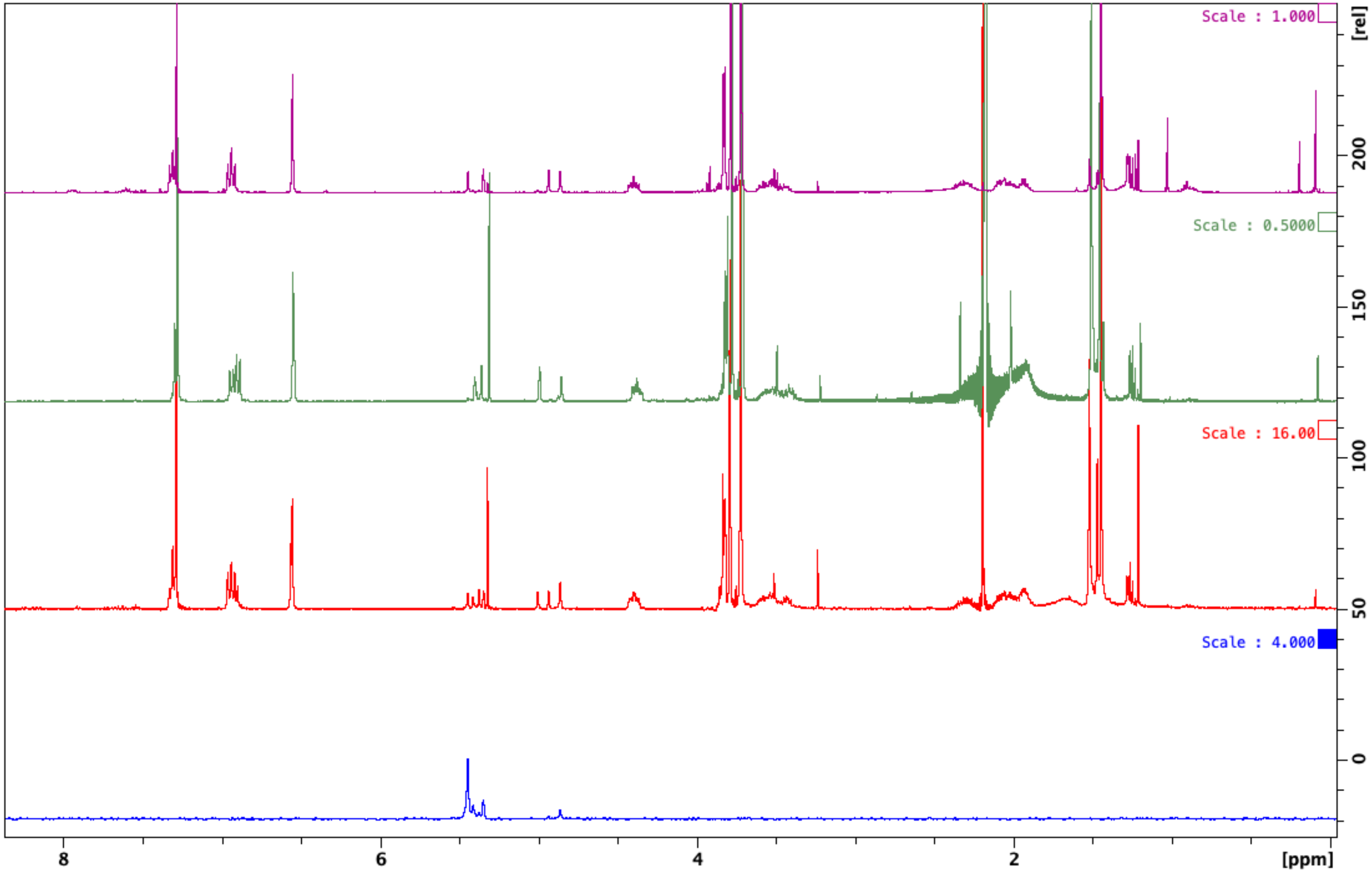

S1.40 Selective TOCSY of **3;** diastereomer mixture **3** at 400 MHz, $CDCl_3$ (blue) with spin lock from 5.45 ppm. Full ^1H NMR of **3**; diastereomer mixture (red), **3DS1** (green), **3DS2** (purple) shown as a reference.

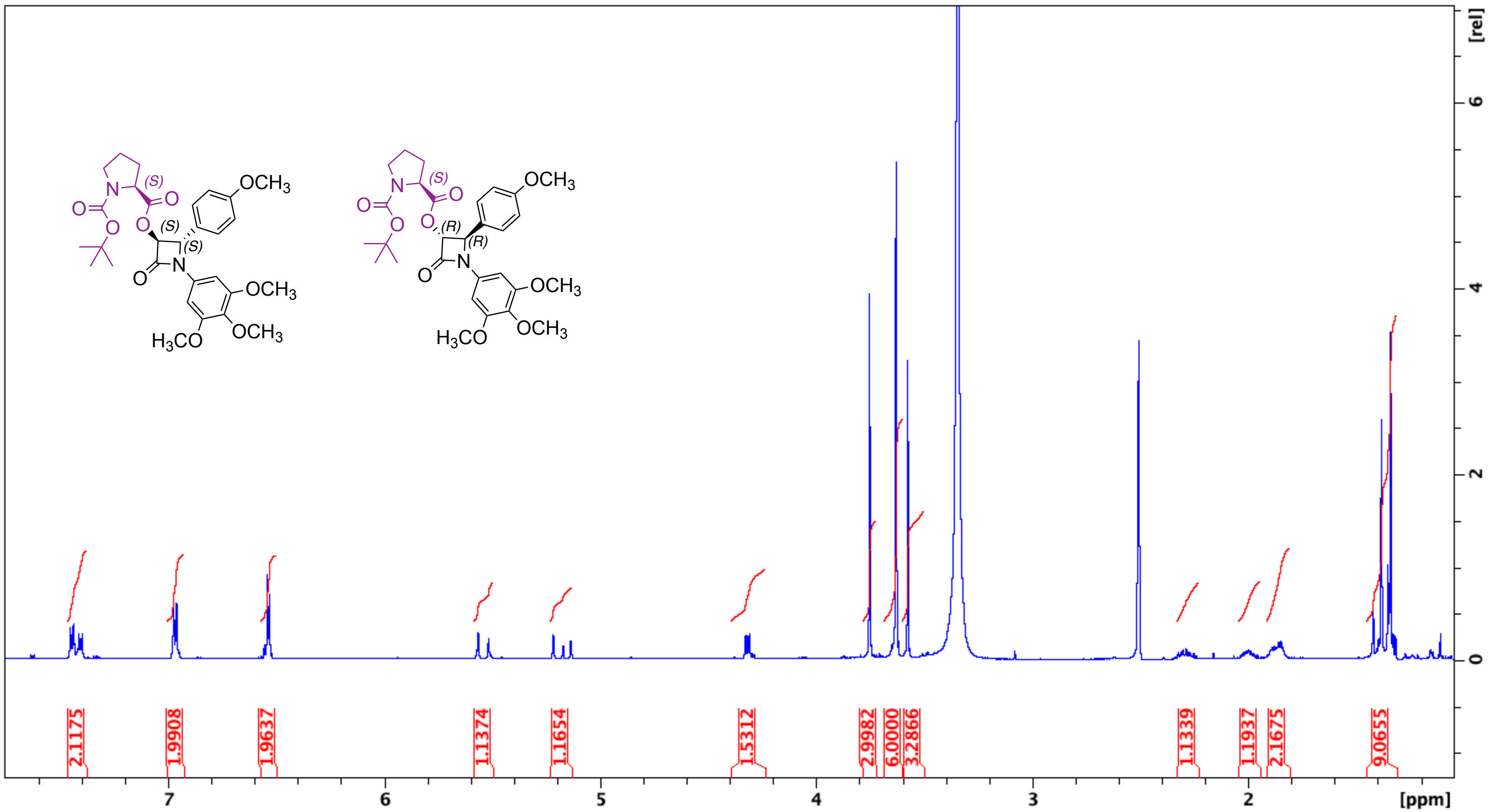

S1.41 ^{1}H NMR for **3** at 400 MHz, DMSO-d_6. **Purity RP-HPLC:** 95%

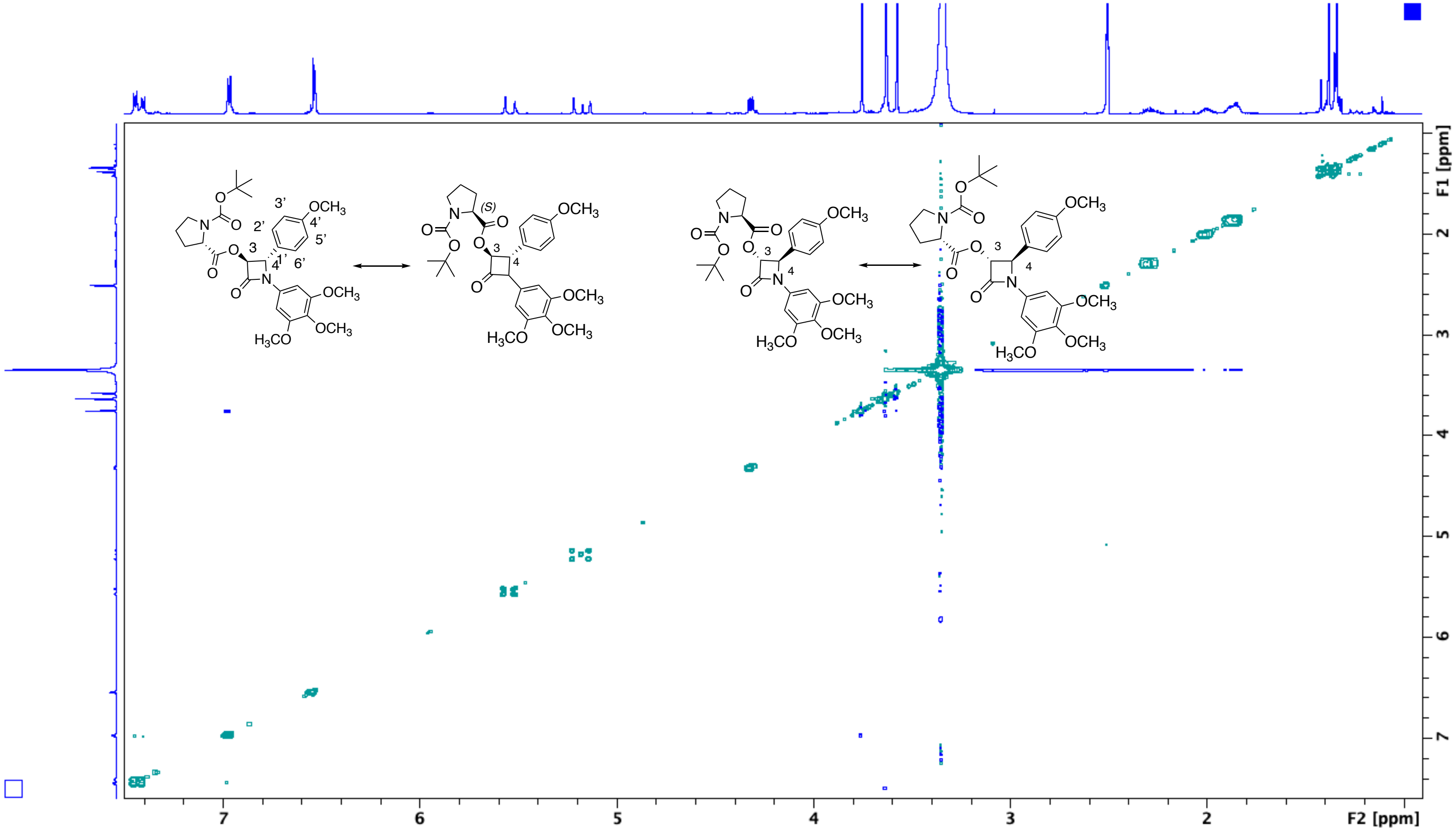

S1.42 2D EXSY spectrum for **3** at 100 MHz, DMSO- d_6. Rotamers observed in H_3 and H_4 region.

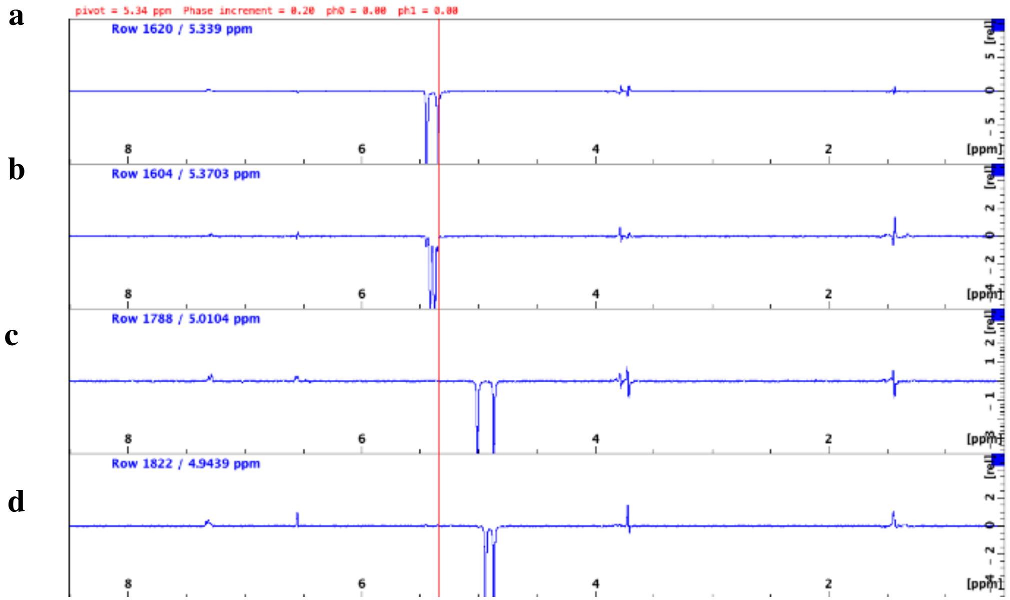

S1.43: Manual phasing of 2D EXSY spectra in $CDCl_3$ for **3** illustrating same phase exchange signals **a: 3DS2** (H_3) **b**: **3DS1** (H_3) **c: 3DS1** (H_4) and **d: 3DS2** (H_4).

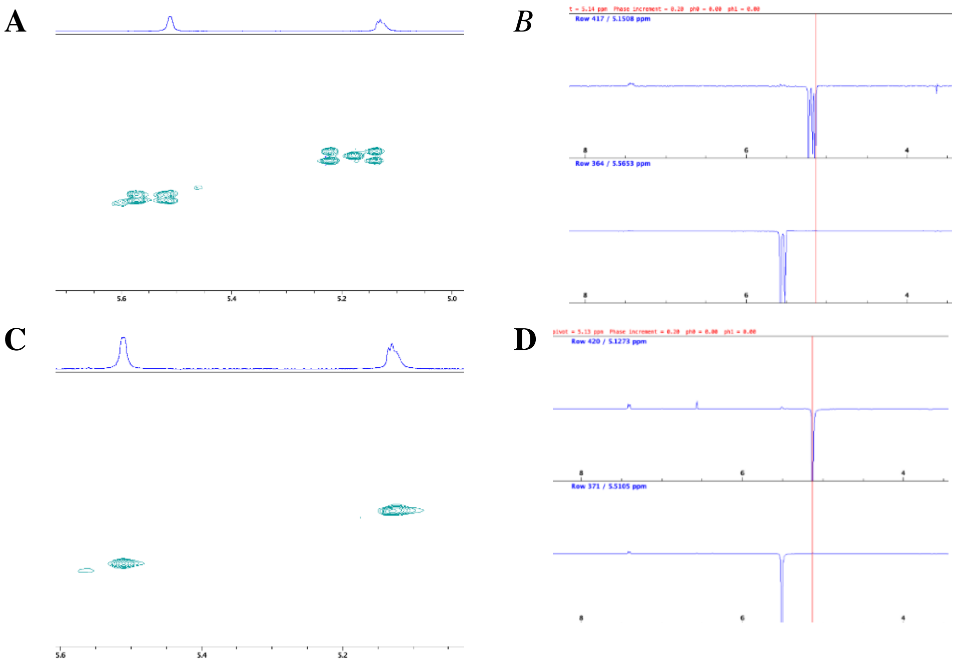

S1.44: 2D EXSY of **3** for H_3 and H_4 region in DMSO-d_6 at 400 MHz at **A** : 25 °C **B:** Manual phasing of H_3 and H_4 at 25 °C **C:** 90 °C **D:** Manual phasing of H_3 and H_4 at 90°C

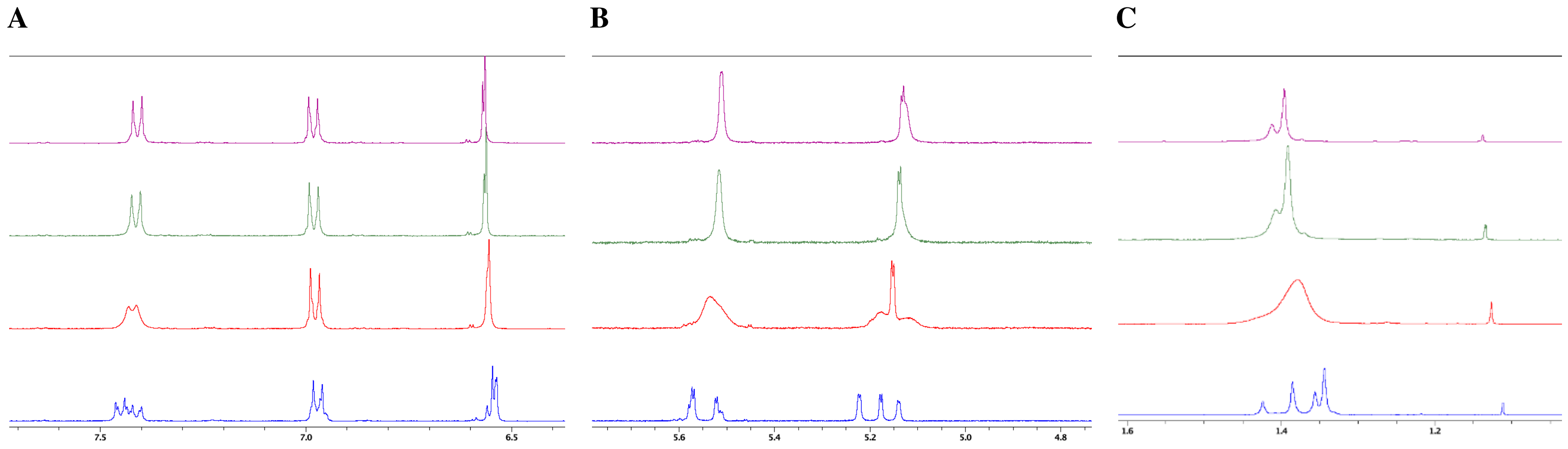

S1.45: VT Experiment for **3** in DMSO-d_6 at 400 MHz. Enlargements of ^{1}H NMR spectrum of **3** **A:** B ring region. **B:** H_3 and H_4 region. **C**: *t*-butoxy region. Blue: 25 °C, red: 60°C, green: 80 °C, purple: 90°C.

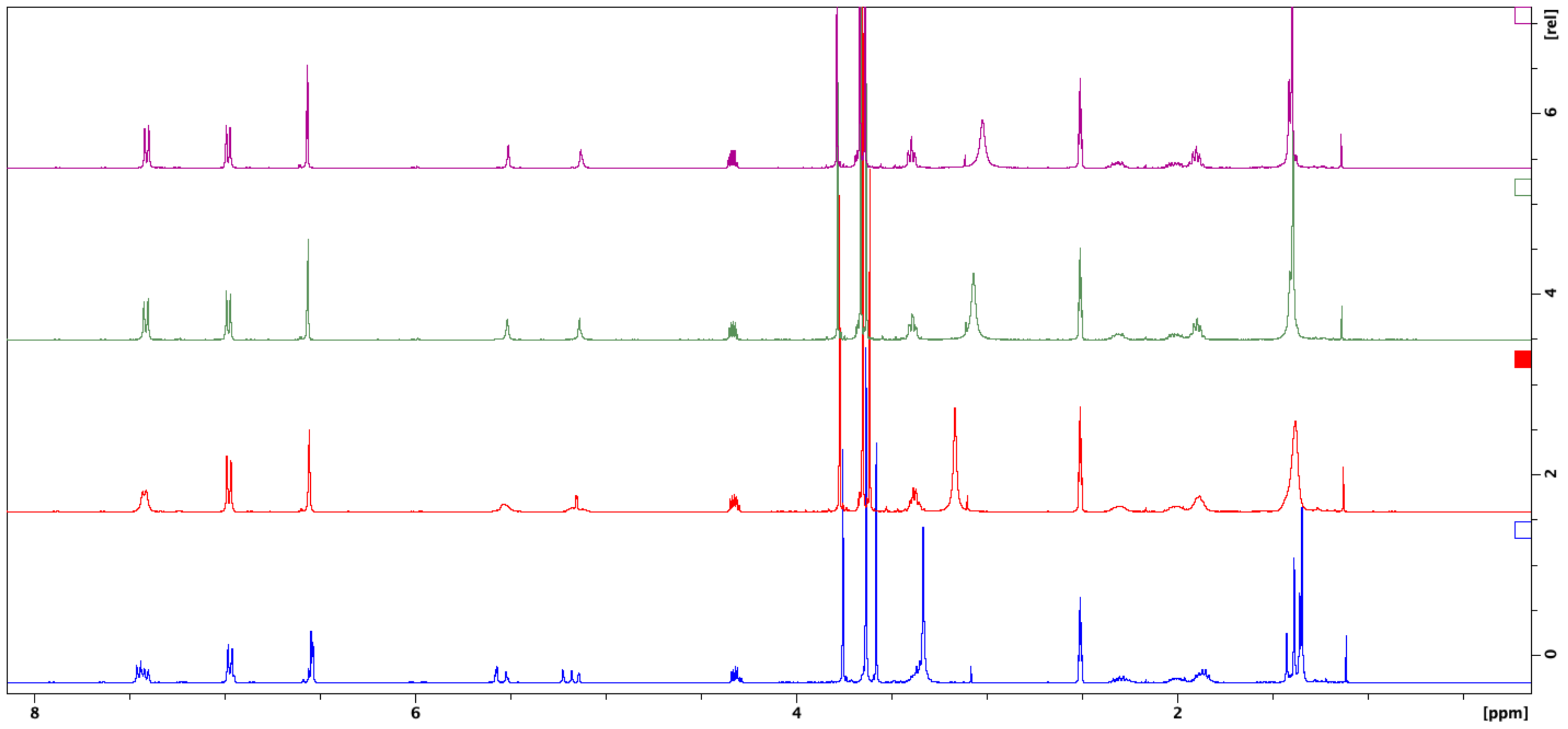

S1.46: VT experiment for **3** at 100 MHz in DMSO-d_6. Blue: 25 °C, red: 60°C, green: 80 °C, purple: 90°C.

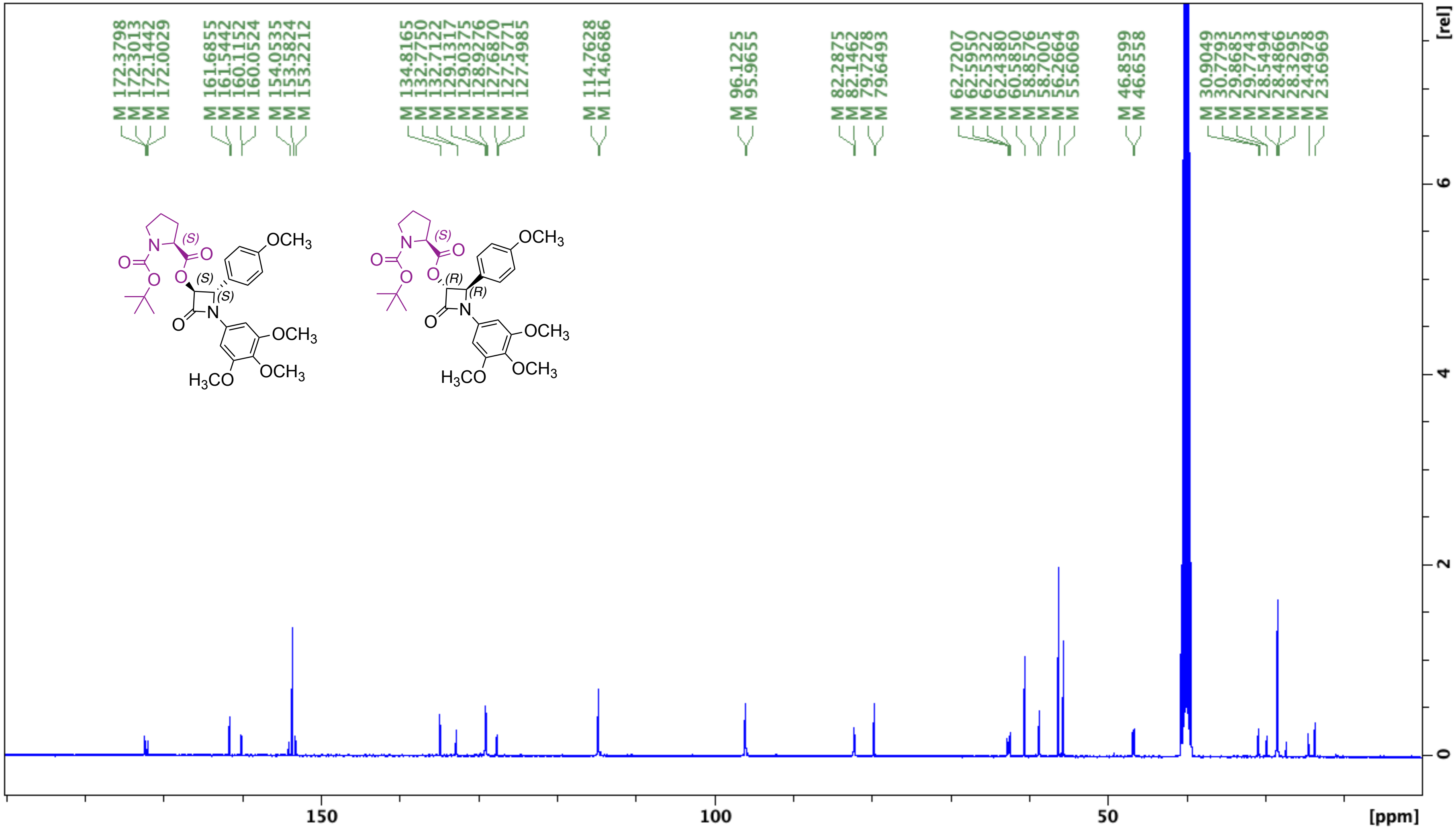

S1.47: ^{13} C NMR for **3** at 100 MHz, DMSO-d_6, 100 MHz. **Purity RP-HPLC:** 99%

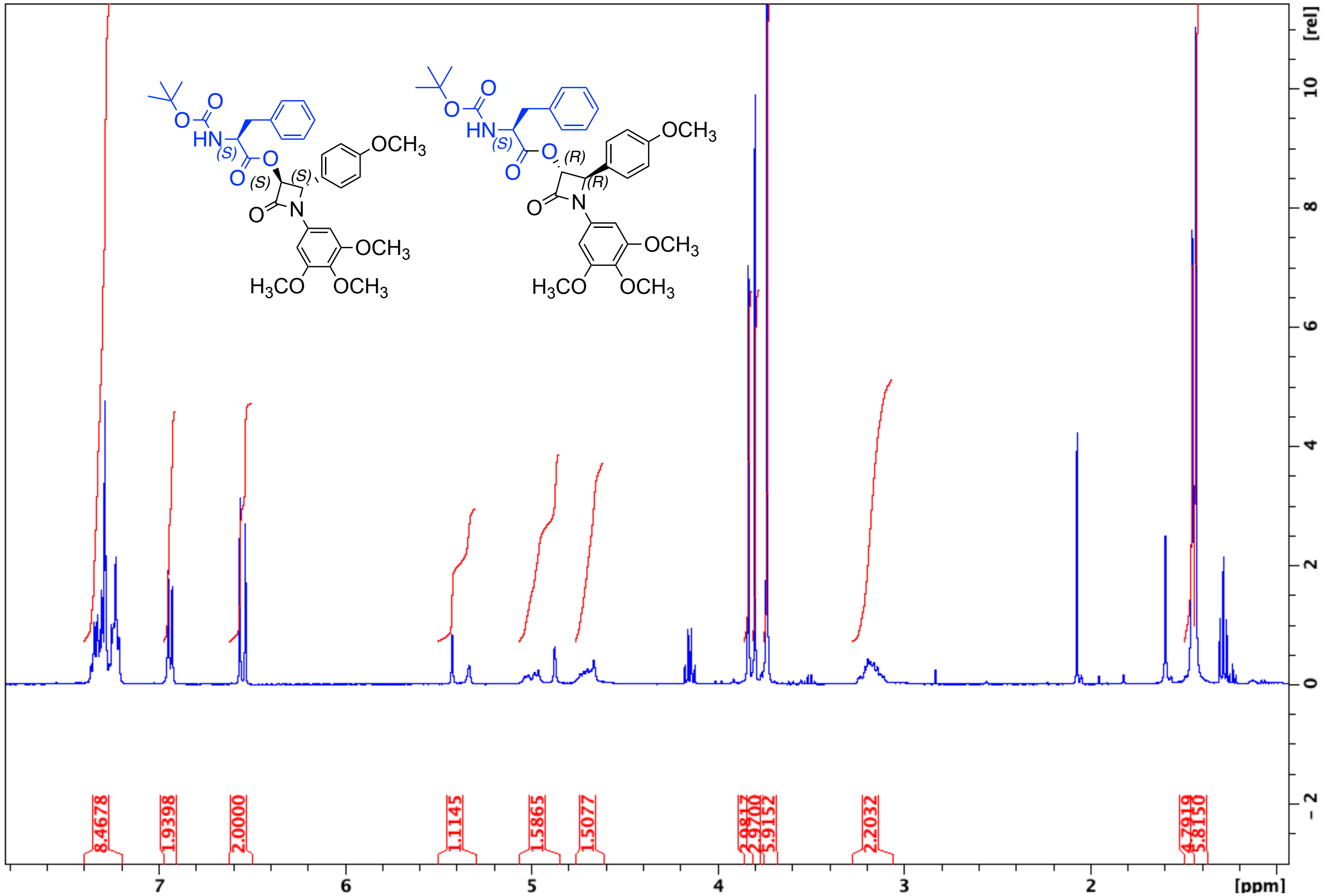

S1.48: ¹H NMR of **4**, $CDCl_3$, 400 MHz. **Purity RP-HPLC:** 99%

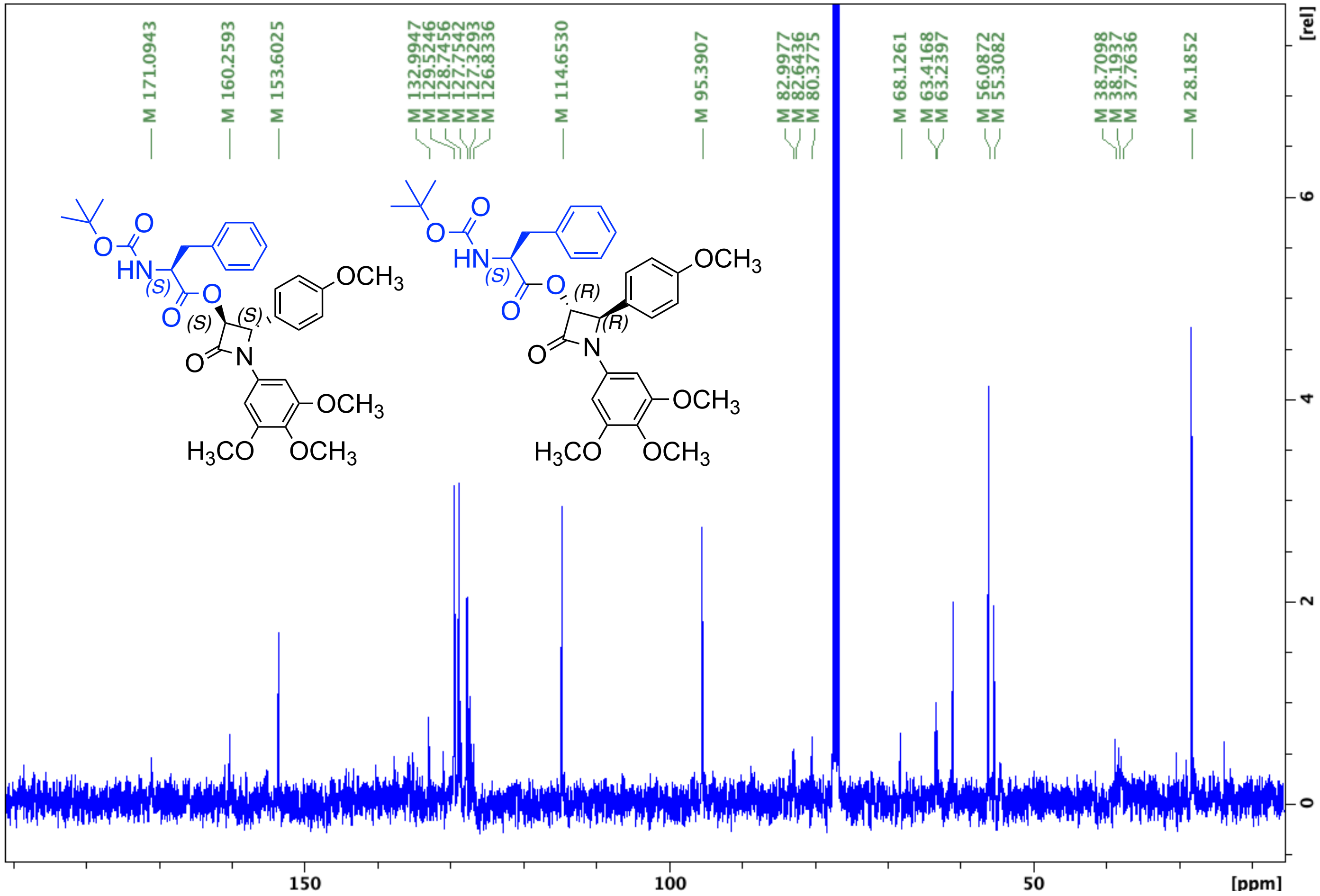

S1.49: [13] C NMR 2-(4-methoxyphenyl)-4-oxo-1-(3,4,5-trimethoxyphenyl)azetidin-3-yl (*tert*-butoxycarbonyl)-*L*-phenylalaninate, **4,** $CDCl_3$, 100 MHz. **Purity RP-HPLC:** 99%

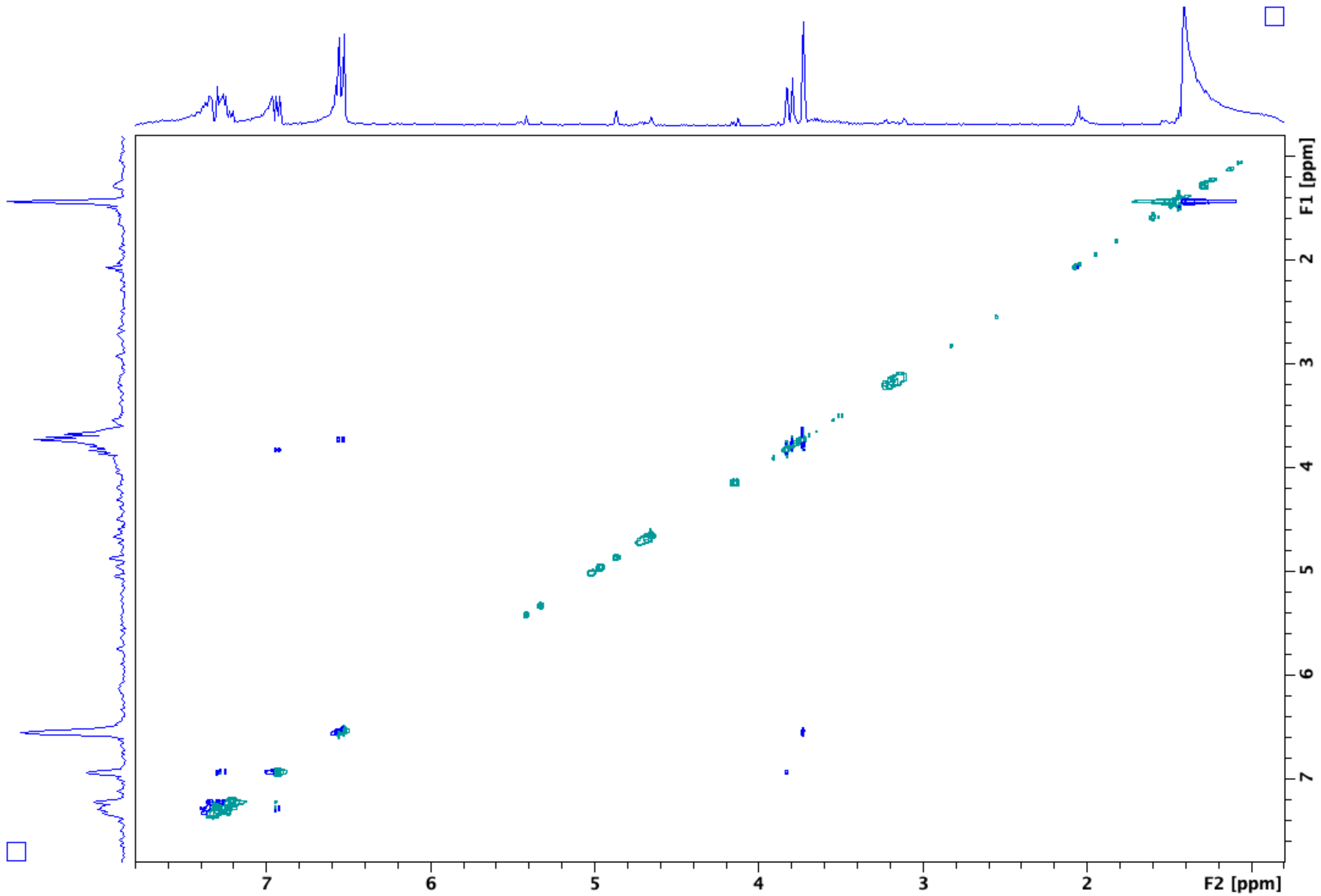

S1.50: 2D NOESY for **4** in $CDCl_3$, 400 MHz

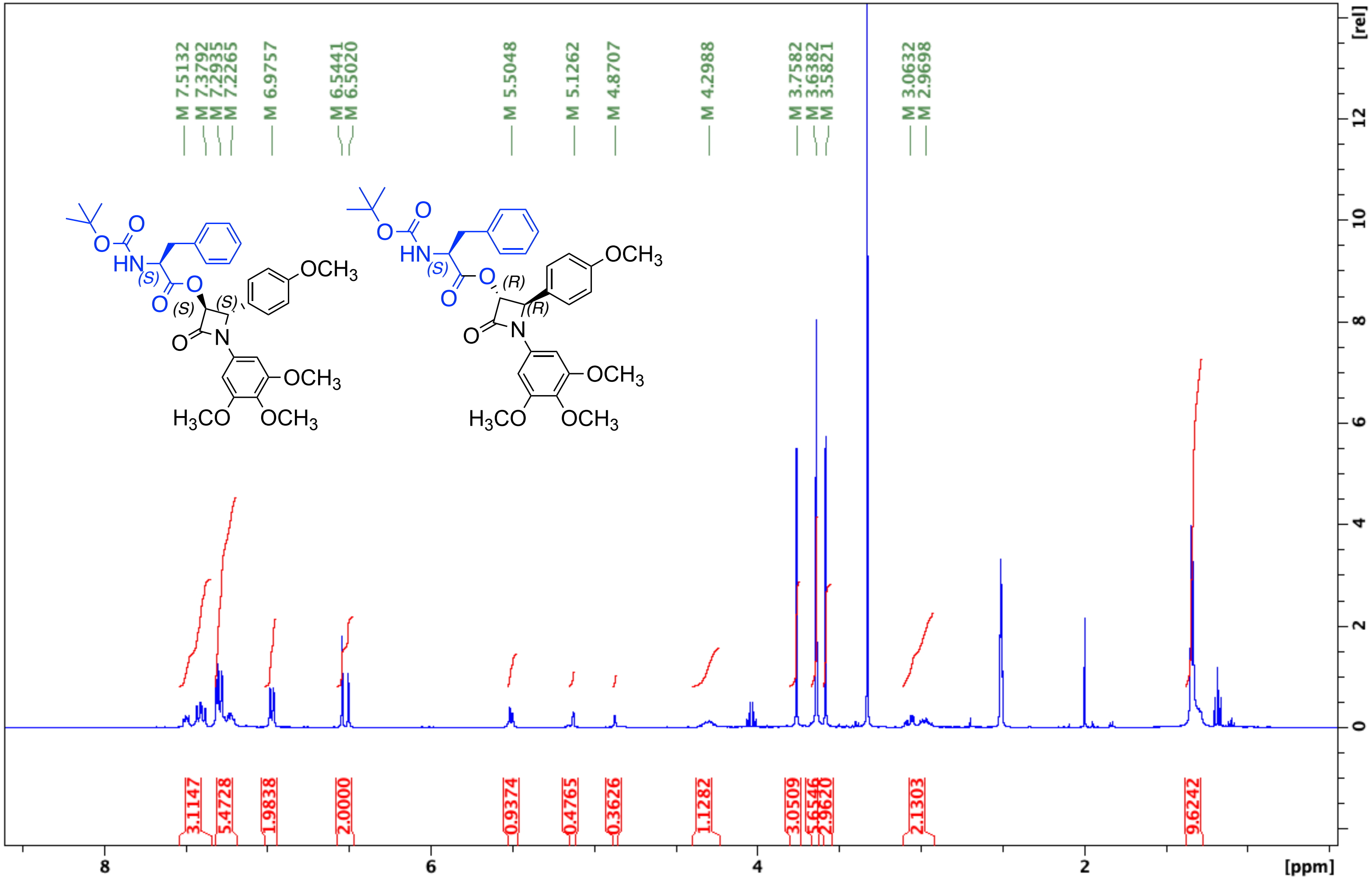

S1.51: ^{1}H NMR of **4,** DMSO-d_6 ,, 400 MHz. **Purity RP-HPLC:** 99%

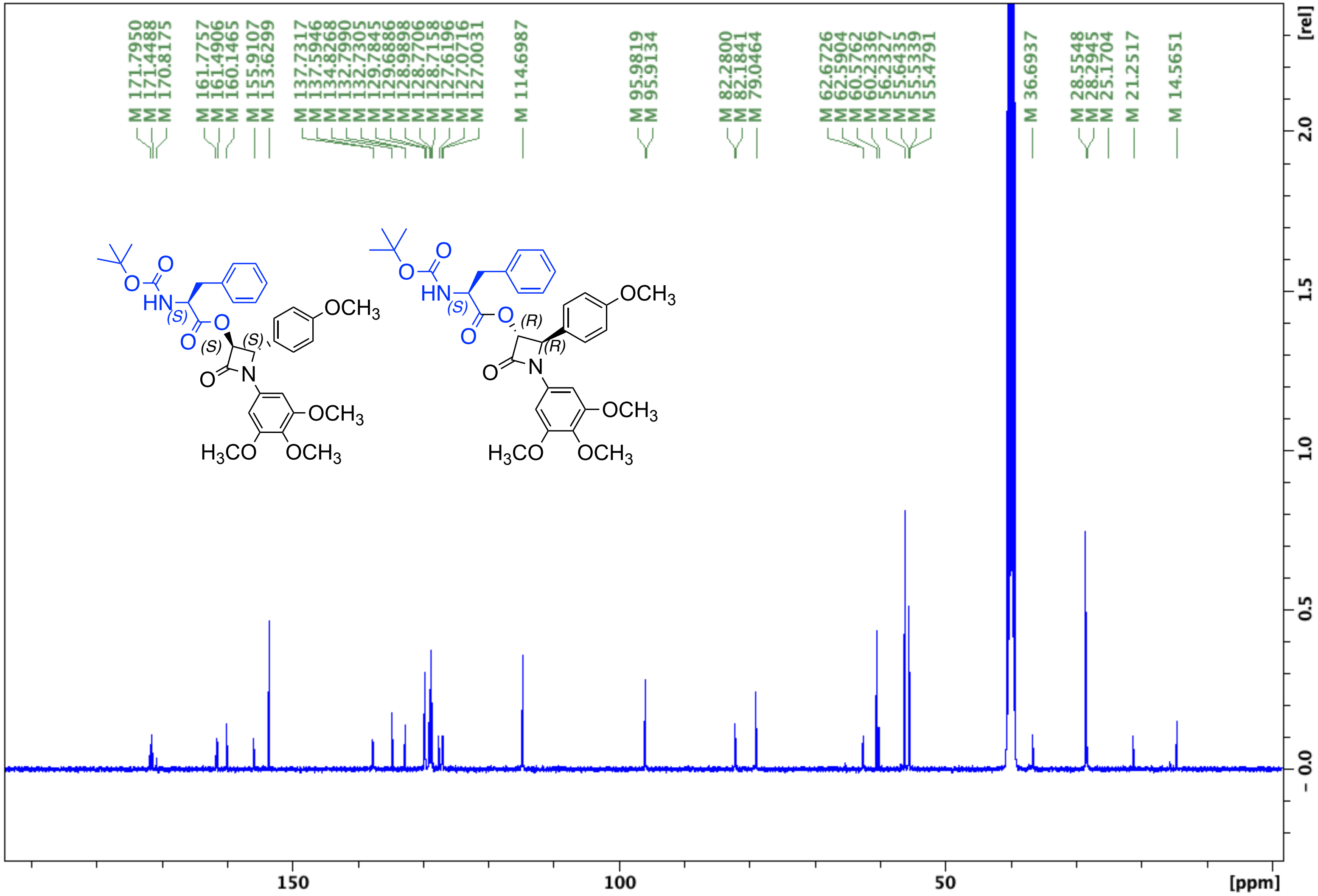

S1.52: ^{13}C NMR of **4,** DMSO-d_6 ,, 100 MHz. **Purity RP-HPLC:** 99%

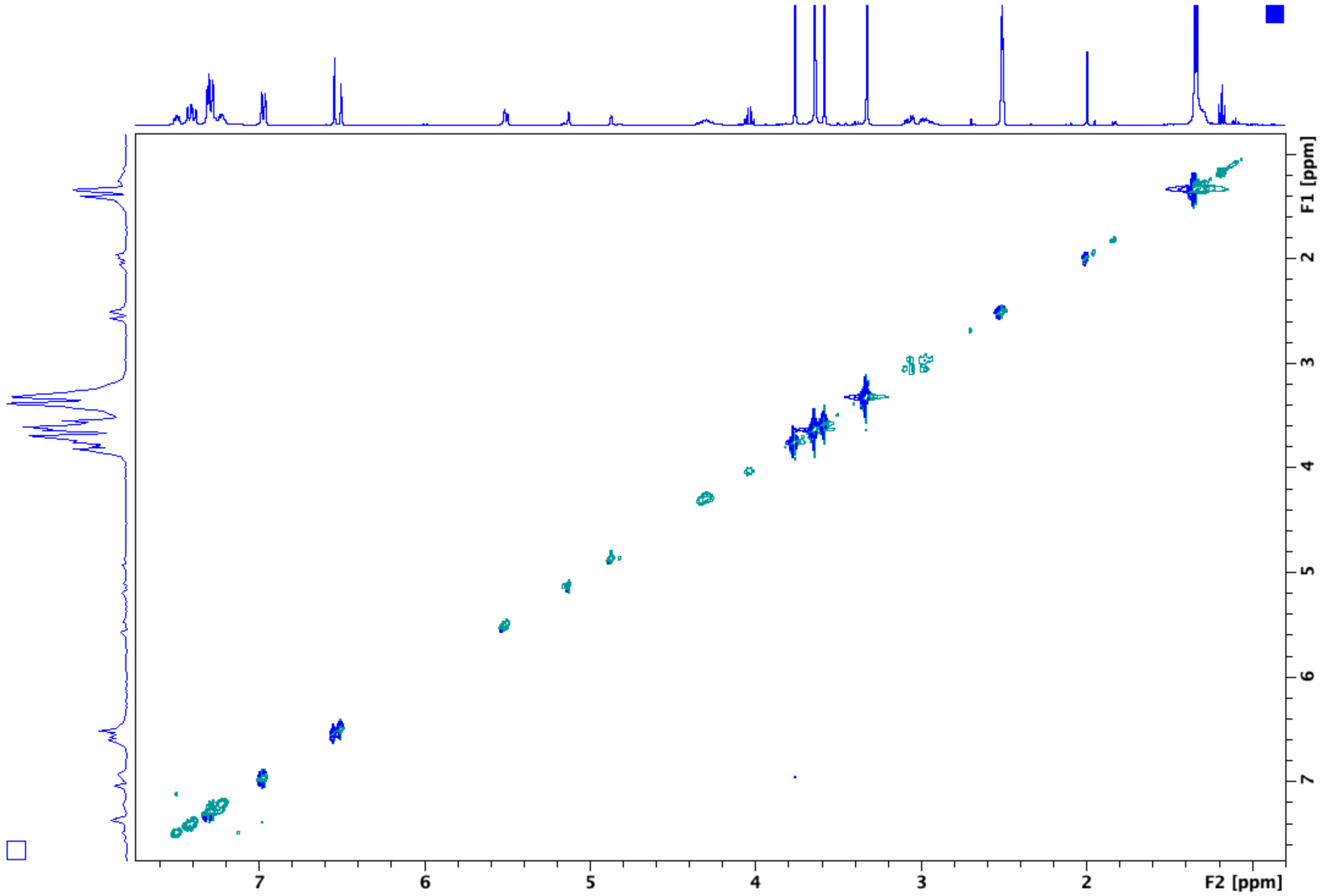

S1.53: 2D NOESY for **4** in DMSO-d_6 , 400 MHz

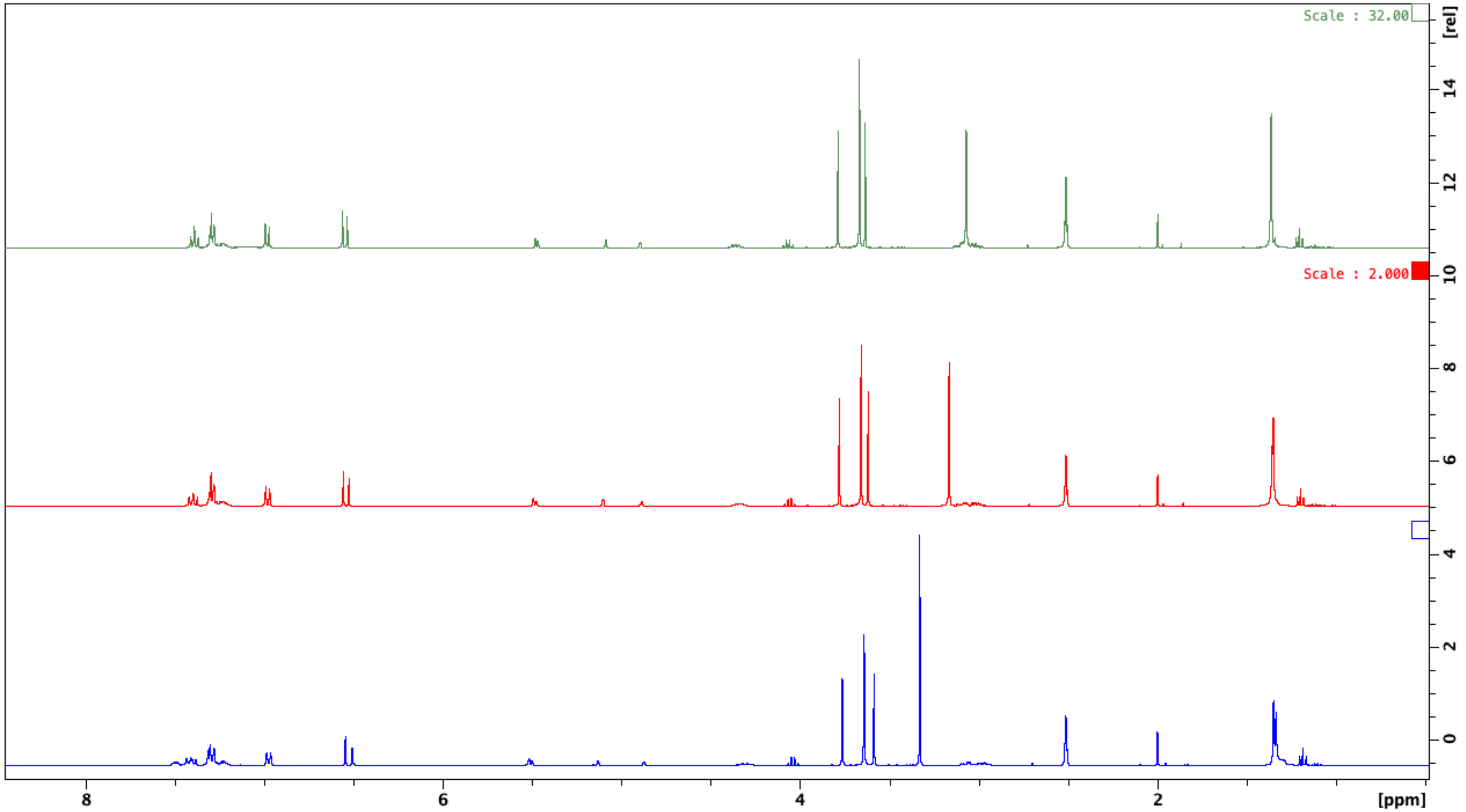

S1.54: VT Experiment for **4** [2-(4-methoxyphenyl)-4-oxo-1-(3,4,5-trimethoxyphenyl) azetidin-3-yl (*tert*-butoxycarbonyl)-*L*-phenylalaninate], DMSO-d_6 , 400 MHz; Blue: 25 °C, Red: 60 °C, Green: 80 °C

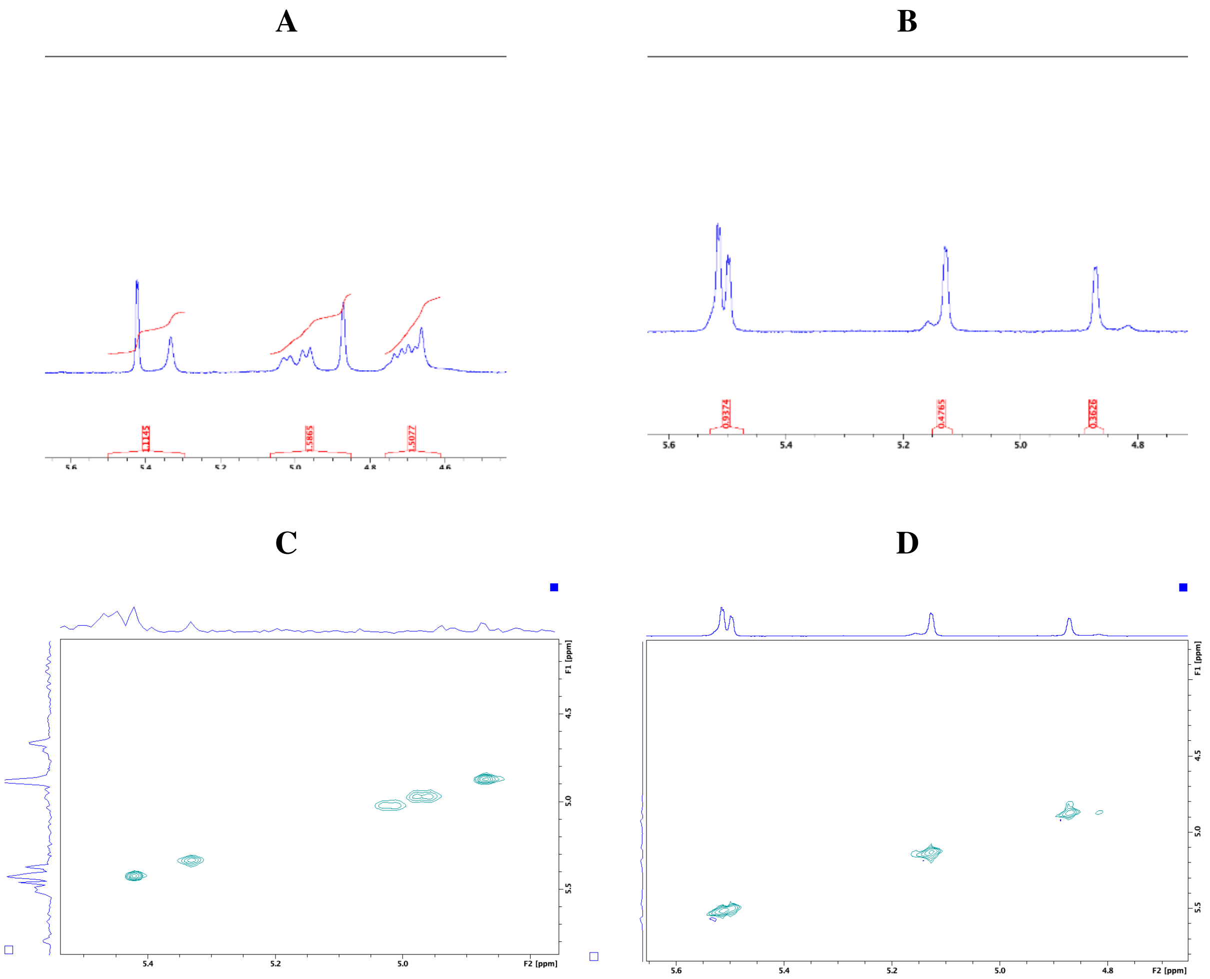

S1:55: H_3 and H_4 region for **4** on ^{1}H NMR in **A.** $CDCl_3$ at 400 MHz and **B**. DMSO-d_6 at 400 MHz. 2D NOESY for H_3 and H_4 region of **4** in **C:** $CDCl_3$ at 400 MHz and **D:** DMSO-d_6 at 400 MHz

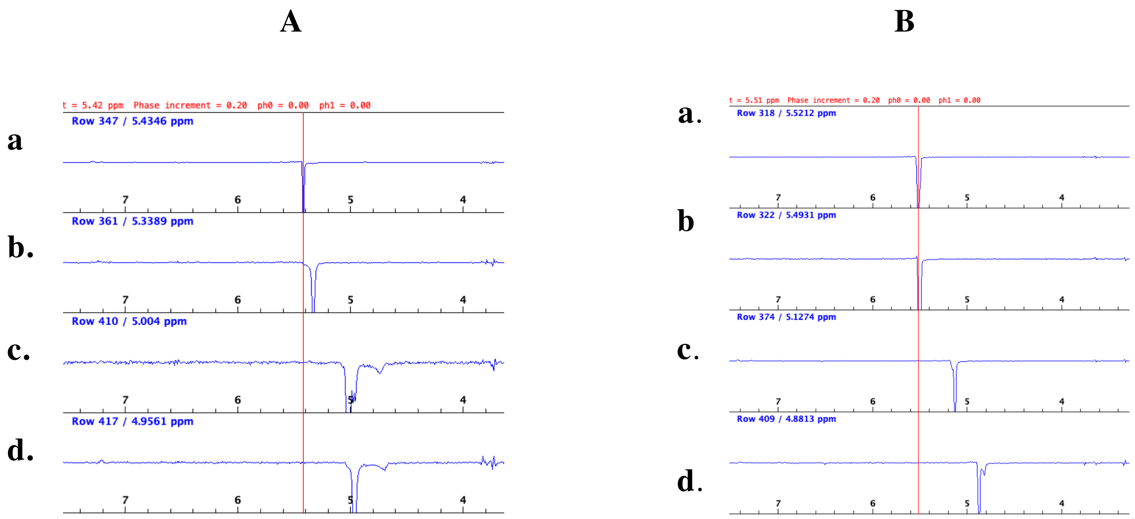

S1:56: A: Manual phasing of 2D NOESY for H_3 and H_4 for diastereomer mixture **4** in $CDCl_3$ at 400 MHz. **B:** in DMSO-d_6 at 400 MHz.

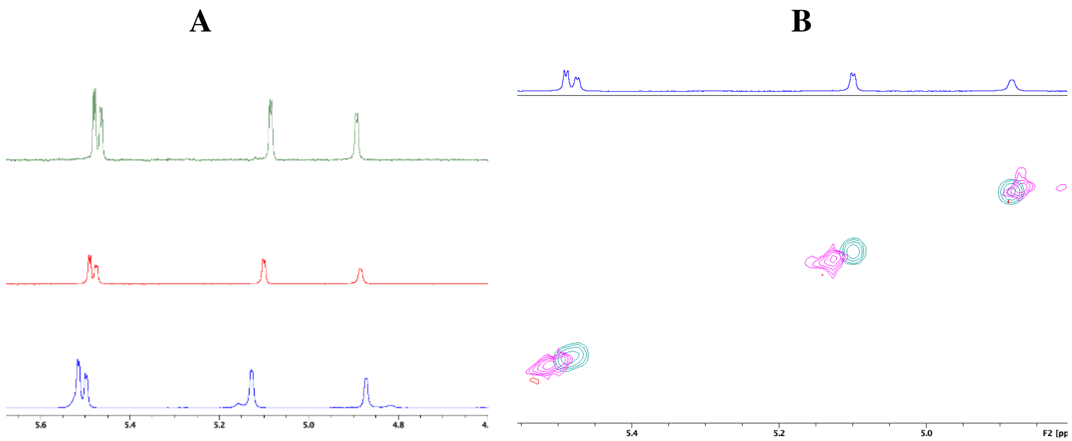

S1:57: VT experiment for **4** in DMSO-d_6 at 400 MHz. .**A:** ^{1}H NMR spectrum with enlargement of H_3 & H_4 region shown. Blue: 25 °C, Red: 60 °C, Green: 80 °C **B:** Green: 2D NOESY at 25°C , Pink: 2D NOESY at 80 °C.

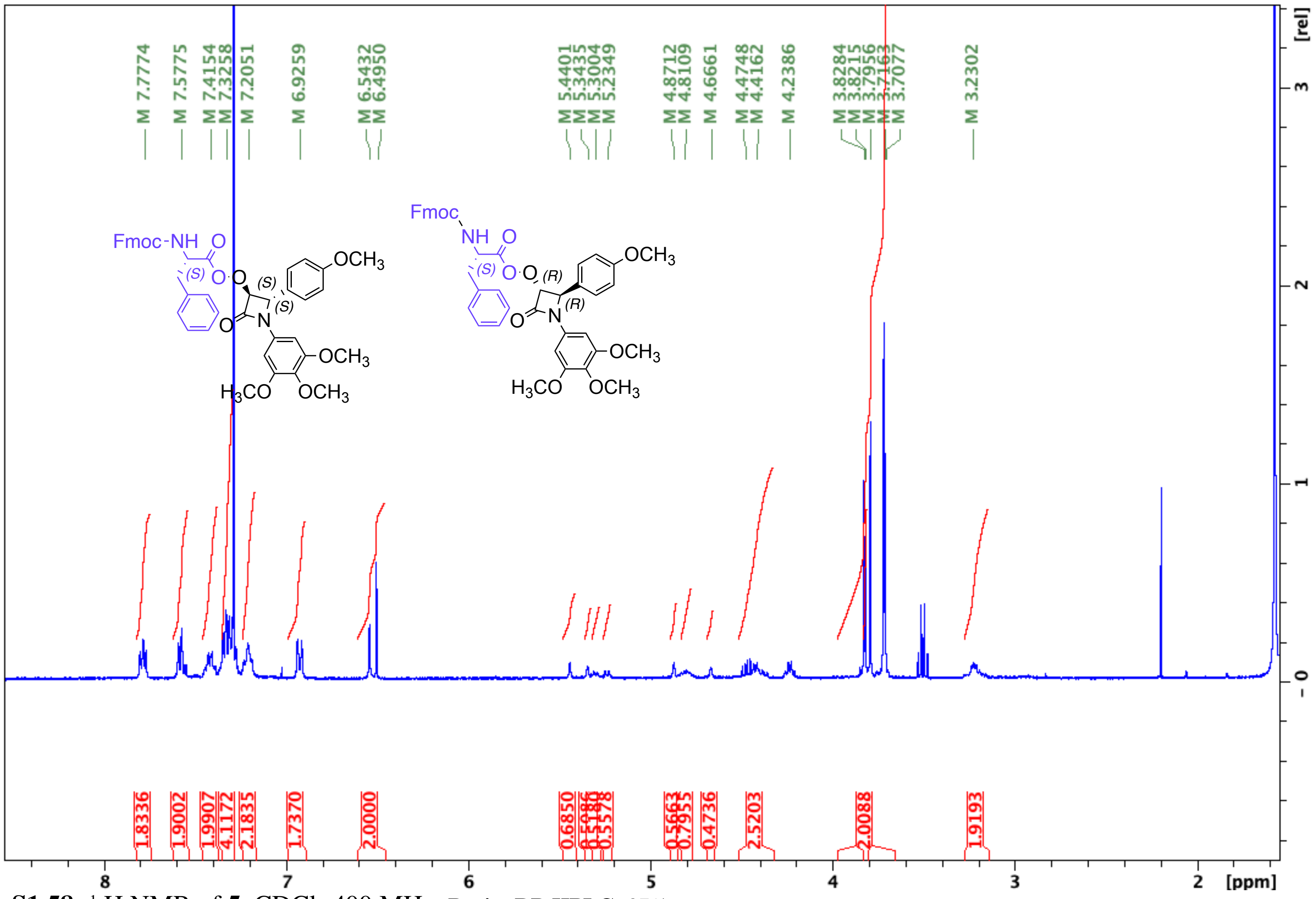

S1.58: [1] H NMR of **5**, $CDCl_3$, 400 MHz. **Purity RP-HPLC:** 87%

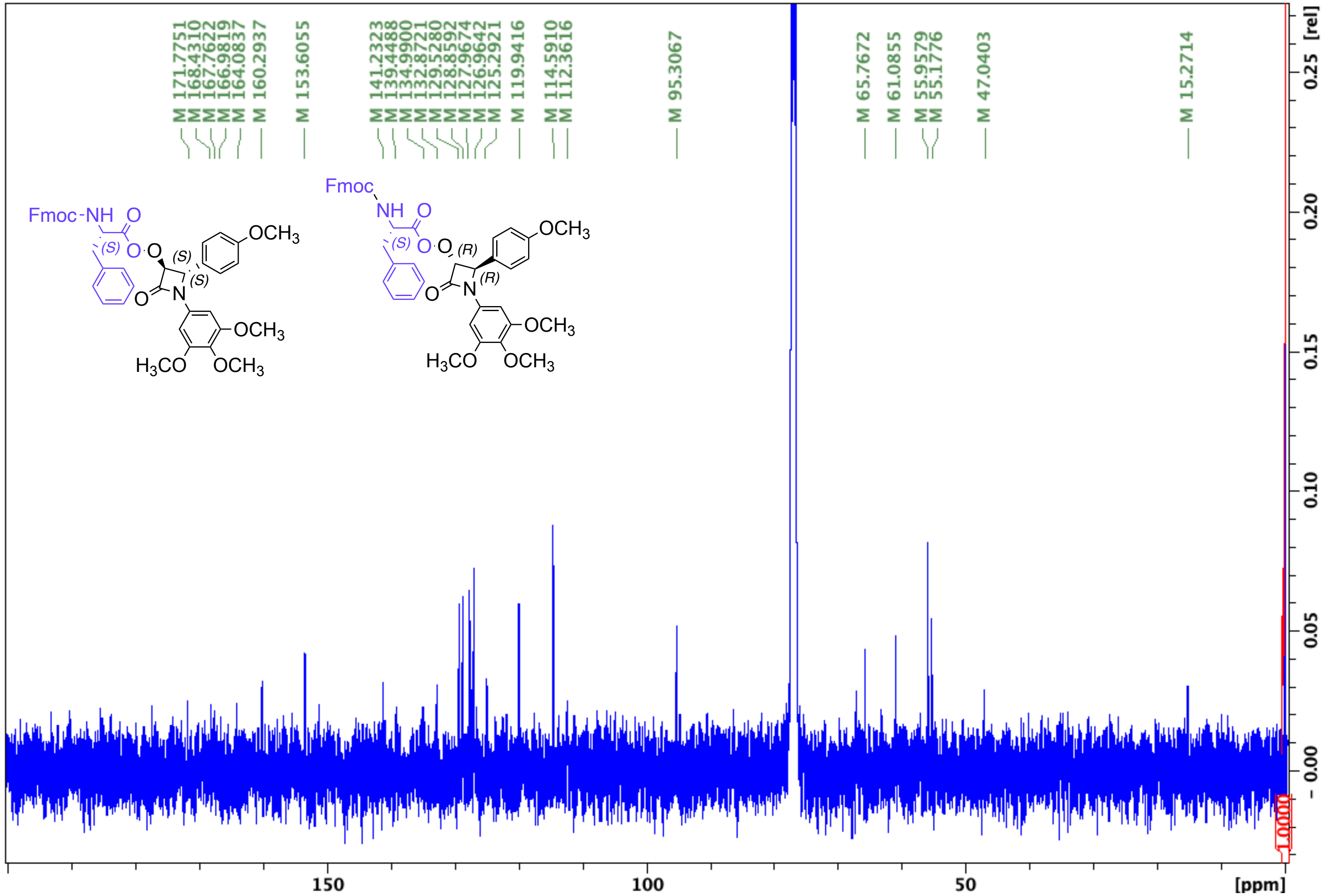

S1.59: ^{13}C NMR of **5**, $CDCl_3$, 100 MHz

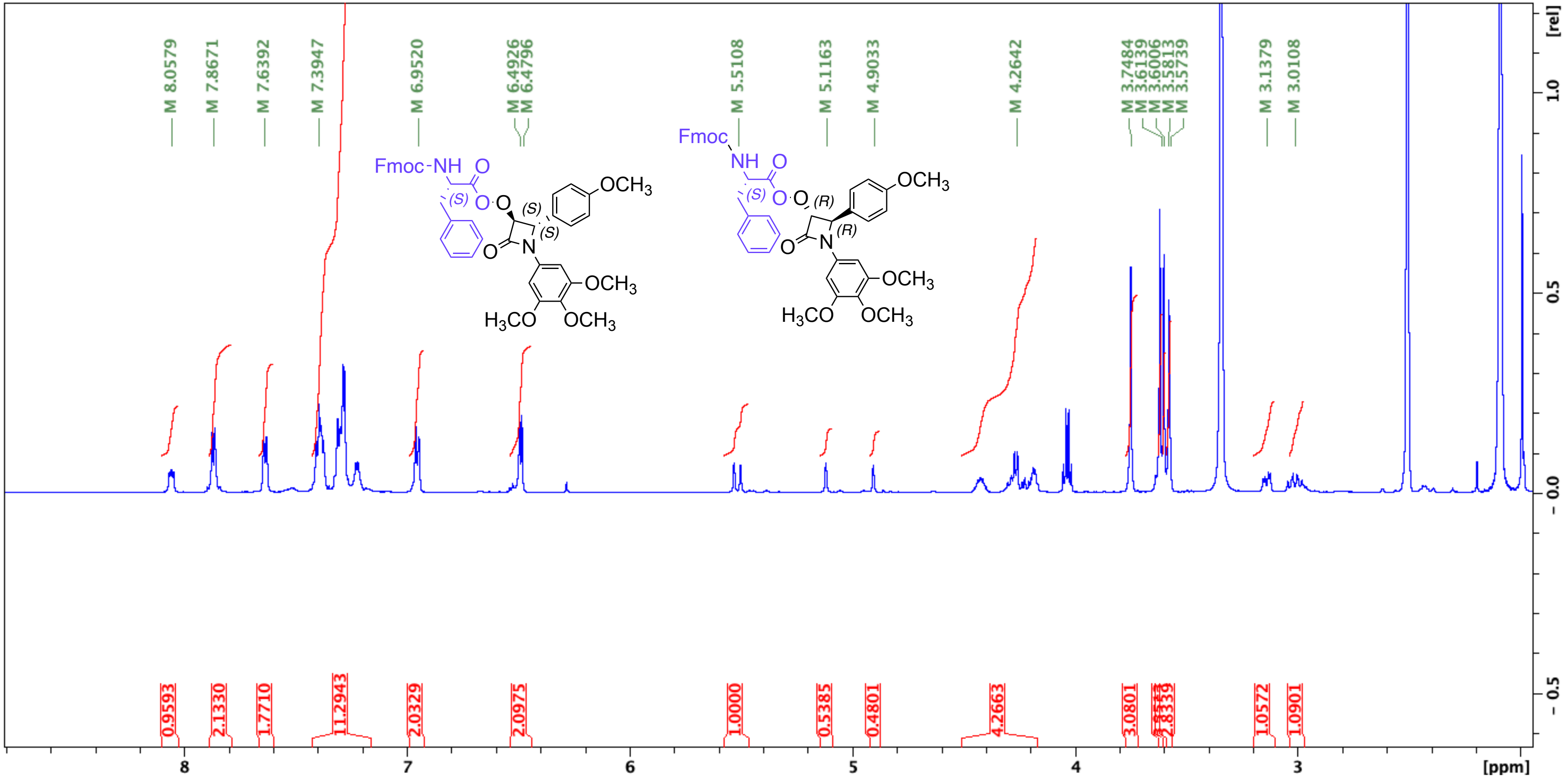

S1.60: ^{1}H NMR of **5,** DMSO-d_6 at 600 MHz. **Purity RP-HPLC:** 87% (Impurities present from 0-1 ppm and unlabelled)

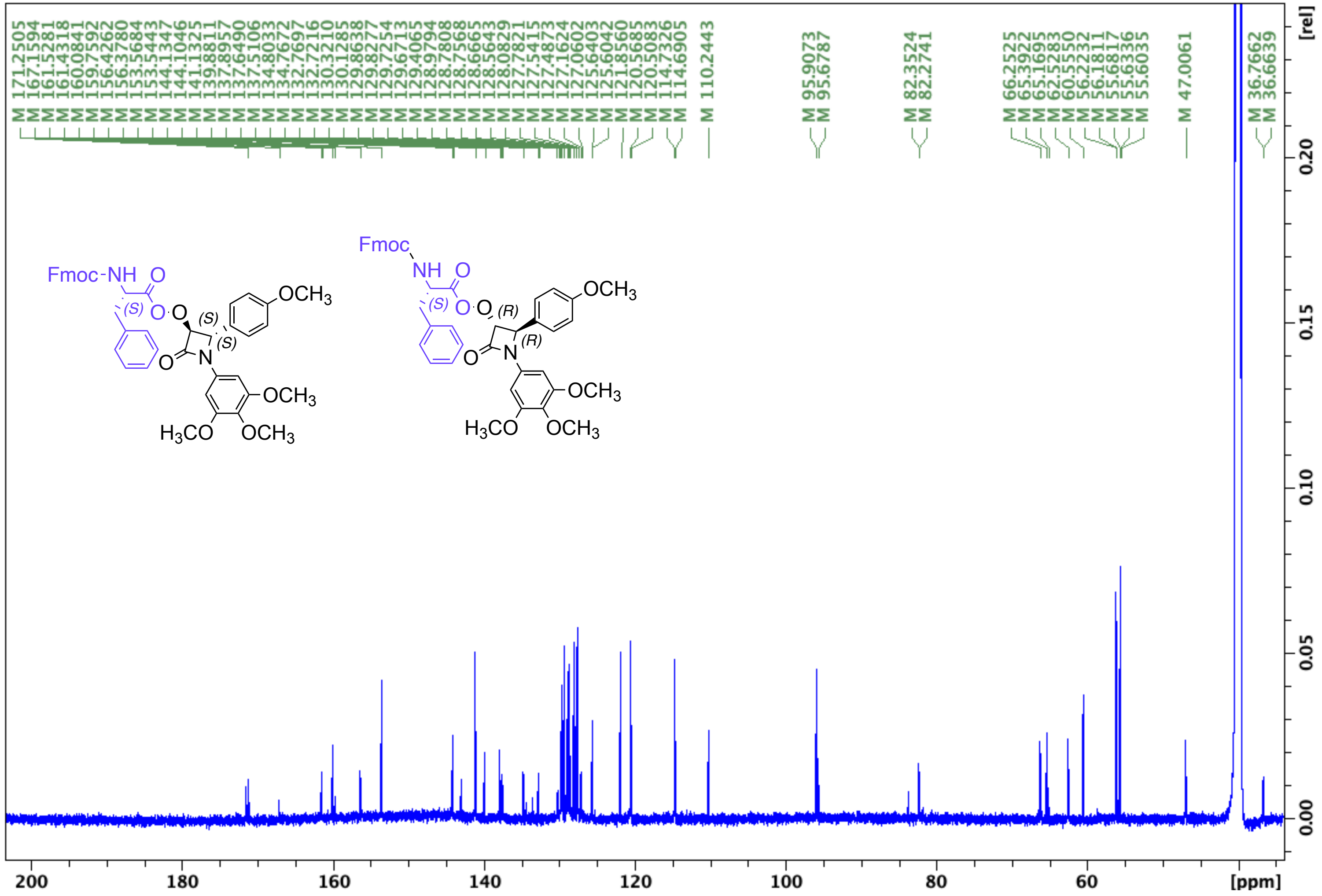

S1.61 ^{13}C NMR of **5**, DMSO-d_6 at 100 MHz. **Purity RP-HPLC:** 87%

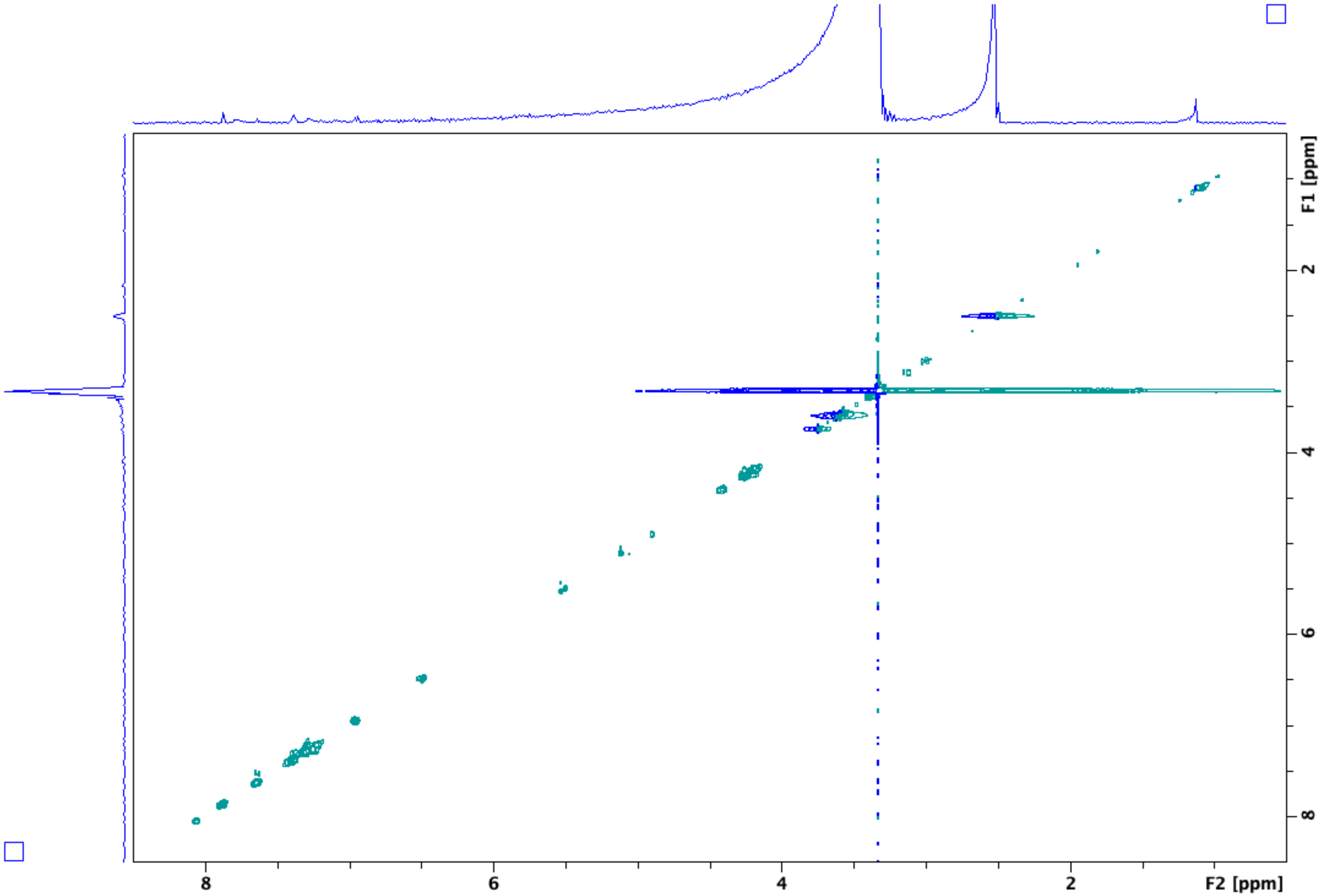

S1.62 2D NOESY for **5,** DMSO-d_6 , 400 MHz

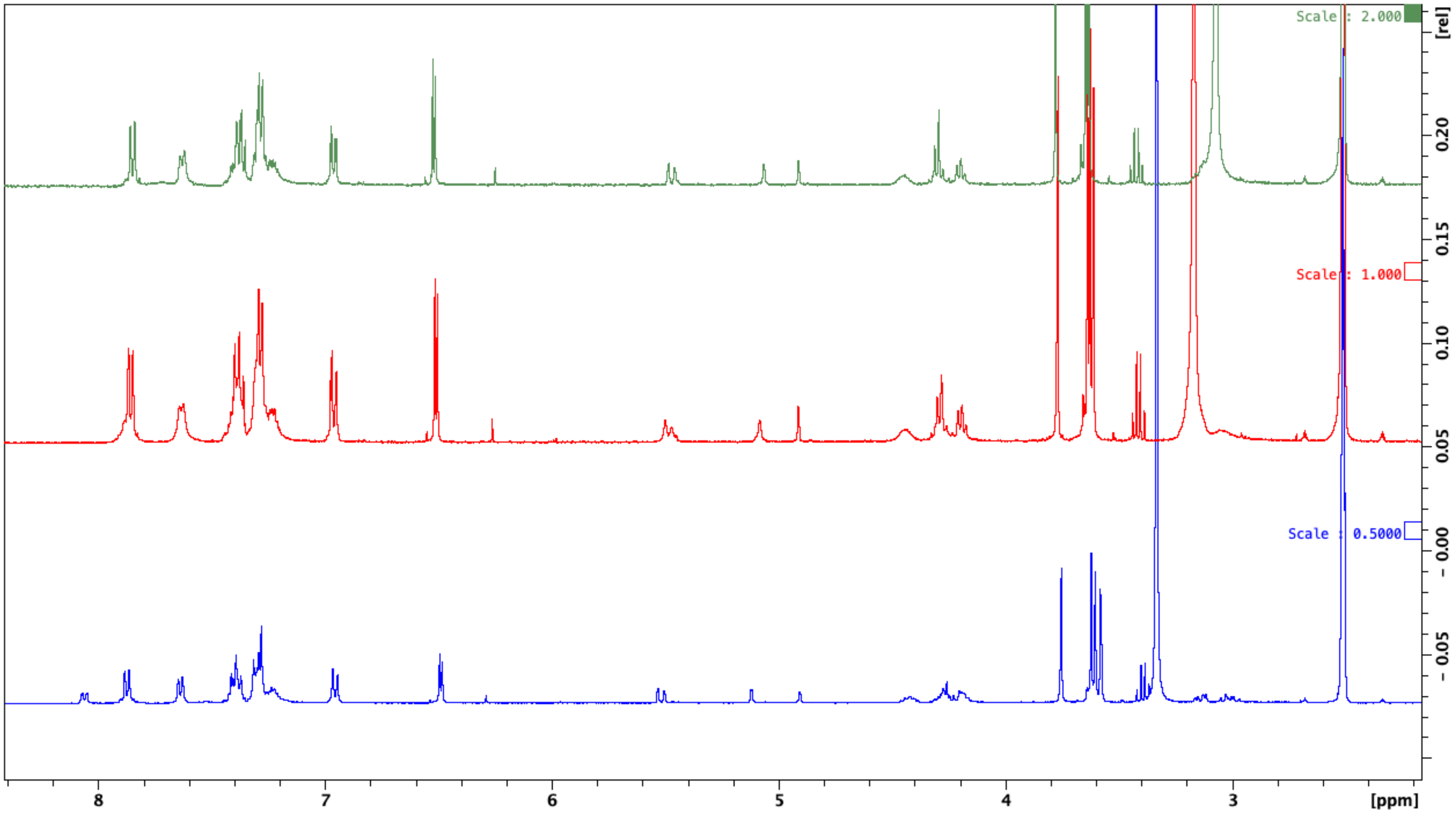

S1.63: VT Experiment for **5**, DMSO-d_6 , 400 MHz; Blue: 25 °C, Red: 60 °C, Green: 80 °C

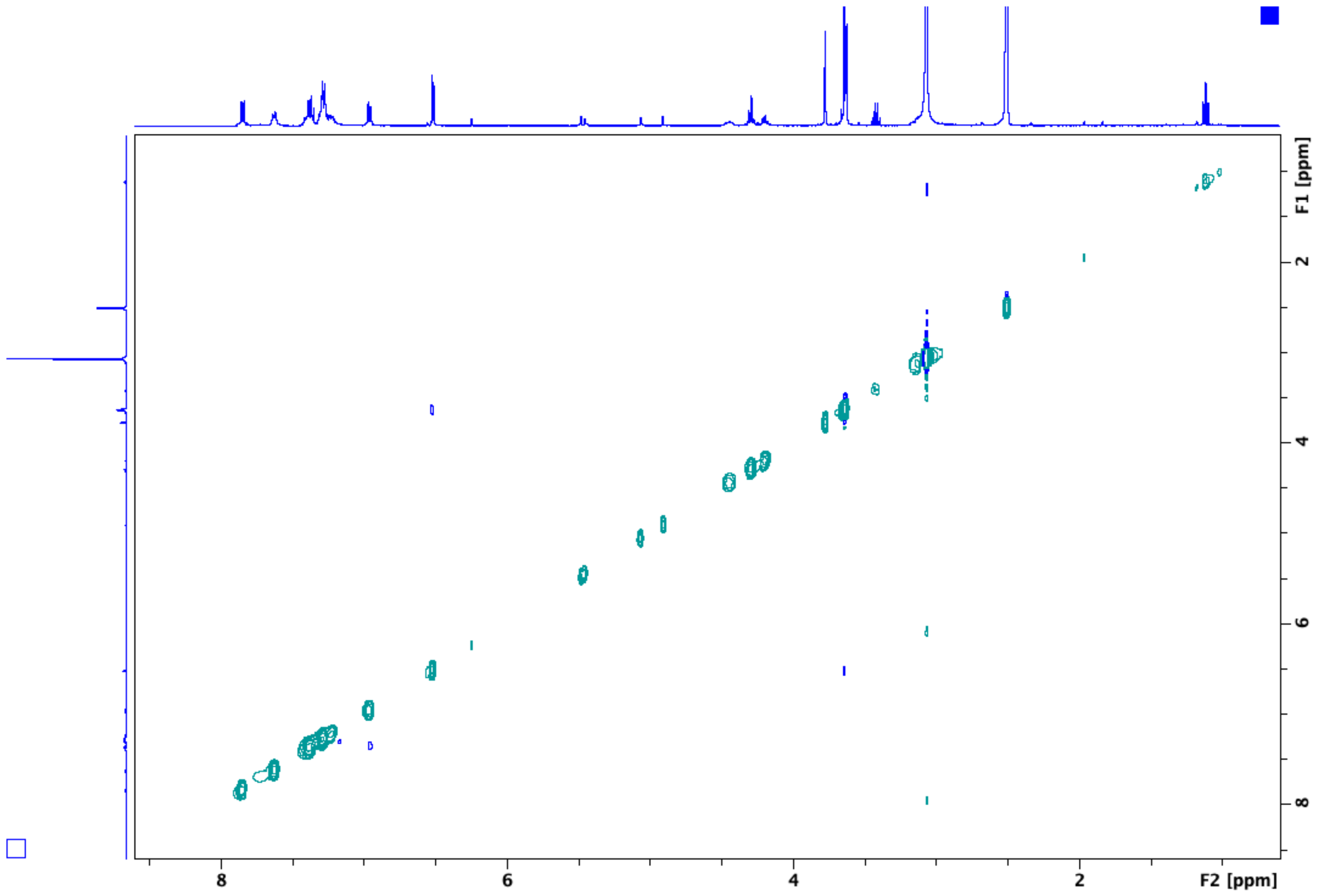

S1.64 VT 2D NOESY for **5,** DMSO-d_6 ,, 400 MHz at 85 °C. **Purity RP-HPLC:** 87%

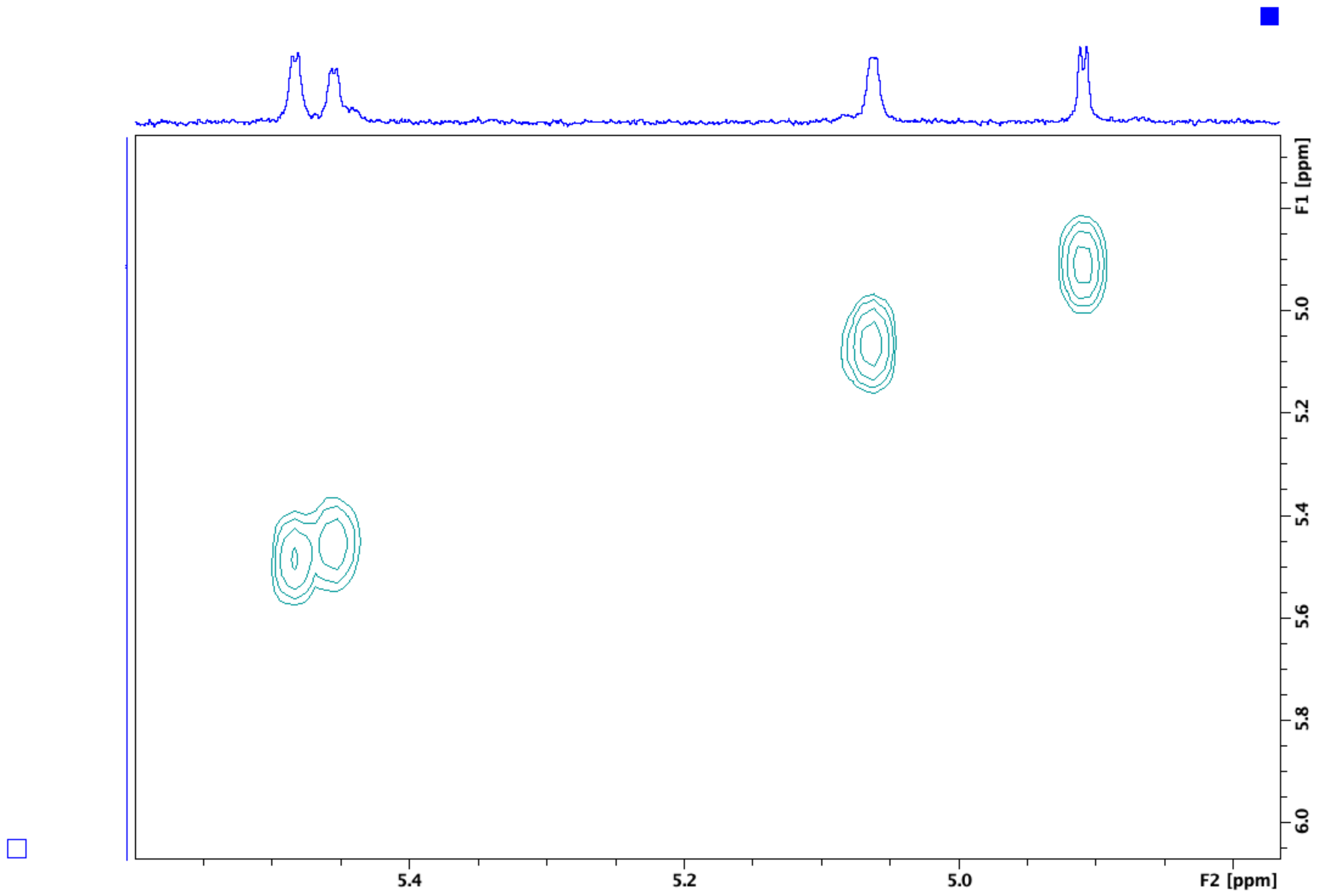

S1.65 VT 2D NOESY for **5,** H_3 & H_4 region shown, DMSO-d_6, 400 MHz at 85 °C

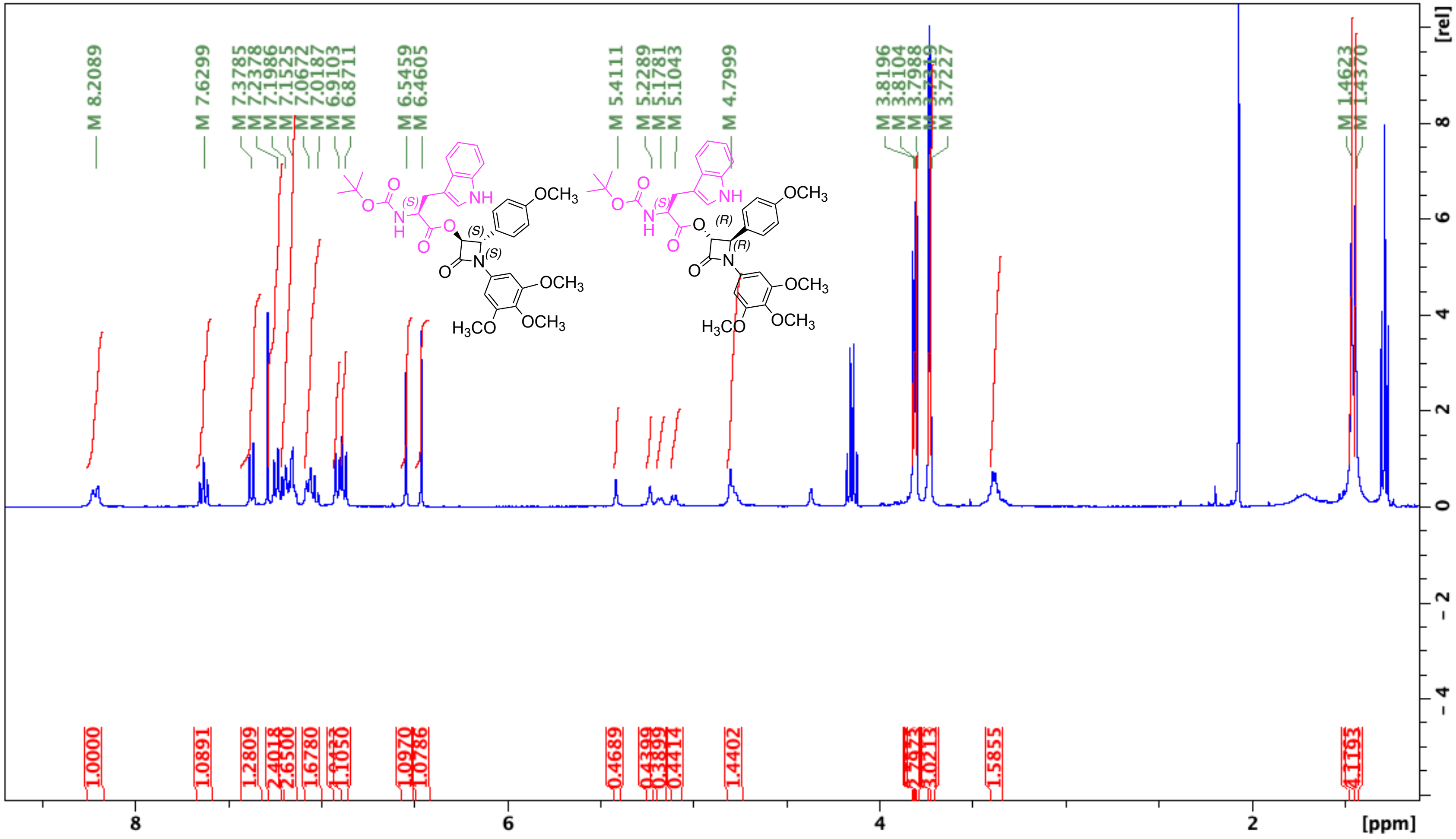

S1.66 ^{1}H of **6**, $CDCl_3$ at 400 MHz. **Purity RP-HPLC:** 99%

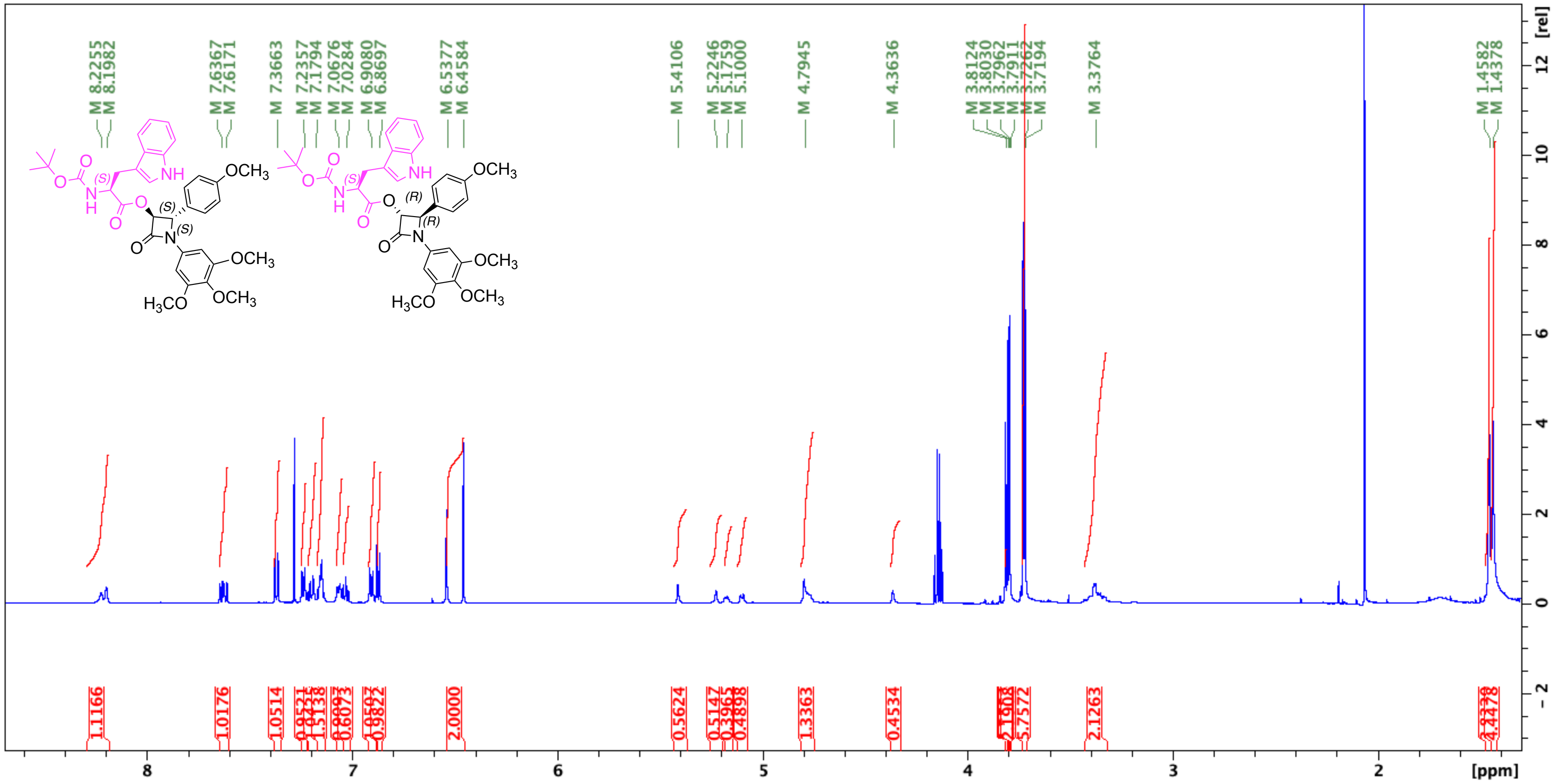

S1.67 ^{1}H of **6** in $CDCl_3$ at 600 MHz. **Purity RP-HPLC:** 99%

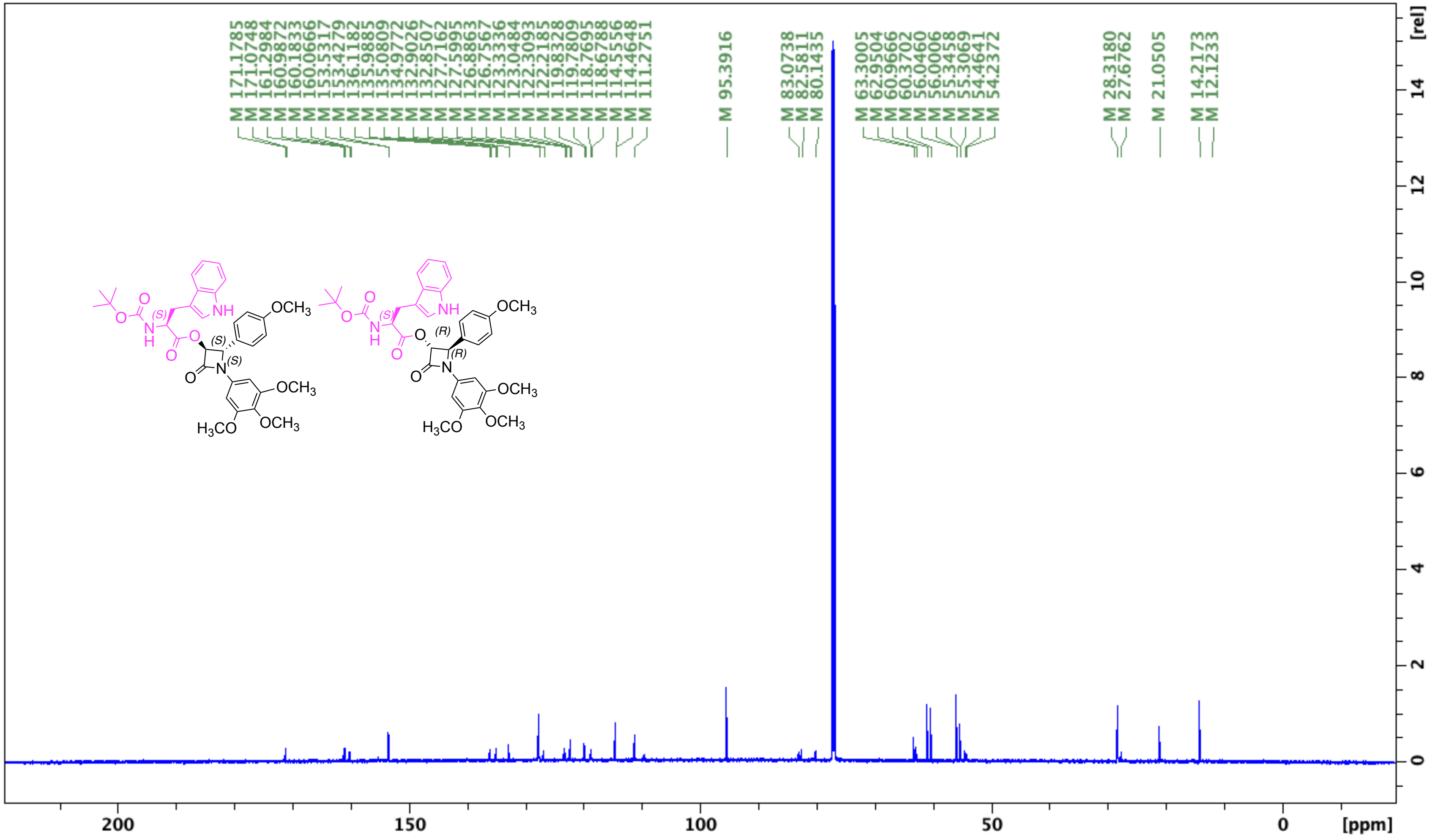

S1.68 ^{13}C of **6,** $CDCl_3$ at 100 MHz. **Purity RP-HPLC:** 99%

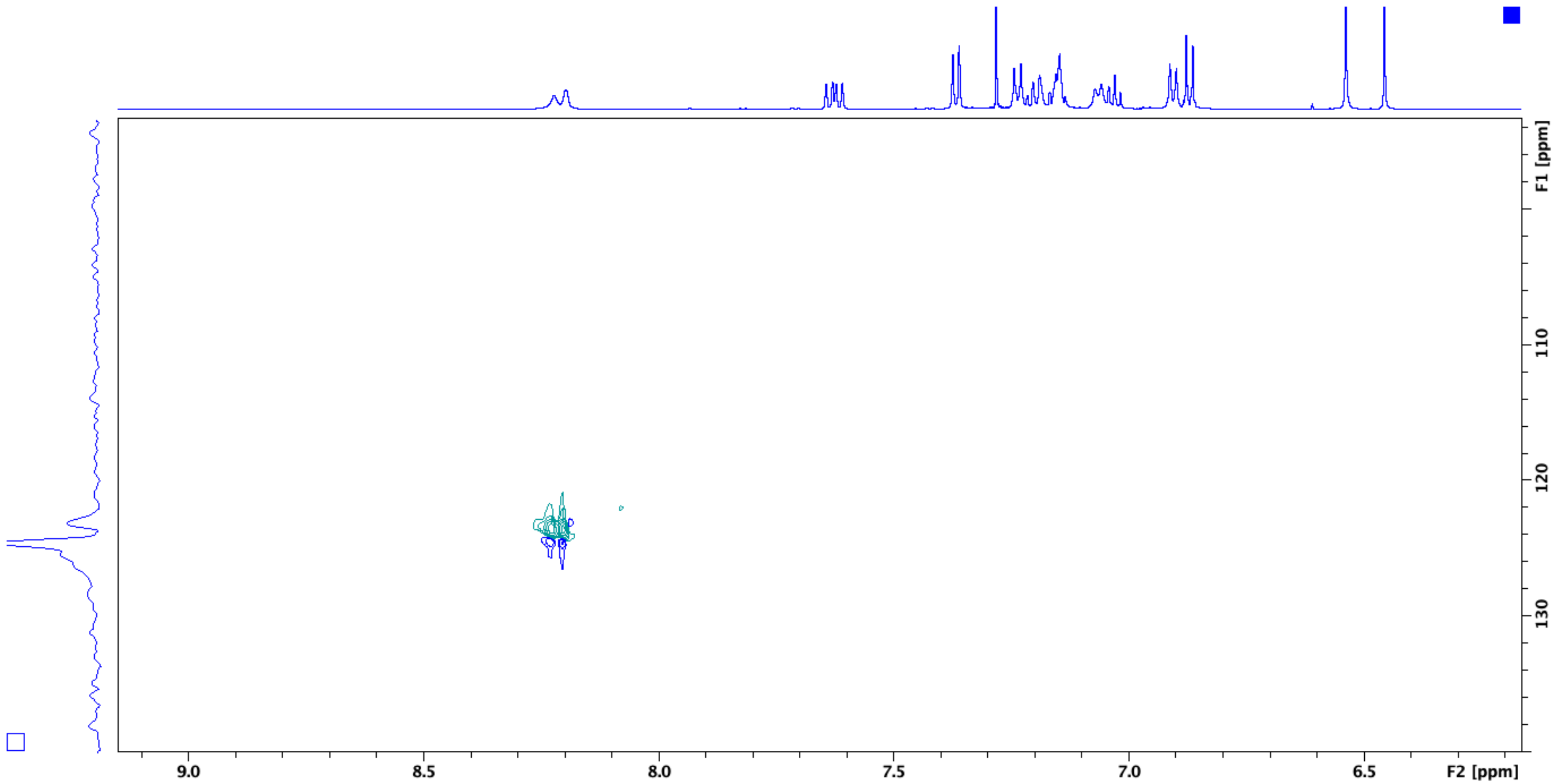

S1.69 ^{15}N HSQC of **6** 2-(4-methoxyphenyl)-4-oxo-1-(3,4,5-trimethoxyphenyl)azetidin-3-yl (*tert*-butoxycarbonyl)-*L*-tryptophanate in $CDCl_3$ at 60.8 MHz demonstrating Tryptophan ring secondary amine (NH) at 8.22 and 8.19 on ^{1}H NMR correlating with the corresponding nitrogens on ^{15}N NMR at 125.3 and 123.3 ppm.

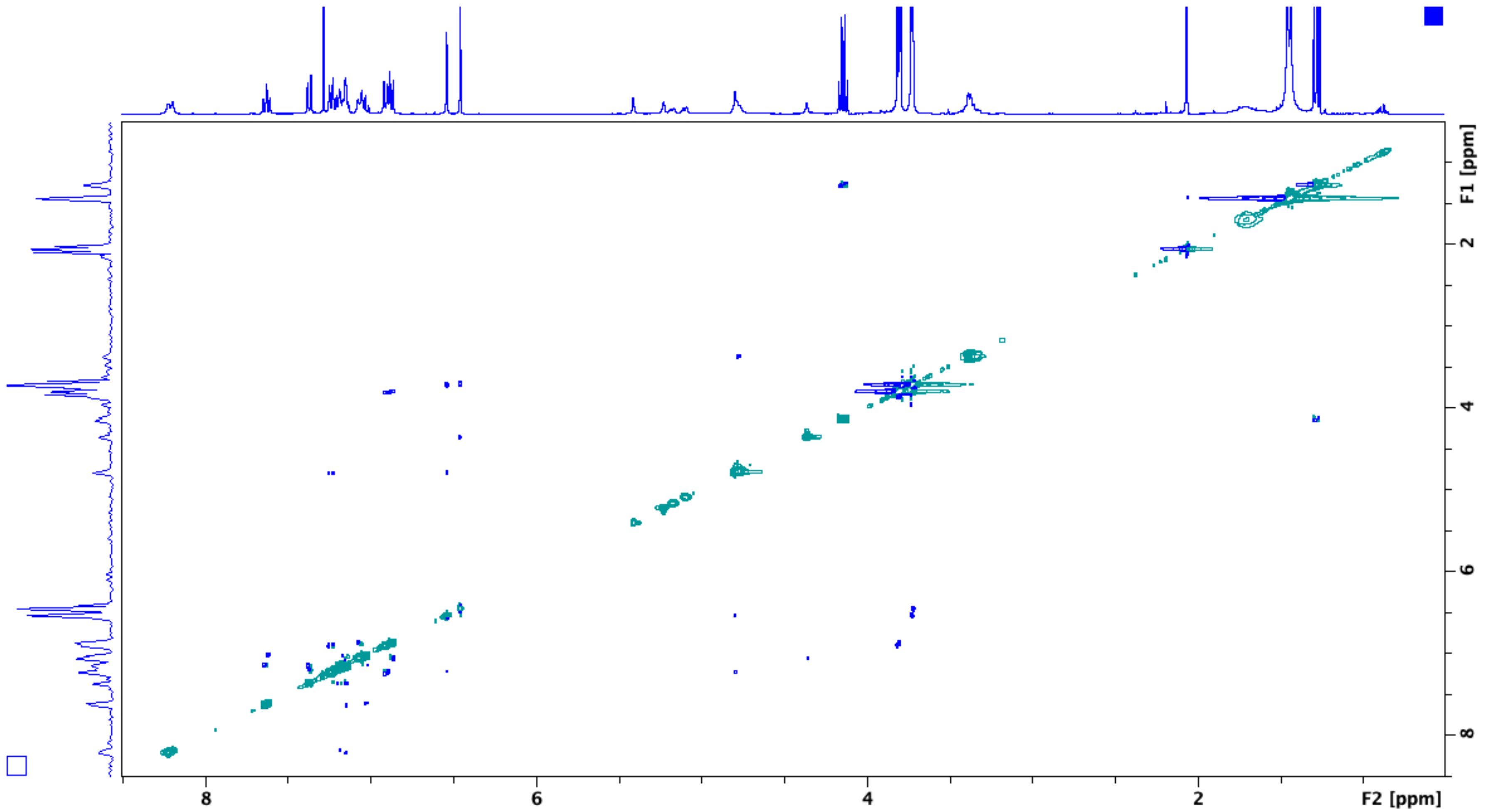

S1.70 2D NOESY of **6** in $CDCl_3$ at 400 MHz at 25 °C. **Purity RP-HPLC:** 99%

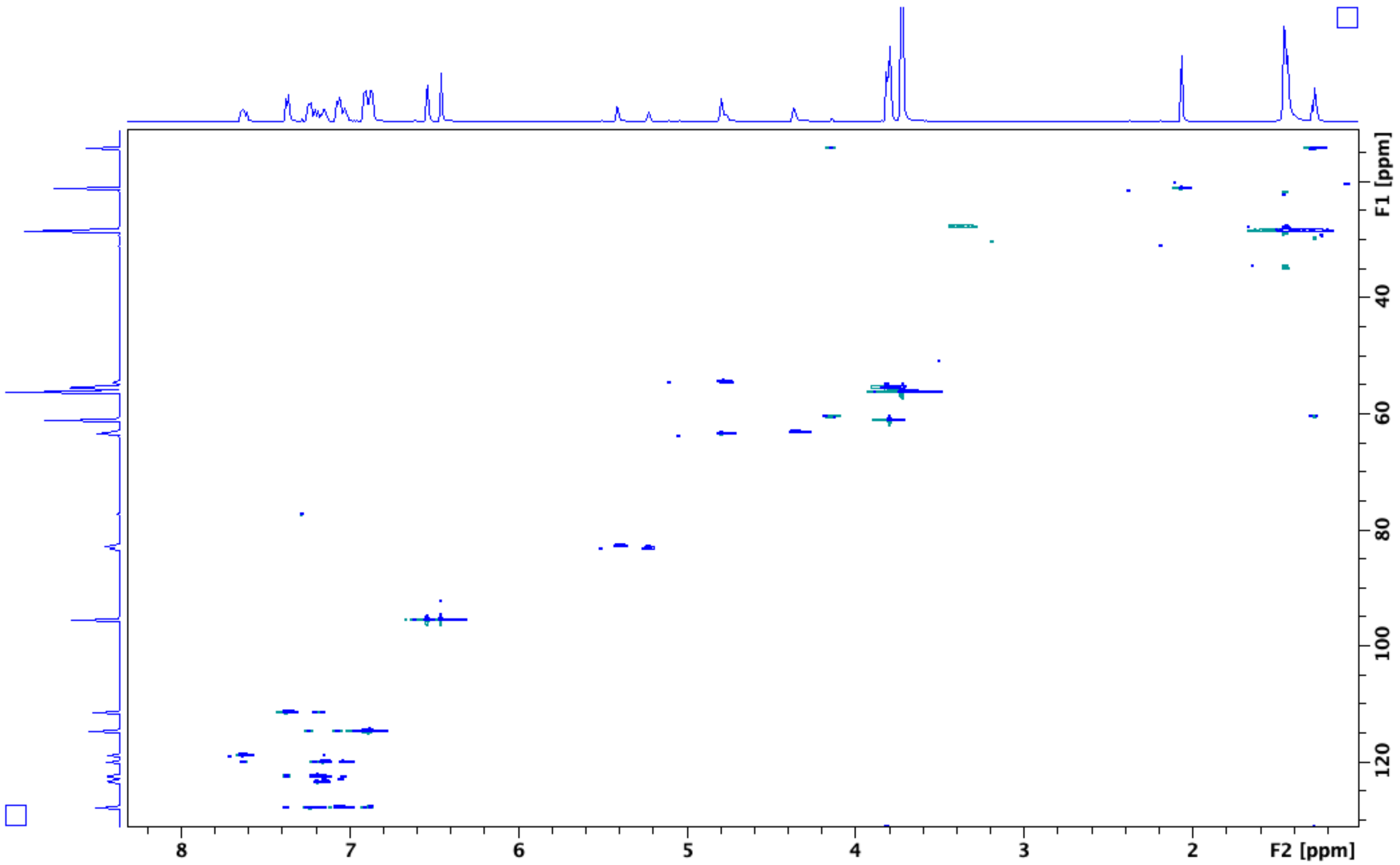

S1.71 HSQC of **6** in $CDCl_3$ at 600 MHz. **Purity RP-HPLC:** 99%

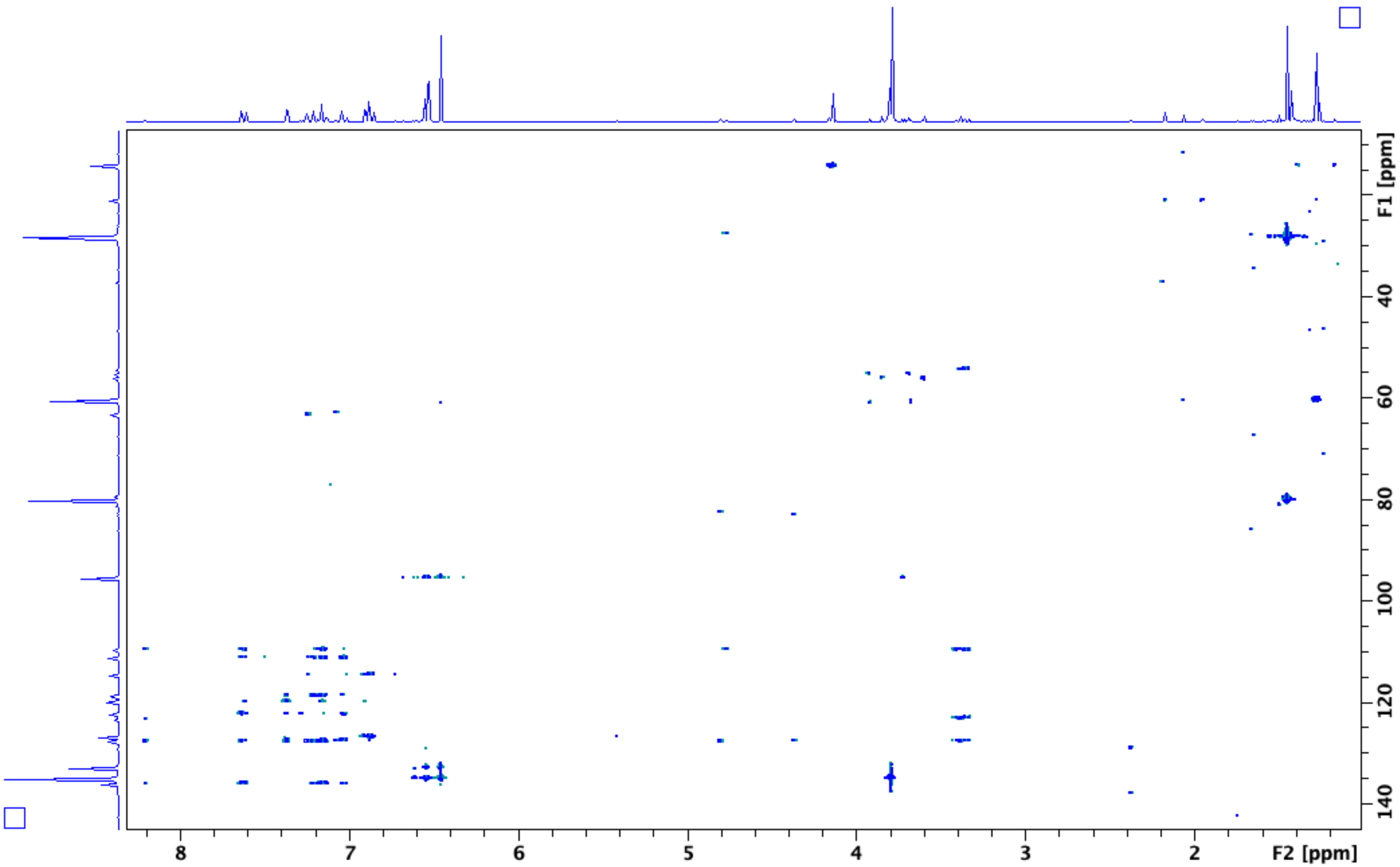

S1.72 HMBC of **6**, $CDCl_3$ at 600 MHz. **Purity RP-HPLC:** 99%

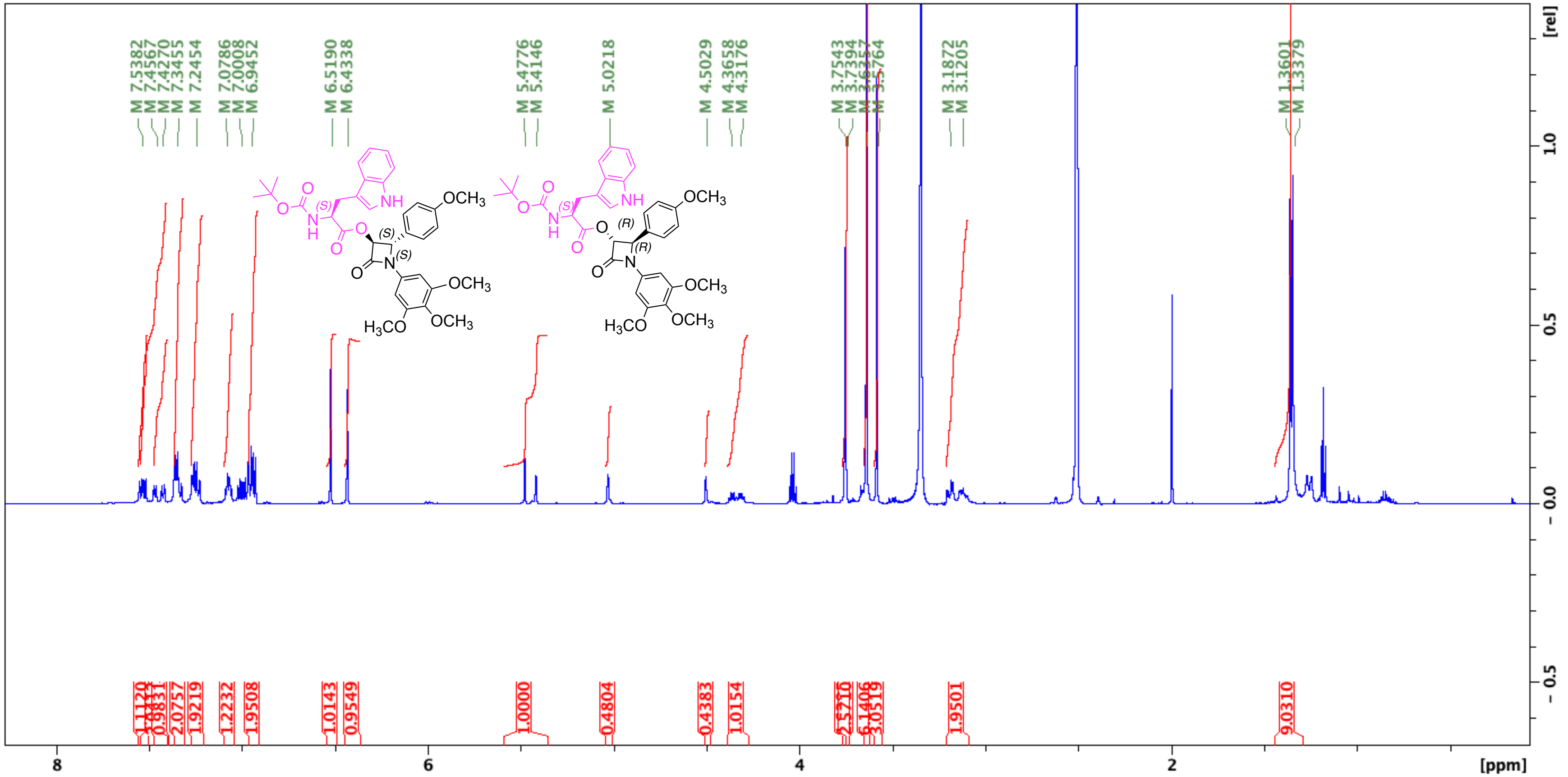

S1.73 ^{1}H NMR of **6** in DMSO-d_6 at 600 MHz. **Purity RP-HPLC:** 99%

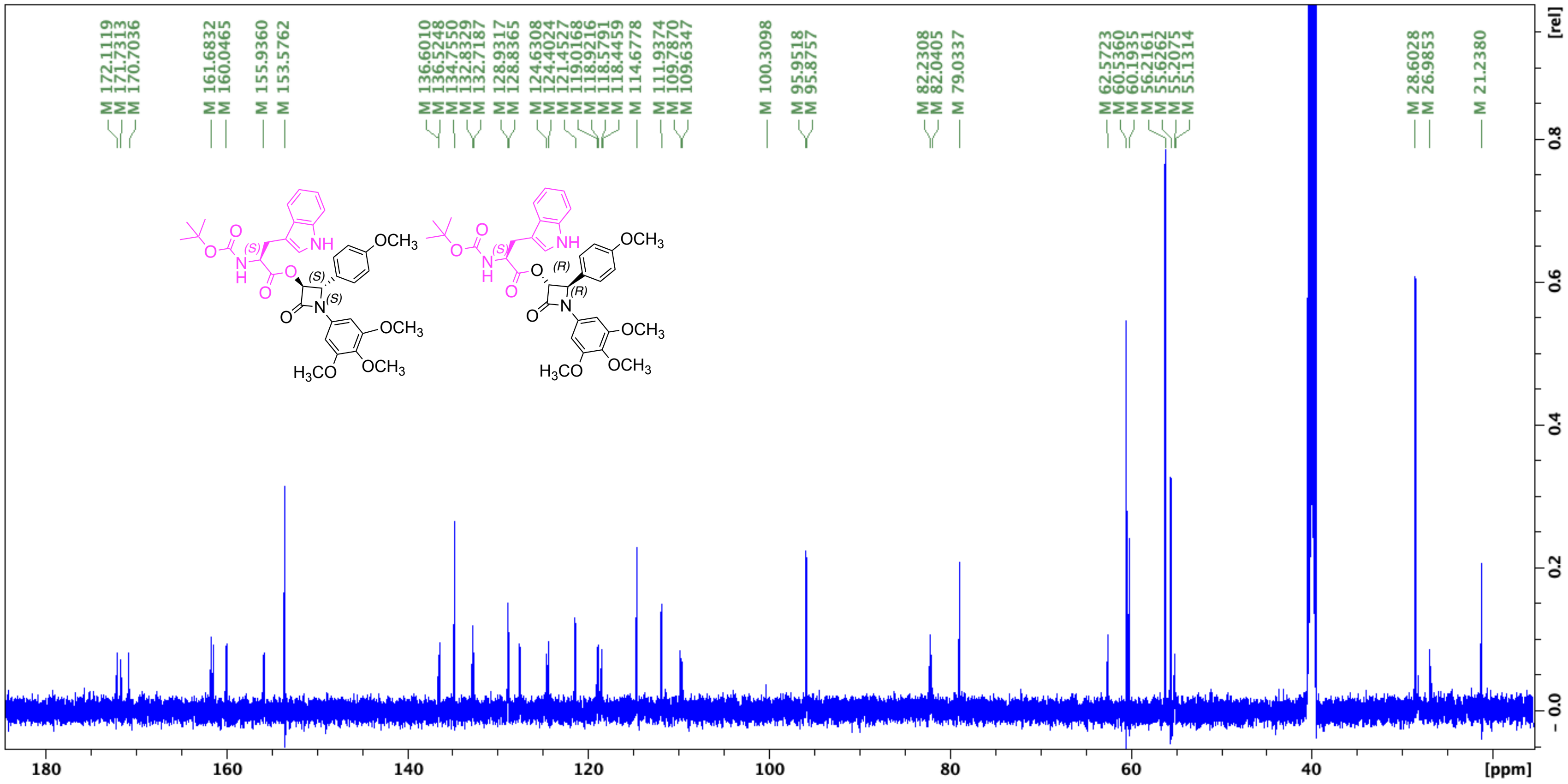

S1.74 ^{13}C of **6** in DMSO-d_6 at 100 MHz. **Purity RP-HPLC:** 99%

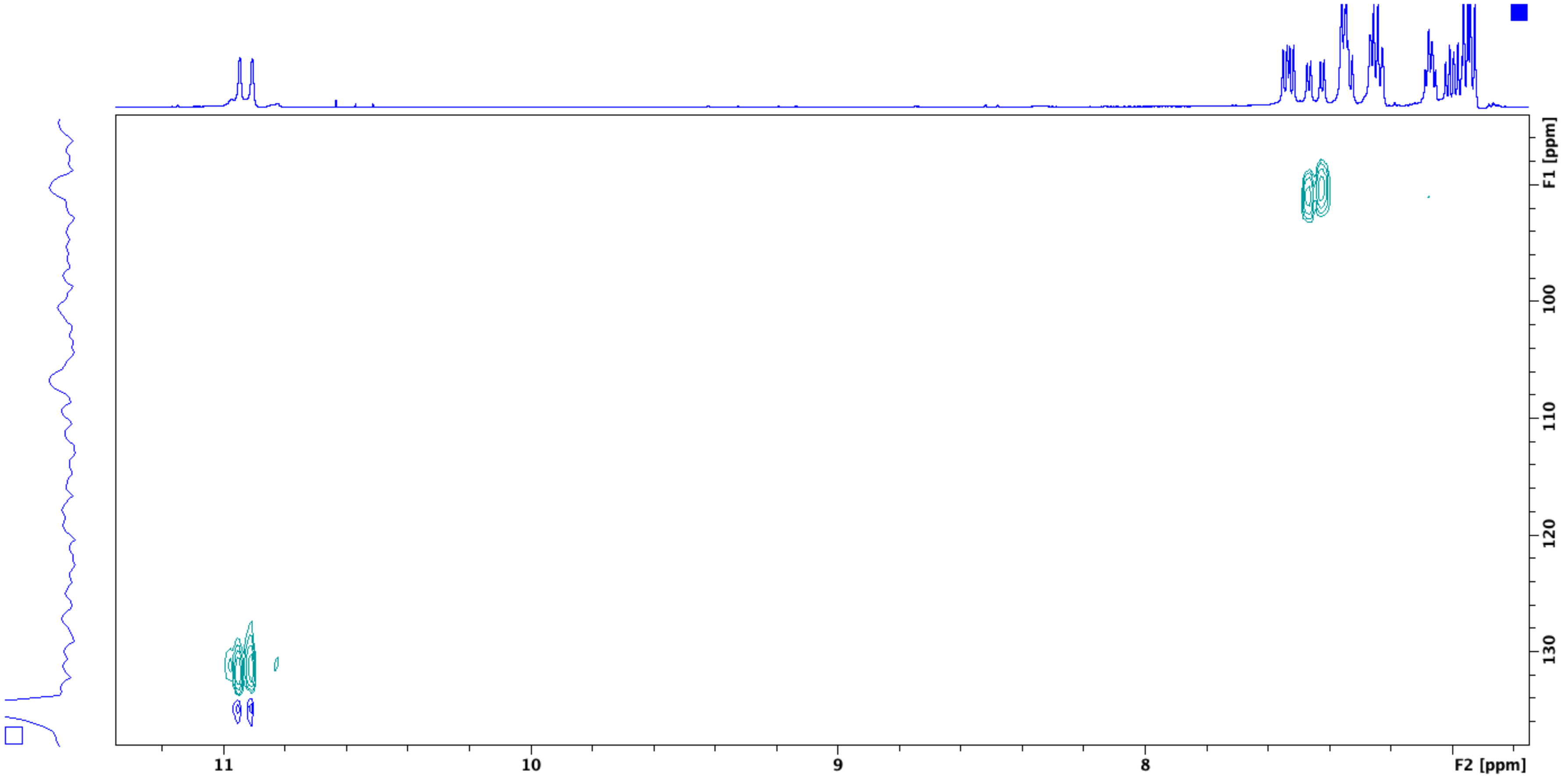

S1.75 ^{15}N HSQC for diastereomer mixture **6** 2-(4-methoxyphenyl)-4-oxo-1-(3,4,5-trimethoxyphenyl)azetidin-3-yl (*tert*-butoxycarbonyl)-*L*-tryptophanate in DMSO-d_6 at 60.8 MHz

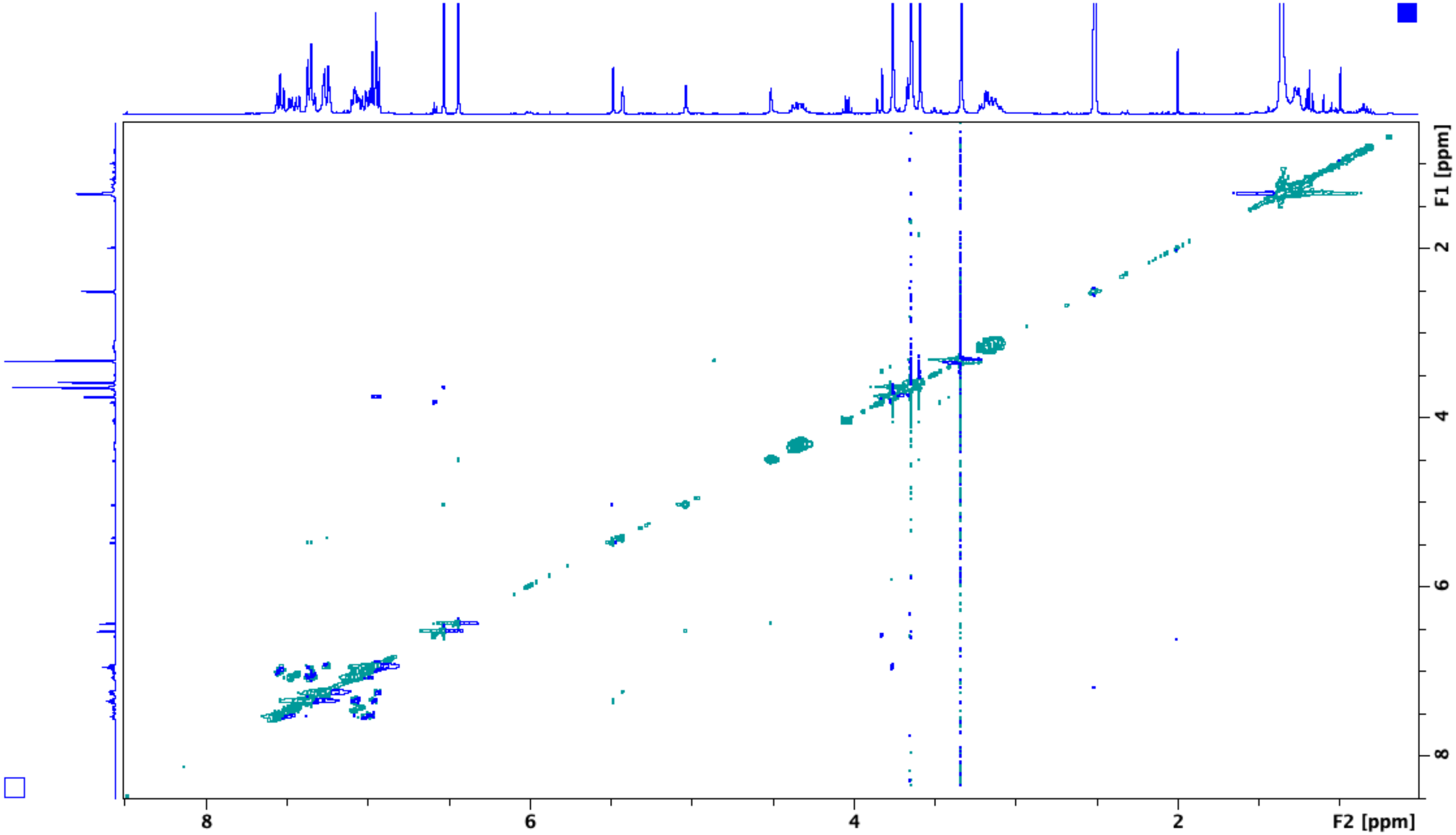

S1.76 2D NOESY of **6** in DMSO-d_6 at 400 MHz at 25 °C.

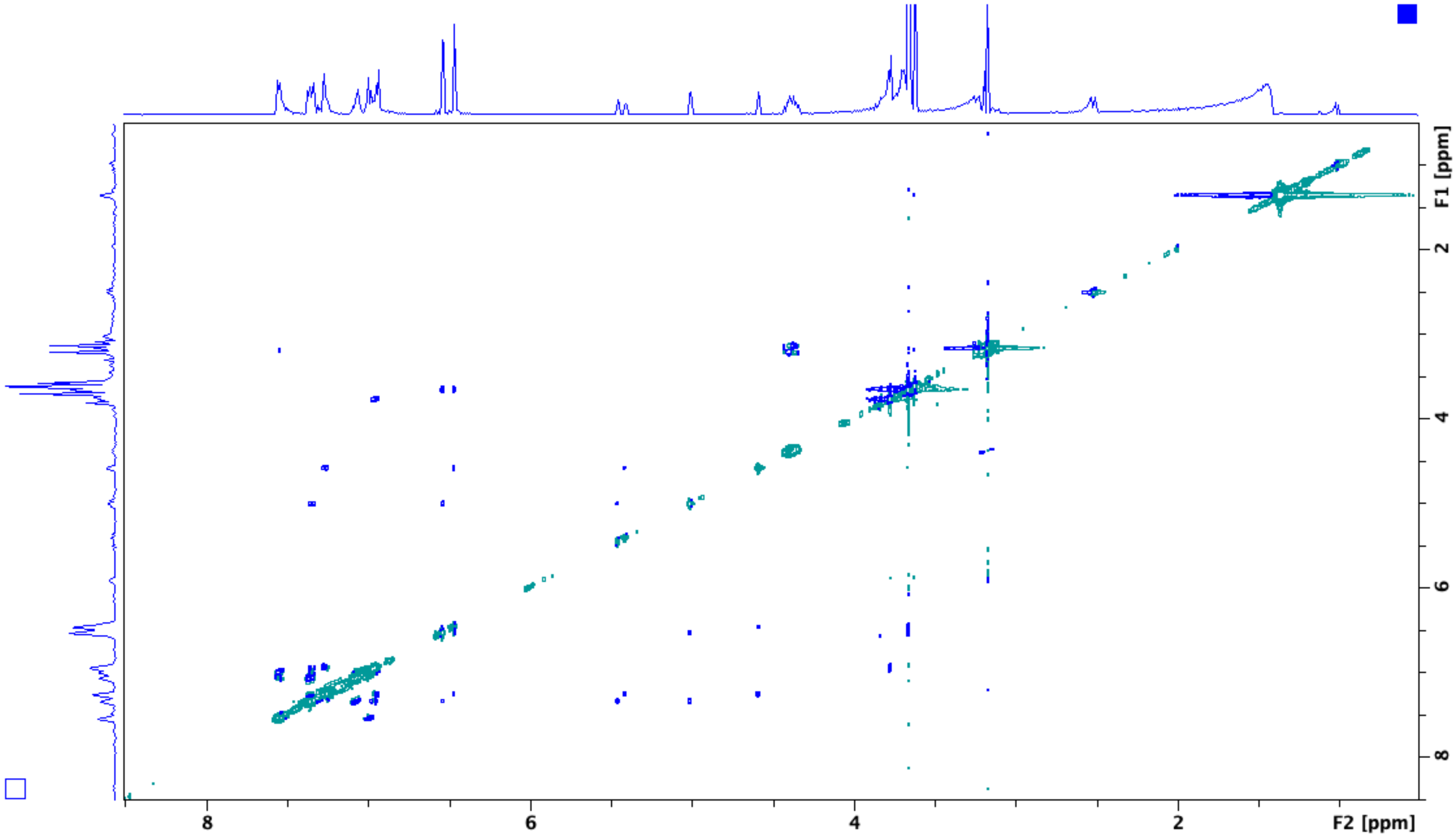

S1.77 VT 2D NOESY of **6** in DMSO-d_6 at 400 MHz at 60 °C. Authors wish to point out that the appearance of the 2D NOESY spectrum is largely identical to 25 °C indicating absence of exchange. Minor coalescence is observed in H_3 and H_4 region (4.9-5.5 ppm)

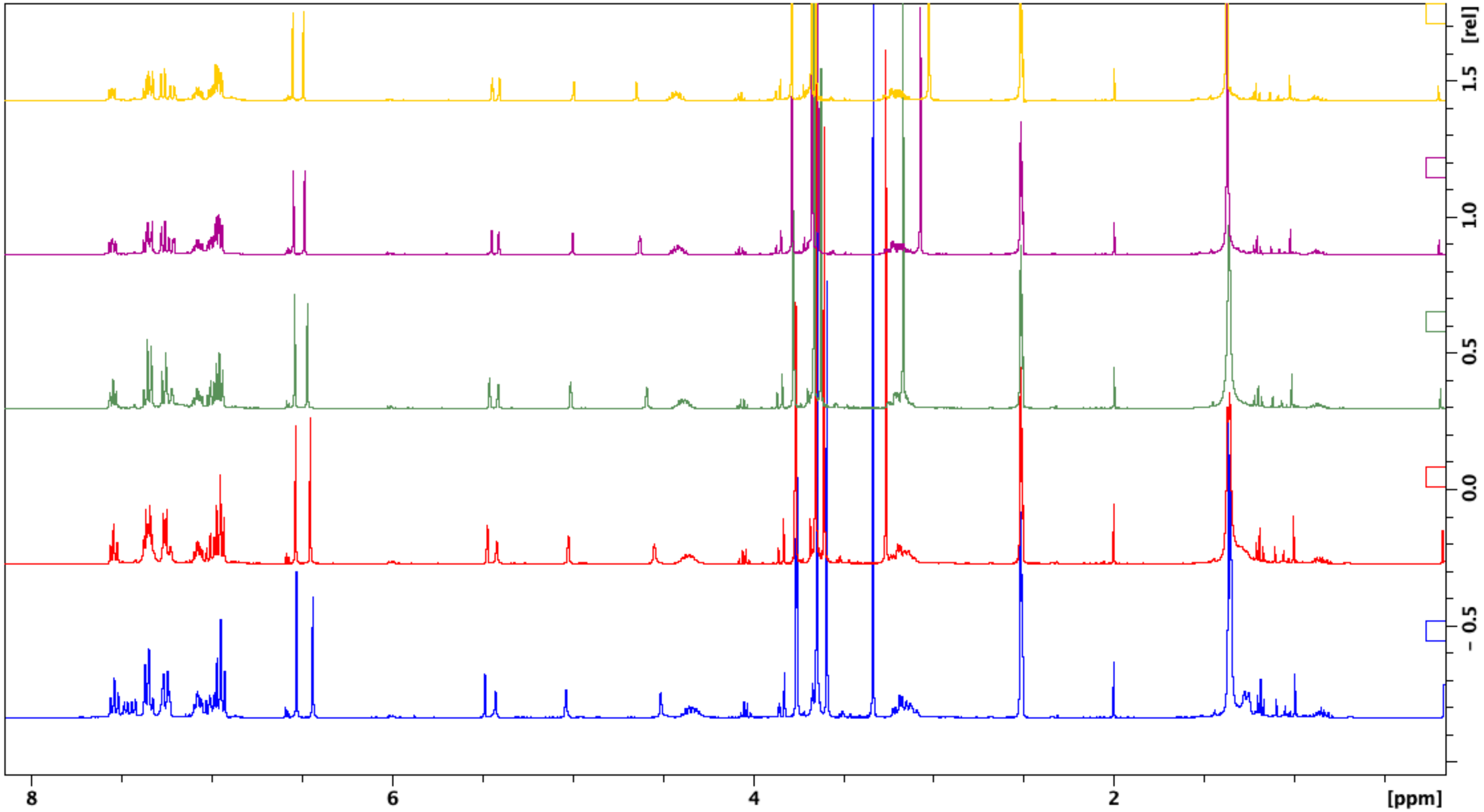

S1.78 VT experiment **6** in DMSO-d_6 at 400 MHz. Blue = 25 °C, red = 40 °C, green = 60°C, purple = 80°C, yellow = 90°C

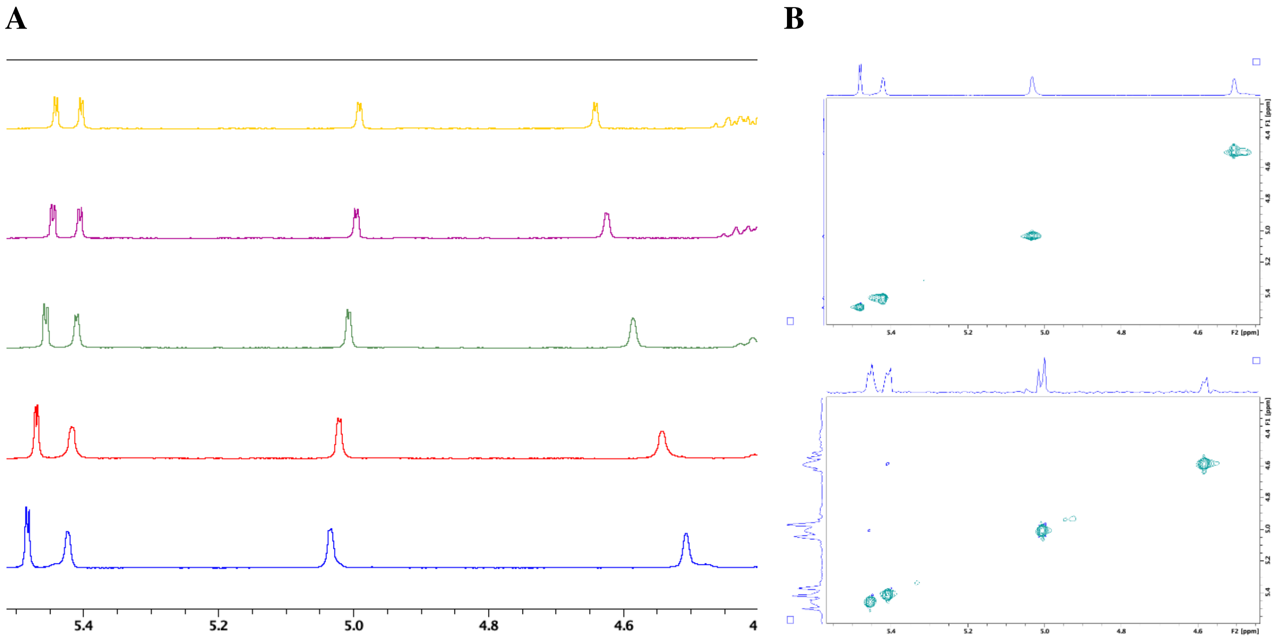

S1.79: VT experiment for **6** in DMSO-d_6. At 400 MHz. **A:** H_3 and H_4 region Blue: 25 °C, Red: 40 °C, Green: 60 °C, Purple: 80 °C, Yellow: 90 °C. **B: Top:** 2D NOESY at 25 °C, 400 MHz for H_3&H_4 region. **Bottom:** 2D NOESY at 60°C, 400 MHz for H_3&H_4 region

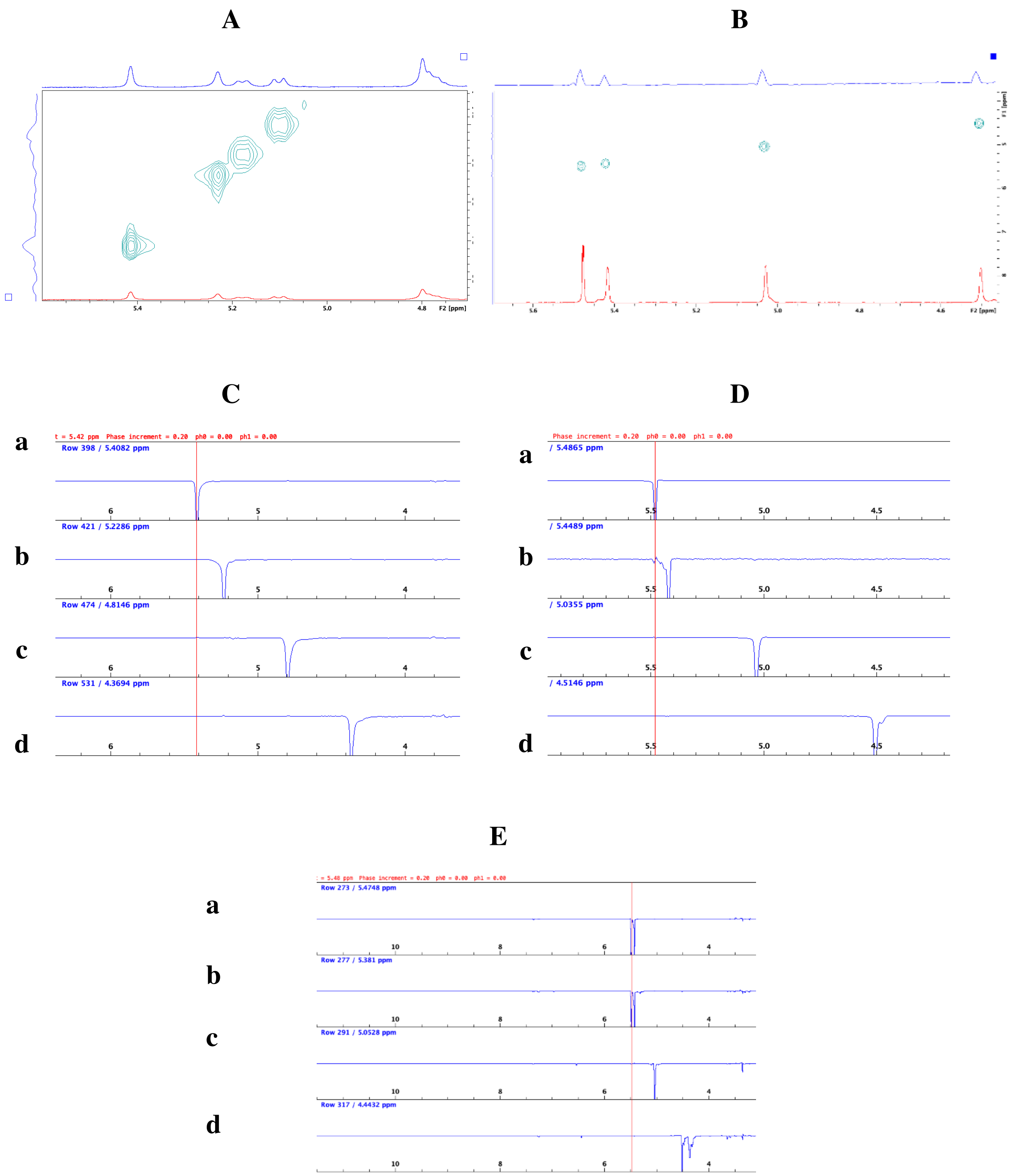

S1.80: A. 2D NOESY of **6** at H_3 and H_4 region, δ 5.05 and 5.45ppm with ^{1}H NMR in $CDCl_3$ at 400 MHz (red) at bottom for reference. **B:** 2D NOESY of **6** at H_3 and H_4 region, δ 4.44 and 5.65 ppm, with ^{1}H NMR in DMSO-d_6 at 600 MHz (red) at bottom for reference **C:** Manual phasing of 2D NOESY spectrum for H_3 and H_4 region for **6** at 400 MHz in $CDCl_3$ **a:** H_3 **6DS1** **b:** H_3 **6DS2**, **c**: H_4 **6DS1** **d:** H_4 **6DS2. D:** Manual phasing of 2D NOESY spectrum for for **6** at 400 MHz in DMSO-d_6 , **a**: H_3 **6DS1** **b**: H_3 **6DS2**, **c:** H_4 **6DS1** **d:** H_4 **E:** Manual phasing of 2D NOESY spectrum for **6** at higher magnetic field strength of 600 MHz in DMSO-d_6, **a:** H_3 **6DS1**, **b**: H_3 **6DS2**, **c:** H_4 **6DS1**, **d:** H_4 **6DS2**

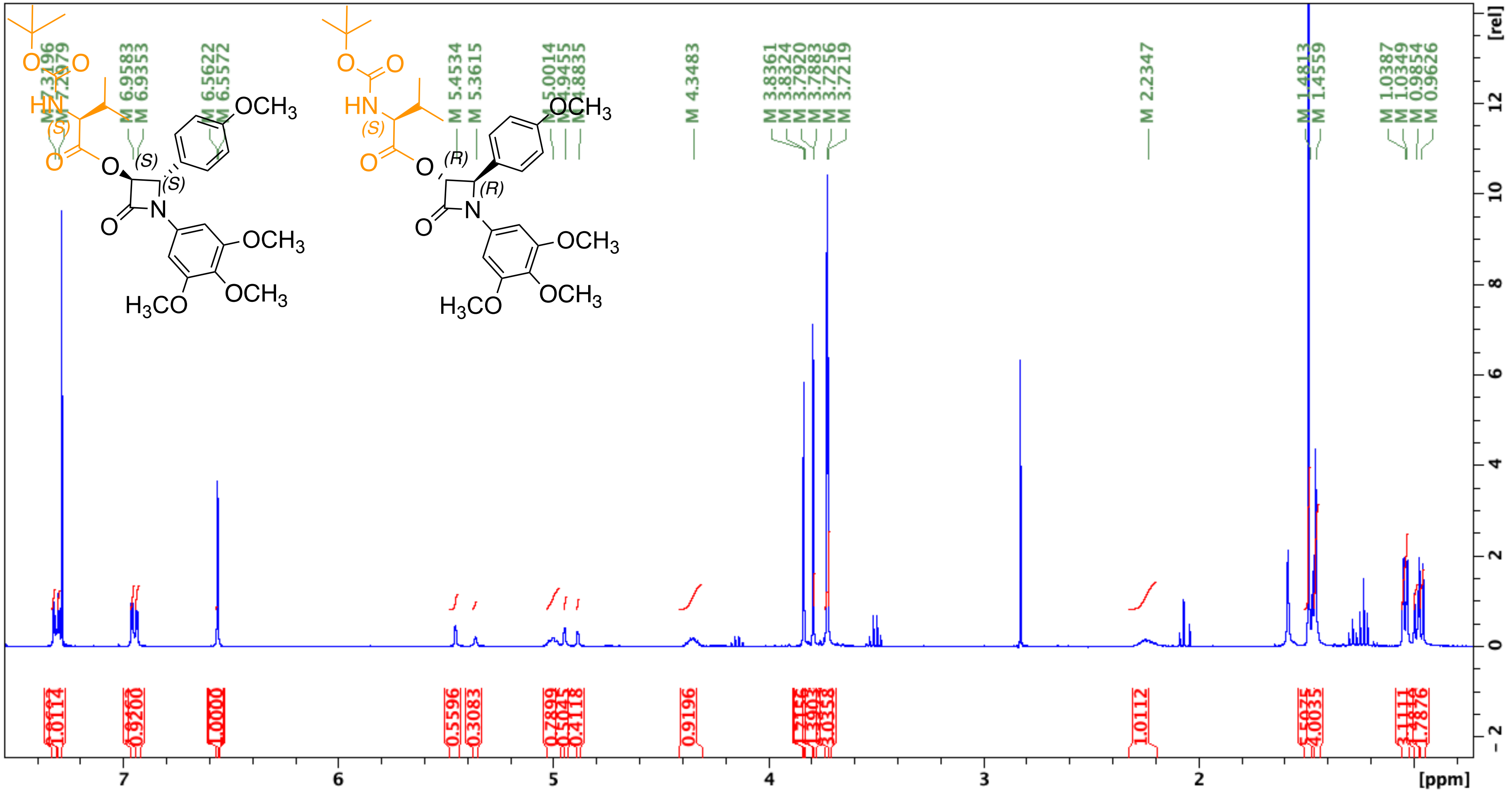

S1.1 [1] H NMR for **7** in $CDCl_3$ at 400 MHz. **Purity RP-HPLC:** 96%

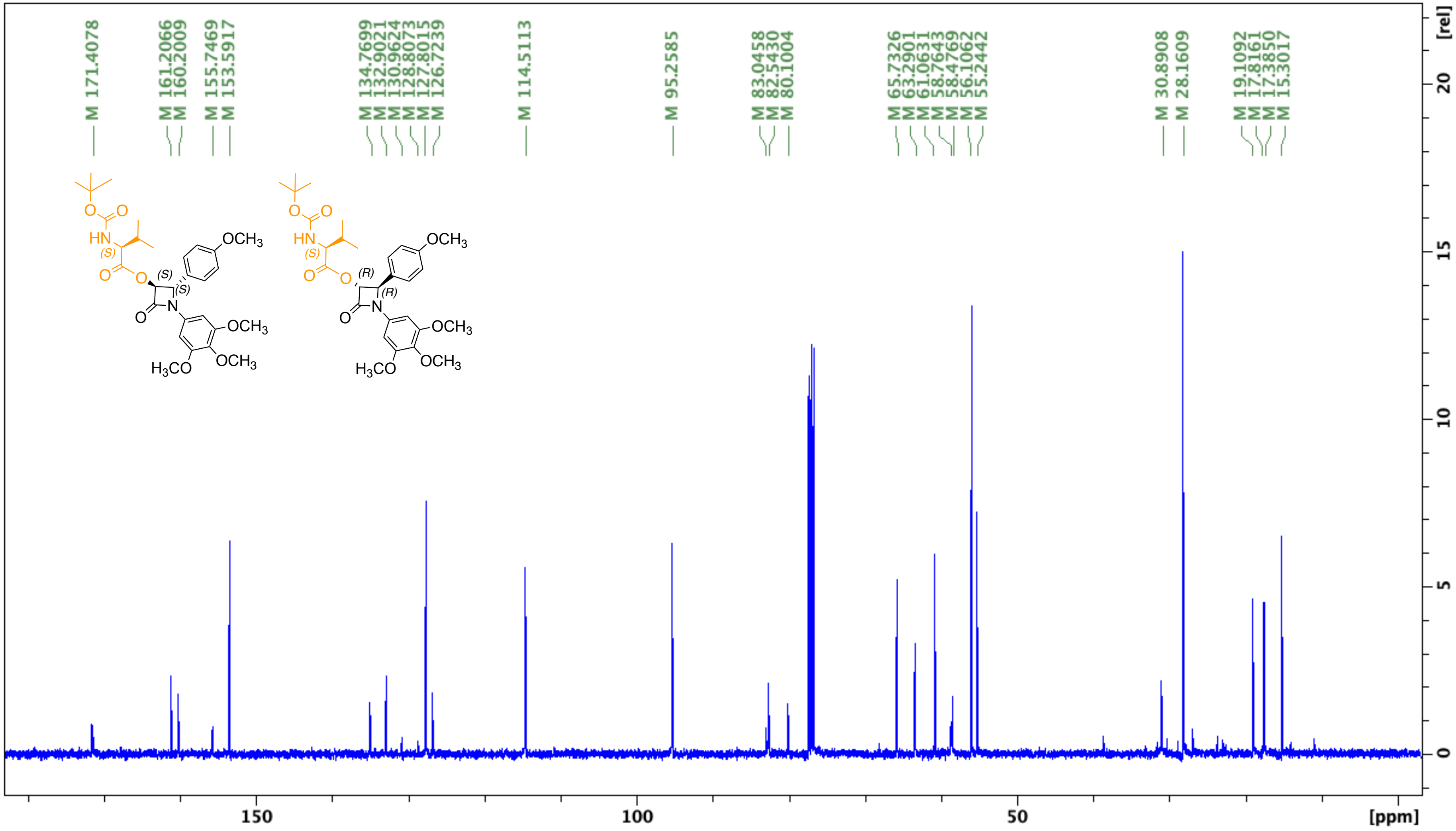

S1.82 ^{13}C NMR for **7** 2-(4-methoxyphenyl)-4-oxo-1-(3,4,5-trimethoxyphenyl)azetidin-3-yl (*tert*-butoxycarbonyl)-*L*-valinate in $CDCl_3$ at 100 MHz. **Purity RP-HPLC:** 96%

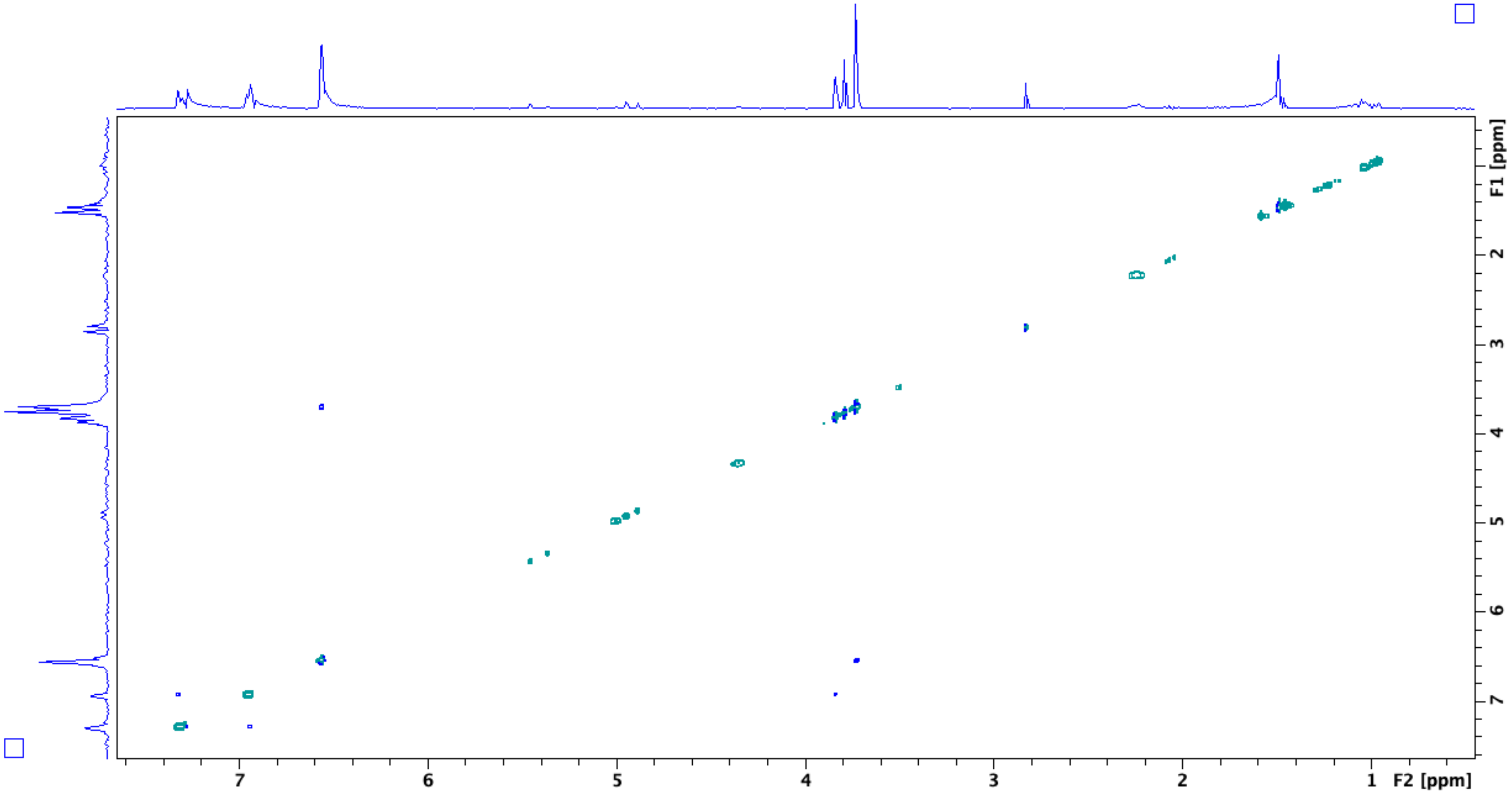

S1.83. 2D NOESY for **7** in $CDCl_3$ at 400 MHz at 25 °C. **Purity RP-HPLC:** 96%

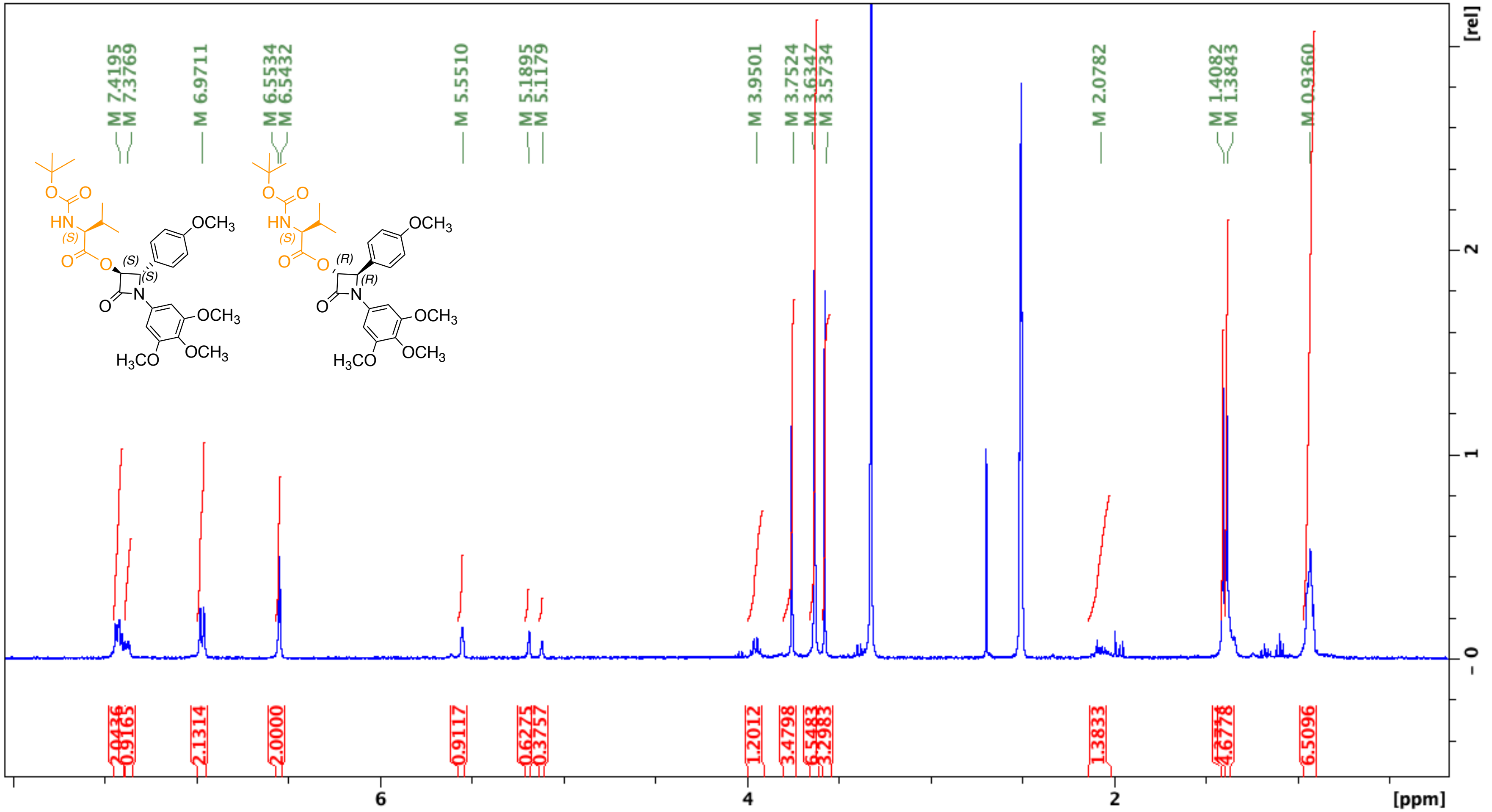

S1.84[1] H NMR for **7** in DMSO-d_6 at 400 MHz. **Purity RP-HPLC:** 96%

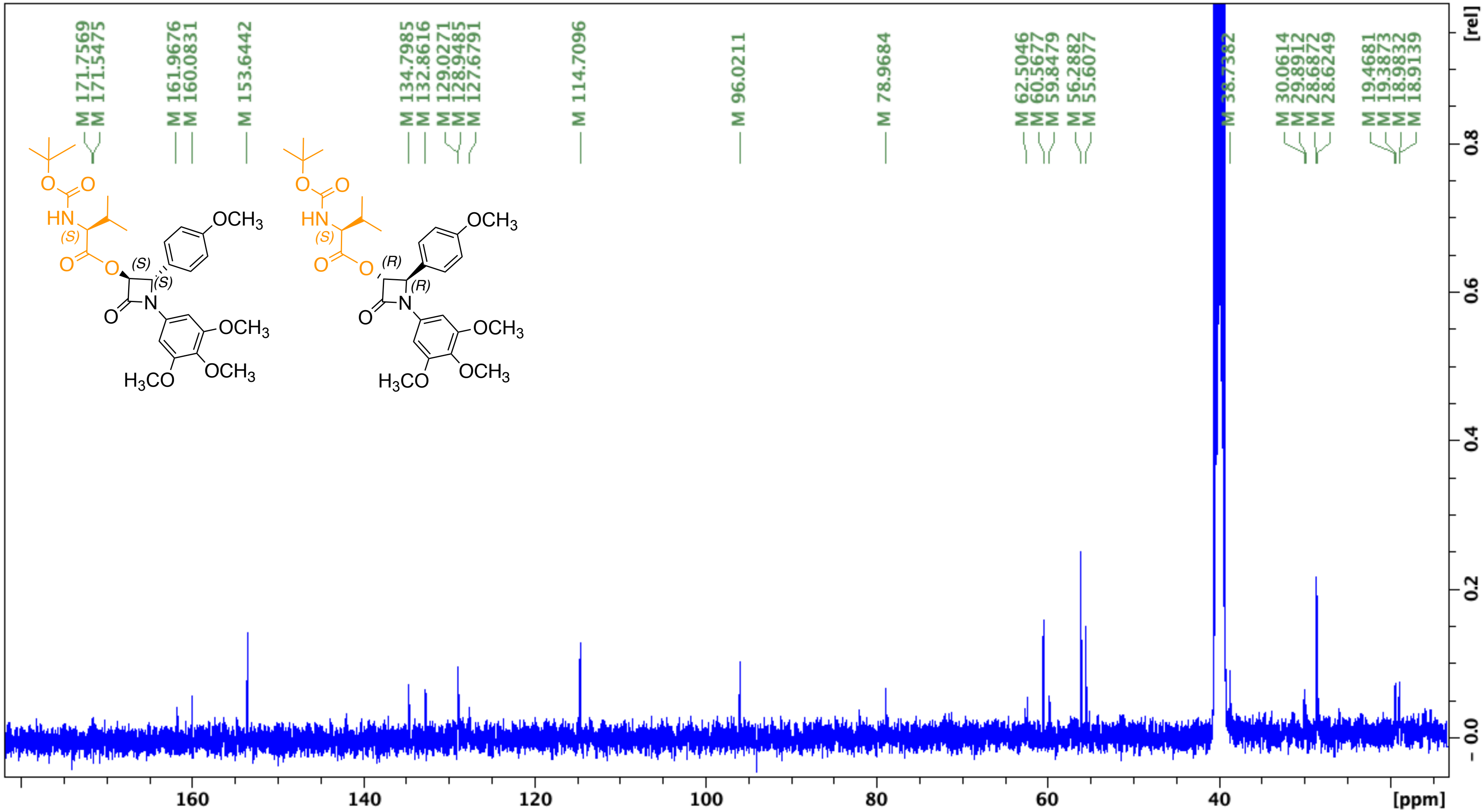

S1.85 ^{13}C NMR for **7** in DMSO-d_6 at 100 MHz. **Purity RP-HPLC:** 96%

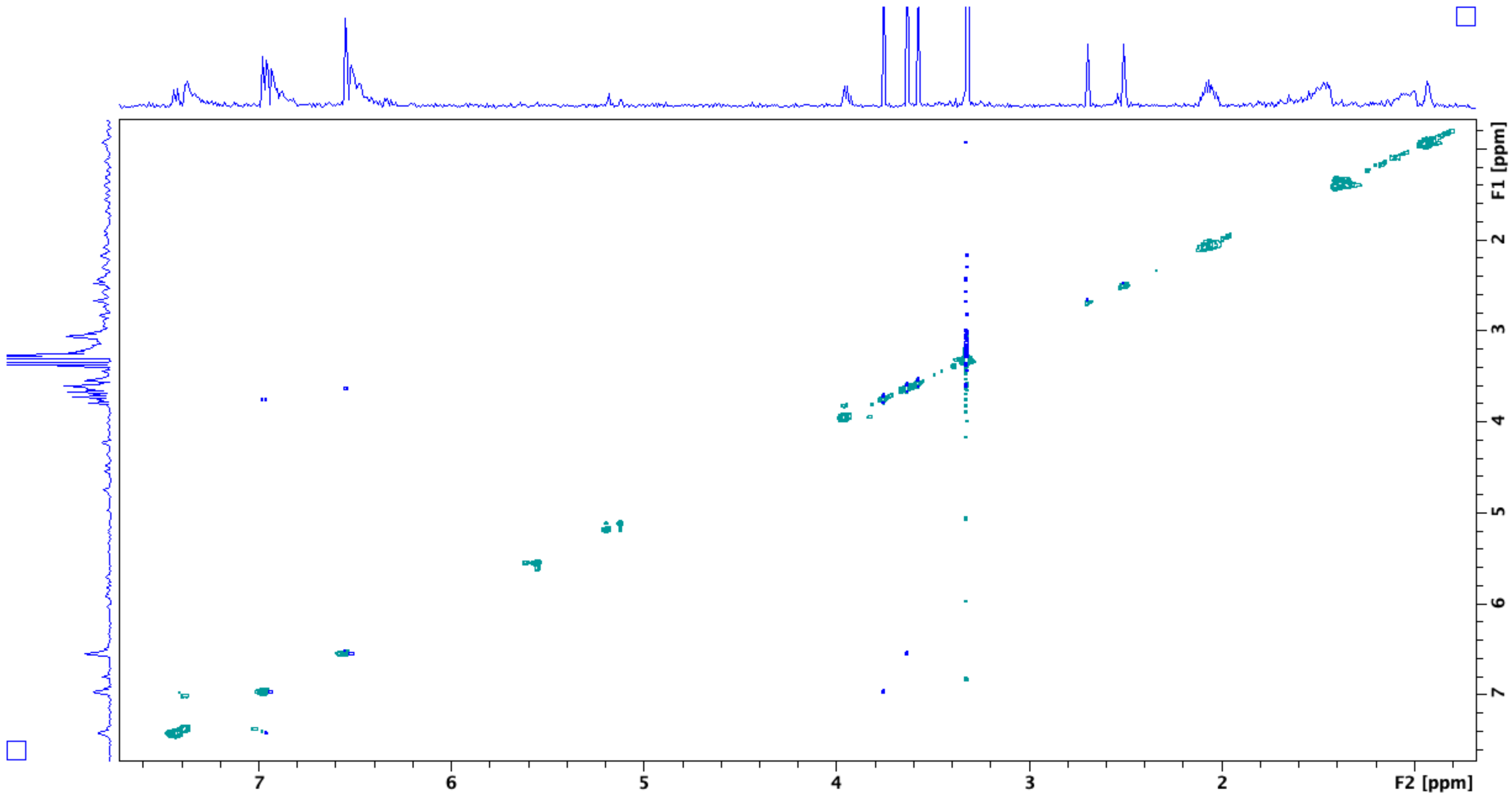

S1.86 2D NOESY for **7** in DMSO-d_6 at 400 MHz at 25 °C

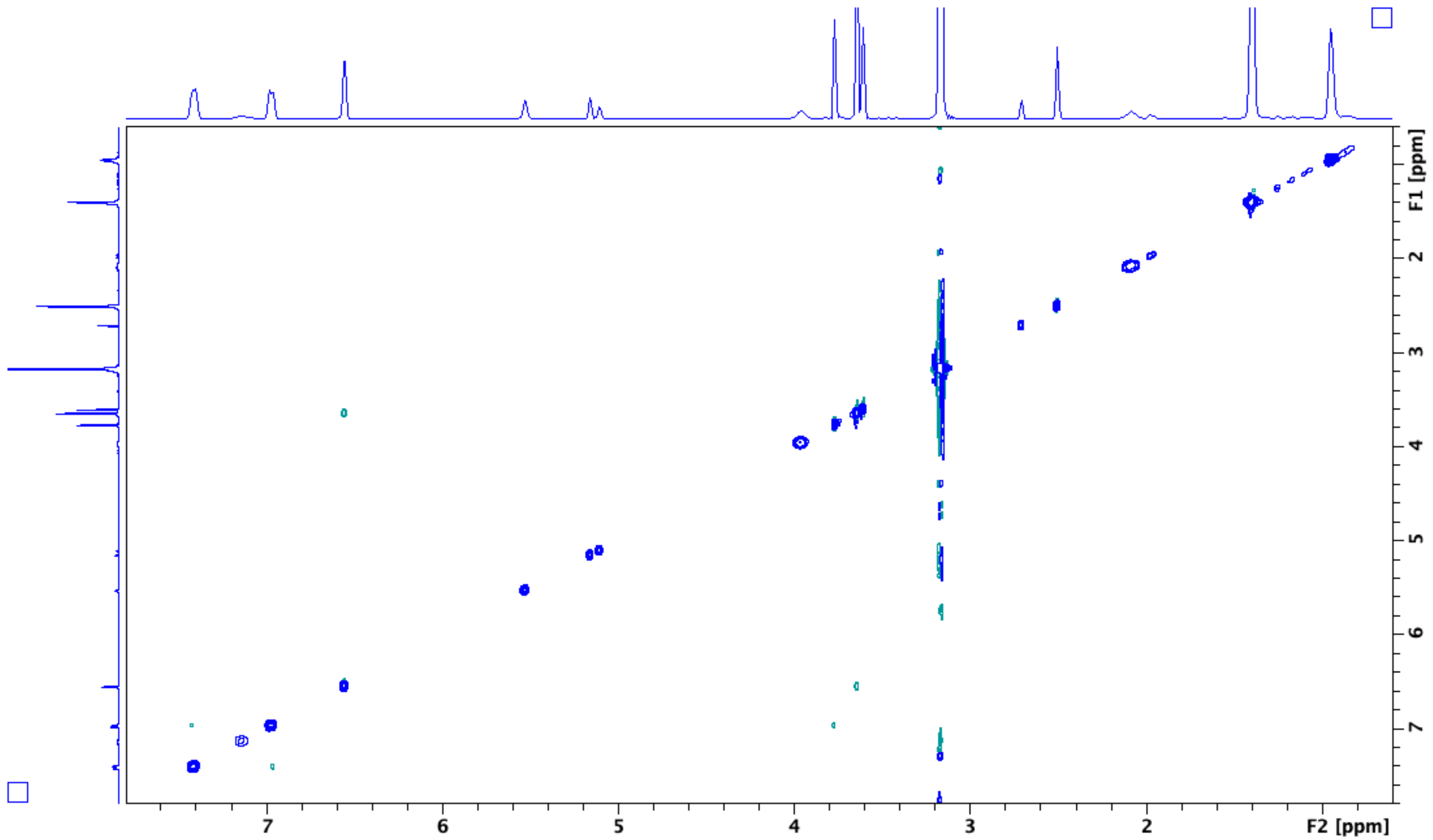

S1.87 2D NOESY for **7** in DMSO-d_6 at 400 MHz at 65 °C. Minor coalescence observed in H_3 and H_4 region at 65 °C compared to 25 °C (**SI.86)** from 5-5.5 ppm.

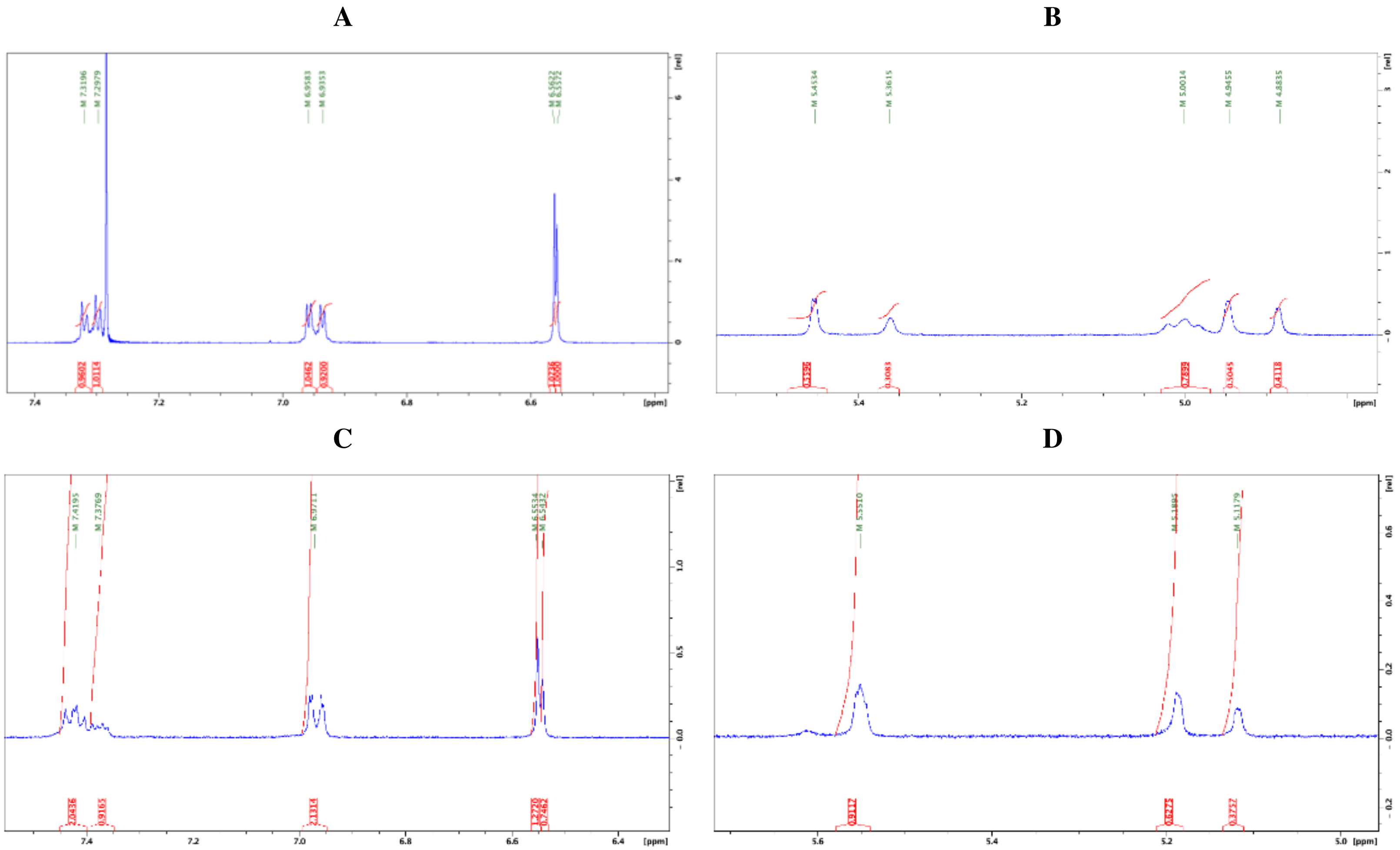

S1.88 [1] H NMR of **7** **A:** B ring region in $CDCl_3$ at 400 MHz. **B:** H_3 and H_4 region in $CDCl_3$ at 400 MHz. **C:** B ring region in DMSO-d_6 at 400 MHz. **D:** H_3 and H_4 region in DMSO-d_6 at 400 MHz

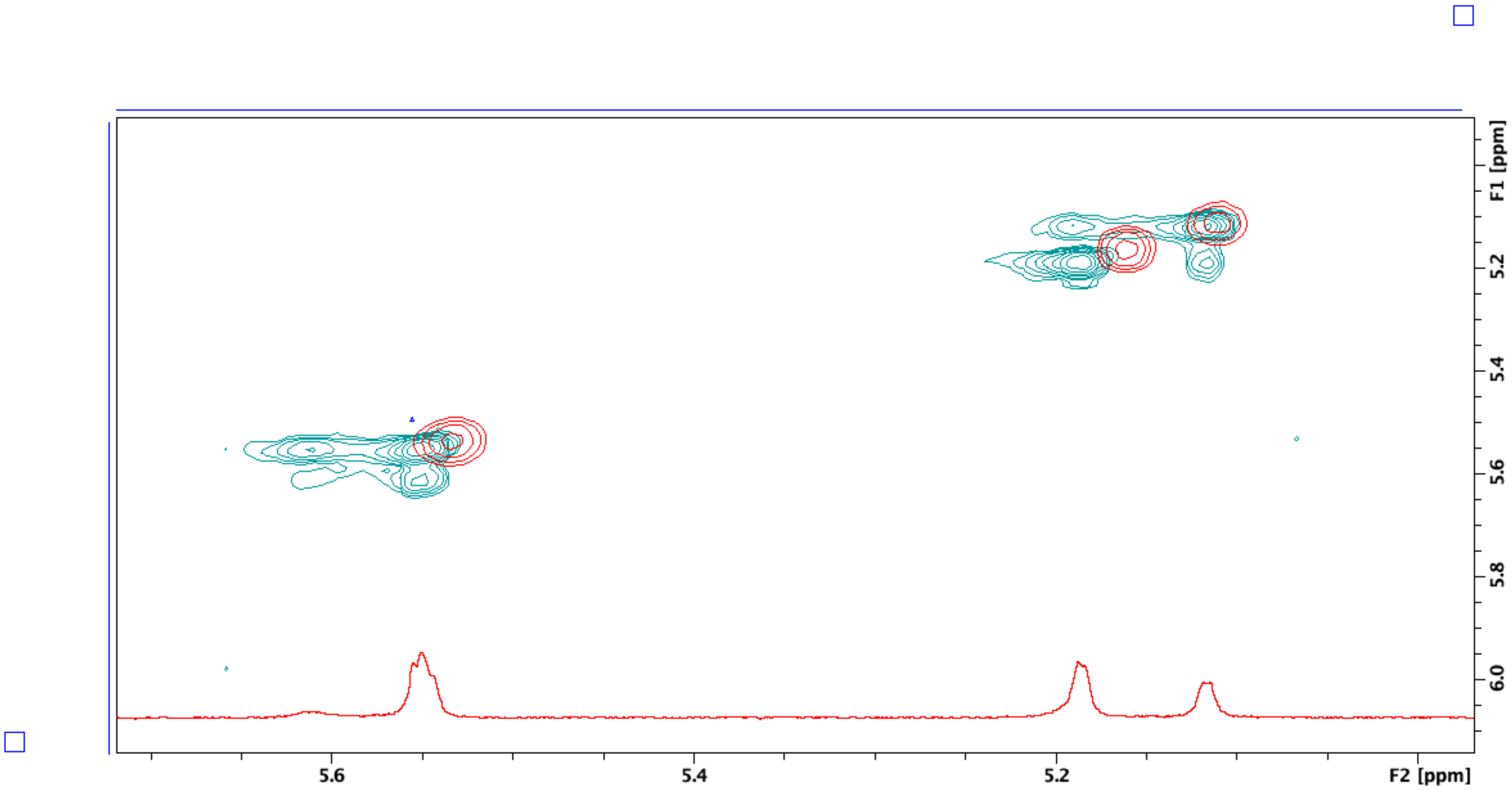

S1.89 2D NOESY studies for **7** at 25°C (green) and 65 °C (red) in DMSO-d_6. Left: B ring region. Right: H_3 & H_4 region. [1] H NMR spectrum illustrated in red (bottom) for reference.

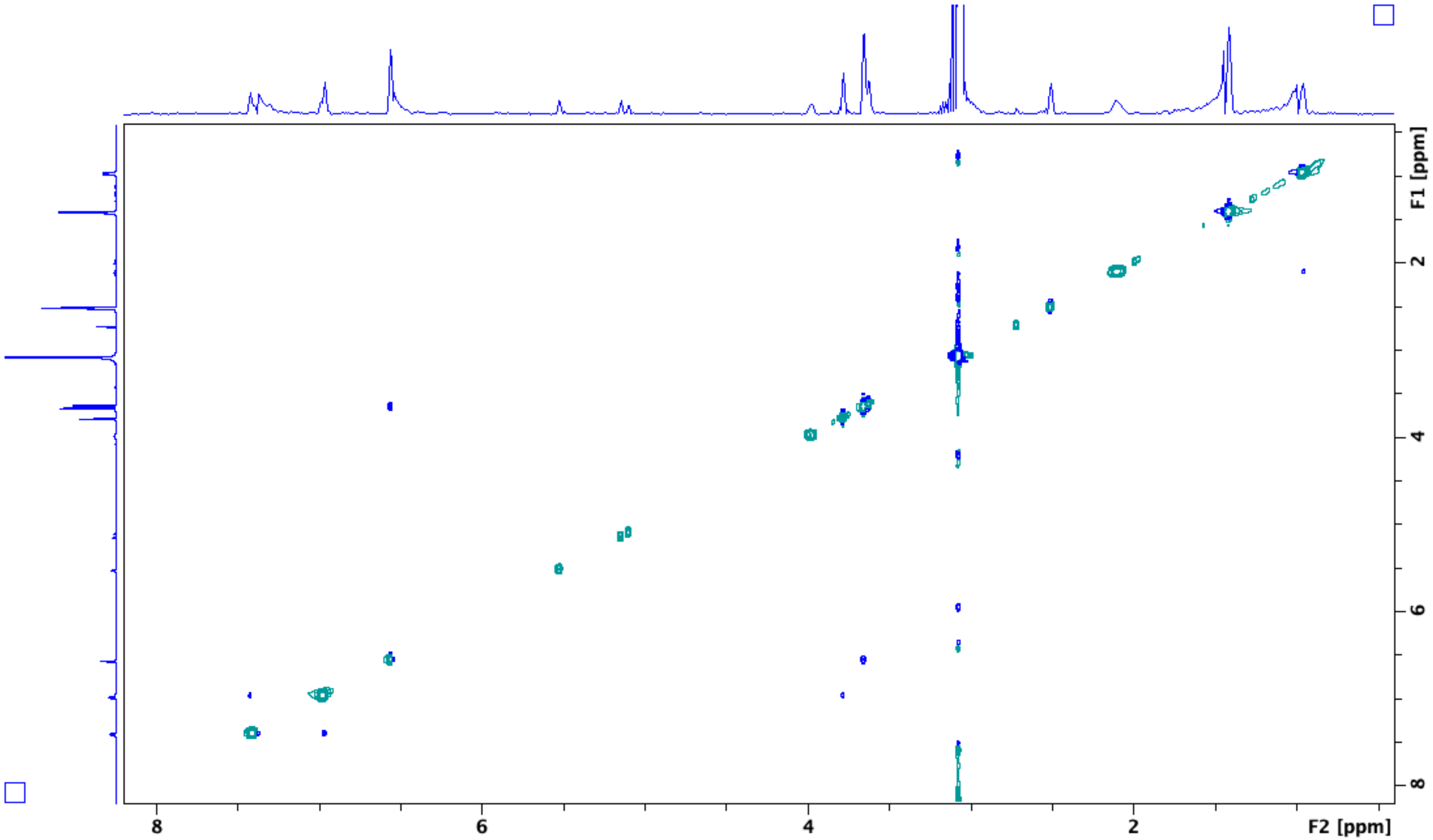

S1.90 2D NOESY for **7** in DMSO-d_6 at 400 MHz at 85 °C. Minor coalescence observed in H_3 and H_4 region at 85 °C compared to 25 °C (**SI.86**) from 5-5.5 ppm. (*spectrum reprocessed with 1 H NMR added onto F2 axis)*

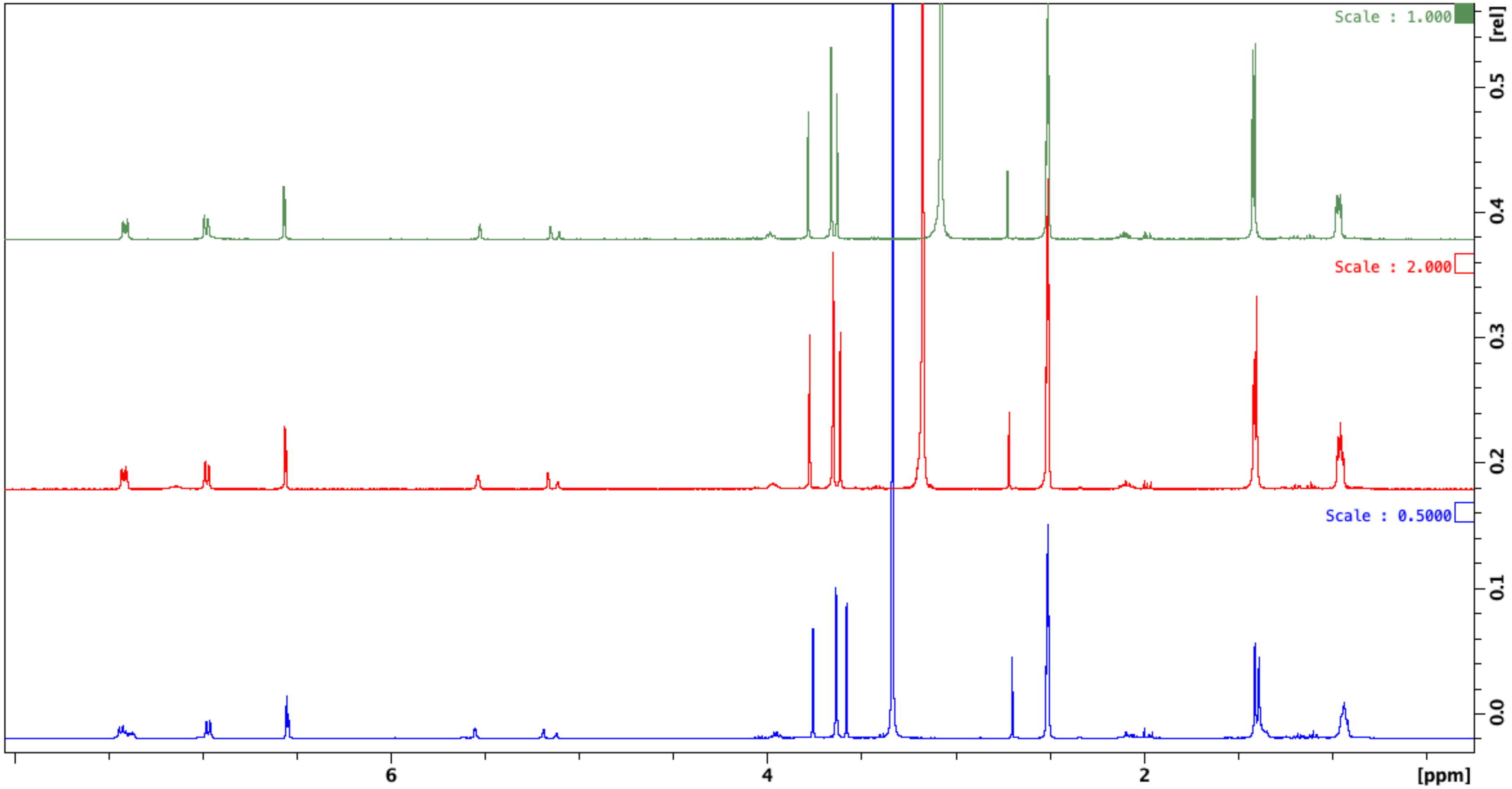

S1.91 VT experiment for diastereomer mixture **7** 2-(4-methoxyphenyl)-4-oxo-1-(3,4,5-trimethoxyphenyl)azetidin-3-yl (*tert*-butoxycarbonyl)-*L*-valinate in DMSO-d_6 at 400 MHz. Blue = 25 °C, red = 60 °C, green = 80°C

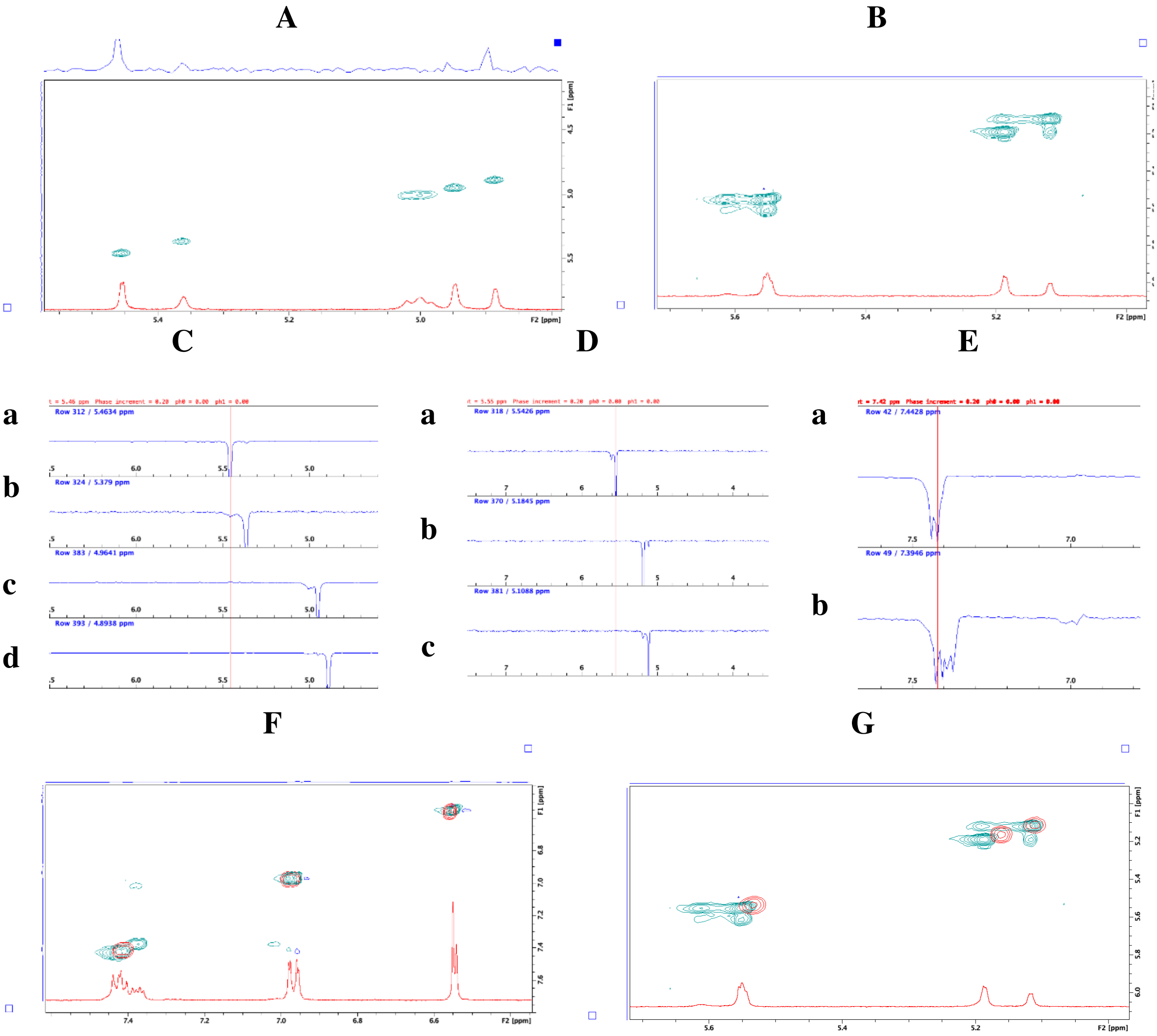

S1:92: 2D NOESY for **7** at 400 MHz, 25 °C in H_3 and H_4 region. [1] H NMR of **7** illustrated in red for reference. **A**: $CDCl_3$ **B**. DMSO-d_6 **C:** Manual phasing of 2D NOESY spectrum of **7** in $CDCl_3$ at 400 MHz for H_3 and H_4 region . **a:** δ 5.45 ppm **b:** δ 5.36 ppm **c:** δ 4.94 ppm **d:** δ 4.98 ppm **D:** Manual phasing of 2D NOESY spectrum for H_3 and H_4 of **7** in DMSO-d_6 **a**: H_3 for **7DS1** and **7DS2** superimposed **b**: H_4 for **7DS1** **c:** H_4 for **7DS2** **E**: Manual phasing of 2D NOESY spectrum for B ring doublet region in DMSO-d_6 at 400 MHz **a:** δ 7.44 ppm and **b:** δ 7.39 ppm. **F:** 2D NOESY from VT studies for **7** at 25°C (green) and 80 °C (red) in DMSO-d_6 in B ring region. **E:** 2D NOESY from VT studies for **7** at 25°C (green) and 80 °C (red) in DMSO-d_6 in H_3 and H_4 region

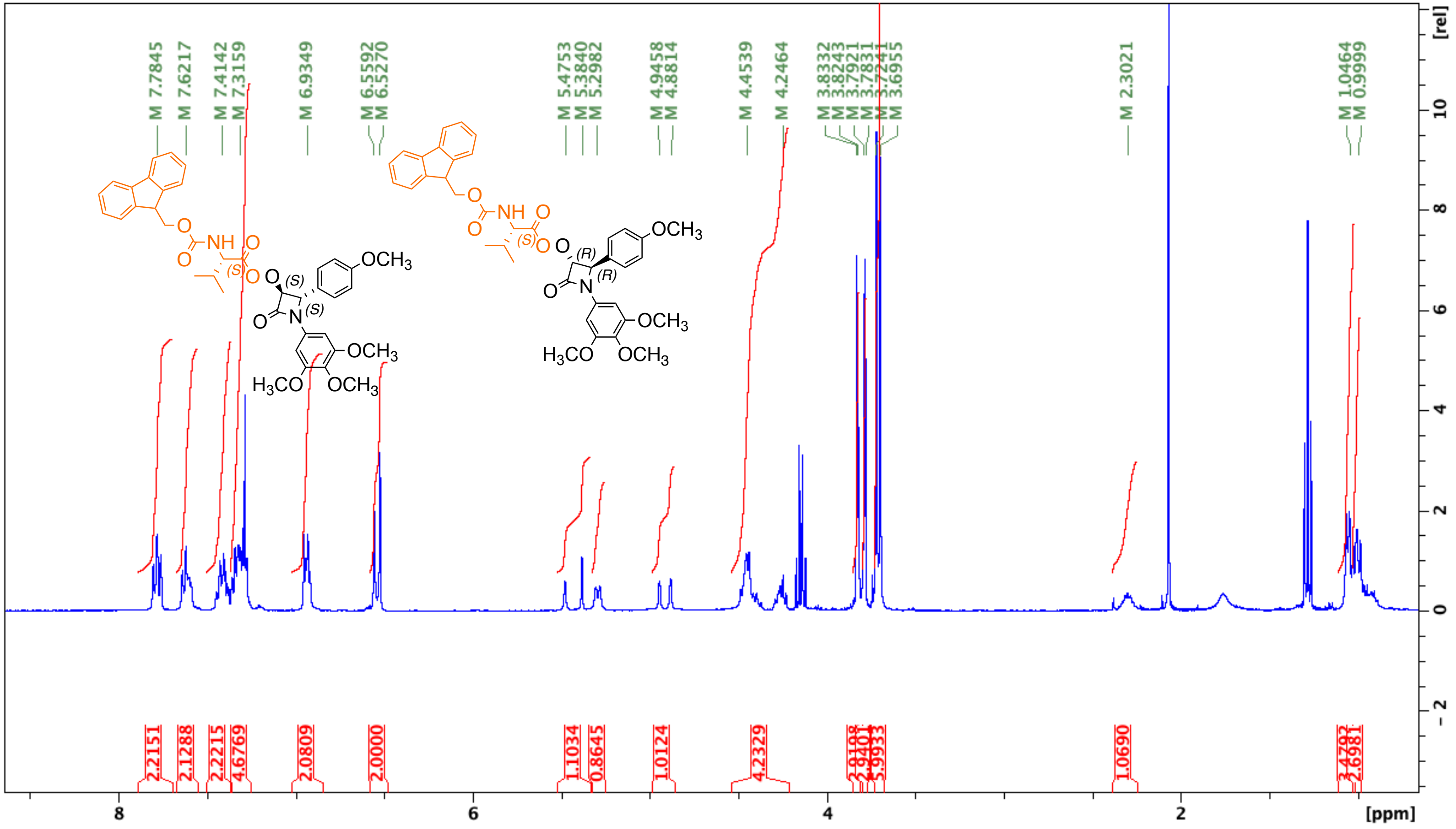

S1.93 ^{1}H NMR of **8** in $CDCl_3$ at 400 MHz. **Purity RP-HPLC:** 70 % (Impurities observed from 0-2 ppm as unlabelled resonances)

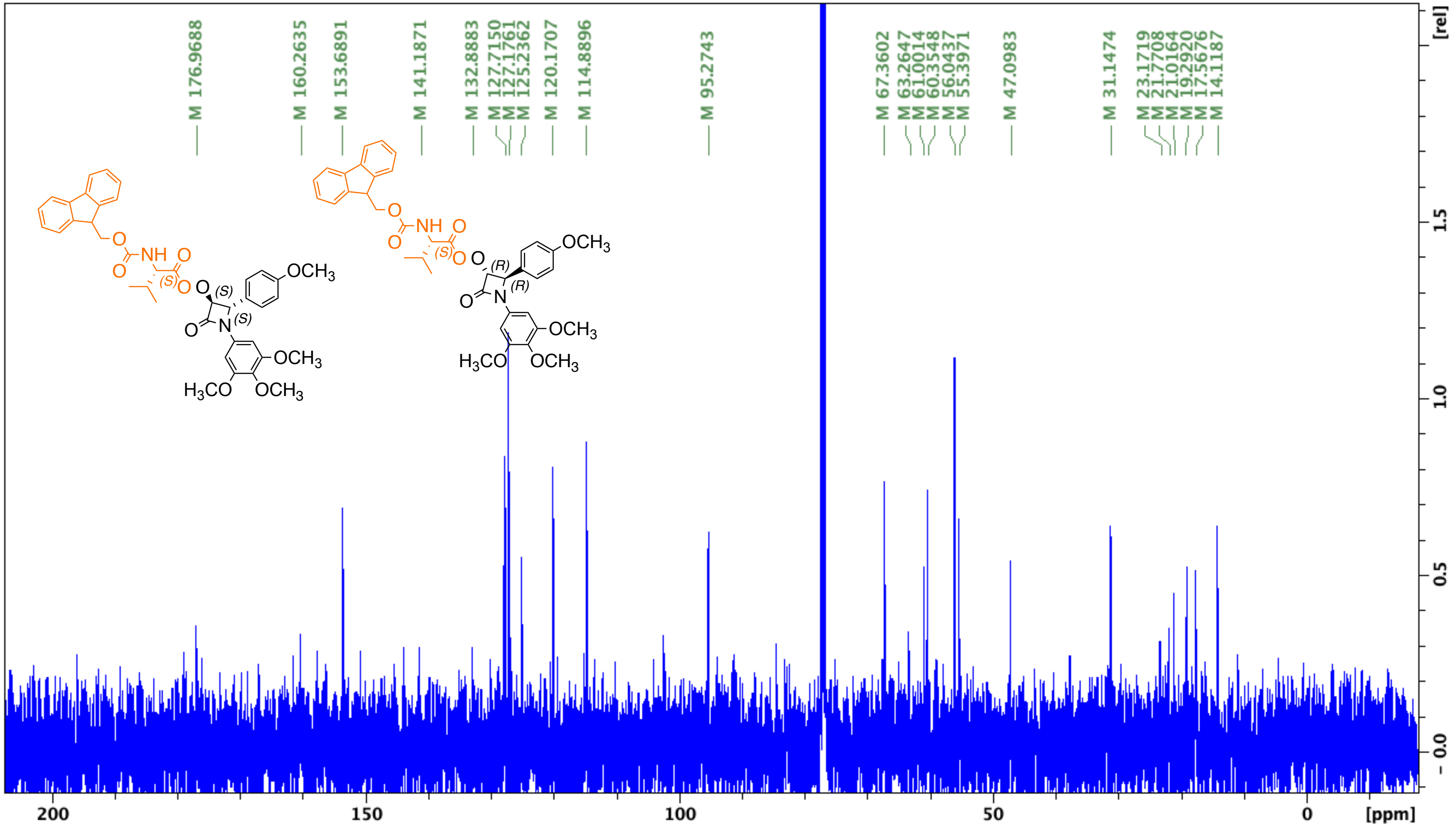

S1.94 ^{13}C NMR of **8** in $CDCl_3$ at 100 MHz. **Purity RP-HPLC:** 70 %

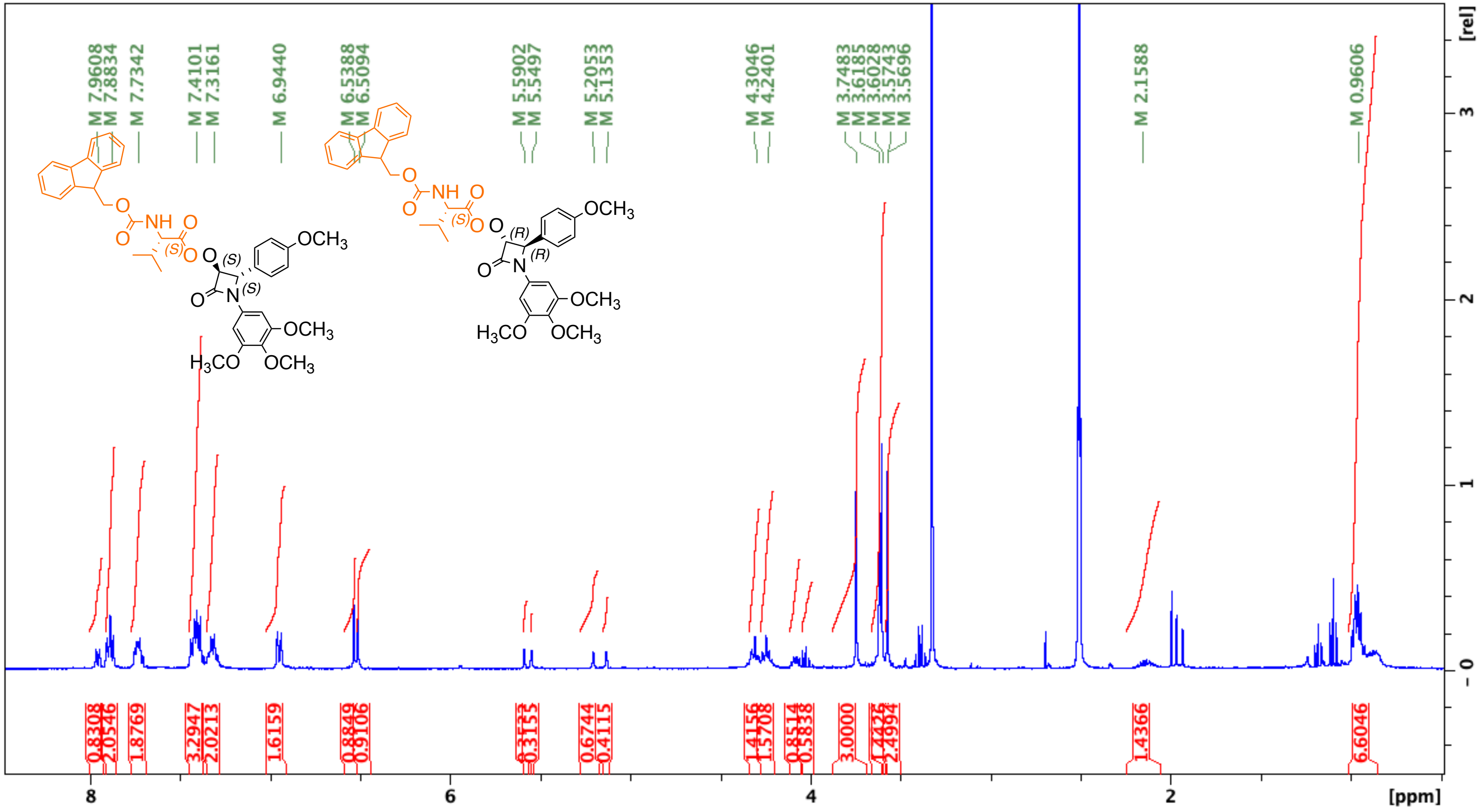

S1.95 [1] H NMR of **8** in DMSO-d_6 at 400 MHz. **Purity RP-HPLC:** 70 %. Impurities observed between 1-2 ppm (unlabelled peaks)

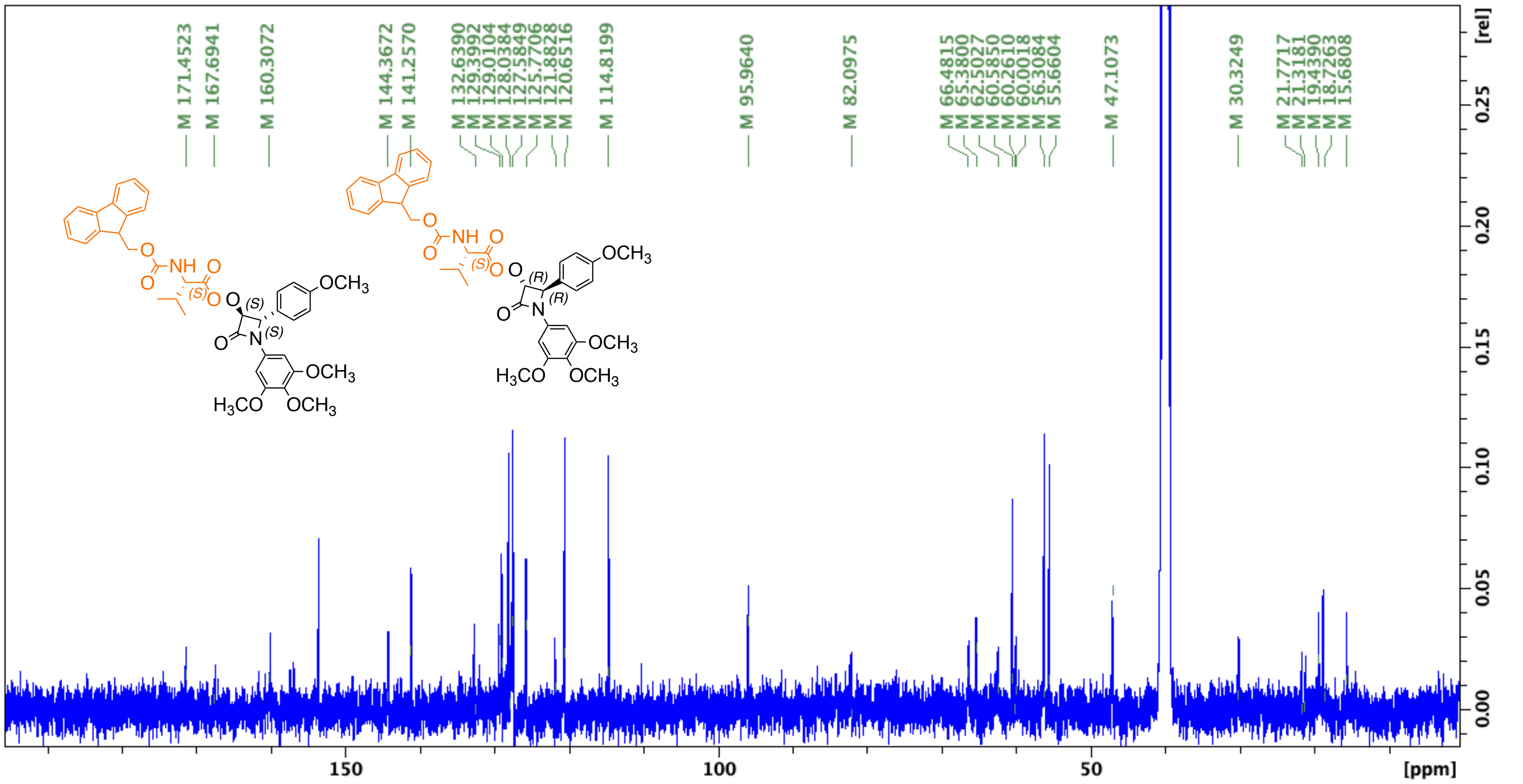

S1.96 [13] C NMR of **8** in DMSO-d_6 at 100 MHz. **Purity RP-HPLC:** 70 %

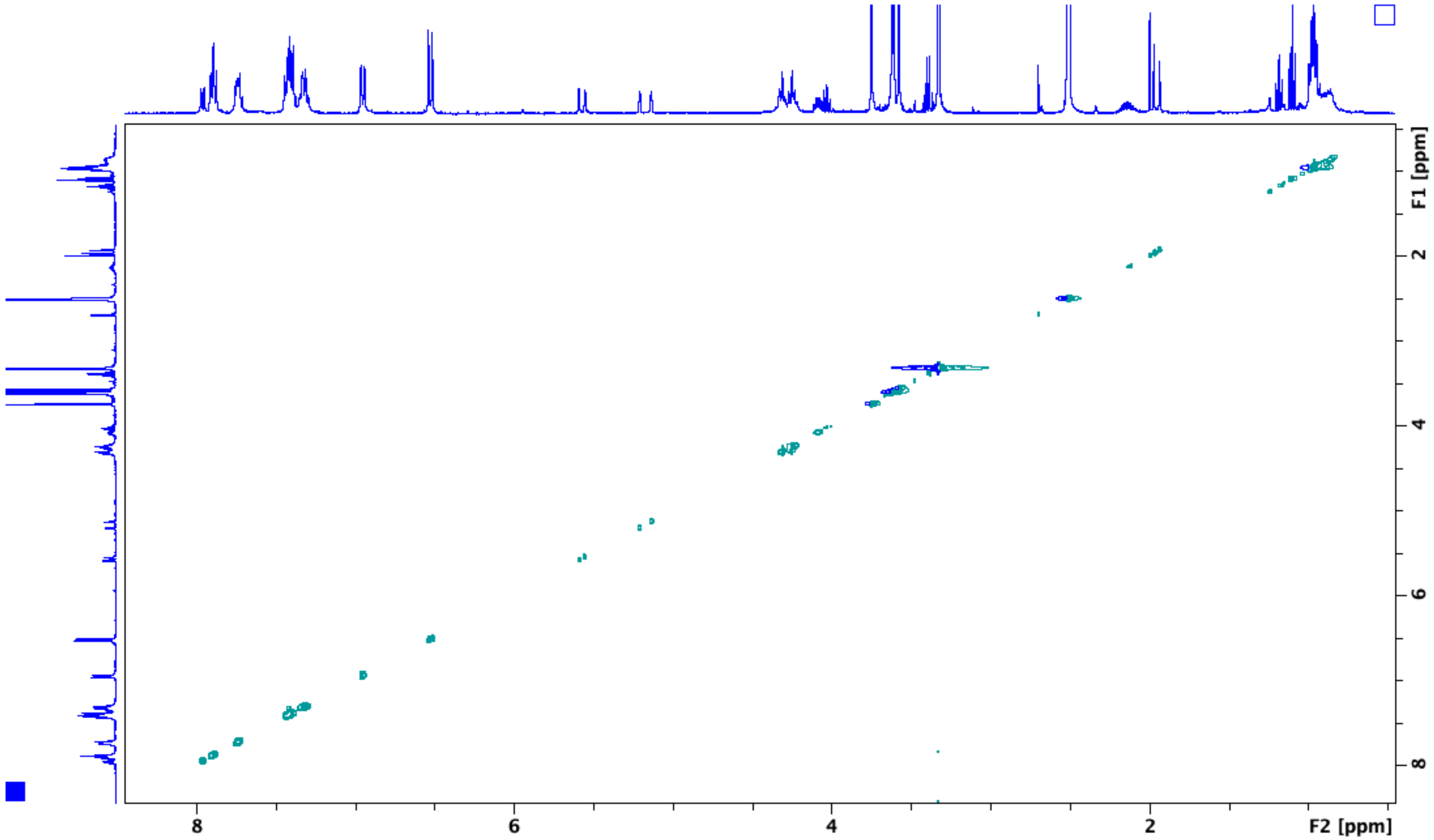

S1.97 2D NOESY of **8** in DMSO-d_6 at 100 MHz. No evidence of cross peaks observed.

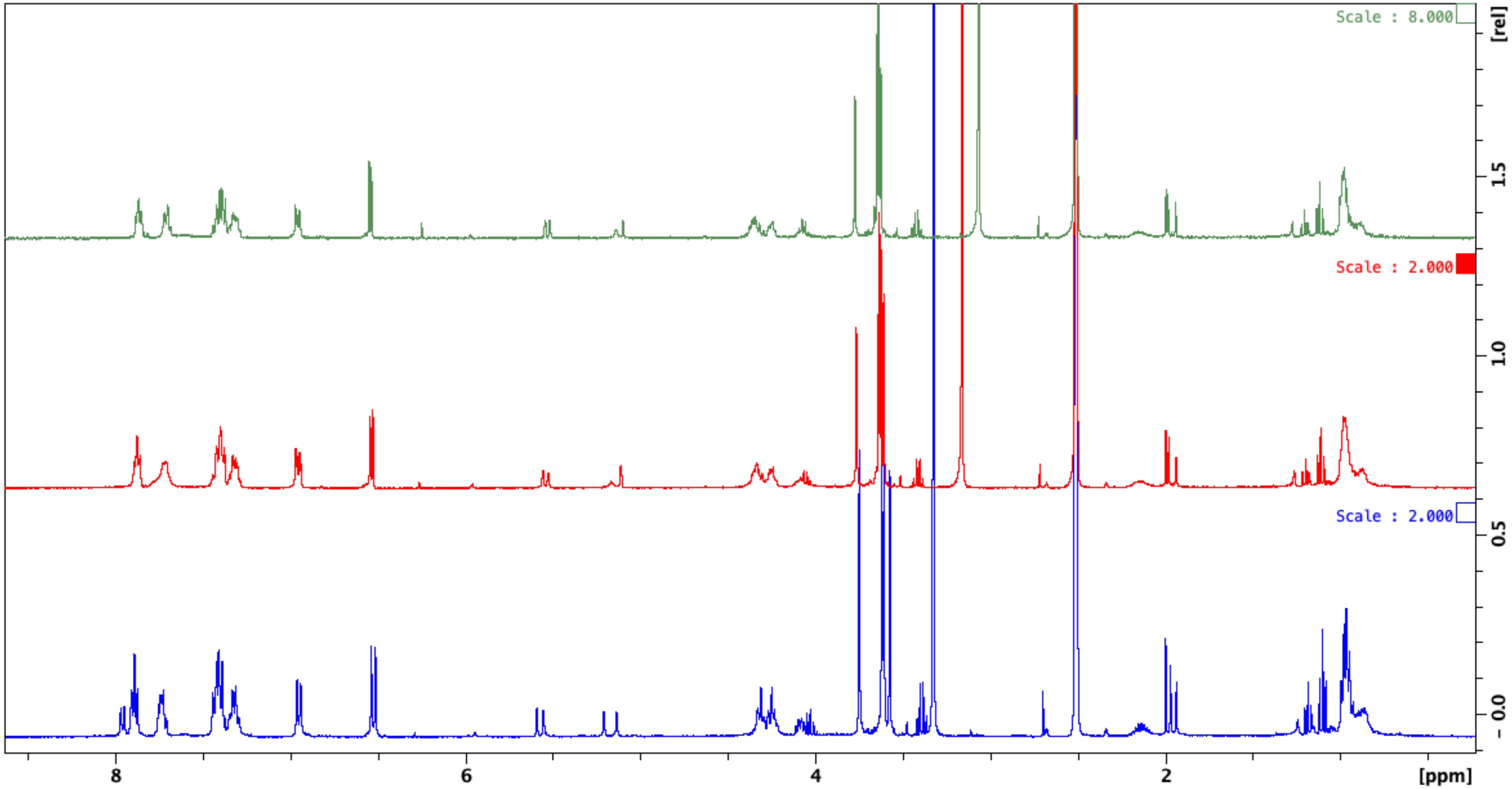

S1.98 VT of **8** in DMSO-d_6 at 400 MHz Blue = 25 °C, red = 60 °C, green = 80°C

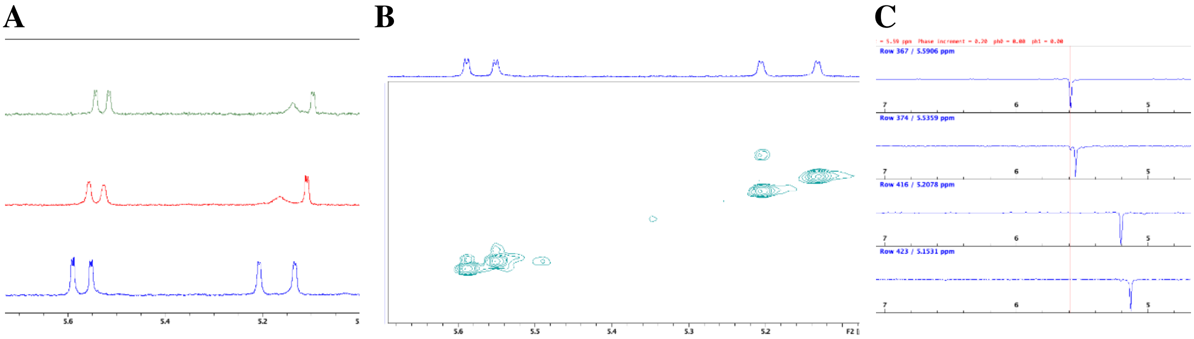

S1.99 A. VT experiment for **8** in DMSO-d_6 at 400 MHz with H_3 and H_4 region shown. Blue = 25 °C, red = 60 °C, green = 80°C. **B.** 2D NOESY studies for **8** at 25° in DMSO-d_6 at 400 MHz. **C.** EXSY outcomes for **7** at H_3 and H_4 region.

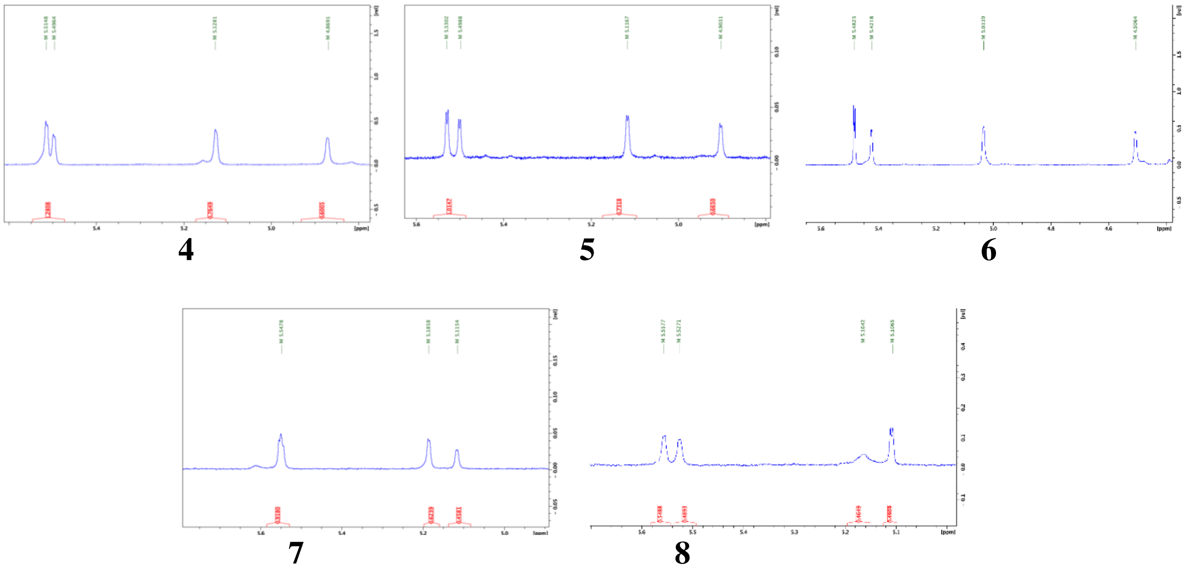

S1.100 400 MHz ^{1}H NMR spectra of expanded H_3 & H_4 region (δ 4.5-5.5 ppm) in DMSO-d_6 for diastereomers **4-8**.

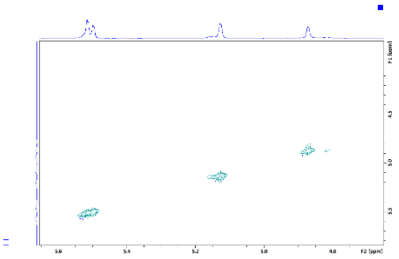

4

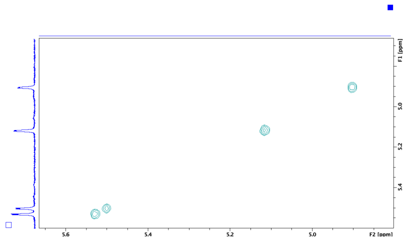

5

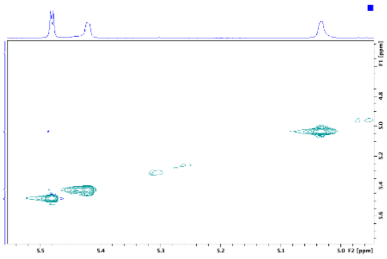

6

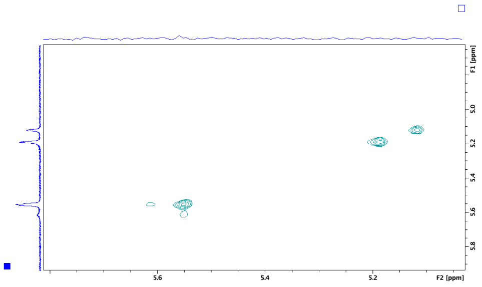

7

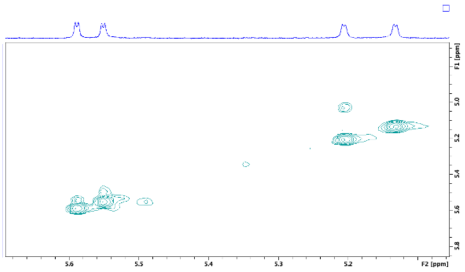

8

S1.101 400 MHz 2D NOESY spectra for H_3 and H_4 region in diastereomeric mixtures **4-8** in DMSO-d_6 at 25°C

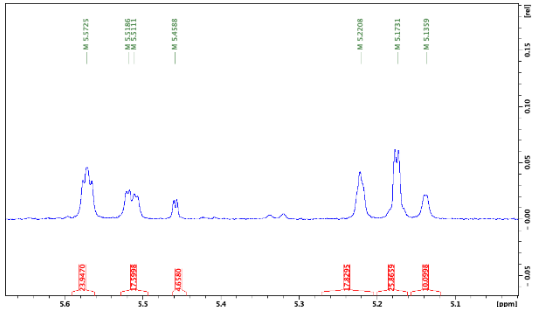

2, DMSO-d_6

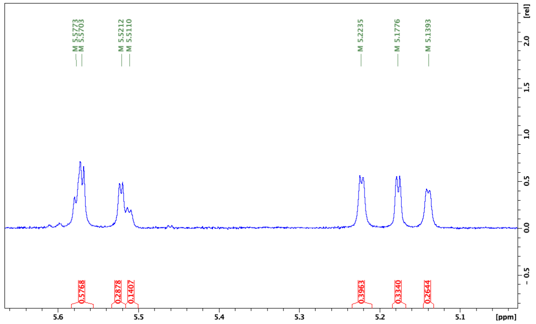

3, DMSO-d_6

S1.102: 400 MHz [1] H NMR spectra at 25°C of the H_3 & H_4 region (δ 4.4-5.5 ppm) for *N-(BOC)-D-* and *-L-*proline CDR diastereomer conjugates of **1 (with rotamers observed)** in DMSO-d_6 which have successfully been resolved using LC. (resonance signal overlap for diastereomers is noted).

S1.103 *Integration of H_3 and H_4 resonances on ^{1}H NMR axis for qNMR determination of de for resolved proline diastereomers* ***2DS1***, ***2DS2***, ***3DS1*** *and* ***3DS2*** *inclusive of expanded H_3 and H_4 region*

	Expansion of H_3 region	Expansion of H_4 region *
**2DS1**	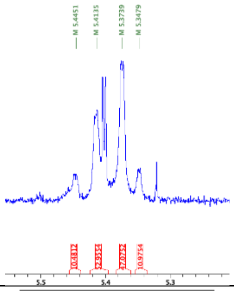	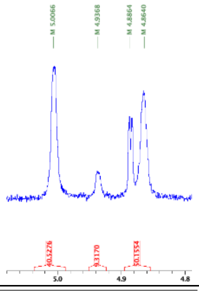
**2DS2**	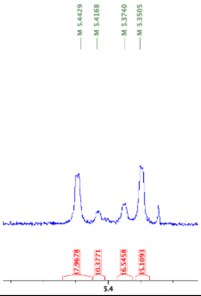	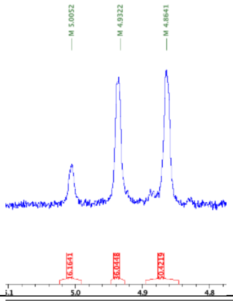
**3DS1**	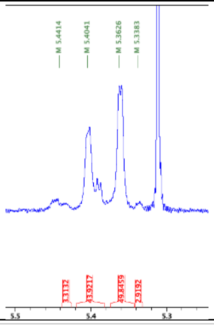	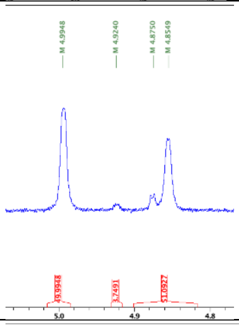
**3DS2**	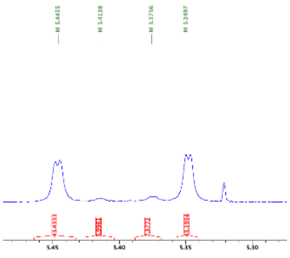	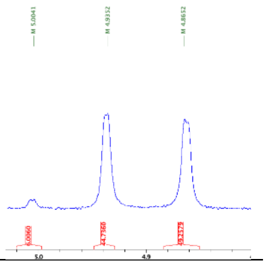

Appendix S2:1 HPLC data

SAMPLE INFORMATION

Sample Name:	01 en1	Acquired By:	System
Sample Type:	Unknown	Sample Set Name	CP run 318 0R 12
Vial:	4	Acq. Method Set:	conn
Injection #:	1	Processing Method	01 en1
Injection Volume:	5.00 ul	Channel Name:	2487Channel 1
Run Time:	10.0 Minutes	Proc. Chnl. Descr.:	254nm

Date Acquired: 8/31/2020 1:11:19 PM IST
Date Processed: 8/31/2020 3:54:45 PM IST

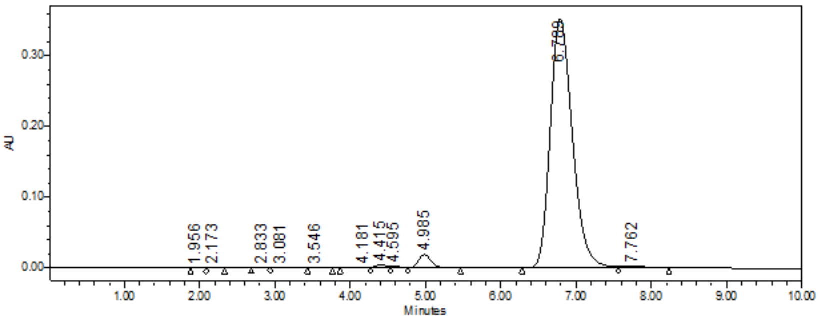

	RT	Area	% Area	Height
1	1.956	6974	0.09	1041
2	2.173	3198	0.04	472
3	2.833	4120	0.05	525
4	3.081	10519	0.14	885
5	3.546	2135	0.03	272
6	4.181	21384	0.28	1540
7	4.415	49371	0.65	4658
8	4.595	23746	0.31	2459
9	4.985	226887	2.97	19158
10	6.789	7229118	94.68	353181
11	7.762	58258	0.76	2479

S2.10 Chiral HPLC chromatogram of 9 (3-OH enantiomer of 3DS1)

SAMPLE INFORMATION

Sample Name:	01 en2	Acquired By:	System
Sample Type:	Unknown	Sample Set Name	CP run 318 0R 12
Vial:	6	Acq. Method Set:	conn
Injection #:	1	Processing Methoc	01 en2
Injection Volume:	5.00 ul	Channel Name:	2487Channel 1
Run Time:	10.0 Minutes	Proc. Chnl. Descr.:	254nm
Date Acquired:	8/31/2020 1:33:03 PM IST		
Date Processed:	8/31/2020 3:55:24 PM IST		

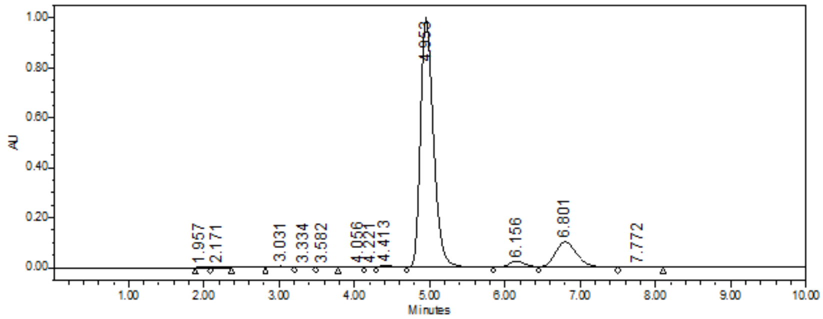

	RT	Area	% Area	Height
1	1.957	7036	0.05	1016
2	2.171	10553	0.07	1567
3	3.031	41083	0.28	4720
4	3.334	22860	0.15	2455
5	3.582	6821	0.05	862
6	4.056	38314	0.26	3795
7	4.221	34752	0.23	4149
8	4.413	131372	0.89	9797
9	4.953	12056248	81.29	999480
10	6.156	392206	2.64	24574
11	6.801	2062580	13.91	102379
12	7.772	26888	0.18	1342

S2.11 Chiral HPLC chromatogram of 10 (3-OH enantiomer of 3DS2)

Appendix S3: Computational Data

Computational Data and Details

Computational Details

All calculations reported in the manuscript were carried out using standard DFT methods as implemented in Gaussian 16.[SI1] All initial explorations of minima structures were performed at the wb97xD[SI2]/def2-SVP[SI3] level of theory in a solvent model SMD[SI4] at 298 K in order to mimic experimental conditions.

The characteristics of the intramolecular interactions were analysed by means of the Atoms in Molecules (QTAIM) theory.[SI5] For this purpose we have located the most relevant bond critical points (BCP), and evaluated the electron density at each of them, by using the QTAIMAll program.[SI6]

[SI1] M. J. Frisch, G. W. Trucks, H. B. Schlegel, G. E. Scuseria, M. A. Robb, J. R. Cheeseman, G. Scalmani, V. Barone, G. A. Petersson, H. Nakatsuji, X. Li, M. Caricato, A. V. Marenich, J. Bloino, B. G. Janesko, R. Gomperts, B. Mennucci, H. P. Hratchian, J. V. Ortiz, A. F. Izmaylov, J. L. Sonnenberg, Williams, F. Ding, F. Lipparini, F. Egidi, J. Goings, B. Peng, A. Petrone, T. Henderson, D. Ranasinghe, V. G. Zakrzewski, J. Gao, N. Rega, G. Zheng, W. Liang, M. Hada, M. Ehara, K. Toyota, R. Fukuda, J. Hasegawa, M. Ishida, T. Nakajima, Y. Honda, O. Kitao, H. Nakai, T. Vreven, K. Throssell, J. A. Montgomery Jr., J. E. Peralta, F. Ogliaro, M. J. Bearpark, J. J. Heyd, E. N. Brothers, K. N. Kudin, V. N. Staroverov, T. A. Keith, R. Kobayashi, J. Normand, K. Raghavachari, A. P. Rendell, J. C. Burant, S. S. Iyengar, J. Tomasi, M. Cossi, J. M. Millam, M. Klene, C. Adamo, R. Cammi, J. W. Ochterski, R. L. Martin, K. Morokuma, O. Farkas, J. B. Foresman and D. J. Fox in *Gaussian 16 Rev. B.01*, *Vol.* Wallingford, CT, **2016**.

[SI2] J.-D. Chai and M. Head-Gordon, *Phys. Chem. Chem. Phys.*, **2008**, 10, 6615-20

[SI3] A. Schaefer, C. Huber, and R. Ahlrichs, *J. Chem. Phys.*, **1994**, 100, 5829-35

[SI4] A. V. Marenich, C. J. Cramer, and D. G. Truhlar, *J. Phys. Chem. B*, **2009**, 113, 6378-96

[SI5] R. F. W. Bader, *Atoms in Molecules: A Quantum Theory*, Clarendon Press, Oxford, **1990**, p.

[SI6] T. A. Keith in *AIMAll, TK Gristmill Software (aim.tkgristmill.com)*, *Vol.* **2011**, pp. TK Gristmill Software,(aim.tkgristmill.com).

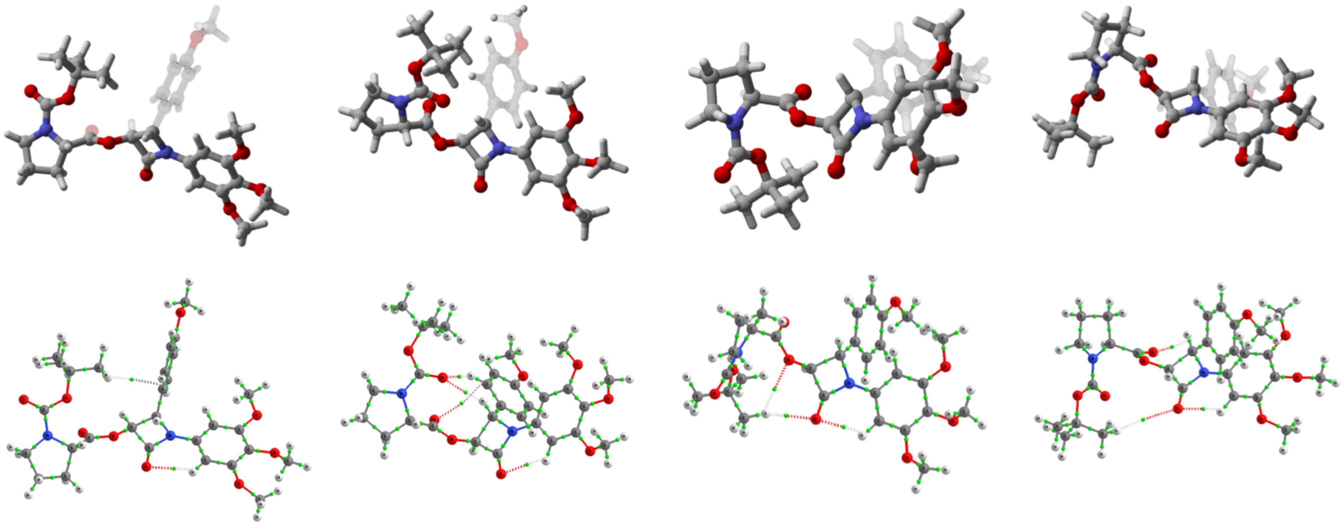

3DS1 major rotamer **3DS1** minor rotamer (a) **3DS1** minor rotamer (b) **3DS1** minor rotamer (c)

S3.1: Top: Conformational analysis for major and minor rotamers of **3DS1**. Bottom: Non-covalent intra molecular interactions for major and minor rotamers of **3DS1**.

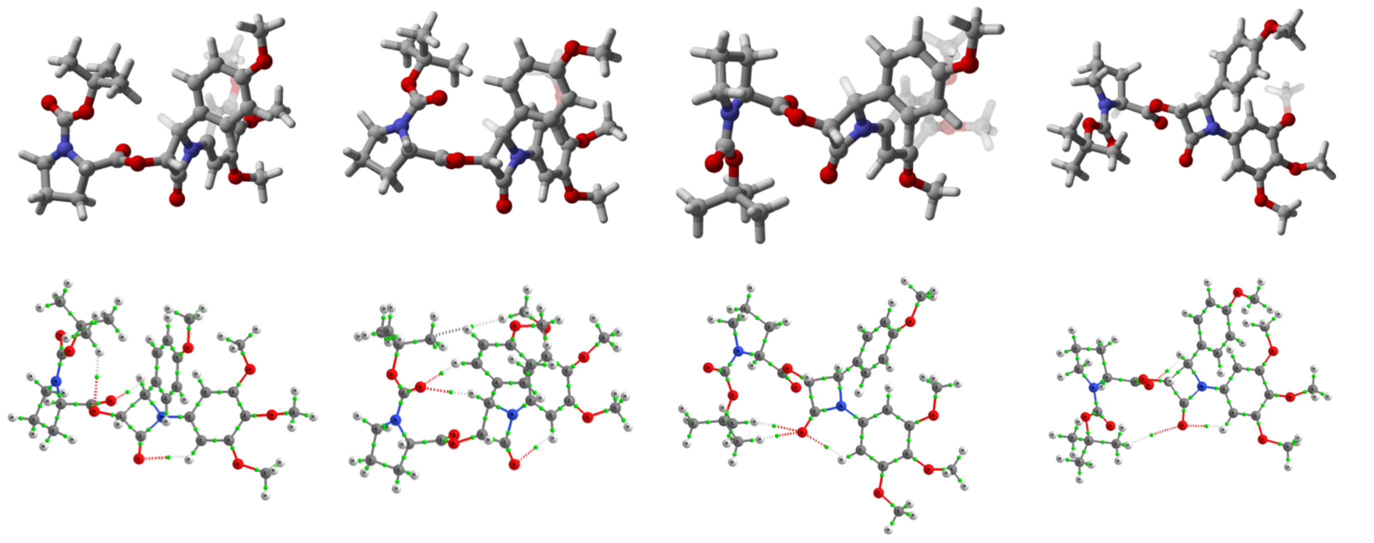

3DS2 major rotamer **3DS2** minor rotamer (a) **3DS2** minor rotamer (b) **3DS2** minor rotamer (c)

S3.2: Top: Conformational analysis for major and minor rotamers of **3DS2**. Bottom: Non-covalent intra molecular interactions for major and minor rotamers of **3DS2**.

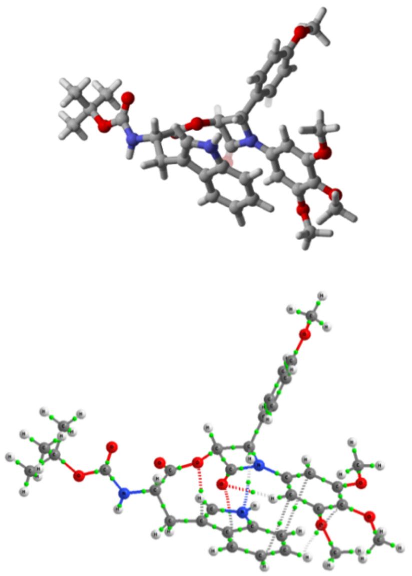

6DS1 major rotamer

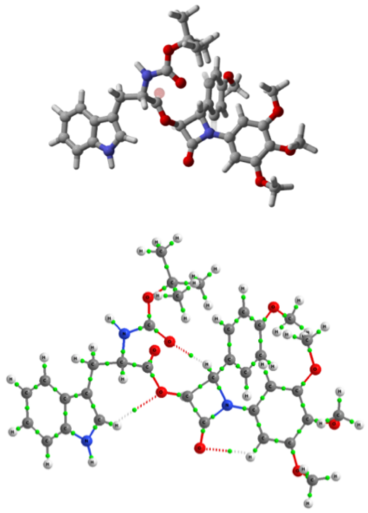

6DS1 minor rotamer

S3.4: Top: Conformational analysis for major and minor rotamer of **6DS1**. Bottom: Non-covalent intra molecular interactions for major and minor rotamers of **6DS1**.

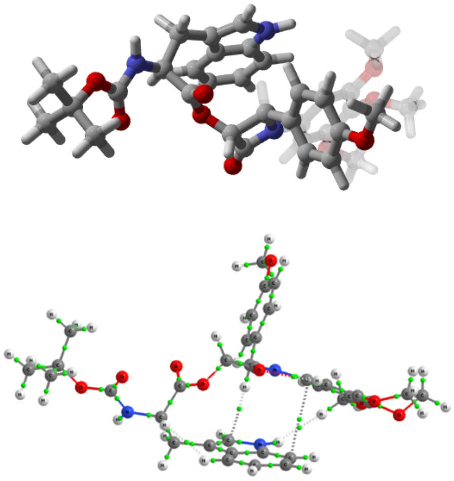

6DS2 major rotamer

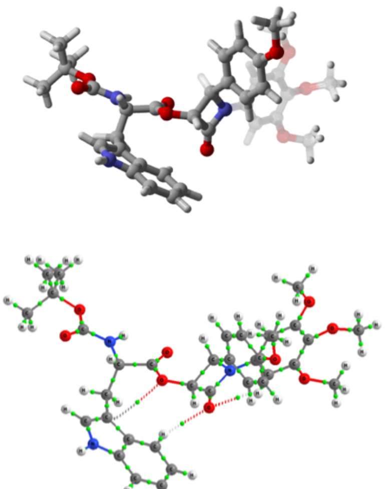

6DS2 minor rotamer

S3.5 Top: Conformational analysis for major and minor rotamer of **6DS2**. Bottom: Non-covalent intra molecular interactions for major and minor rotamers of **6DS2**.
rotamers of **6DS1** and **6DS2** in chiral resolving solvents MTBE and *n*-hexane

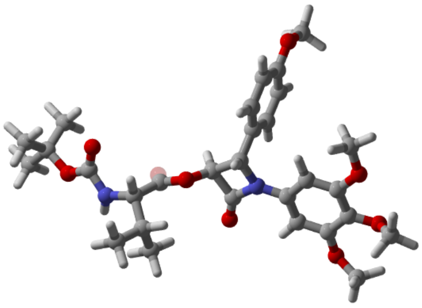

7DS1 major rotamer

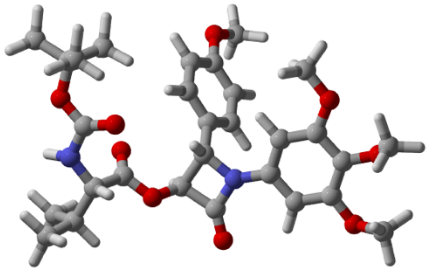

7DS1 minor rotamer

S3.6: Conformational analysis for major and minor rotamer of **7DS1**. Intramolecular non-covalent interactions were not observed.

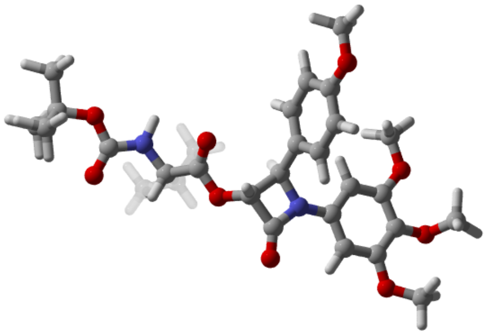

7DS2 major rotamer

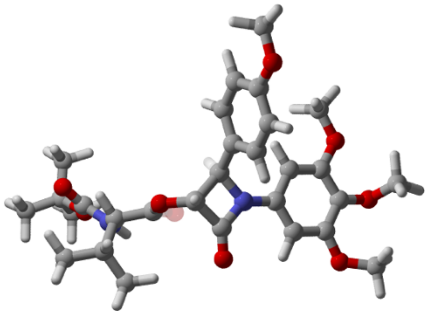

7DS2 minor rotamer

S3.7: Conformational analysis for major and minor rotamer of **7DS2**. Intramolecular non-covalent interactions were not observed.

S4: Characterisation of diastereomer mixtures 2 and 4-8

1-(*tert*-Butyl) 2-(2-(4-methoxyphenyl)-4-oxo-1-(3,4,5-trimethoxyphenyl)azetidin-3-yl) (*R*)-pyrrolidine-1,2-dicarboxylate (2)

Compound **2** was synthesised from **1** and N-(*tert*-butoxycarbonyl)-D-proline using General Method I. **Yield:** 200 mg (0.36 mmol) 52 %; **Appearance:** white amorphous powder/ gel; **m.p.:** 65-70 °C, **R_f:** 0.53 (*n*-hexane: ethyl acetate 1:1); **^{1}H NMR (600 MHz, $CDCl_3$): δ** 7.33-7.29 (m, 2H), 6.96-6.9 (m, 2H), (6.56, 6.55, 6.55, 6.55 (singlets, 2H)), 5.45 (apparent s, 0.2H), 5.41 (apparent s, 0.2H), 5.4 (d, 0.16H, *J* = 1.8 Hz), 5.4 (d, 0.3H, *J* = 1.8 Hz), 5.34 (d, 0.2H, *J* = 1.8 Hz), 5.00 (apparent singlet, 0.3H), 4.94 (apparent singlet. 0.3H), 4.89 (d, 0.2H, *J* = 1.8 Hz), 4.86 (apparent singlet, 0.35H), 4.42 – 4.37 (m, 1H), (3.85, 3.85, 3.84, 3.83, 3.82, 3.82 (singlets, 3H)), (3.79, 3.78, (singlets, 3H)), (3.72, 3.72 (singlets, 6H)), 3.66-3.40 (m, 2H), 2.36-2.25 (m, 1.5H), 2.1 – 1.9 (m, 3H), 1.52 (s, 3.5H), 1.47 (s, 2H), 1.45 (s, 3.5H);

^{13}C NMR (100 MHz, $CDCl_3$): δ 172.4, 172.3, 172.1, 172.0, 169.7, 161.7, 161.5, 161.4, 161.2, 160.3, 160.2, 160.1, 160.0, 157.7, 154.5, 153.6, 153.5, 135.1, 135.0, 134.9, 134.9, 133.1, 133.1, 133.0, 132.9, 128.0, 127.9, 127.8, 127.7, 127.7, 127.1, 127.0, 126.8, 114.7, 114.6, 114.6, 114.5, 95.5, 95.4, 95.3, 82.9, 82.8, 82.7, 82.5, 80.4, 80.2, 80.1, 65.9, 63.7, 63.6, 63.5, 63.4, 61.2, 61.0, 59.4, 59.1, 58.8, 58.7, 58.6, 56.3, 56.1, 55.4, 47.3, 46.7, 46.7, 46.4, 34.5, 31.1, 31.0, 29.0, 29.9, 28.5, 28.4, 28.4**; ^{1}H NMR (600 MHz, DMSO-*d_6*):** δ 7.44 (m, 1H), 7.41 (m, 1H), 6.97 (m, 2H), 6.56 (s, 0.35H), 6.54 (s, 0.5H), 6.53 (s, 1H), 5.57 (d, 0.2H, *J* = 1.8 Hz), 5.56 (d, 0.2H, *J* = 1.8 Hz), 5.51 (d, 0.2H, *J* = 1.8 Hz), 5.50 (d, 0.2H, *J* = 1.8 Hz), 5.45 (d, 0.2H, *J* = 1.8 Hz), 5.52 (apparent singlet, 0.4H), 5.17 (d, 0.4H, *J* = 1.8 Hz), 5.13 (apparent singlet, 0.3H), 4.32-2.29 (m, 1H), 3.75 (s, 3H), 3.63 (s, 6H), 3.58 (s, 3H), 2.32-2.23 (s, 2H), 2.03-1.78 (m, 4H), (1.42, 1.38, 1.35, 1.34 (singlets, 9H); **^{13}C NMR (100 DMSO-*d_6*): δ** 174.8, 174.4, 172.4, 172.3, 172.2, 172.0, 169.9, 162.0, 161.6, 161.5, 160.2, 160.1, 160.1, 154.1, 154.0, 154.0, 153.7, 153.2, 134.8, 132.8, 132.8, 132.7, 129.1, 129.0, 129.0, 129,0, 128.9, 127.9, 127.7, 127.6, 127.6, 12.6, 114.8, 96.1, 96.0, 96.0, 92.1, 92.1, 82.3, 82.2, 82.2, 82.1, 79.7, 79.7, 79.1, 65.4, 62.7, 62.6, 62.5, 62.5, 62.4, 61,0, 59.1, 58.8, 58.8, 58.8, 58.7, 58.7, 55.6, 46.9, 46.7, 30.9, 30.8, 29.8, 28.59, 28.5, 28.5, 28.4, 28.4 **HRMS (APCI):** *m/z* calcd for $C_{29}H_{36}N_2O_9$+Na^+: 579.2313 [*M*+Na]$^+$; found 579.2308. Purity RP-HPLC: 85 %

1-(*tert*-Butyl) 2-((2*S*,3*S*)-2-(4-methoxyphenyl)-4-oxo-1-(3,4,5-trimethoxyphenyl)azetidin-3-yl) (*R*)-pyrrolidine-1,2-dicarboxylate (2DS1). **2DS1** was isolated from **2** using gravity column chromatography over silica gel with a gradient of 4:1 to 1:4 *n*-hexane: TBME. **Yield:** 30 mg (0.054 mmol) 15 %, **Appearance:** white fluffy powder; **R_f:** 0.33 (*n*-hexane/TBME 4:6), plate developed twice; **^{1}H NMR (400 MHz, $CDCl_3$): δ** 7.31 (m, 2H), 6.93 (m, 2H), (6.56, 6.56, 6.55, (singlets, 2H)), 5.41 (d, 0.08H, *J* = 1.8 Hz), 5.4 (apparent singlet, 0.25H), 5.4 (d, 0.2H, *J* = 1.8 Hz), 5.4 (d, 0.3H, *J* = 1.8 Hz), 5.34 (d, 0.1H, *J* = 1.8 Hz), 5.0 (apparent singlet, 0.35H), 4.94 (apparent singlet, 0.1H), 4.89 (d, 0.2H, *J* = 1.8 Hz), 4.86 (apparent singlet, 0.3H), 4.39 (m, 1H), 3.83 (s, 1.7H), 3.81 (s, 1.3H), 3.79 (s, 2.2H), 3.78 (s, 1H), 3.72 (s, 3H), 3.71 (s, 3H), 3.62-3.4 (m, 2H), 2.35-2.2 (m,2H), 2.1-1.9 (m, 2H), 1.52 (s, 5H), 1.48 (s, 3H), 1.43 (s, 1H); **^{13}C NMR (100 MHz, $CDCl_3$): δ** 172.1, 171.9, 161.5, 161.2, 160.3, 160.0, 154.5, 153.6, 153.5, 153.6, 153.5, 153.4, 135.1, 134.9, 133.1, 133.0, 127.1, 126.8, 114.7, 114.6, 95.5, 95.4, 82.8, 82.7, 80.4, 80.1, 65.9, 63.7, 63.5, 63.4, 63.4, 61.0, 59.4, 59.2, 58.8, 58.5, 56.9, 56.1, 55.4, 46.7, 46.4, 31.1, 31.0, 29.9, 29.7, 28.5, 28.4, 24.6, 23.7;**^{1}H NMR (600 MHz, DMSO-*d_6*): δ** 7.44 (d, 1.4H,

J = 8.9 Hz), 7.42 (d, 0.6H, J = 8.9 Hz), 6.98 (m, 2H), 6.55 (s, 0.7H), 6.52 (s, 1.3H), 5.57 (s, 0.5H, J = 1.8 Hz), 5.51 (s, 0.08H, J = 1.8 Hz), 5.50 (d, 0.2H, J = 1.8 Hz), 5.5 (d, 0.1H, J = 1.8 Hz), 5.22 (d, 0.2H, J = 1.8 Hz), 5.17 (d, 0.7H, J = 1.8 Hz), 4.29 (m, 1H), 3.79 (s, 3H), 3.36 (s, 6H), 3.6 (s, 3H), 2.36-2.25 (m,2H), 1.98 -1.78 (m, 4H), 1.42 (s, 2.5H), 1.42 (s, 2H), 1.40 (s, 1H), 1.38 (s, 1H), 1.34 (s, 4H), 1.34 (s, 2H); **^{13}C NMR (100 DMSO-*d_6*): δ** 172.3, 161.5, 160.1, 153.4, 153.0, 147.6, 146.2, 136.4, 135.0, 132.8, 129.0, 114.7, 1025, 95.9, 82.3, 79.7, 62.6, 60.5, 59.3, 58.8, 56.3, 55.7, 46.8, 30.6, 28.6, 28.3, 23.7; **HRMS (APCI):** *m/z* calcd for $C_{29}H_{36}N_2O_9$+H$^+$: 555.2340 [*M*+H$^+$], found 555.2320. **Purity RP-HPLC:** 85%

1-(*tert*-Butyl)-2-((2*R*,3*R*)-2-(4-methoxyphenyl)-4-oxo-1-(3,4,5-trimethoxyphenyl)azetidin-3-yl) (*R*)-pyrrolidine-1,2-dicarboxylate (2DS2). **2DS2** was isolated from **2** using gravity column chromatography over silica gel with a gradient of 4:1 to 1:4 *n*-hexane/TBME. **Yield:** 10 mg (0.018 mmol) 5%, **Appearance:** white fluffy powder; **R_f:** 0.30 (*n*-hexane/TBME 4:6), plate developed twice; **^{1}H NMR (400 MHz, $CDCl_3$):** δ 7.30 (m, 2H), 6.89 (apparent triplet, 2H, J = 9.6 Hz), 6.56 (s, 2H), 5.4 (apparent singlet, 0.5H), 5.35 (apparent singlet, 0.5H), 5.1 (apparent singlet, 0.1H), 4.93 (apparent singlet, 0.3H), 4.86 (apparent singlet, 0.4H), 4.39 (m,1H), 3.84 (s, 2H), 3.82 (s, 1H), 3.78 (s, 3H), 3.72 (s, 6H), 3.62-3.4 (m, 2H), 2.39 – 1.89 (m, 4H), 1.53 (s, 3H), (1.48, 1.47 (singlets, 3H)), 1.43 (s, 5H); **^{1}H NMR (600 MHz, DMSO-*d_6*): δ** 7.45 (d, 1H, J = 8.6 Hz), 7.41 (1H, J = 8.9 Hz), 6.97 (d, 2H, J = 8.1 Hz), 6.54 (s, 0.3H), 654 (s, 0.9H), 6.53 (s, 0.8H), 6.55 (d, 0.5H, J = 1.8 Hz), 5.51 (d, 0.6H, J = 1.8 Hz), 5.21 (d, 0.5H, J = 1.8 Hz), 5.17 (d, 0.3H, J = 1.8 Hz), 5.14 (d, J = 0.3H), 4.32 (m, 1H), 3.73 (s, 3H), 3.63 (s, 6H), 3.58 (s, 3H), 2.33 -2.16 (m, 3H), 2.04-1.7 (m, 4H), 1.39 (s, 2H), 1.38 (s, 2H), 1.34 (s, 3H), 1.32 (s, 3H); **^{13}C NMR (100 DMSO-*d_6*): δ** 174.9, 174.4, 172.3, 172.0, 171.0, 161.7, 154.0 153.7, 153.6, 153.2, 134.9, 132.8, 132.7, 129.6, 129.1, 128.9, 127.6, 127.5, 114.7, 114.6, 114.7, 96.1, 96.0, 94.0, 92.2, 92.1, 82.2, 82.2, 79.7, 79.7, 78.9, 78.5, 65.4, 63.6, 62.8, 62.4, 60.6, 60.5, 59.1, 58.7, 57.3, 57.1, 56.2, 56.0, 46.8, 46.7, 46.7, 46.6, 37.2, 35.3, 28.5, 28.4, 28.4, 24.9;**IR (ATR): $\tilde{\nu}$** 1756 (β-lactam C=O) cm^{-1}, 1694 cm^{-1} (*N*-Boc-Proline C=O) **HRMS (APCI):** *m/z* calcd for $C_{29}H_{36}N_2O_9$-H$^+$: 555.2348 **[*M*-H$^+$]**; found 555.2317;. HRMS (**ESI**)**:** *m/z* calcd for $C_{29}H_{36}N_2O_9$+Na$^+$: 579.2313 **[*M*+Na$^+$]**; found 579.2318**. Purity RP-HPLC:** 95%

2-(4-Methoxyphenyl)-4-oxo-1-(3,4,5-trimethoxyphenyl)azetidin-3-yl (*tert*-butoxycarbonyl)-*L*-phenylalaninate (4). Diastereomeric mixture **4** was synthesised from **1** and *N*-(*tert*-butoxycarbonyl)-L phenylalanine using General Method I. **Yield:** 363 mg (0.6 mmol) 60% **R_f:** 0.5 (*n*-hexane/ethyl acetate 3:2); **m.p.:** 72 °C; **^{1}H NMR (400 MHz, $CDCl_3$):** δ 7.34-7.2 (m, 7H), 6.94 (d, 2H, J = 8.3 Hz), 6.6 (s, 0.2H), 6.56 (s, 0.8H), 6.53 (s, 1H), 5.4 (apparent s, 0.5H), 5.33 (apparent s, 0.5H), 5 (m, 1H), 4.87 (apparent s 0.2H), 4.7 (m, 1.6H), 3.83 (s, 3H), 3.8 (s, 3H), 3.73 (s, 6H), 3.18 (m, 2H), 1.45 (s, 3H), 1.43 (s, 6H); **^{13}C NMR (100 MHz, $CDCl_3$):** δ 171.1, 160.5, 153.6, 153.1, 129.6, 128.7, 127.5, 127.3, 127.0, 126.7, 126.7, 114.6, 95.5, 83.0, 82.8, 80.3, 68.2, 63.5, 63.3, 61.0, 56.1, 55.4, 28.2; **^{1}H NMR (600 MHz, DMSO- *d_6*):** 7.5 (d, 0.5H, J= 4.6 Hz), 7.48 (d, 0.5H, J= 4.6 Hz), 7.42 (d, 1H, J= 8.6 Hz), 7.39 (d, 1H, J= 8.6 Hz), 7.3-7.19 (m, 5H), 6.97 (d, 2H, J = 8.6 Hz), 6.54 (s, 1H), 6.5 (s, 1H), 5.51 (d, 0.5, J = 1.6 Hz), 4.98 (d, 0.5, J = 1.6 Hz), 5.13 (apparent singlet, 0.5H), 4.87 (apparent singlet 0.49 H), 4.3 (m, 1H), 3.75 (s, 3H), 3.64 (s, 6H), 3.58 (s, 3H), 3.09-2.93 (m, 2H), 1.34 (s, 4.5H), 1.33 (s, 3H); **^{13}C NMR (100 MHz, DMSO- *d_6*):** δ 171.9, 171.5, 161.6, 160.1, 156.0, 153.6, 137.8, 137.6,

134.8, 132.8, 132.7, 129.8, 129.0, 127.6, 127.1, 127.0, 114.7, 96.0, 95.9, 82.3, 79.0, 62.6, 60.5, 60.2, 56.3, 55.6, 55.5, 36.7, 36.6, 28.6, 21.2; **IR (ATR): *ν̃*** 1756 cm^{-1} (C=O, β-lactam), 1709 cm^{-1}; (*N*-Boc-L-proline C=O); **HRMS (ESI):** *m/z* calcd for $C_{33}H_{38}N_2O_9$+Na^+: 629.2469 **[*M*+Na+]**; found 629.2472 **Purity RP-HPLC:** 99%

2-(4-Methoxyphenyl)-4-oxo-1-(3,4,5-trimethoxyphenyl)azetidin-3-yl (((**9*H*-fluoren-9-yl) methoxy**) **carbonyl)-*L*-phenylalaninate** (**5**). Diastereomeric mixture **5** was synthesised from **1** and Fmoc- L-phenylalanine using General Method I. **Yield:** 226 mg (0.31 mmol) 31% **R_f:** 0.53 (*n*-hexane/ethyl acetate 3:2)**.** **m.p.:** 77 ^{O}C; **^{1}H NMR (400 MHz, $CDCl_3$):** δ 7.7 (t, 2H, *J* = 6.6 Hz), 7.6 (t, 2H, *J* = 6.6 Hz), 7.41 (m, 2H), 7.32 (m,5H), 7.2 (m, 2H), 6.93 (apparent d, 2H, *J* = 8.8 Hz), 6.54 (s, 0.7H), 6.49 (s, 1.3H), 5.44 (apparent singlet, 0.4H), 5.34 (apparent singlet. 0.4H), 5.3 (apparent multiplet, 0.4H), 5.23 (apparent multiplet, 0.4H), 4.87 (apparent singlet, 0.6H), 4.81 (m, 1H), 4.66 (apparent singlet, 0.5H), 4.47-4.35 (m, 2H), 4.23 35 (m, 1H), 3.83 (s, 2H), 3.82 (s, 1H), 3.8 (s, 3H), 3.72 (s, 3.5H), 3.71 (s, 2.5H), 3.23 (m, 2H); **^{13}C NMR (100 MHz, $CDCl_3$):** δ 171.8, 168.5, 167.8, 167.0, 164.0, 160.3, 153.5, 141.2, 139.4, 132.9, 129.5, 128.9, 128.0, 127.0, 125.3, 120.0, 114.6, 112.4, 95.4, 66.0, 61.1, 56.0, 55.2, 47.1; **^{1}H NMR (400 MHz, DMSO-*d*$_6$):** δ 8.06 (m, 1H, NH), 7.86 (d, 2H, *J* = 8 Hz), 7.63 (d, 2H, *J* = 8 Hz), 7.39 (m, 4H), 7.29 (m, 5H), 7.23 (m, 1H), 6.95 (d, 2H, *J* = 8 Hz), 6.49 (s, 1H), 6.49 (s, 1H), 5.53 (apparent singlet, 0.6H), 5.5 (apparent singlet, 0.3H), 5.2 (apparent s, 0.5H), 4.9 (apparent singlet, 0.5H), 4.4 (m, 1H), 4.31-4.17 (m, 3H), 3.75 (s, 3H), 3.61 (s, 3H), 3.60 (s, 2H), 3.57 (s, 3H), 3.56 (s, 1H), 3.14 (apparent dd, 1H, *J*= 5 Hz, *J* = 14 Hz), 3.00 (m, 1H); **^{13}C NMR (100 MHz, DMSO-*d*$_6$):** δ 171.5, 171.16, 170.8, 161.5, 160.0, 156.4, 153.6, 144.1, 141.2, 137.9, 137.7, 134.8, 132.8, 129.8, 129.6, 128.8, 128.0, 127.5, 127.1, 127.0, 125.6, 121.8, 120.6, 114.7, 95.9, 82.4, 82.3, 66.2, 62.5, 60.5, 60.2, 56.2, 55.7, 55.6, 47.0, 31.2, 31.0; **^{15}N NMR (60.8 MHz, DMSO-*d*$_6$):** δ 88.5 (NH); **IR (ATR):** *ν̃* 1756 cm^{-1} (C=O, β-lactam), 1709 cm^{-1} (C=O); **HRMS (ESI):** *m/z* calcd for $C_{43}H_{40}N_2O_9$+Na^+: 751.2626 **[*M*+Na$^+$]**; found 751.2644 **Purity RP-HPLC:** 87%

2-(4-Methoxyphenyl)-4-oxo-1-(3,4,5-trimethoxyphenyl)azetidin-3-yl (***tert*-butoxycarbonyl)-L-tryptophanate** (**6**). Diastereomeric mixture **6** was synthesised from **1** and *N*-(*tert*-butoxycarbonyl)-L-tryptophan using General Method I. **Yield:** 250 mg (0.38 mmols); 59% **Appearance:** white powder; **m.p.:** 87-92 °C; **^{1}H NMR (600 MHz, $CDCl_3$):** δ 8.22 (bs, 0.5H), 8.2 (bs, 0.5H), 7.63 (d, 0.5H, *J* = 8.6 Hz), 7.6 (d, 0.5H, *J* = 8.6 Hz), 7.38 (s, 0.5H), 7.36 (s, 0.5H), 7.24 (d, *J* = 8.6 Hz), 7.22 – 7.14 (m, 3H), 7.1 – 7.0 (m, 2H), 6.91 (d, 1H, *J* = 8.6 Hz), 6.88 (d, 1H, *J* = 8.6 Hz), 6.54 (s, 1H), 6.45 (s, 1H), 5.4 (apparent s, 0.6H), 5.23 (apparent s, 0.5H), 5.2 (apparent d, 0.4H *J* = 7.5 Hz, α NH,), 5.1 (d, 0.5H, *J* = 7.5 Hz, α NH), 4.78 (m, 1H), 4.36 (apparent s, 0.5H), 3.8 (s, 1H), 3.8 (s, 1.5H), 3.79 (s, 1H), 3.79 (s, 2H), 3.72 (s, 3H), 3.71 (s, 3H), 3.38 (m, 2H), 1.46 (s, 4.5H), 1.44 (s, 4.5H); **^{13}C NMR (100 MHz, $CDCl_3$):** δ 171.2, 171.0, 161.3, 161.0, 160.2, 153.3, 153.4, 136.1, 136.0, 135.1, 135.0, 132.9, 127.7, 127.6, 126.9, 126.8, 123.3, 122.3, 119.8, 119.8, 118.8, 118.7, 114.6, 114.5, 111.3, 95.4, 83.1, 82.6, 80.2, 60.4, 56.0, 56.0,5z5.3, 54.5, 54.2, 28.3, 27.7, 21.1, 14.2; **^{15}N NMR (60.8 MHz, $CDCl_3$)**: δ 125.3, 123.3, 86.8; **^{1}H NMR (400 MHz, DMSO-*d*$_6$):** δ 10.95 (s, 0.5H), 10.91 (s, 0.5H, NH), 7.54 (d, 0.5H, *J* = 7.8 Hz), 7.52 (d, 0.5H, *J* = 7.8 Hz), 7.47 (d, 0.5H, *J* = 7.2 Hz), 7.42 (d, 0.5H, *J* = 7.2 Hz), 7.34 (m, 2H), 7.24 (m,

2H), 7.07 (m, 1H), 7.02-6.97 (m, 1H), 6.95 (d, 1H, *J* = 8 Hz), 6.93 (d, 1H, *J* = 8 Hz), 6.53 (s, 1H), 6.43 (s, 1H), 5.47 (d, 0.5H, *J* = 1.8 Hz), 5.42 (apparent singlet, 0.4H), 5.03 (apparent singlet, 0.5H), 4.5 (apparent singlet, 0.4H), 4.33 (m, 1H), 3.75 (s, 1H), 3.74 (s, 2H), 3.64 (s, 6H), 3.58 (s, 3H), 3.2-3.09 (m, 2H), 1.36 (s, 4.5H), 1.34 (s, 4.5H); **^{13}C NMR (100 MHz, DMSO- d_6):** δ 172.1, 171.7, 170.7, 161.7, 160.0, 155.9, 153.6, 136.0, 136.5, 134.8, 132.8, 132.7, 128.9, 128.8, 124.6, 124.4, 121.5, 119.0, 118.9, 118.6, 118.5, 114.7, 111.9, 109.8, 109.6, 100.3, 96.0, 82.2, 82.0, 79.0, 62.6, 60.5, 60.2, 56.2, 55.6, 55.1, 28.6, 27.0, 21.2; **^{15}N NMR (60.8 MHz, DMSO-d_6)**: δ 134.9, 91.4, 89.9 **IR (ATR):** $\tilde{\nu}$ 1730 cm^{-1} (tryptophan C=O), 1757 cm^{-1} (C=O, β-lactam); **HRMS (APCI):** *m/z* calcd for $C_{35}H_{39}N_3O_9$-H^+: 644.2613 **[M - H$^+$]**; found 644.2619 **Purity RP-HPLC:** 99%

2-(4-Methoxyphenyl)-4-oxo-1-(3,4,5-trimethoxyphenyl)azetidin-3-yl (*tert*-butoxycarbonyl)-L-valinate (7). Diastereomeric mixture **7** was synthesised from **1** and *N*-(*tert*-butoxycarbonyl)-L-valine using General Method I. **Yield:** 329 mg (0.58 mmol) 58%, white solid; **R_f:** 0.38 (*n*-hexane/TBME 1:4); **m.p.:** 78-83 ^{0}C; **^{1}H NMR (400 MHz, $CDCl_3$):** δ 7.32 (d, 1H, *J* = 8.3 Hz), 7.29 (d, 1H, *J* = 8.3 Hz), 6.95 (d, 1H, *J* = 8.6 Hz), 6.94 (d, 1H, *J* = 8.6 Hz), 6.56 (s, 1H), 6.55 (s, 1H), 5.45 (d, 0.6 H, *J* = 1.8 Hz), 5.35 (apparent singlet, 0.3H), 5.03 (apparent m, 1, NH), 4.95 (apparent singlet, 0.6H), 4.88 (apparent singlet, 0.5H), 4.34 (m, 1H), 3.84 (s, 1.5H), 3.83 (s, 1.5H), 3.79 (s, 1.5H), 3.78 (s, 1.5H), 3.73 (s, 3H), 3.72 (s, 3H), 2.23 (m, 1H), 1.48 (s, 6H), 1.46 (s, 3H), 1.04 (d, 1.5H, *J*= 6.6 Hz), 1.03 (s, 1.5H, *J*= 6.6 Hz), 0.98 (d, 1.5H, *J*= 6.6 Hz), 0.96 (d, 1.5 H, *J*= 6.6 Hz); **^{13}C NMR (100 MHz, $CDCl_3$):** δ 171.4, 161.2, 160.2, 155.8, 134.8, 131.0, 128.8, 127.8, 126.7, 114.5, 95.3, 83.0, 82.5, 80.1, 65.7, 63.3, 61.06, 58.8, 58.5, 56.1, 55.3, 30.9, 28.2, 19.1, 17.9, 17.4, 15.3; **^{1}H NMR (400 MHz, DMSO-d_6):** δ 7.42 (m, 1H), 7.38 (m, 1H), 6.55 (s, 1.3H), 6.54 (s, 0.7H), 5.55 (apparent m, 1H), 5.89 (apparent singlet, 0.6H), 5.12 (apparent singlet, 0.4H), 3.95 (m, 1H), 3.73 (s, 3H), 3.63 (s, 6H), 3.57 (s, 6H), 2.1 (m, 1H), 1.40 (s, 4.5H), 1.38 (s, 4.5H), 0.94 (m, 6H); **^{13}C NMR (100 MHz, DMSO- d_6):** δ 1718, 171.6, 162.0, 153.7, 134.8, 132.9, 129.0, 127.7, 114.7, 96.0, 79.0, 62.5, 60.6, 59.8, 56.3, 55.6, 38.7, 30.1, 29.9, 28.7, 28.6, 19.5, 19.4, 19.1, 18.9;**IR (ATR)**: $\tilde{\nu}$ 1757 cm^{-1} (C=O, β-lactam); **HRMS (ESI):** ***m/z*** calcd for $C_{29}H_{38}N_2O_9$+Na^+: 581.2469 **[*M*+Na$^+$]**; found 581.2479 **Purity RP-HPLC:** 96%

2-(4-Methoxyphenyl)-4-oxo-1-(3,4,5-trimethoxyphenyl)azetidin-3-yl (((9*H*-fluoren-9-yl) methoxy) carbonyl)-L-valinate (8). Diastereomer mixture **8** was synthesised from **1** and Fmoc-L-valine using General Method I. **Yield:** 370 mg, (0.54 mmol) 54%, white solid; **R_f:** 0.38 (*n*-hexane/TBME 1:4); **m.p.:** 78-83 ^{0}C; **^{1}H NMR (400 MHz, $CDCl_3$) :** δ 7.78 (t, 2H, *J* = 7.6 Hz), 7.642 (m, 2H, *J* = 7.6 Hz), 7.41 (complex multiplet, 2H), 7.32 (m, 5H), 6.93 (apparent triplet, 2H, *J*= 8 Hz), 6.55 (s, 0.91H), 6.52 (s, 1.1), 5.47 (apparent s, 0.4H), 5.38 (d, 0.4H, *J* = 1.8 Hz), 5.30 (apparent s, 0.3H), 5.29 (apparent s, 0.4H), 4.94 (apparent s, 0.5H), 4.88 (apparent s, 0.5H), 4.49-4.36 (m, 3H), 4.25 (m, 1H), 3.83 (s, 1.5H), 3.82 (s, 1.5H), 3.79 (s, 1.5H), 3.78 (s, 1.5H), 3.72 (s, 3H), 3.69 (s, 3H), 2.3 (m, 1H), 1.0-0.98 (m, 6H); **^{13}C NMR (100 MHz, $CDCl_3$):** δ 176.9, 175.9, 161.5, 153.4, 143.9, 141.4, 133.0, 129.9, 127.7, 127.1, 120.0, 114.7, 95.5, 95.2, 67.1, 61.1, 60.4, 56.1, 55.4, 47.0, 30.9, 21..0. 19.1, 17.9, 14.2; **^{1}H NMR (400 MHz, DMSO-d_6):** δ 7.69 (d, 1H, NH, *J* = 7.7 Hz), 7.83 (m, 2H), 7.73 (m, 1H), 7.41 (m, 3H), 7.32 (m, 2H), 6.94 (d, 2H), 6.54 (s, 1H), 6.51 (s, 1H), 5.59 (d, 0.5H, *J* = 1.8 Hz), 5.55 (d, 0.5H, *J* = 1.8 Hz), 5.21 (d, 0.5H, *J* = 1.8 Hz), 5.14 (d, 0.5H, *J* = 1.8 Hz), 4.31 (m, 1H), 4.24 (m, 1H), 4.08 (m, 1H), 3.74

(s, 3H), 3.62 (s, 3H), 3.60 (s, 3H), 3.57 (s, 3H), 2.15 (s, 1H), 0.96 (m, 6H); **^{13}C NMR** (**100 MHz**, **DMSO-** ***d*$_6$**)**:** δ 171.5, 167.7, 160.3, 144.4, 141.3, 132.6, 129.4, 129.0, 128.0, 127.6, 125.8, 121.9, 120.7, 114.9, 96.0, 82.1, 66.5, 65.4, 62.5, 60.6, 60.3, 60.0, 56.3, 55.7, 47.1, 30.3, 21.8, 21.3; **IR** (**ATR**)**:** $\tilde{\nu}$ 1757 cm^{-1} (C=O, β-lactam), 1730 cm^{-1} (valine C=O); **HRMS (ESI):** *m/z* calcd for $C_{39}H_{40}N_2O_9$+Na^+: 703.2626 **[*M*+Na$^+$]**; found 703.2643. **Purity RP-HPLC:** 70 %
